# Corrigendum: Whole genome analysis of a schistosomiasis-transmitting freshwater snail

**DOI:** 10.1038/ncomms16153

**Published:** 2017-08-23

**Authors:** Coen M. Adema, LaDeana W. Hillier, Catherine S. Jones, Eric S. Loker, Matty Knight, Patrick Minx, Guilherme Oliveira, Nithya Raghavan, Andrew Shedlock, Laurence Rodrigues do Amaral, Halime D. Arican-Goktas, Juliana G. Assis, Elio Hideo Baba, Olga L. Baron, Christopher J. Bayne, Utibe Bickham-Wright, Kyle K. Biggar, Michael Blouin, Bryony C. Bonning, Chris Botka, Joanna M. Bridger, Katherine M. Buckley, Sarah K. Buddenborg, Roberta Lima Caldeira, Julia Carleton, Omar S. Carvalho, Maria G. Castillo, Iain W. Chalmers, Mikkel Christensens, Sandra Clifton, Celine Cosseau, Christine Coustau, Richard M. Cripps, Yesid Cuesta-Astroz, Scott F. Cummins, Leon Di Stefano, Nathalie Dinguirard, David Duval, Scott Emrich, Cédric Feschotte, Rene Feyereisen, Peter FitzGerald, Catrina Fronick, Lucinda Fulton, Richard Galinier, Sandra G. Gava, Michael Geusz, Kathrin K. Geyer, Gloria I. Giraldo-Calderón, Matheus de Souza Gomes, Michelle A. Gordy, Benjamin Gourbal, Christoph Grunau, Patrick C. Hanington, Karl F. Hoffmann, Daniel Hughes, Judith Humphries, Daniel J. Jackson, Liana K. Jannotti-Passos, Wander de Jesus Jeremias, Susan Jobling, Bishoy Kamel, Aurélie Kapusta, Satwant Kaur, Joris M. Koene, Andrea B. Kohn, Dan Lawson, Scott P. Lawton, Di Liang, Yanin Limpanont, Sijun Liu, Anne E. Lockyer, Ty Anna L. Lovato, Fernanda Ludolf, Vince Magrini, Donald P. McManus, Monica Medina, Milind Misra, Guillaume Mitta, Gerald M. Mkoji, Michael J. Montague, Cesar Montelongo, Leonid L. Moroz, Monica C. Munoz-Torres, Umar Niazi, Leslie R. Noble, Francislon S. Oliveira, Fabiano S. Pais, Anthony T. Papenfuss, Rob Peace, Janeth J. Pena, Emmanuel A. Pila, Titouan Quelais, Brian J. Raney, Jonathan P. Rast, David Rollinson, Izinara C. Rosse, Bronwyn Rotgans, Edwin J. Routledge, Kathryn M. Ryan, Larissa L. S. Scholte, Kenneth B. Storey, Martin Swain, Jacob A. Tennessen, Chad Tomlinson, Damian L. Trujillo, Emanuela V. Volpi, Anthony J. Walker, Tianfang Wang, Ittiprasert Wannaporn, Wesley C. Warren, Xiao-Jun Wu, Timothy P. Yoshino, Mohammed Yusuf, Si-Ming Zhang, Min Zhao, Richard K. Wilson

Nature Communications 8: Article number: 15451 ; DOI: 10.1038/ncomms15451 (2017); Published 05 16 2017; Updated 08 23 2017

The original version of this Article contained an error in the spelling of the author Leon Di Stefano, which was incorrectly given as Leon di Stephano. This has now been corrected in both the PDF and HTML versions of the Article.

