# Supplementary material for: Whole genome analysis of a schistosomiasis-transmitting freshwater snail
Source: Nat Commun. 2017 May 16;8:15451. doi: 10.1038/ncomms15451 (PMC5440852; doi:10.1038/ncomms15451)
Supplement: Supplementary Information — Supplementary Figures, Supplementary Notes and Supplementary References [file ncomms15451-s1.pdf]

TABLE OF CONTENTS SUPPLEMENTARY INFORMATION

SUPPLEMENTARY FIGURES

1 Protocol for spreading of *B. glabrata* chromosome 4
2 Karyotype of *B. glabrata* and ideograms of chromosomes........ 5
3 Fluorescence *in situ* hybridization on *B. glabrata* chromosomes 6
4. SNV distribution among *B. glabrata* genes. 7
5. Distribution of genes and genes with SNVs across KEGG Orthology (KO) categories. 7
6. GO term analysis of secretome. 8
7. Secretome and tissue-specific expression 9
8. Tissue-specific gene expression in *B. glabrata* 10
9. Tissue/organ-specific expression in *B. glabrata* 11
10. GPCRs identified from the *B. glabrata* genome 14
11. GPCRs clustered on LGUN_random_Scaffold39........ 15
12. Phylogenetic tree of commensal and cloned 16S sequences and other mollicutes 16
13. Genome organization of viruses associated with *Biomphalaria glabrata*. 17
14. Gene structure of the *B. glabrata* heat shock protein (HSP) families 18
15. The number of Nimbus (BgI) elements and functional domains in the genome 19
16. Phylogenetic tree of the heat shock protein (HSP) families........ 20
17. Pathogen-specific expression regulation of HSP70 and *Nimbus* in *B. glabrata* 21
18. Proteomics recovery of GREP-related peptides 22
19. Expression of GREP-like sequences 23
20. Proteomics annotation of HSP70 family proteins 24
21. Proteomics annotation of HSP60 family proteins 25
22. Proteomics annotation of HSP90 family proteins 26
23. TLR signaling pathway in *B. glabrata*........ 27
24. FREP domain structure 28
25. Intron-exon structures of gastropod FREP genes........ 29
26. Phylogenetic tree FREPs........ 30
27. Alignment FREP sequences 31
28. *B. glabrata* C1q-1 amino acid alignment 40
29. Phylogenetic tree Complement C3 sequences........ 41
30. Apoptotic signaling pathways in *Biomphalaria*. 42
31. Alignment of full length caspase proteins from *B. glabrata* 43
32. Bcl2-family members in *B. glabrata*........ 44
33. Production and metabolism of reactive oxygen species and nitric oxide........ 45
34. *B. glabrata* assembly lacks common AMPs 46
35. CpG observed/expected (o/e) ratio in coding regions. 47
36. LG1i_random_Scaffold7, sequence content and CpG composition 48
37. CpG observed/expected (o/e) ratio in repetitive regions........ 49
38. Western Blots show histone modifications 50
39. ClustalW alignment of amino-acid sequences of histones H3 and H4 51
40. Secondary structures of the *B. glabrata* pre-miRNA sequences........ 52
41. Weblogo of *B. glabrata* mature miRNAs 53
42. Alignment of bgl-mir-100 with animal homologs. 54
43. Phylogenetic tree of bgl-mir-100 with animal homologs and mir-99 precursors. 55
44. Alignment of the bgl-mir-1a with homologs from Bilateria 56

45. Phylogenetic tree of bgl-mir-1a and homologs from Bilateria ... 57
46. Alignment of bgl-mir-137 with homologs from Bilateria ... 58
47. Phylogenetic tree of bgl-mir-137 and homologs from Bilateria ... 59
48. Alignment of bgl-mir-216a and bgl-mir-216b with homologs from Bilateria ... 60
49. Phylogenetic tree of mir-216 family, bgl-mir-216a/216b, and homologs from Bilateria and mir-283 homologs ... 61
50. Alignment of bgl-mir-29a, bgl-mir-29b with homologs from Bilateria ... 62
51. Phylogenetic tree of bgl-mir-29a and bgl-mir-29b with homologs from Bilateria and mir-285 homologs ... 63
52. Alignment of bgl-mir-2001 with homologs from Bilateria,secondary structure of mature miRNAs ... 64
53. Alignment of bgl-mir-252a and bgl-mir-252b with homologs from Bilateria, secondary structure of mature miRNAs ... 65
54. RNA secondary structure of the cluster bgl-mir-71/2a-1/2d/2b/2a-2 ... 66
55. Alignment of the bgl-mir-1175 with homologs from Protostomia ... 67
56. Phylogenetic tree of bgl-mir-1175 and homologs from Protostomia ... 68
57. Alignment of bgl-mir-279 with homologs from Protostomia ... 69
58. Phylogenetic tree of bgl-mir-279 and homologs from Protostomia ... 70
59. Alignment of bgl-mir-750 with homologs from Protostomia ... 71
60. phylogenetic tree of bgl-mir-750 and homologs from Protostomia ... 72
61. Alignment of bgl-mir-1990 with homologs from Lophotrochozoa and secondary structure highlighting the mature miRNAs ... 73
62. Alignment of bgl-mir-1992 with homologs from Lophotrochozoa and secondary structure highlighting the mature miRNAs ... 74
63. RNA secondary structure of the cluster bgl-mir-745a/745b ... 75
64. Alignment of bgl-mir-1986 with homologs from Mollusca and secondary structure highlighting the mature miRNAs ... 76
65. Alignment of *B. glabrata* mature miRNAs with orthologs from *L. gigantea* ... 77
66. Predicted binding sites of novel microRNAs from *B. glabrata*. ... 80
67. Precision-recall on *Anolis carolinensis* hold-out data ... 81
68. Characterisation of five *B. glabrata* neuropeptides ... 82
69.Phylogenetic tree and gene structure of ovipostatins from *B. glabrata* ... 84
70. Steroidogenesis ... 85
71. ePKs of *B. glabrata*, *S. mansoni* and *Homo sapiens*. ... 86
72. Phylogenetic tree of the protein serine/threonine phosphatases of *B. glabrata*. ... 87
73. Phylogenetic tree of the protein tyrosine phosphatases (PTPS) of *B. glabrata*. ... 87
74. Phylogenetic tree of the dual specificity phosphatases (DUSPs) of *B. glabrata*. ... 88
75. Four tyrosinases map to LGUN_random_Scaffold2224 ... 89
76. Ranked high-copy TE abundance ... 90
77. Taxonomic diversity of interspersed repeats by major class ... 90
78. Evolutionary profile of Nimbus LINE ... 91

SUPPLEMENTARY NOTES

1. Karyotype of the snail *Biomphalaria glabrata* ... 92
2. *B. glabrata* linkage mapping ... 94
3. RNAseq mapping, variant calling for annotated proteome and secretome ... 95
4. Tissue-specific expression (RPKM) in *B. glabrata*. ... 99

5. Proteomic characterization of released proteins 100
6. GPCRs, putative receptors for aquatic odor perception 102
7. Genome of a mycoplasma or related mollicute associated with *B. glabrata* 103
8. Virus discovery from *B. glabrata*.. 105
9. HSPs, annotation and expression in *B. glabrata* 107
10. Proteogenomic annotation of stress- and anti-parasite response genes of *B. glabrata* 110
11. Cytochrome P450 (CYP) genes of *B. glabrata* 113
12. Pattern recognition receptors (PRR) and cytokines of *B. glabrata* 115
13. Toll-like receptor (TLR) signaling pathway in *B. glabrata* 119
14. *B.glabrata* FREP genes 120
15. Complement-like sequences of *B. glabrata* 122
16. The *Biomphalaria* apoptotic network... 125
17. Production of reactive oxygen species and protection against oxidative damage 127
18. Antimicrobial peptides and proteins in *B. glabrata* 130
19. Characterizing unknown transcripts from *B. glabrata* 133
20. Epigenetic toolbox of *B. glabrata* 134
21. miRNA/piRNA pathway genes and conserved miRNAs in *B.glabrata* 137
22. Identification of novel *B. glabrata* miRNA, prediction of associated targets......................... 142
23. Potential circadian clock genes of *B. glabrata* 145
24. Major neurohormones in *B. glabrata* 147
25. Ovipostatin-like male accessory gland protein in *B. glabrata*. 148
26. Endocrine mechanisms in *B. glabrata* 149
27. Eukaryotic protein kinases (Epks) of *B. glabrata* 151
28. Protein Phosphatases in the *B. glabrata* genome 152
29. Evolutionary conservation of cardiac specification genes in *B. glabrata* 153
30. Expression and evolution of actin genes in *B. glabrata* 155
31. Biomineralization genes of *B. glabrata* 157
32. Repeat and transposable element composition of the *B. glabrata* genome 158
33: *Biomphalaria glabrata*, sequence similarities with African *Biomphalaria pfeifferi* 160

Supplementary references 1-322 161

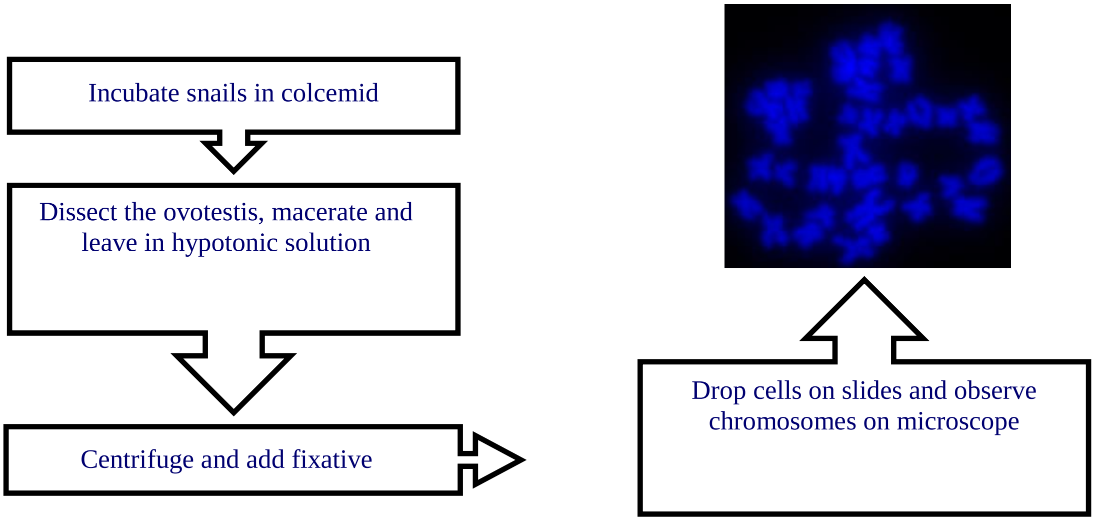

Supplementary Figure 1 *Biomphalaria glabrata* chromosome preparation. A description of the steps to attain well-spread *B. glabrata* mitotic chromosomes.

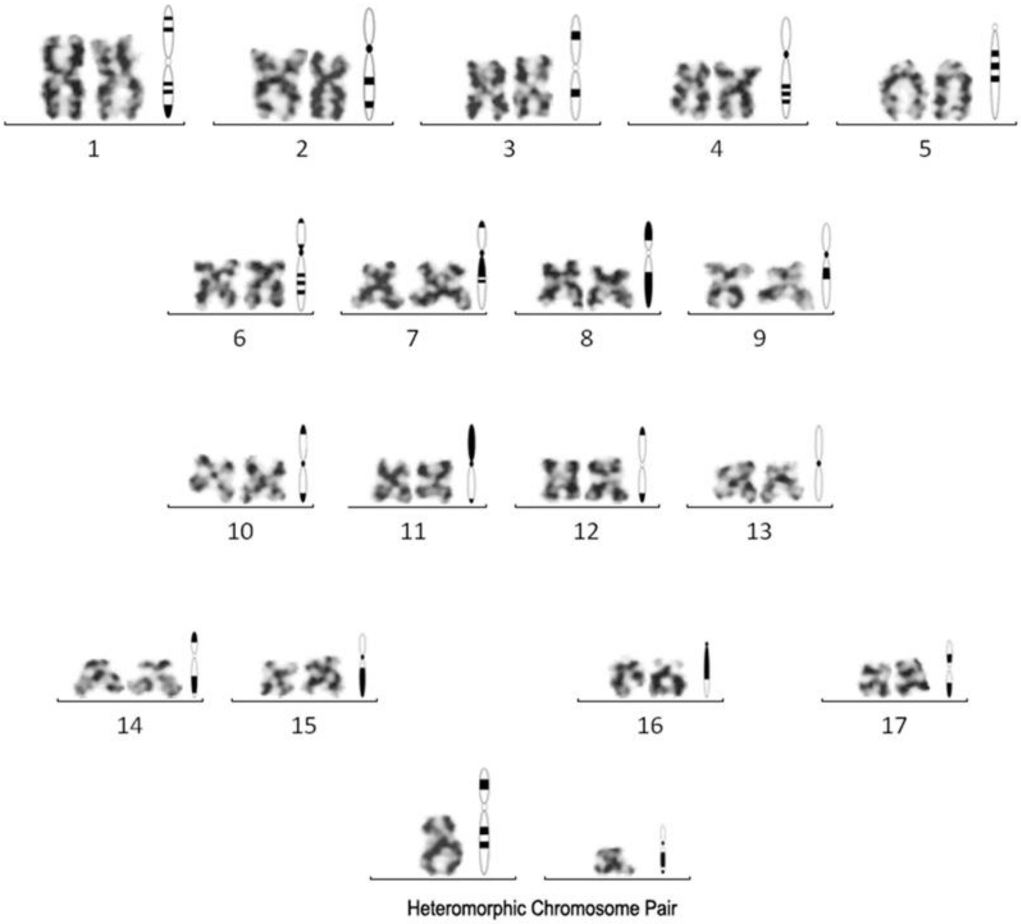

Supplementary Figure 2: Karyotype of BB02 *Biomphalaria glabrata* and ideograms of chromosomes: Distinct G-bands on the chromosomes identified 17 pairs of homologous *Biomphalaria glabrata* chromosomes. Pairs of homologous chromosomes were arranged according to size, centromere position, and G-banding pattern and assigned to groups: A (chromosomes 1-5); B (6-9); C (10-13); D(14,15); E (16); F (17). Two chromosomes were consistently left out of the pairing and have been described here as a heteromorphic chromosome pair.

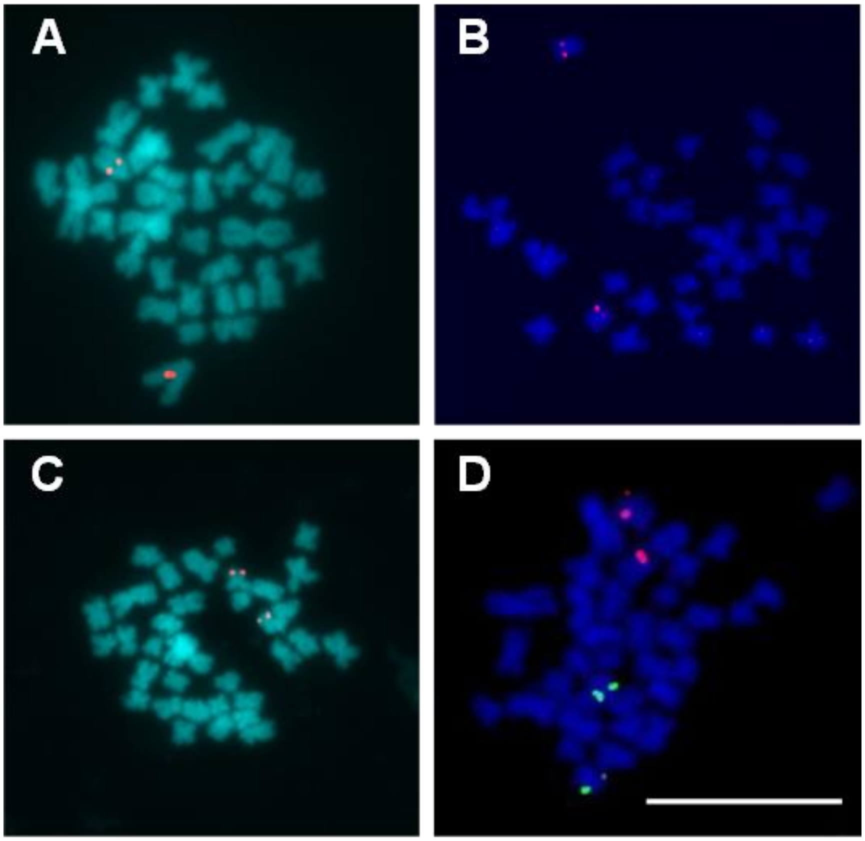

Supplementary Figure 3: Fluorescence *in situ* hybridization on *Biomphalaria glabrata* chromosomes. *Actin* [**A],** *Ferritin* **[B],** and *Hsp70* **[C]** genes were mapped onto chromosomes from the BB02 strain. *Actin* maps on to the large acrocentric chromosome 5 of group A. *Ferritin* and *Hsp70* both map onto small metacentric chromosomes of group C. Dual color FISH with *Ferritin* (red) and *Hsp70* (green) **[D]**. Scale bar = 10µm.

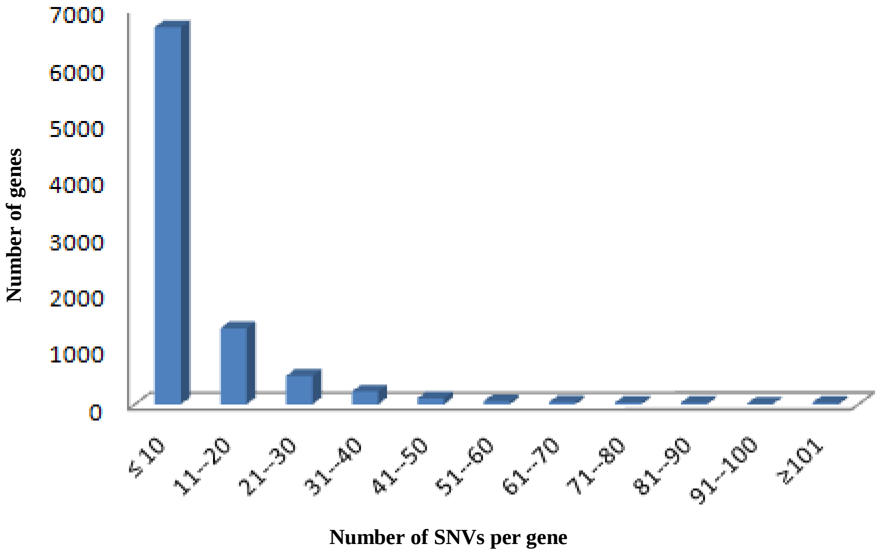

Supplementary Figure 4. SNV distribution among *B. glabrata* genes. A total of 85,790 single nucleotide variations (SNVs) were identified in 9,030 genes. As shown above, the majority of genes presented 10 or less SNVs per gene, 1,843 gene sequences contained between 11 and 30 SNVs and 516 gene sequences harbored more than 30 SNVs each.

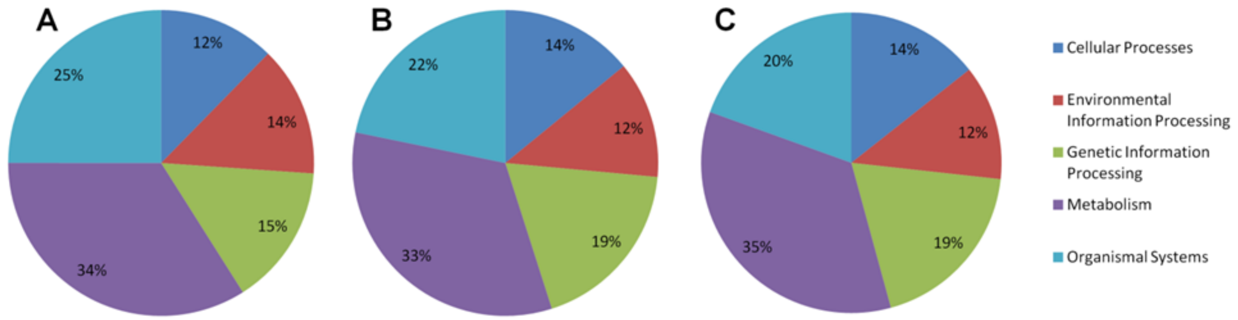

Supplementary Figure 5. **Distribution of genes and genes with SNVs across KEGG Orthology (KO) categories**. The percentage distribution for the top-level KO categories was observed to be similar for the total number of genes (A), genes with SNVs (B) and genes with nonsynonymous SNVs (C).

A

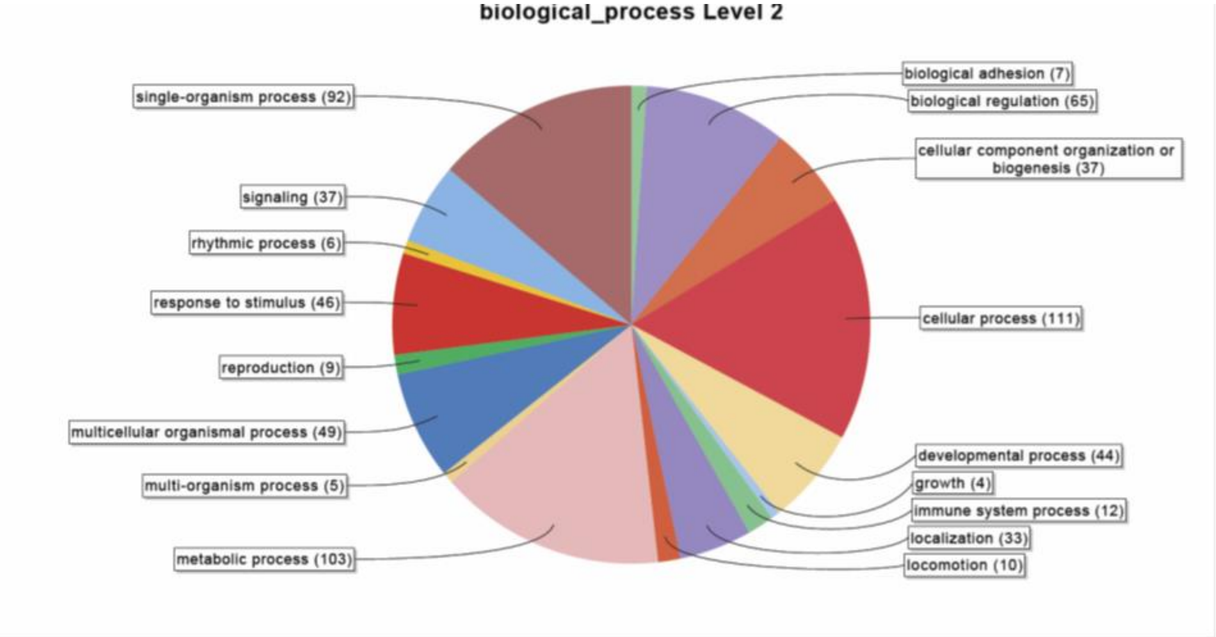

B

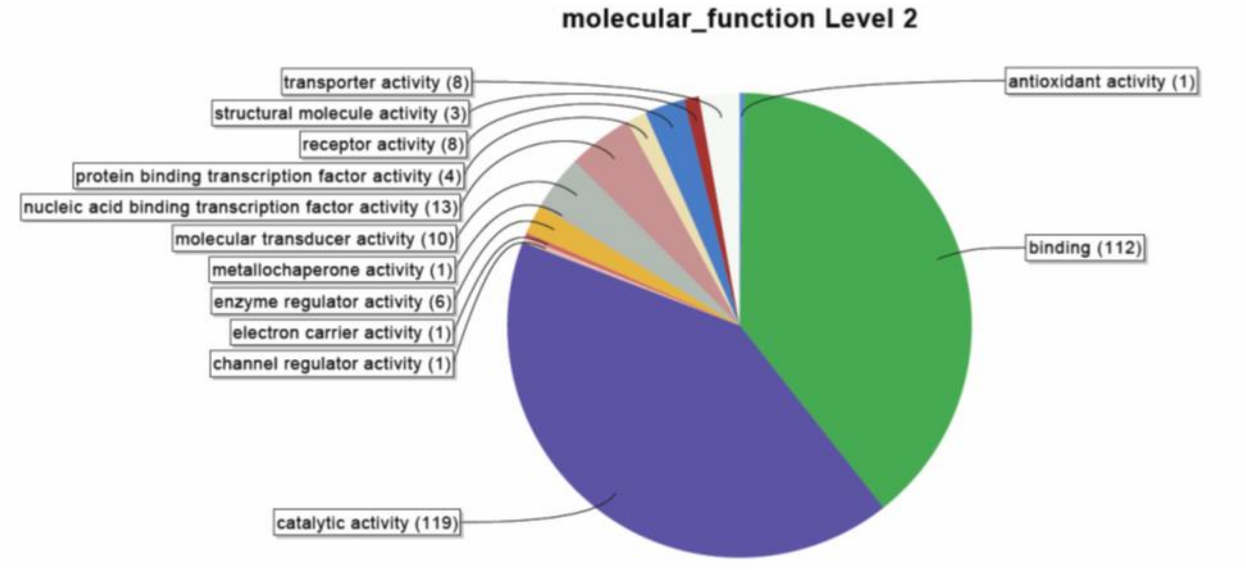

Supplementary Figure 6: GO term analysis of secreteome.

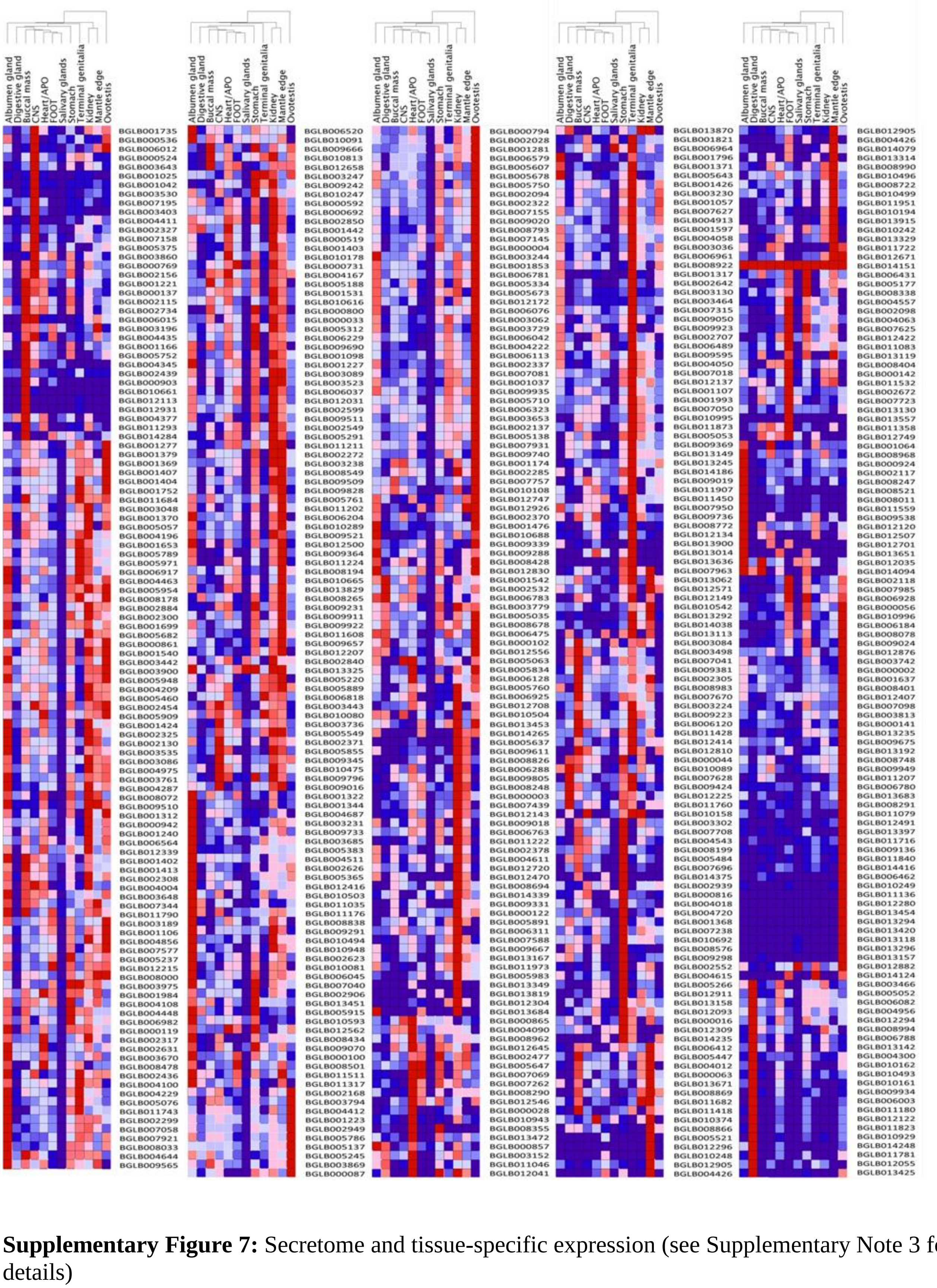

Supplementary Figure 7: Secretome and tissue-specific expression (see Supplementary Note 3 for details)

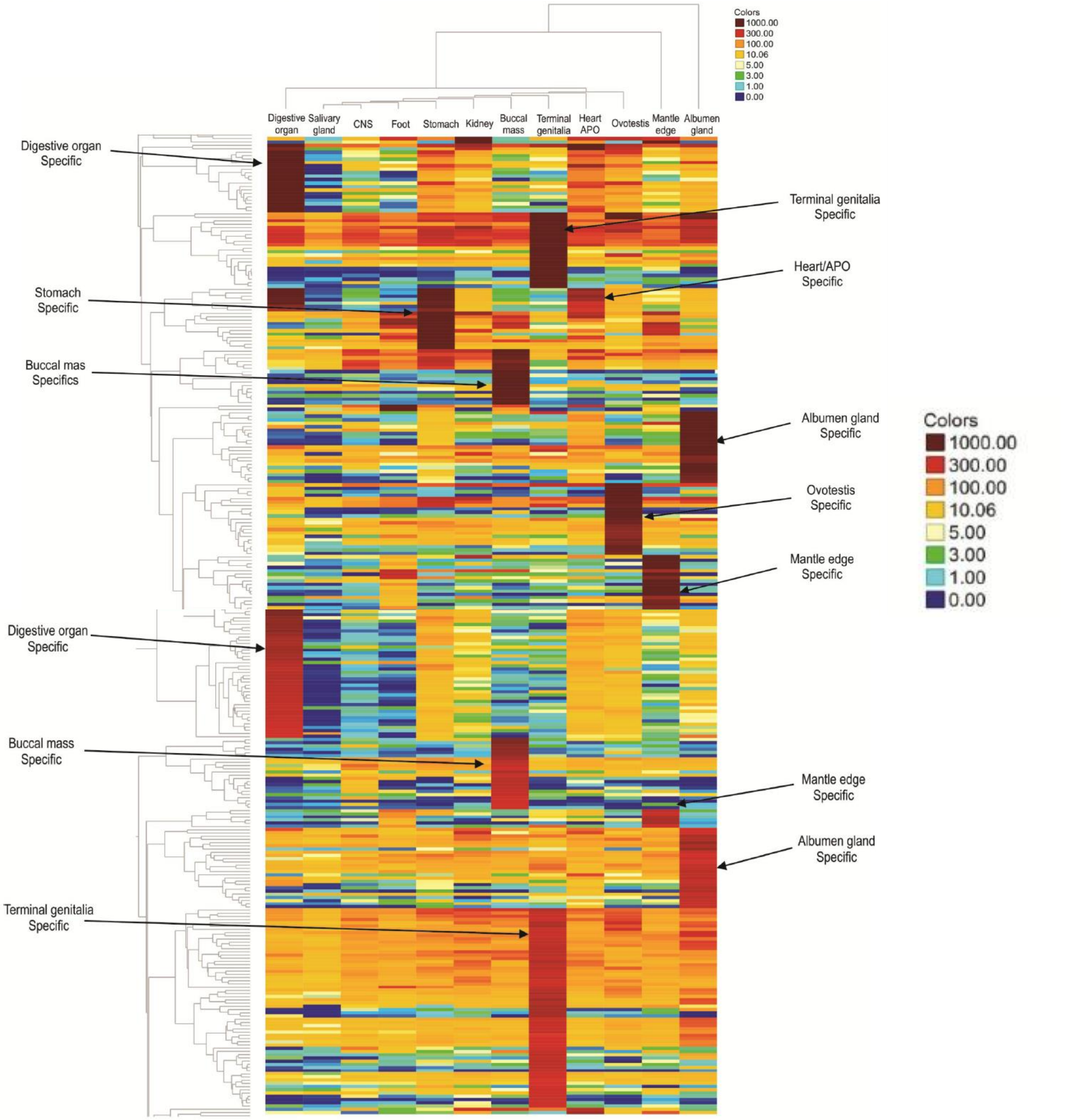

Supplementary Figure 8: **Broad diversity of tissue-specific transcripts in *B.glabrata*.** Identification of tissue specific gene expression in *B. glabrata*.Comparison of RPKM values for individual transcripts (see color scale) across tissues (indicated above the figures). The arrows point to groups of transcripts that were highly and differentially expressed in specific tissues Hierarchical clustering was performed using *Spotfire* (TIBCO Software Inc.), based on RPKM (Reads Per Kilobase per Million mapped reads). UPGMA (unweighted pair-group method of averages) clustering method was used to calculate the row and column dendrograms together with euclidiean distance measure, and ordering weight was an averaged value.. See details in Supplementary Note 4. All transcriptome data was analyzed against the *Biomphalaria* gene models. Also see Supplementary Figure 9.

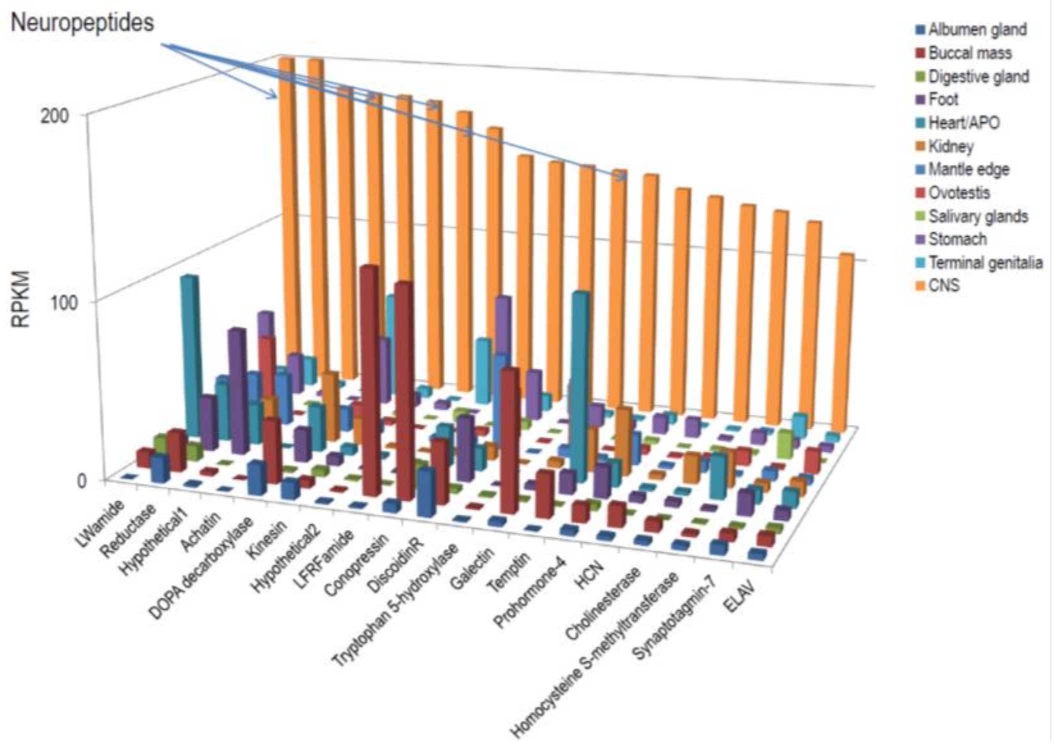

Supplementary Figure 9A: Genes highly and differentially expressed in CNS of *B. glabrata*

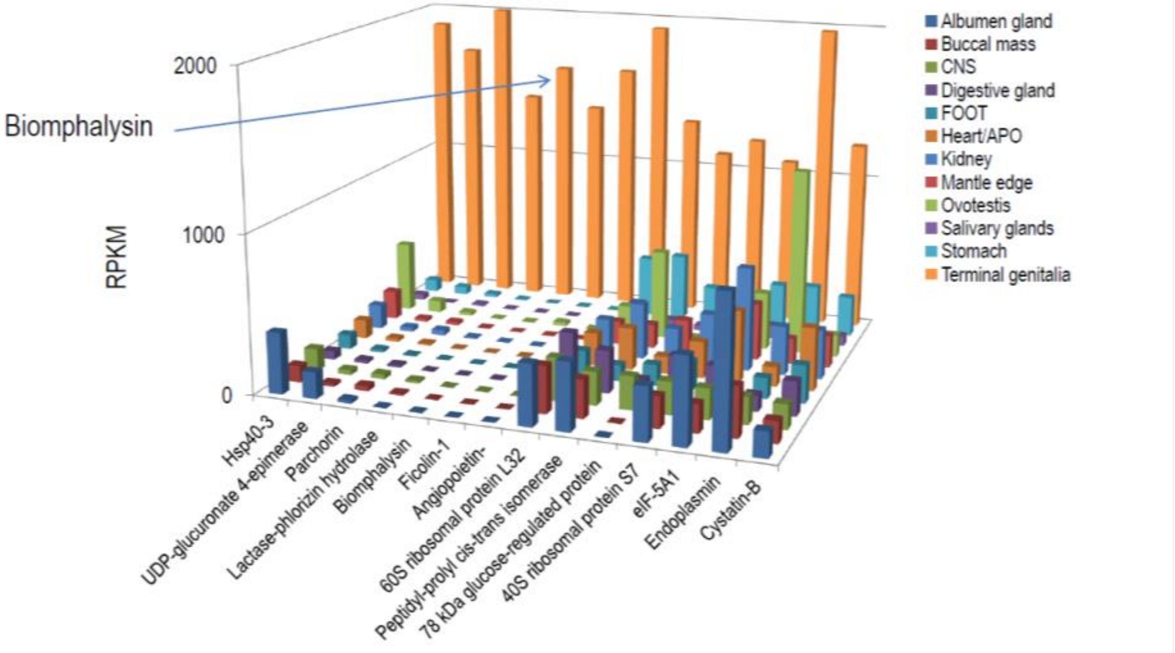

Supplementary Figure 9B: Genes highly and differentially expressed in terminal genitalia of *B. glabrata*

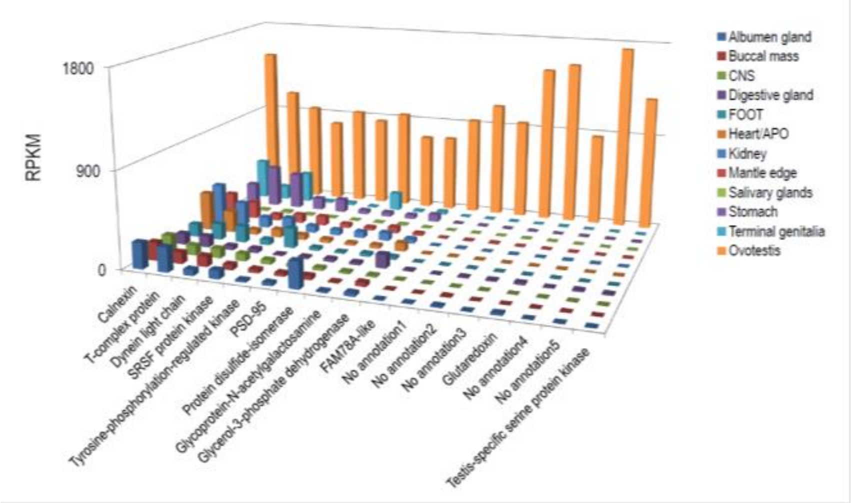

Supplementary Figure 9C: Genes highly and differentially expressed in *B .glabrata* ovotestis

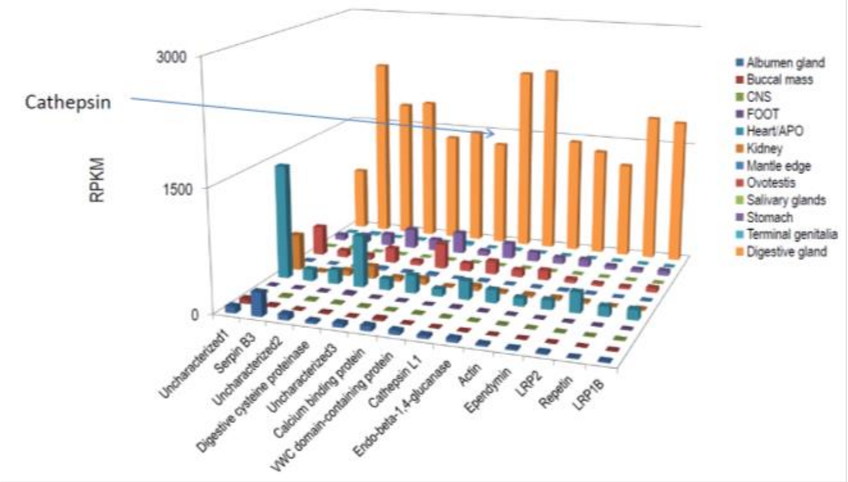

Supplementary Figure 9D: Genes highly and differentially expressed in *B.glabrata* digestive gland

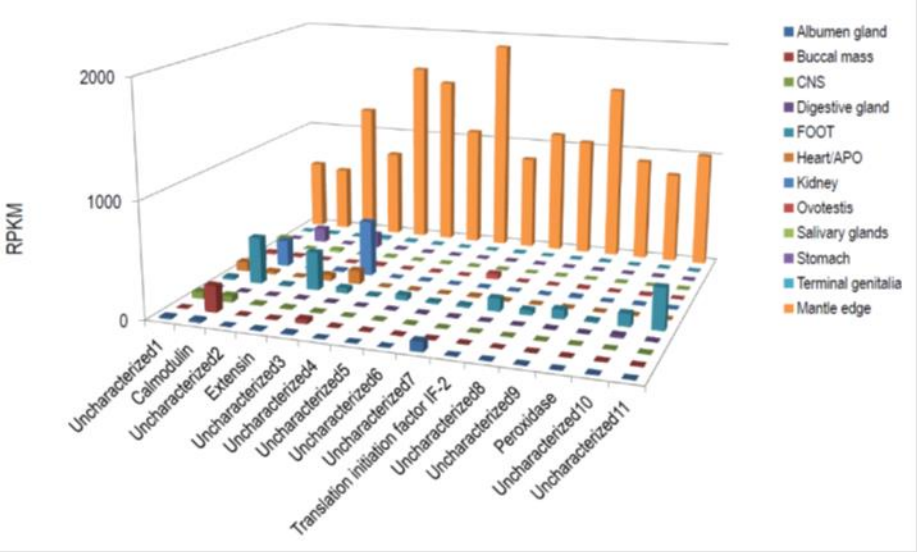

Supplementary Figure 9E: Genes highly and differentially expressed in the mantle edge of *B. glabrata*.

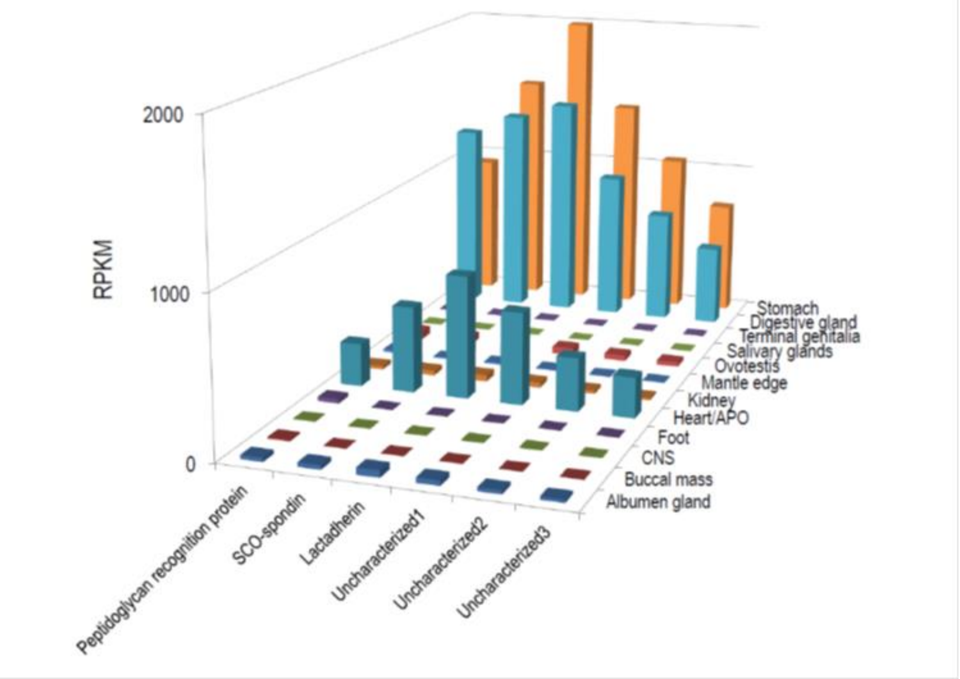

Supplementary Figure 9F: Genes differentially highly and differentially expressed in stomach and digestive gland of *B. glabrata*

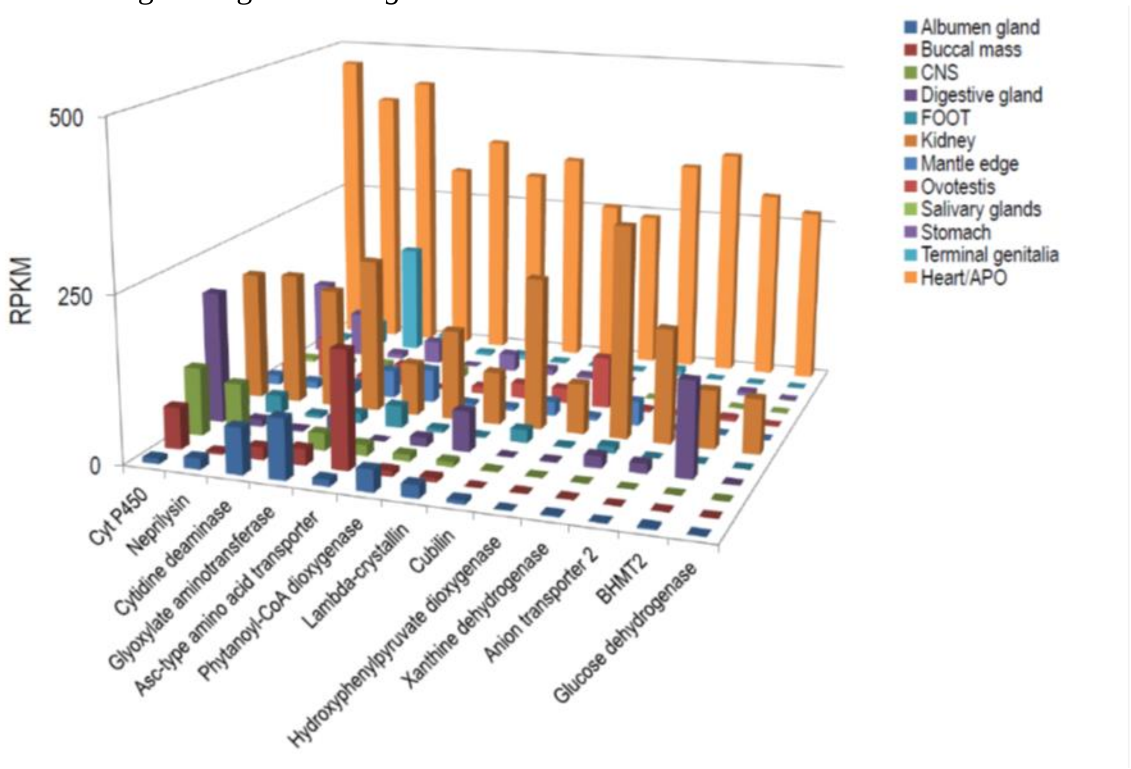

Supplementary Figure 9G: Genes differentially highly and differentially expressed in heart/APO of *B. glabrata*

Supplementary Figure 9. Tissue/organ-specific expression in *B. glabrata*. Bar graphs (RPKM values) highlight genes that are highly and differentially expressed in particular tissues. See Section 4 and Fig 8 for details and RPKM calculations

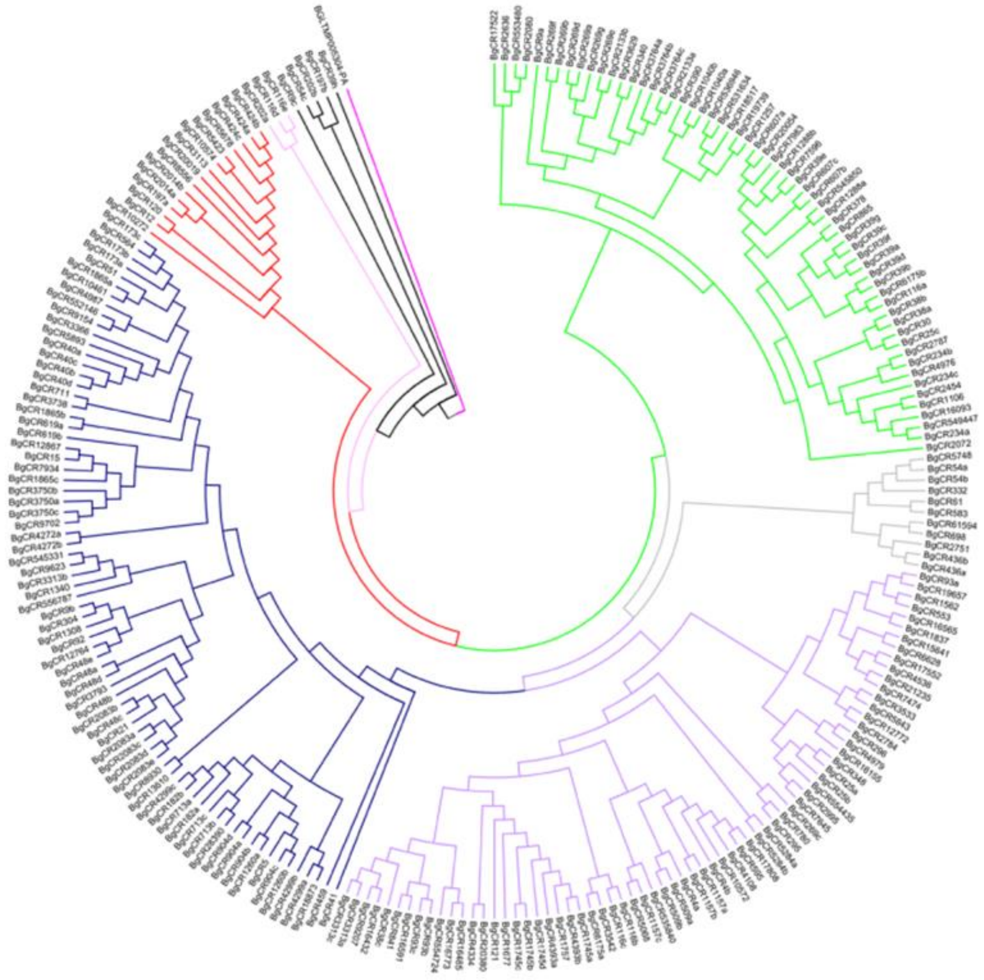

Supplementary Figure 10. Candidate chemosensory receptors identified from the *B. glabrata* genome Phylogenetic reconstruction of 242 7-transmembrane rhodopsin G-protein coupled receptor-like gene sequences identified within the *B. glabrata* genome. Each color represents a subfamily. The sequences were aligned with Clustal W and the tree was built with the Neighbor-joining algorithm.

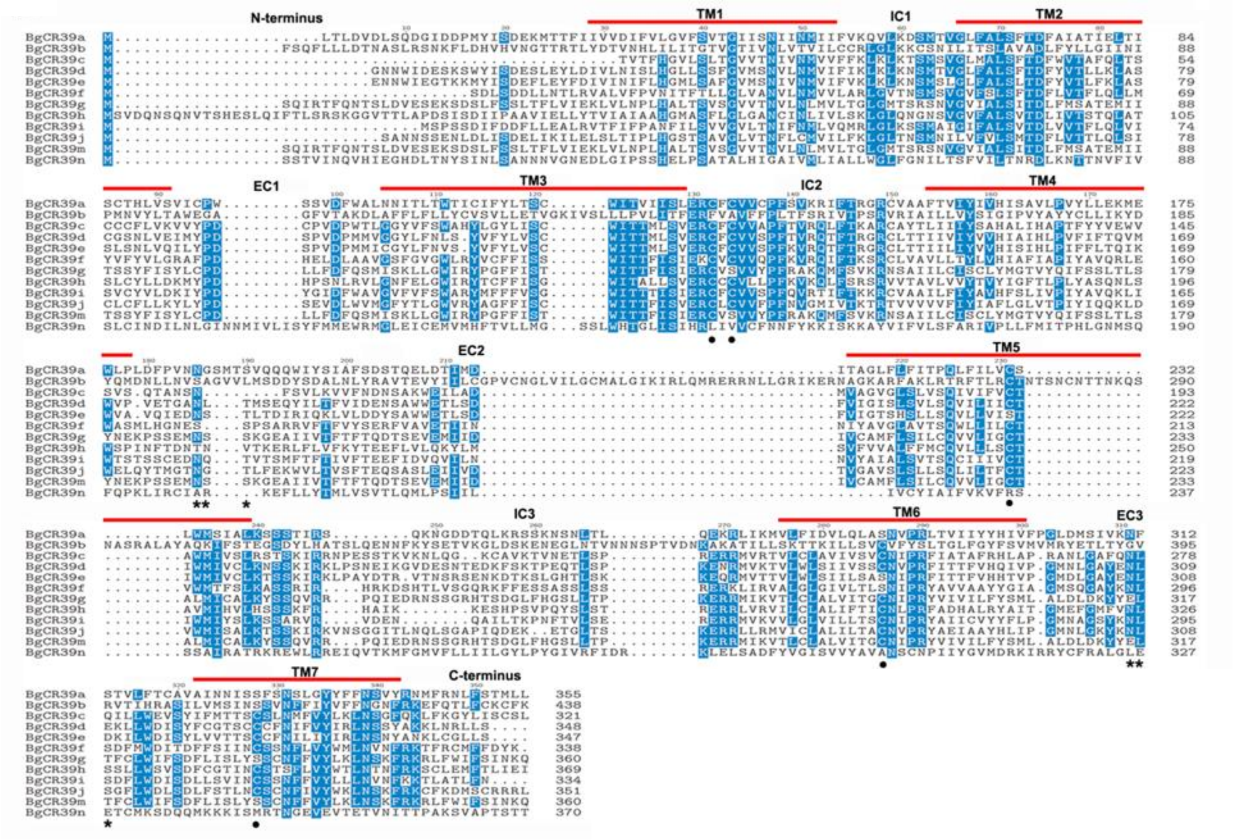

SupplementaryFigure 11. Multiple sequence alignment of candidate chemosensory receptors that cluster within *B. glabrata* genome LGUN_random_Scaffold 39. These sequences are highlighted in yellow in Supplementary Data 11

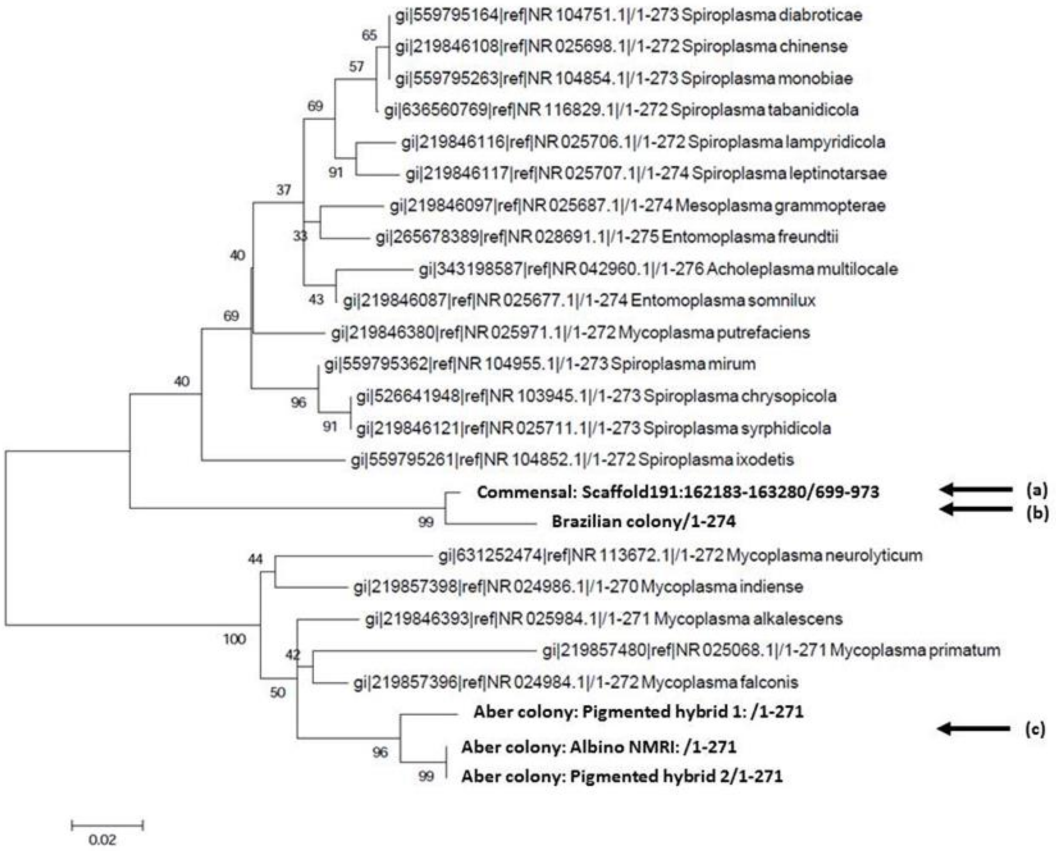

Supplementary Figure 12. Phylogenetic analysis (NJ tree) of 16S sequence from the commensal noted from the *B. glabrata* genome assembly (a), the cloned 16S sequences from genomic DNA obtained from other BB02 *B. glabrata* (b:Brazilian colony), three *B. glabrata* strains maintained at the laboratory at Aberystwyth University, Wales, UK (c: Aber colony) and other mollicutes.

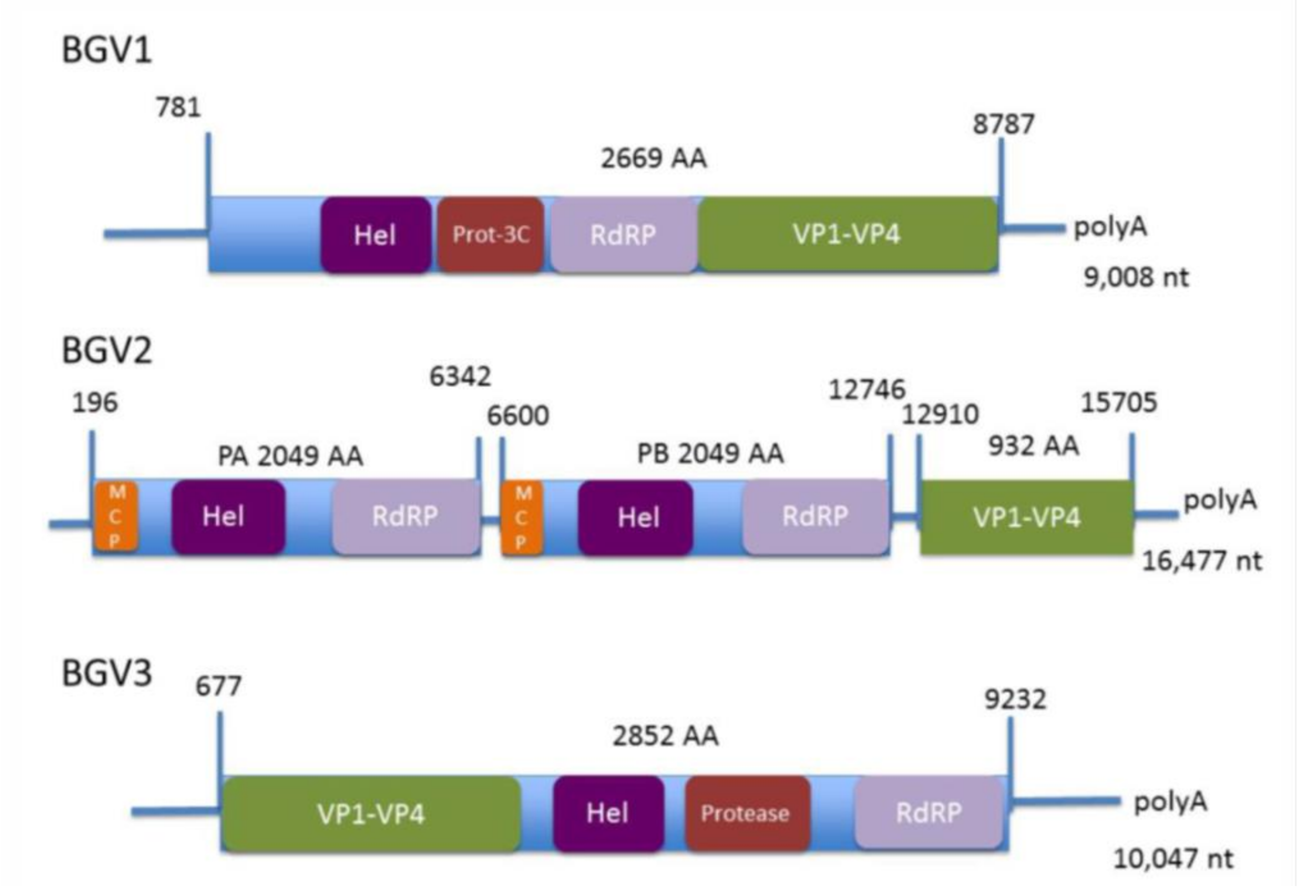

Supplementary Figure 13. Genome organization of viruses associated with *B. glabrata*

Top. BGV1. The genome has 9,008 nt with a polyA tail and the single ORF located between 781-8,787 nt of the genome. The non-structural protein located at the N'-terminus encodes the Helicase, 3C-Protease (3C-prot) and RNA-dependent RNA polymerase (RdRp) domains; the C'-terminus encodes the capsid proteins (viral proteins, VP1-VP4). **Center.** BGV2. The genome is currently 16,477 nt in length and encodes 3 ORFs. The two identical non-structural proteins (Pa and Pb) encode Helicases (Hel), polymerase and a Methyl-accepting chemotaxis protein (MCP), signaling domain. The structural ORF contains 2 rhv coat protein domains. **Bottom**. BGV3. The sequence encoding CP contains 2 rhv-like coat protein domains. The non-structural proteins have Helicase (Hel), protease and RdRp domains. Note that the protease domain for BGV3 is C4-like (similar to potyvirus proteases), instead of the C3-like domains typical for other snail small RNA viruses (Liu *et al.*, unpublished data).

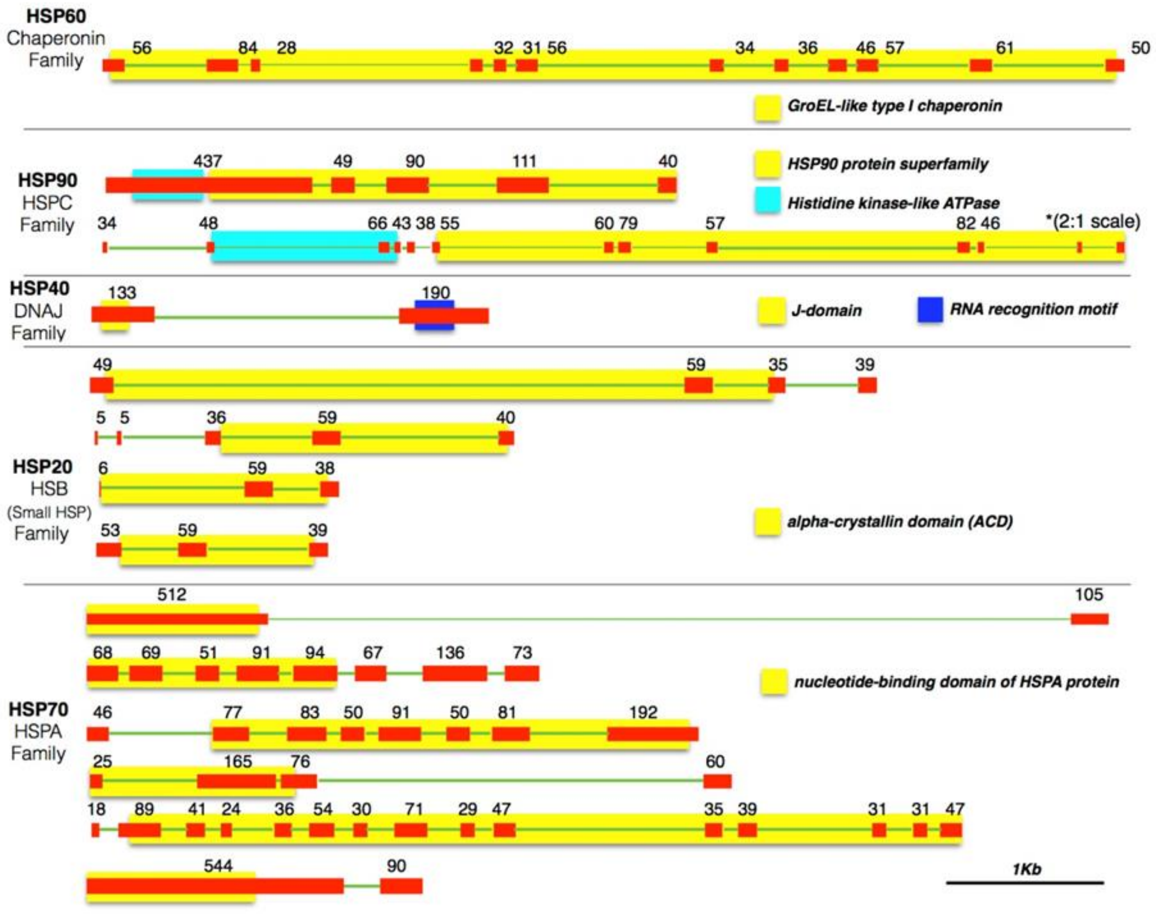

Supplementary Figure 14. Gene structure of the *B. glabrata* heat shock protein (HSP) families: The nomenclature is based on the guidelines assigned by the HUGO Gene Nomenclature Committee and used in the National Center of Biotechnology Information Entrez Gene database for the heat shock genes. The intron/exon structures are drawn to scale shown at the bottom of the image (1 Kb=1 inch) except for the second member of the HSP90 family (shown by a asterisk on the right) where it is reduced to half size in order to accommodate the entire gene structure. The exons are represented by red boxes and the introns by green lines. The numbers on top of the red boxes represent the number of amino acids present in each exon. The yellow boxes around each HSP represent the location of heat shock domains representative of that family while the cyan and blue boxes represent a specific motif within a family. The representative conserved domains for each of the HSP families are: *Chaperonin (HSP60*) - GroEL (cd03344); *HSPC (HSP90)* - HSP90 protein superfamily and Histidine kinase-like ATPases (cd00075); *DNAJ (HSP40)* - J-domain and RNA recognition motif (cd12429); *HSPB (small HSP, HSP20)* - alpha-crystallin domain (ACD, cd06526); *HSPA (HSP70)* - nucleotide-binding domain of HSPA1-A, -B, -L, HSPA-2, -6, -7, -8, and similar proteins (cd10233) and nucleotide-binding domain of the sugar kinase/HSP70/actin superfamily (cd17037).

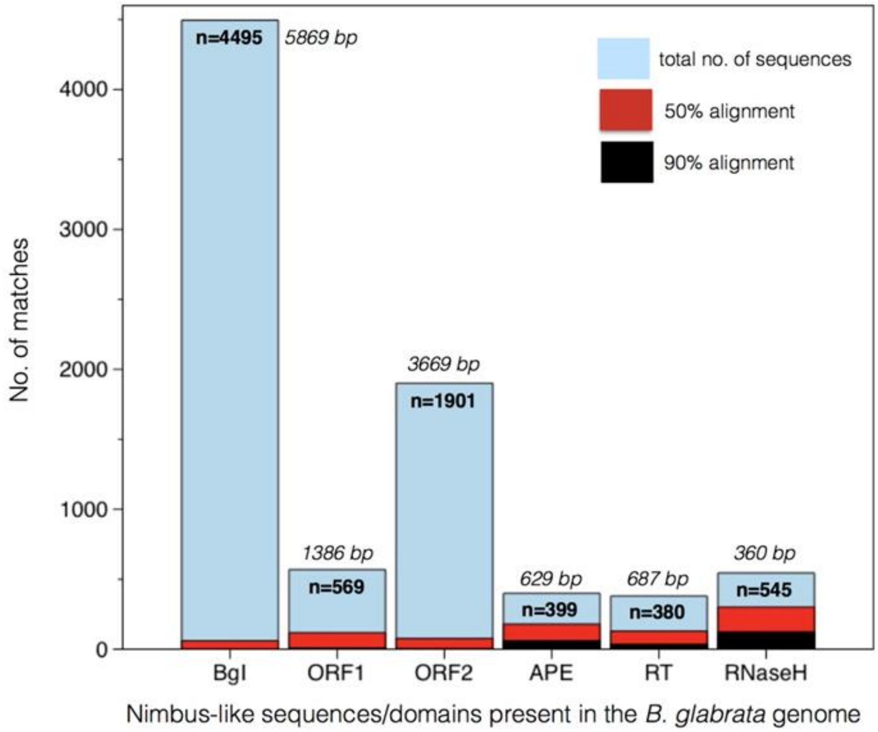

Supplementary Figure 15. The number of *Nimbus (BgI)* elements and functional domains in the genome: Blat analysis of the *B. glabrata* genome was performed using the entire *nimbus (BgI)* sequence (GenBank EF413180) and its individual components, ORF1 (gag-like protein), ORF2 (pol-like protein) and the pol sub-domains expressing Endonuclease (APE), Reverse Transcriptase (RT) and Ribonuclease H (RNase H). The above sequence queries are represented in the X-axis. The Y-axis represents the number of matches to these queries in the snail genome. The blue bars show the total number of matches of query sequences to the snail genome. The length of the query sequence (in bp) used in the search is shown in italics and the total number of matches is represented by the "n" value within the blue bar. Regions is red and black represent the number of matches/sequences where either 50% or 90% of the bases in the query sequence are aligned to the target sequence in the snail genome. The number of sequence matches that align with either 50% or (90%) of the query sequence are as follows: complete *BgI element* – 60 (2), *ORF1* – 118 (8), *ORF2* – 76 (4), *APE* – 1789 (60), *RT* – 129 (16), *RNaseH* – 301 (124).

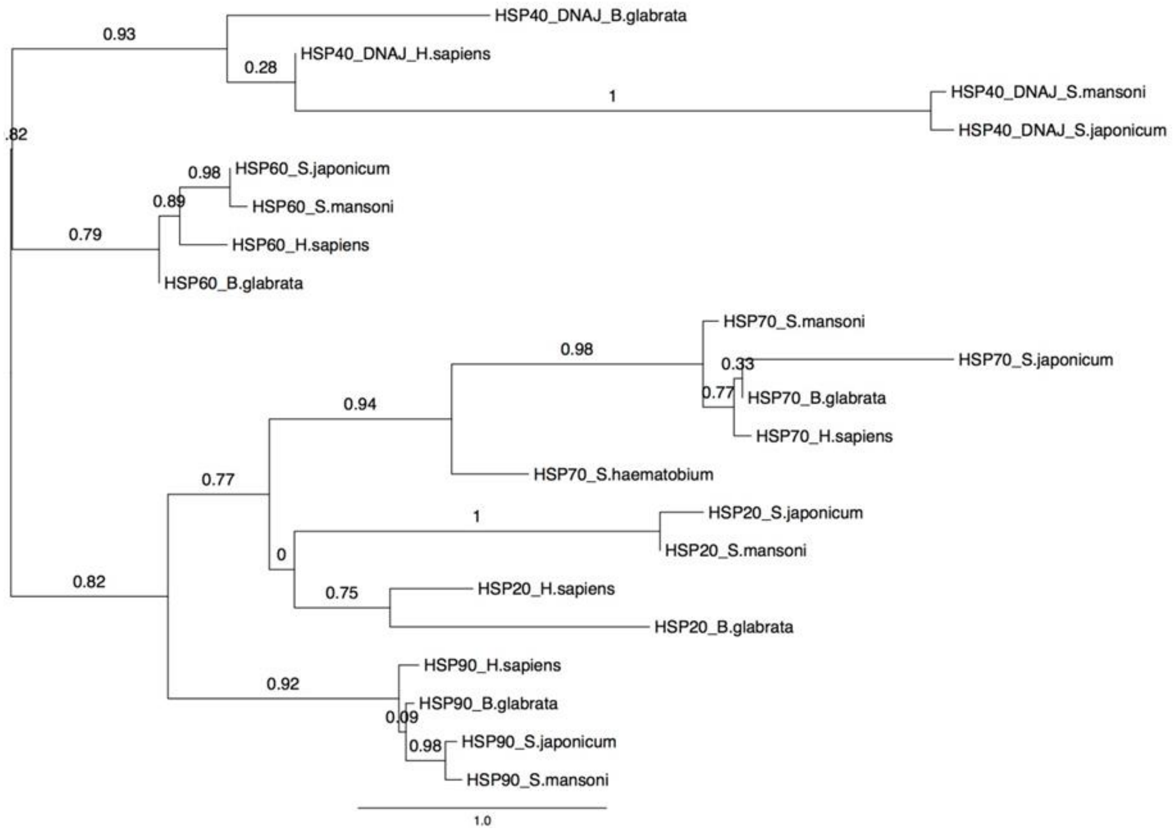

Supplementary Figure 16. Phylogenetic tree of the *B. glabrata* heat shock protein (HSP) families. Heat Shock Protein sequences from *B. glabrata*, *Homo sapiens*, *Schistosoma mansoni*, *S. japonicum* and *S. hematobium* were aligned using Phlogeny.fr. MUSCLE was used for the initial alignment, PhyML was used for phylogeny and the tree rendering by TreeDyn. The resulting newick format and visualized using the tree figure drawing tool Figtree v1.4.0 (http://tree.bio.ed.ac.uk/software/figtree/). The numbers next to each branch between 0 and 1 show support for the node, where 1 represents maximal support done by bootstrapping. A high value indicates that sequences to the right of the node cluster together to the exclusion of any other. The horizontal lines are branches that represent evolutionary lineages changing over time (the longer the branch the larger the amount of change). The bar at the bottom of the dendogram is a scale that shows the length of branch that represents an amount genetic change of 1.

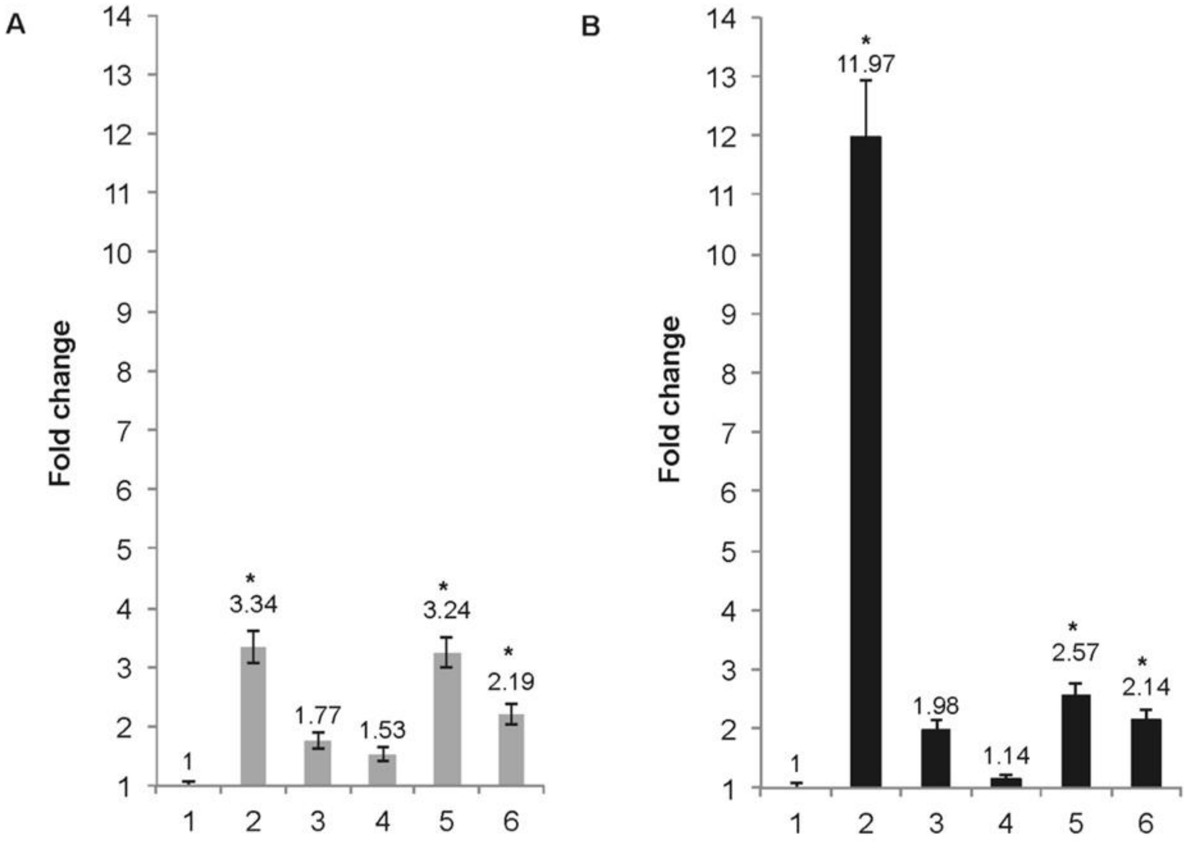

Supplementary Figure 17. Pathogen-specific expression regulation of HSP70 gene and *Nimbus* in *B. glabrata*. Real time qPCR analysis of RNA from (1) normal *B. glabrata* (2) *B. glabrata* exposed for 2 h to *S. mansoni* miracidia (3) *B. glabrata* exposed for 2 h to *S. japonicum* miracidia (4) *B. glabrata* exposed to *S. hematobium* miracidia and (5) *O. hupensis hupensis* exposed for 2 h to *S. japonicum* miracidia, and (6) *B. truncatus* exposed to *S. hematobium* miracidia. Snails were monitored under a dissecting microscope during the exposure to either compatible or incompatible parasite species for successful miracidial penetration. Note that up-regulation of Hsp 70 (3.34 fold change) and RT (11.97 fold change) occurs in *B. glabrata* snails exposed to *S. mansoni* but not to either *S. japonicum* or *S. hematobium*. Real time qPCR was done in triplicates from 12 biological replicates using the *B. glabrata* myoglobin transcript as reference gene. The fold change of Hsp 70 and RT gene transcript levels were calculated by the delta-delta Ct method using myoglobin for gene normalization. The statistical significance (indicated by asterisk) with P-values ≤ 0.05 were calculated by comparing the gene fold change (N = 12) for each group using the Student's t-test in order to determine if the differential expression of HSP70 or *Nimbus (BgI)* RT enriched transcripts between the normal and exposed groups were significant.

(A) Scaffol18083 (Grep-like): nucleotides and corresponding translated sequence:

```
  1  A  I  K  S  L  S  M  S  L  Y  N  E  T  S  K  T  F  D  V  L  Y  T  I  D  K  E  T
  1 GCCATCAAATCGCTCTCTATGTCCCTTTATAATGAAACAAGTAAAACTTTTGACGTCCTATATACAATAGACAAAGAAACA

 28  L  S  L  K  Q  I  V  E  L  K  G  V  Q  I  S  F  G  N  M  F  I  L  L  T  I  P  S
 82 CTGAGTCTCAAACAGATTGTAGAACTTAAAGGTGTACAGATTAGTTTTGGAAATATGTTCATATTGCTGACTATTCCCAGT

 55  P  S  Q  Y  D  C  Q  T  Y  S  C  I  A  N  G  Y  N  K  Y  G  N  N  D  S  I  S  T
163 CCAAGTCAGTATGATTGTCAAACGTACAGCTGCATTGCTAATGGATATAATAAATATGGTAATAATGATTCAATTTCAACA

 82  K  V  K  V  E  S  R  T  N  I  T  E  Y  I  K  E  I  N  R  L  K  K  L  E  A  N  S
244 AAGGTGAAGGTAGAAAGCAGAACAAATATAACAGAATATATCAAAGAAATCAATCGTTTGAAGAAATTGGAAGCTAACTCA

109  I  M  E  D  N  V  P  L  K  N  I  E  K  L  I  L  A  N  M  N  I  Q  M  C  S  L  T
325 ATAATGGAAGACAATGTACCTTTGAAAAACATTGAAAAGCTTATTTTAGCGAATATGAACATACAAATGTGTTCATTAACA

135  T  N  K  T  S  E  D  L  I  E
407 ACCAACAAGACATCGGAAGATCTAATTGAA
```

FREP 3.3 (AAZ80799) and translated Scaffold18083:1677-2111 alignment

```
18083     IKSLSMSLYNETSKTFDVLYTIDKETLSLKQIVELKGVQISFGNMFILLTIPSPSQYDCQTYSCIANGYNKYGNNDS
          I+SL++S YNET + FD L  +D  T +LKQ V  K  QISFGN++I LT+P+P+Q+D + Y C A+G N  G N S
FREP3.3   IRSLTLSRYNETIREFDELIALDSLTQNLKQFVRFKYSQISFGNLYITLTLPNPTQFDARIYRCNADGANSEGTNIS

18083     ISTKVKVESRTNITEYIKEINRLKKLE
           +  K  VE  TN T  I+EI R+KK E
FREP3.3   LFAKKAVEYETNSTALIEEIRRIKKDE
```

GREP (KM975647) and translated Scaffold18083:1677-2111 alignment

```
18083 AIKSLSMSLYNETSKTFDVLYTIDKETLSLKQIVELKGVQISFGNMFILLTIPSPSQYDCQTYSCIANGYNKYGNNDSIS
      AI+SLSMS YNETSKTFDVLYTIDKETLSLKQIVELKG QISFGN+FILLTIPSPSQYDCQTYSCIANGYNK+GNNDSIS
GREP  AIQSLSMSRYNETSKTFDVLYTIDKETLSLKQIVELKGAQISFGNLFILLTIPSPSQYDCQTYSCIANGYNKFGNNDSIS

18083 TKVKVESRTNITEYIKEINRLKKLEANSIMEDNVPLKNIEKLILANMNIQMCSLTTNKTSEDLIE
      TKVKVESRTNITEYIKEINRLKKLEANSIMEDNVPLKNIEKLILANMNIQMCSLTTNKTSEDLIE
GREP  TKVKVESRTNITEYIKEINRLKKLEANSIMEDNVPLKNIEKLILANMNIQMCSLTTNKTSEDLIE
```

(B) Scaffold 47310:874-1190 (Grep-like): nucleotides and corresponding translated sequence:

```
  1  E  T  N  G  V  L  A  T  I  S  R  D  Q  Q  V  G  I  S  Q  D  T  S  L  S  Q  V  Q
  1 GAGACCAACGGAGTTCTGGCCACCATTTCAAGAGATCAACAGGTTGGAATCAGTCAGGACACAAGTTTGAGTCAAGTTCAA

 28  G  Q  L  S  D  Q  D  F  N  I  S  Y  L  Q  V  I  W  I  K  P  S  P  S  Q  S  G  K
 82 GGCCAACTCTCTGACCAAGATTTCAATATCTCTTATCTTCAGGTGATATGGATAAAGCCAAGTCCTTCACAGTCTGGCAAA

 55  Y  V  C  G  A  N  I  V  N  Q  E  G  Q  L  E  K  L  K  A  S  L  E  I  T  I  E  K
163 TACGTGTGTGGAGCCAACATCGTCAACCAGGAGGGACAACTGGAAAAGTTGAAGGCTTCACTAGAAATAACAATTGAGAAA

 82  T  S  L  D  D  L  V  P  I  V  I  D  L  L  A  E  R  T  K  V  R  H  I  L
244 ACAAGTTTAGATGATTTGGTTCCTATTGTGATCGATTTATTAGCAGAAAGAACTAAGGTCAGACATATACT
```

FREP 3.3 (AEO50747.1) and translated Scaffold47310: 874-1190 alignment

```
47310     ETNGVLATISRDQQV-GISQDTSLSQVQGQLSDQDFNISYLQVIWIKPSPSQSGKYVCGANIVNQEGQLEKLKASLE
          E NG++ATIS+DQ V   +QD +L  V G+L D     SYLQV W  P  S+SGKY CGA +  N +G+ E     L
FREP3.3   EANGIIATISKDQPVVTTNQDVNLLAVIGKLHDDSSKNSYLQVTWSNPKFSESGKYFCGAHANNAQGRNEHWNEMLT

47310     ITIEKTSLDDLVPIVIDL
          IT+E+   DD+V ++ D+
FREP3.3   ITVERLQFDDIVKVMYDI
```

GREP (KM975647) and translated Scaffold47310: 874-1190 alignment

```
47310  ETNGVLATISRDQQVGISQDTSLSQVQGQLSDQDFNISYLQVIWIKPSPSQSGKYVCGANIVNQEGQLEKLKASLEITI
       ETNGVLATISRDQQVGISQDTSLSQVQGQLSDQDFNISYLQVIWIKPSPSQSGKYVCGANIVNQEGQLEKLKASLEITI
GREP   ETNGVLATISRDQQVGISQDTSLSQVQGQLSDQDFNISYLQVIWIKPSPSQSGKYVCGANIVNQEGQLEKLKASLEITI

47310  EKTSLDDLVPIVIDLLAERTKVRHIL
       EKTSL+DLVPIVIDLLAERTKVRHIL
GREP   EKTSLEDLVPIVIDLLAERTKVRHIL
```

Supplementary figure 18. Proteomics recovery of GREP-related peptides. Partial gene and protein sequence information for the BS-90 plasma Grep-related protein within LGUN_random_Scaffold18083 (A) and LGUN_random_Scaffold47310 (B). The shaded amino acid sequences represent the original peptides identified in initial proteomic analyses. Also shown is the alignment of the scaffold-encoded Grep-like protein with the *B. glabrata* FREP3.3 and Grep which exhibited highest protein sequence homologies.

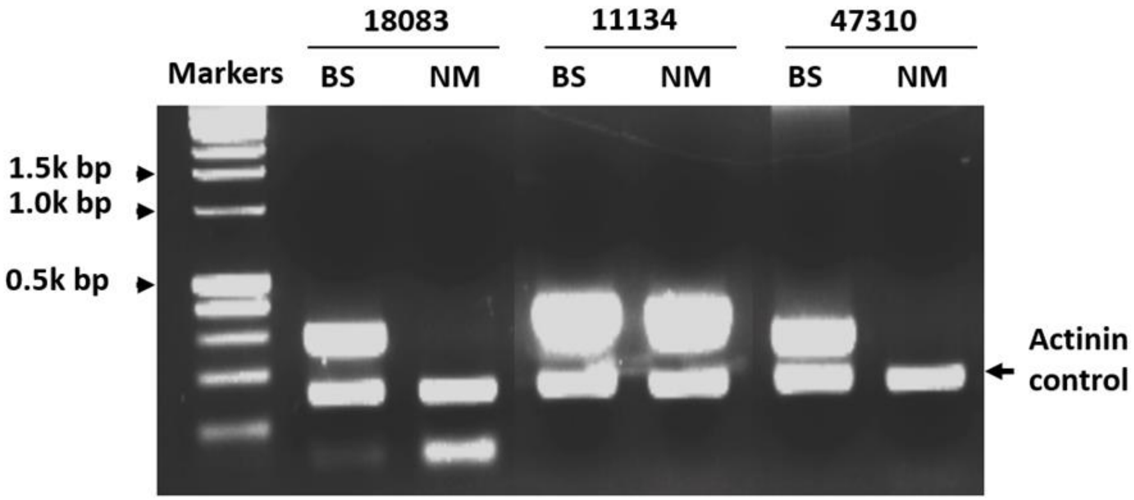

Supplementary figure 19. Expression of GREP-like sequences. RT-PCR reactivities utilizing primers specific for coding regions identified in grep-like sequences (LGUN_random_Scaffold18083 (A) and LGUN_random_Scaffold47310) in cDNA synthetized from headfeet of the resistant BS-90 (BS) and susceptible NMRI (NM) strains of *B .glabrata*. primers designed from a conserved region of Frep12.1 (LGUN_random_Scaffold11134) served as a positive control. In addition primers for *B. glabrata* actinin served as loading control. Note that Grep amplicons of predicted size were produced using cDNA of BS-90 origin, but not for the NMRI sample, indicating expression of Grep-like gene(s) only in resistant BS-90 tissues. Positive and loading controls indicate that primers were reactive in tissue preparations of both snail strains and sample loading was equivalent between tissues.

HSP70

>BGLB007783-PA
MSKAPAVGIDLGTTYSCVGVFQHGKVEIIANDQGNRTTPSYVAFTDNERLIGDAAKNQVAMNPENTVFDAKRLIGRRFDDPTVASDMKHWPFTVINEGGKPKIRVEYKGEKKTFFPEEISSMVLTKM
KETAEAYLGKTVTDAVVTVPAYFNDSQRQATKDAGTISGLNVLRIINEPTAAAIAYGLDKKVGGERNVLIFDLGGGTFDVSILTIEDGIFEVKSTAGDTHLGGEDFDNRMVNHFIQEFKRKHKKDISENKR
AVRRLRTACERAKRTLSSSTQANIEIDSLFEGIDFYTSITRARFEELNADLFRGTLEPVEKSLRDAKLDKAQVHEIVLVGGSTRIPKIQKLLQDFFNGKELNKSINPDEAVAYGAAVQAAILHGDKSEEVQDL
LLLDVAPLSLGIETAGGVMTALIKRNTTIPTKQTQTFTTYSDNQPGVLIQVYEGERAMTKDNNLLGKFELTGIPPAPRGVPQIEVTFDIDANGILNVSAADKSTGKENKITITNDKGRLSKEEIERMVNDA
EKYKNEDEKQKTRISAKNALESYAFHMKSTVEDEKLKDKISADDKKIIIDKCNEIIHWLDANQLADEEEFQHKQKEIEGVCNPIITKLYQGMGGAGGMPDFSGAAGAGAQHSAGTGGGSGPTIEEVD

>BGLB013335-PA
VIIFNGAFRRCWFQKRFKSDKVKGHVIGIDLGTTNSCVAVMEGKQAKVLENSEGSRTTPSVVAFTKDGERLVGMPAKRQAVTNSQNTLHATKRLIGRRFDDPEVQKDIKTVPYKIVKASNGDAWVEA
QGKLYSPSQIGAFVLTKMKETAENYLGTKVKNAVVTVPAYFNDSQRQATKDAGQIAGLNVLRVINEPTAAALAYGMDKTEDKIIAVYDLGGGTFDISILEIQKGVFEVKSTNGDTFLGGEDFDNALVNF
LTTEFKKEQGIDITKDSMAMQRLREASEKAKCELSSSMQTEINLPYLTMDSSGPKHMNLKLTRSKFESIVENLVKRTIGPCQKALQDADVKKTDIGEVILVGGMTRMPKVQTTVQELFGRTPSKSVNPD
EAVAIGAAIQGGVLAGDVTDVLLLDVTPLSLGIETLGGVFTKLINRNTTIPTKKSQVFSTAADGQTQVEIKVHQGEREMAADNKLLGQFTLVGIPPAPRGVPQIEVTFDIDANGIVNVSARDKGTGKEQ
QIVIQSSGGLSKDEIENMVRNAELHAEEDKKKKEMIEVLNQADGIIHDTESKMEEYKDQLPKEECDKLKQKIATVKEIIANKESKTPEEIKKETSDLQQASLKLFEMAYKK

>BGLB008248-PA
MERFTPFLMLLLVSSTCVFAKDDDDGEKKKSDVGTVIGIDLGTTYSCVGVFKNGRVDIIANDQGNRITPSYVAFTPDGERLIGDAAKNQLTSNPENTVFDVKRFIGRSWDDKSVQHDIKFYPFKVVNKN
NKPYVVVDTSDGEKTFAPEEISAMVLGKMRDIAEEYLGKKITNAVVTVPAYFNDAQRQATKDAGVIAGLNVMRIINEPTAAAIAYGLDKKDGEKNILVFDLGGGTFDVSLLTIDNGVFEVVSTNGDTHL
AQSNINWHFVLGGEDFDQRVMDHFIKLYKKKKGKDIRKDNRAVQKLRREVEKAKRALSSAHQVRLEIESFFEGEDFSESLTRAKFEELNMDLFRSTMKPVKQVLEDADLKTDDIDEIVLVGGSTRIPKV
QQLVKEFFNGKEPSRGINPDEAVAYGAAVQAGVLSGEEDTGDLVLLDVNPLTMGIETVGGVMTKLIPRNTVIPTKKSQIFSTAADNQPTVTIQVYEGERSMTKDNHLLGKFDLTGIPPAPRGVPQIEV
TFEIDVNGILKVTAEDKGTGSKNQIVIQNDQNRLSPEDIERMINDAEKYADEDKKLKEKVDAKNEIESYAYSLKNQINDKEKLGAKLSESDKEKITEAVEDAIKWLESHPDADVDEYKEKKTELEGIVQPIM
TKLYEQSGGSPPPSGEEPEEKDEL

>BGLB002386-PA
MSRKNKTPAVGIDLGTTYSCVGVFQHGKVEIIANDQGNRTTPSYVAFTDAERLVGDAAKNQAAMNPSNTIFDAKRLIGRKFSDKTVQADIKHWPFTVVEVDGRPKIKAEYKGEQKLFAPEEISSMVLT
KMKETAEAYLGGKVTDAVITVPAYFNDAQRQATKDAGAIAGLNVLRIINEPTAAALAYGLDKGERGEKNVLIFDLGGGTFDVSVLTIDEGSMFEVKATAGDTHLGGEDFDNRLVAHFMEEFKRKFNKD
MSKNPRAVRRLRTACERAKRTLSSSTEASVEIDSLFEGIDFYTKVTRARFEELCIDLFRSTIQPVENALKDAKLDKSKIHEVVLVGGSTRIPKVQKLLGDFFNGKELNKSINPDEAVAFGAAVQAAILSGDQS
NTIKDVLLVDVAPLSLGIETAGGVMTSLVKRGTTIPTKMSQIFSTYSDNQPGVDIQVFEGERAMTKDNNLLGRFELMGIPPAPRGIPQIEVTFDIDANGILNVSALDKTTGRSNNIQIKNERGRLSQREID
RMVADAEKYKVDDDRQRERIASRNQYESYVYGVKQAVETAGNRLSSSERDTALDACNAGIRWLEGNSLAEKEEFEDRLKNLQKTCAPIMVKLHQGASSDNSSGPRVEEVD

>BGLB002387-PA
MQRRNKVPAIGIDLGTTYSCVGVFQHGKVEIIANDQGNRTTPSYVAFTDAERLVGDAAKNQAAMNPSNTIFDAKRLIGRQFSDATVQADIKHWPFTVVEEGGRPKIKVDYNGGHNLFAPEEISSMVL
THMKETAEIYLEGKVTDAVITVPAIFNDAQRQATKDAGAIAGLNVLQIINEPTAAALAYGLDKGERGEKNVLIFDLGGGTFDVSVLTIDEGSMFEVKATAGDIHLGGEDFDNRLVAHFMEEFKRKFNKD
MSKNPRAVRRLRTACERAKRTLSSSTEASMEIDSFFEGIDFNSKVSRATFEELCINMFESAIEIVECALKDAKMEKNDIHEVVLVGGSTRIPKVQKLLGDFFDGKSLTKSINPDEAVAYGAAVQAAILNGD
QSNAIKDVHLVDVTPLSLGIESYDGYMTTIVKRGTPIPTRQFQGYTTPFDNTTKLSIKVFEGEGRMTKDNHLLGKFLLTGIYPAPKGQPKIEVIFDIDASGILQVTALDKTSGRSNHIQIANKGGRTSQCEIN
RMVSHVDDEQIEKERLAAKSQLESYIQEAKQTIESSSELFTLSDKDTVLNACNLSIEWLNDNPLSTKEECEDGLNTLLRTCAPIMMKLCQMMSSNQACKRKISDIDEN

>BGLB003615-PA
MPGRNKAPAIGIDLGTTYSCVGVFQHGKVEIIANDQGNRTTPSYVAFTDTERLVGDAAKNQAAMNPSNTIFDAKRLIGRKFNDKTVQSDMKHWPFKVVEVDGKPKIKAEYRGEQKLFAPEEISSMVL
TKMKETAEAYLGQKVTDAVITVPAYFNDSQRQATKDAGAIAGINVLRIINEPTAAALAYGLDKGQKGEKNVLIFDLGGGTFDVSILTIDEGSMFEVKATAGDTHLGGEDFDNRMVAHFVQEFKRKHNK
DMSNNPRAIRRLRTACERAKRTLSSSSEASIEIDSLYEGIDFYTKITRARFEELCGDLFRSTLQPVETALRDAKLDKGKIDEVVLVGGSTRIPKVQKLLSDFFNGKELNKSINPDEAVAYGAAVQAAVLTGDT
SETIKDVLLVDVAPLSLGIETAGGVMTPLVKRGTTIPTKTSQIFTTYSDNQPGVDIQVYEGERAMTKDNNLLGQFHLTGIPPAPRGVPQIEITFDIDANGILNVSAQDKSTGKSNNITIKNDKGRLSQAEID
RMLSEAEKYKDEDKRQQERVSARNQLENYVFSVKQAVDAAGEKLSSNDKDTVLNVCNSTL

>BGLB007604-PA
MSSRKKAPAIGIDLGTTYSCVGVFQHGKVEIIANDQGNRTTPSYVAFTDTERLVGDAAKNQAALNPSNTIFDAKRLIGRKFNDKTVQSDMKHWPFKVVEVDGRPKIQAEFKGETKLFAPEEISSMVLTK
MKETAEAFLGQKVTDAVITVPAYFNDSQRQATKDAGAIAGINVLRIINEPTAAALAYGLDKGHKGEKNVLIFDLGGGTFDVSILTIDEGSMFEVKATAGDTHLGGEDFDNRMVAHFVQEFKRKHNKD
MSNNARAIRRLRTACERAKRTLSSSSEASIEIDSLFDGVDFYTKITRARFEELCGDLFRSTLVPVESALRDAKLDKGRIDEVVLVGGSTRIPKVQKLLTDFFNGKELNKSINPDEAVAYGAAVQAAVLTGDTS
DTIKDVLLVDVAPLSLGIETAGGVMTPLVKRGTTIPTKTSQIFTTYSDNQPGVDIQVYEGERAMTKDNNLLGKFHLTGIPPAPRGVPQIEVTFDIDANGILNVSAQDKSTGKSNNITIKNDKGRLSQADID
RMLADAERYKDEDKKLQERVTARNQLESYVFGVKQAVEAAGDKLSSSDKDTVMTACETALKWLENNSLAEKEEFEDKLKELQKISSSVMAKLHSQGAPSSSSGQSDGHHSGPTVEEVD

>BGLB012438-PA
MPKVPSVGIDLGTTYSCVGVFQHGKVEIIANDQGNRTTPSYVAFTDTERLVGDAAKNQAAMNPSNTIYDAKRLIGRKFSDKTVQNDMKHWPFQVVEVDGRPKIKAEYRGEQKLFAPEEISSMVLTK
MKETAEAYLGQKVKDVVITVPAYFNDSQRQATKDAGAIAGLNVLRIINEPTAAALAYGLDKGQRGEKHVLIFDLGGGTFDVSILTIDEGSMFEVKATAGDTHLGGEDFDNRMVAHFVQEFKRKFNKD
MSSNPRSIRRLQTACERAKRTLSSSTEASIEIDSLFDGIDFYTKITRARFEELCGDLFRSTLQPVETALRDAKLDKSKIDEVVLVGGSTRIPKVQKLLTEFFNGKELNKSINPDEAVAYGAAIQAAVLTGDTSD
TIKDVLLVDVAPLSLGIETAGGVMTPLVKRGTTIPTKTSQTFTTYSDNQSGVDIQVYEGERAMTKDNNLLGKFHLTGIPPAPRGVPQIEVTFDIDANGILNVTAQDKSTGKSSNITIKNERGRLSQADIDR
MLSEAEKYKEEDQKQQERVMARNHLEGYVFSVKQAVDAAGDKLTTSDKETVLSACETTLKWLESNSLAEKEEYEDKLKEIQKISSSVMSKLHSQGHSGPSPSGQHHNGPTVEEVD

>BGLB004431-PA
MKHWPFKVVEVDGRPKIQAEYRGENKLFAPEEISSMVLTKMKETAEAYLGQKVTDAVITVPAYFNDSQRQATKDAGAIAGLNVLRIINEPTAAALAYGLDKGHKGEKNVLIFDLGGGTFDVSILTIDEG
SMFEVKATAGDTHLGGEDFDNRMVTHFVQEFKRKYNKDMSSNPRAIRRLQTACERAKRTLSSSSEASIEIDSLYEGIDFYTKITRARFEELCGDLFRSTLQPVETALRDAKLDKGKIDEVVLVGGSTRIPK
VQKLLTDFFNGKELNKSINPDEAVAYGAAVQAAVLTGDTSDTIKDVLLVDVAPLSLGIETAGGVMTPLVKRGTTIPTKTSQIFTTYVDNQPGVDIQVYEGERAMTKDNNLLGKFHLTGIPPAPRGVPQI
EVTFDIDANGILNVSAQDKSTGKTNNITIKNDKGRLSQAEIDRMLSDAEKYKDEDKKQQDRVSARNQLENYVFNVKQAVDAAGEKLSSSDKDTVLNACSSTLKWLDNNSLAEKEEYEDKLKEIQKLSSS
VMAKLHSQGGSAPGHTQDHNSGPTVEEVD

>BGLB013913-PA
MDEDSSLEVFPRFHAVPFSKMLTFFRKEPFTLTASYSPNSGVPYPNLEIGKAQFEELAAPLFQRLEDTLQAVLDSAKLKPSDVAAVEIIGGSSRVPAFKSIVQKVFSKEPSTTLNADEAVARGIVLATAFDN
QLGGRDLDELIVNYMCEEFKKHYKIDPKTKAKAYIRLAQECEKLKKLMSANVQPIPLNIECFMEDKDVHSSMDRSYVSLGDKSRVIGVAAKQQAVTNFKNTICGFKRVLGRKFDDPVVQTEILKRVARA
GGIETIANEYSDRCTP

Supplementary figure 20. Proteomics annotation of HSP70 family proteins. HSP70 family proteins annotated in the *B. glabrata* genome that contain amino acid (aa) sequences that match with peptides from proteomic analyses of Bge cells. Bge cell peptides with exact matches to the snail sequence. Underlined peptides identify peptides that contain miss-matched aa (designated in red). Underlined and shaded peptides indicate the presence of both identical and mismatched peptides in Bge cells.

HSP60

>BGLB013300-PA
MLRVASVFRSSATRQLVPMLCRHYAKDIKFGSDARALMLQGVDLLADAVAVTMGPKGRNVILEQSWGSPKITKDGVTVAKGIDLKDKFQNIGAKL
VQDVANNTNEEAGDGTTSATVLARSIAKEGFERISRGANPVEIRRGVMLAVDAVVEHLKKMSRQVTTPEEIAQVATISANGDKSIGELISSAMKKV
GRDGVITVKDGKTLKDELETIEGMKFDRGYISPYFMNTAKGAKCEFQDALVLLSEKKISSIQSIIPALELANQARKPLLIVAEDVDGEALSTLVLNRIKVG
LQVCAVKAPGFGDNRKNTLIDMAIATGGVVFGDEGNLYKLEDIQMQDFGNVGEVTVTKDDTLLMKGKGNKADIEKRIAQIKDEIEISTSEYEKEKFG
ERLAKLSNGVAVLKIGGTSEVEVNEKKDRINDALNATRAAVEEGIVPGGGTALLRCISVLDSVKTENEDQITGVNIIRKALRVPALTIAQNAGVDAHV
VVEKVLNSSGDIGYDALNNEYVNLIEQGIIDPTKVVRTALVDAAGVASLLTTAEAVVVDLPKEEKEAGMGGMGMGGMGGMGGMM

T-Complex protein1 (TCP-1)

>BGLB007989-PA
MQRNKAMPKTITNAKIACLDFSLQKTKMKLGVQLLVEDPEQLELMRKRESDITKEKIQKILNAGANVILTTGGIDDLCLKYFVQAGAMAVRRCKKT
DLKRIAKATGAQLLLTLANMEGEETFEASYLGLADEVSQERICDDELIIIKGPKARTASSIILRGANDYMVDEMERSIHDALCVVRRVLESKKVVAGGG
AVEAALSIYLENFATSLASREQLAIAEFAQSLLVIPKQLAVNAAQDSTDLVAKLRAFHNTSQTRAEHNELRWVGLDLYEGVVRDNKKAGVLEPAISKI
KSLKFATEAAITILRIDDMIKLIAEKQGGPSYQDACRSGELDD

>BGLB011376-PA
MGAEEEKSETARMSSFVGAIAIGDLVKSTLGPKGMDKILQTSSNNETIQVTNDGATILKSIGVDNPAAKVLVDISKVQDDEVGDGTTSVVVLACELL
KEAENLVAQKIHPQTVIAGWRKAVDAARQALTDAARDNGKDPVKFREDLMNIARTTLSSKILTQHKDLFSKLAVDSVLRLKGSGNLDAIQLIKKLGG
NLSDSYLDEGFLLDKKVGVNQPKRVENAKILIANTPMDTDKIKVFGSRVRVDAISKVAQLELAEKEKMKSKVEKILKHGCNVFINRQLIYNYPEQLFSD
AGVMAIEHADFEGKYLTYLCYYFNVCSLGGEIVSTFDCPEKVKLGTCDLIEEVMIGEDKLLKFSGVALGEACTIVLRGATQQILDEAERSLHDALCVLS
QTVKETRTVYGGGCSEMLMADAVSKLAAKTPGKESVAMEAFAKSLRQLPTIIADNGGYDSADLIAQMRAAHTNGKHTIGLDMERGRIA
DMAELGITESYQVKRQVLMSGAEAAEMIMRVDNIIRAAPRARAPDHRHH

>BGLB011864-PA
CKSFINLNDYVISDQNTKRESGRKVQIGNIEAGKSDESIIMDMRIMNERTVADIIRTCLGPRSMLKMLMDPMGGIVMTNDGNAILRELNVQHPAA
KSMIEVARTQDEETGDGTKSVIILAGEVLSVAELFIHQQMHPTVIIAAYRQALEDILEDLKHKIAMDLACSIALEATTTVATEENNKKEIDIKRYAKVEKI
PGGLIEDSKVLKGVMFNKDITHPKMRRYFYIYLYL

>BGLB005581-PA
MPQVGAYGGGGDNVSRSEYKDKDKPTQIRYSNITAGKAVADAIRTSLGPRGMDKMIQAANGDVTITNDGATILKQMQVLHPAAKMLVELSKAQ
DIEAGDGTTSVVVLAGSFLDASANLLARGIHPTVISEAFQRAAVKSTEILESMVTPLDLSDRESLLKSASTSLNSKVVSQYSSILSPIAVDAVMKVIDPIT
AKNVDLKDIKIVKKLGGTVEDTELIEGLVFSSKTLSAGGPTKVEKAKVGLIQFCISPPKTDMDNQVIVSDYTQMDRVLREERAYILEIVKKIKKAGCNVL
LIQKSILRDAVSDLALHFLAKMKILVVKDVEREDVEFVCKTLGCRPIASVDHFLPEYLGTAELVEEVQAGSGKIVKITGIANPGQTVTVLIRGSNKLVLEE
ADRSLHDALCVIRCLVKKRALIAGGGAPEIELSLKLMEYANTLTGIEQYCFRAFAEALEVIPFTLAENAGLNPIATVTELRTRHARGERTAGINVRKGAI
TNILEESVVQPILVTVSAINLAAETVRSILKIDDIVNCVRN

>BGLB007375-PA
MNILPTQLAFDEYGRPFIILRDQDKQSRLTGKDAHKSHILAAKSVANVIKTSLGPKGLDKMMVGPDGDVTITNDGATILDLMDVDHQIAKLMAEKL
LDRGIHPIRISDGYEMAAKIATEKLEKISETFKVDLTDKEPLIRLAMTTLGSKIINRCHRQMAEIAVDAILSVADFDRKDVDFELIKVESKVGGKLEDTML
VKGVIIDKDMSHPQMPKEVRDARIAILTCPFEPPKPKTKHKLDVTSVEDYHKLREYEKEKFDSMVQQVKDTGANLVICQWGFDDEANHLLLQRELP
AVRWVGGPEIELIAIATGGRIVPRFEELTTEKLGKAGLVKELVFGTTKDRMLVIEECHNSRAVTIFIRGGNKMIVDEAKRSIHDALCVIRNIVRDNRIVY
GGGAPEISASLAIQEAADKISTLEQYAVRAFGEALESVPLALAENSGLSPFQTLAEVKSRQVKENNPFLGIDCLNRGTCDMKEQHVIETLTAKKQQILL
AAQLVRMILKIDDIRAPNDHM

Supplementary figure 21. Proteomics annotation of HSP60 family proteins. HSP60 family proteins annotated in the *B. glabrata* genome that contain amino acid (aa) sequences that match with peptides from proteomic analyses of Bge cells. All peptides from Bge cell match to the single HSP60 sequence found in VectorBase . Shaded sequences represent Bge cell peptides with exact matches to the snail sequence. However, there were fewer exact matches and greater numbers of mis-matches in peptides aligning with the T-complex proteins. Underlined peptides identify peptides that contain mis-matched aa (designated in red). Underlined and shaded peptides indicate the presence of both identical and mismatched peptides in Bge cells.

HSP90/84

>BGLB001827-PA
MPEINPAEEMEGEKETFAFQAEIAQLMSLIINTFYSNKEIFLRELISNSSDALDKIRYESLTDPSKLDSGKDLHIRIIPDKENKTLTIEDSGIGMTKADLVN
NLGTIAKSGTKAFMEALQAGADISMIGQFGVGFYSAYLVADRVVVDSKHNDDEQYTWESSAGGSFTVSPSRAAPLSRGTRITLYMKEDQLEYLEEK
RIKDVIKKHSQFIGYPIKLLVEKERDKEISDDEEEEKKDDDKEEKEEDKPKVEDLDENDDADAEKDKKKKKKIKEKYNEEEELNKTKPLWTRNADDITQE
EYAEFYKSLTNDWEDHLAVKHFSVEGQLEFRALLFIPKRAPFDMFENKKKKNNIKLYVRRVFIMDNCEDLIPEYLNFVKGVVDSEDLPLNISREMLQQ
SKILKVIRKNLVKKCVELIEDLTEDKENYKKFYEQFAKNLKLGIHEDSTNRKKLAEFLRYYTSQSGDELCSLKDYVSRMKENQKDIYYITGESREAVQNSA
FVERVKKRGYEVIYMIDPIDEYSVQQLKEFEGKNLVCVTKEGLQLPEDEAEKKRLEEAKSQFEGLCKVMKEILDKKVEKVVVSNRLVTSPCCIVTSQYG
WSANMERIMKAQALRDTSTMGYMAAKKHLEINPDHPIIKTLKEKADADKNDKAVKDLCLLLFETSLLASGFSLEDPTSHANRIHRMIKLGLGIDDDD
SSATEGSGTDAASTEDMPPLEGDEDDASRMEEVD

Glucose regulated protein 90

>BGLB002553-PA
VQALDDDDDDDDVKLEDDIGKSRDGSRTDDEVVQREEEAISLDGLSVAQMKELREKSEKFQFQAEVNRMMKLIINSLYKNKEADKDNKVLHITDT
GIGMTKDELVKNLGTIAKSGTSDFLAKLNDANTVTETNDLIGQFGVGFYSAFLVADRVIVTSKHNDDDQYVWESDAESFSVIKDPRGNTLGRGTTIS
LQLKEEAHDYLEESTLKNLVKKYSQFINFNIYLWTSQEQEVEEPVDNEEAKSETKAAEDDEEEGKVEEEEESDKPKTKKVKKTVWDWELMNNVTDE
EYNAFYKSISKESSEPMARIHFTAEGEVTFKSILFVPSTAPYDTFTNYGKKVDHIKMYVRRVFITDNFEDMMPKYLSFIRGVVDSDDLPLNVSRETLQQ
HKLLKVIKKKLVRKALDMIKKIDKEDYEKFWKEFSTNIKLGVIEDTSNRTRLAKLLRFFSSNSDTEQTFLADYVERMKEKQEAIYFIAGTSREEVEKSPFV
ERLLKKGYEVLYLVEPVDEYCIQSLPEFEGKKFQNIAKEGLNIDTSEKAKVRKEELEKEFKPLLDWLKDSALKDQIEKASISERLTNSPCALVASQYGWS
GNMERIMKSQAYAKQGDASNQYYANQKKTLEINPRHPLIKELKSRVQADSADDTARDLAKVLFDTATLRSGYSVKDSLDFAQRIERMMKMSMGL
DIDA

Supplementary figure 22. Proteomics annotation of HSP90 family proteins. Only 2 of the 3 annotated HSP90 family proteins were found in Bge cells, with Bge cell HSP90/84 being the most abundant and closely related to the snail sequences, relative to the glucose regulated protein 90. Shaded sequences represent Bge cell peptides with exact matches to the *B. glabrata* sequence. Underlined peptides identify peptides that contain mis-matched aa (designated in red). Underlined and shaded peptides indicate the presence of both identical and mismatched peptides in Bge cells.

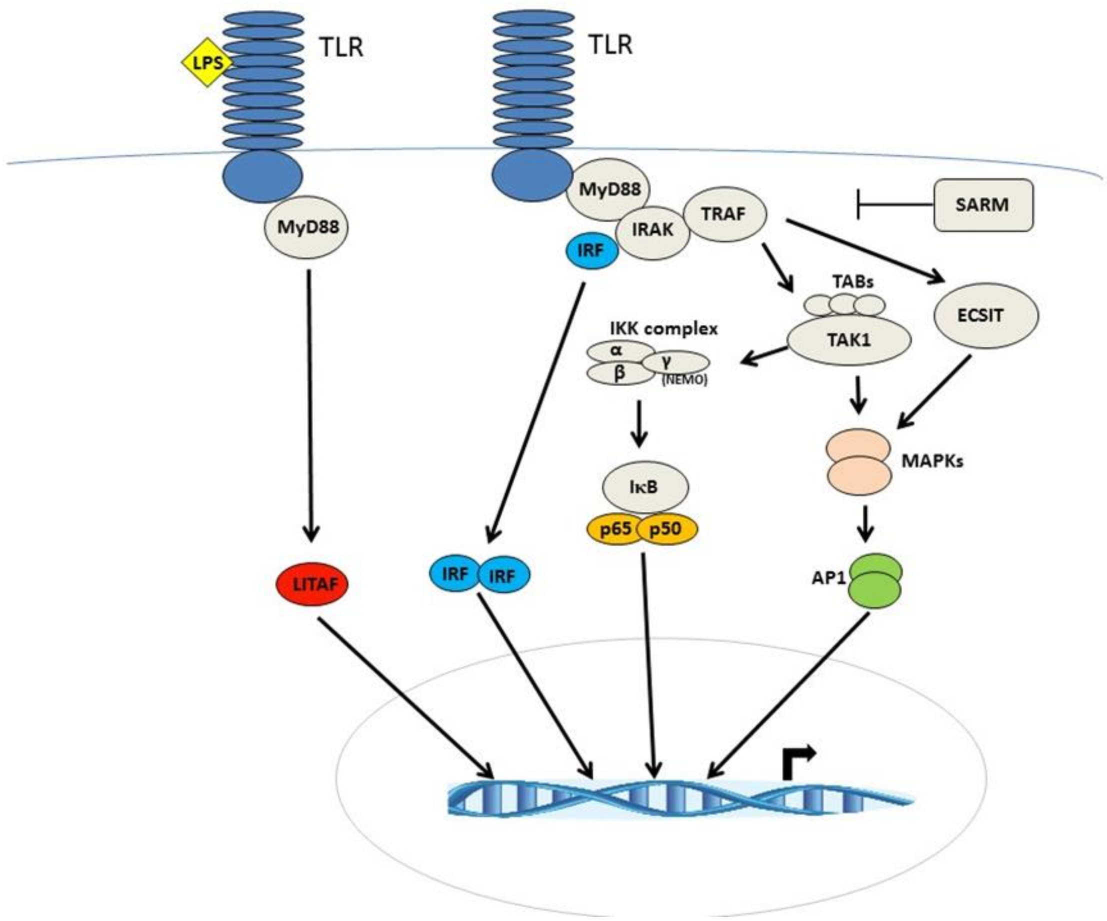

Supplementary Figure 23. TLR-associated signaling proteins identified in the *B. glabrata* genome. TLR receptors signal to several cytoplasmic factors to regulate activation of transcription factors that translocate to the nucleus and bind to regulatory sites to initiate gene expression.

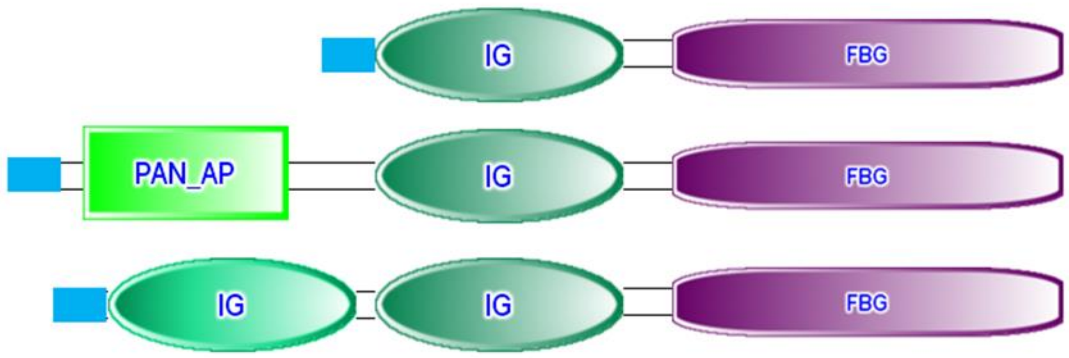

Supplementary Figure 24. Domain structure of *B. glabrata* FREP genes. Top: single IG domain FREP; center: single IG FREP with upstream sequence similarity to PAN_AP (green rectangle), a divergent subfamily of APPLE domains predicted to possess protein- and/or carbohydrate-binding functions, SMART accession SM00473; bottom: a dual IG FREP. Signal peptides are indicated in blue, Immunoglobulin superfamily domains are green ovals, purple: FBG fibrinogen-related domain.

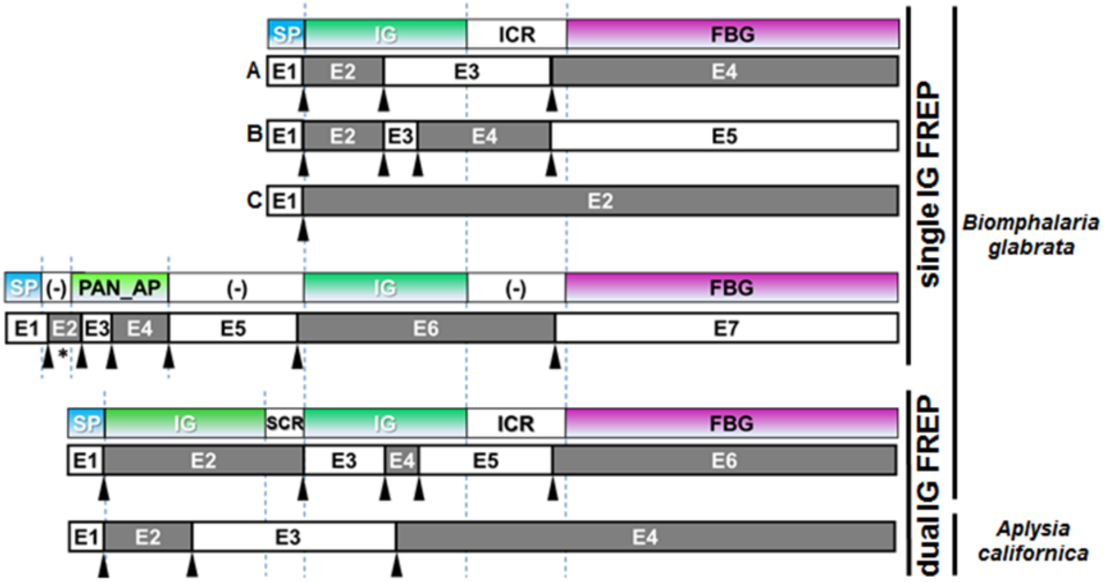

Supplementary Figure 25. Intron-exon structures of gastropod FREP genes.
Biomphalaria glabrata possesses two categories of FREPs with either single or dual upstream IG domains. Genome analysis revealed a novel type of single IG FREP gene that contains upstream sequence with similarity to PAN_AP domains. Only dual IG-type FREPs were reported from *Aplysia california*, sea hare. SCR: short connecting region; ICR: interceding region; (-): unnamed sequence region). Exons are numbered and indicated as alternating white and grey boxes, arrowheads indicate locations of introns. A,B,C indicate three alternative exon/intron structures for single IG FREPs.*: exon 2 not present on the final assembly of LG4_random_Scaffold480; yet it is present in several ESTs. Figure is not to scale.

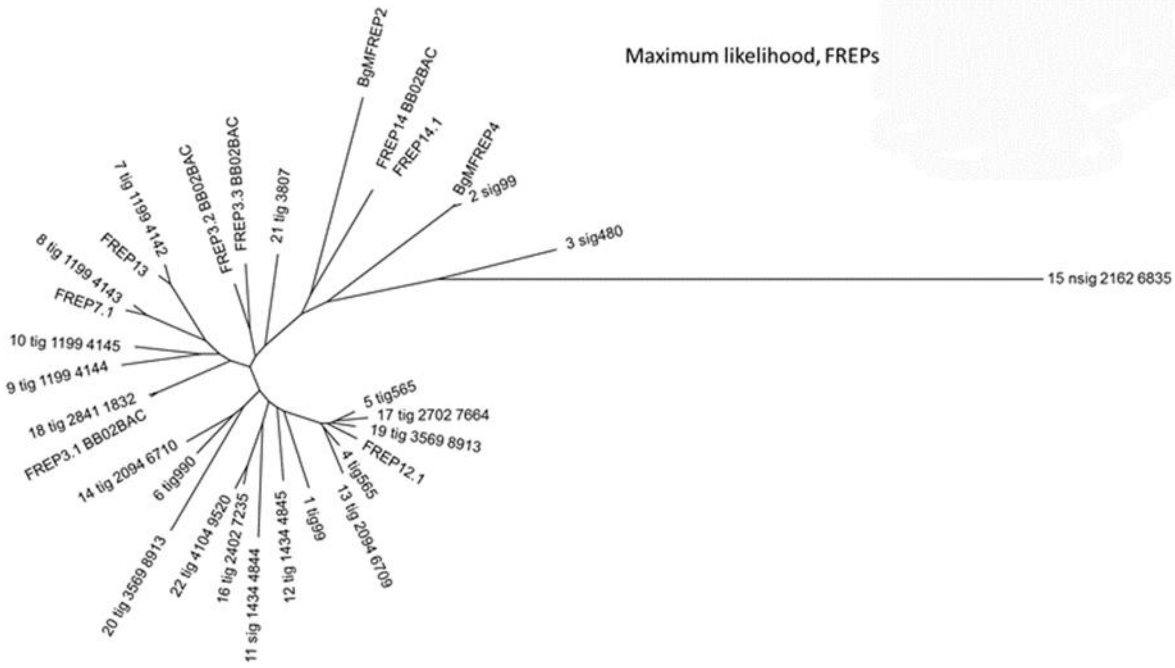

Supplementary Figure 26. Phylogenetic tree of FREPs
Phylogenetic tree based on alignment of coding sequences (nucleotides) of previously characterized FREPs (BgMFREP, FREP) and FREP genes recorded from the genome assembly. The latter are designated by a number (1-22), sig/tis (single/two IG domain) or nsig (novel single IG), scaffold number, gene model (if available). Note that FREP genes that occur on the same scaffolds (LGUN-random_Scaffold565 and LGUN-random_Scaffold1199) are highly similar.
This unrooted Maximum Likelihood gene tree was generated with MEGA (default settings, excluding gap columns).

Supplementary Figure 27. Alignment FREP Sequences.

Amino acid level alignments of coding sequences of previously characterized FREPs (BgMFREP, FREP with genbank accessions) and FREP genes recorded from the genome assembly. The latter are designated by a number (1-22), sig/tsi (single/two IG domain) or nsig (novel single IG), scaffold number, gene model (if available, note that genes model may be updated through user contributed annotation) or sequence interval. Sequences za_tig_4714; zb_tig_15796; x_tig_565_no-Cs; z_tig_6870_frameshifts; represent incomplete FREP gene-like sequences or possible pseudogenes that were included for comparison.

```
CLUSTAL W (1.8) multiple sequence alignment (BioEdit-generated mock-up)

BgMFREP2_AY012700          M~ASLPLRLVLLVSMATLIRS~~~~~~~~~~~~~~~~~~~~~~~~~~~~~
FREP3.1_BB02BAC_AEO50745   M~ARLFPRLVLCVFIVPLAGSELVIDVQPNVISPEITPQLVINCSVTNNQ
FREP3.2_BB02BAC_AEO50746   M~ERLFLLLVLCVFVVSLAGSELVIDVQPNVISPEITPQLVINCSITNNK
FREP3.3_BB02BAC_AEO50747   M~LLSLELIRKFPTDTRSSH~ELVIDAQPEVISLELTPQLVVNCSITDSH
BgMFREP4_AY012701          MKNLLLC~~~LFLVSATLGSR~~~~~~~~~~~~~~~~~~~~~~~~~~~~~
FREP7.1_AY028462           M~TNLLLRLVFFQSLLPLLSSELVIDVQPDIISAEITAQLVINCSVTNNQ
FREP12.1_AF515464          M~TLLQHFLLFVSLFLFSSS~ELVIDVQPNVISAEITAQLVINCSITNNQ
FREP13_AF515468            M~ATFLYVILVVALVTLSTS~ELVIDVQPNVISPEITAQLAINCSVTNNQ
FREP14_BB02BAC_AEO50744    MGALILQVILCAFIVPVISSR~~~~~~~~~~~~~~~~~~~~~~~~~~~~~
FREP14_EF467292            MGALILQVILCAFIVPVISSR~~~~~~~~~~~~~~~~~~~~~~~~~~~~~
1_tig_99_BGLB000116        M~ALLLHLALWASLVSLSTS~ELLIDVQPNVISEELTPKLVINCSISNNQ
1_sig_99_BGLB000152        MKNFLLCLSLVSLVSATLGSR~~~~~~~~~~~~~~~~~~~~~~~~~~~~~
3_sig_480_BGLB000179       M~KKNINTNIPETSSITTKTEDECVSKCLADSKCRLSRYDVSQRLCQLAS
4_tig_565_BGLB000073       M~ALFQHFLLFVSLFLLSTS~ELVIDVQPNVISAEITAQLVINCSITNNQ
5_tig_565_BGLB011626       ~~~~~~~~~~~~~~~~~~~~~~LVIDVQPNVISAEITAQLVINCSVTNNE
6_tig_990_BGLB014388       ~~~~~~~~~~~~~~~~~~~~~~LVIDVQPSVISLETTPQLVINCSITNNQ
7_tig 1199_BGLB000140      M~ATFLYVILVVALVTLSTS~ELVIDVQPNVISPEITAQLVINCSITNNQ
8_tig_1199_BGLB004529      M~TNLLLRLVFFQSILPLITSELVINVQPDIISAEITAQLVINCSVTNNQ
9_tig_1199_BGLB004530      M~AHMVHLTLCVVLVSLSSS~ELIIDVRPDIISLEITPQLVINCSITNNK
10_tig_1199_BGLB000019     M~AHIPHLVICLSLICMSTS~ELVIDVHPSVILPEITPQLVINCSITNNQ
11_sig_1434_BGLB000011     M~TVIIFNYKHLCLVIMFSGDK~~~~~~~~~~~~~~~~~~~~~~~~~~~~
12_tig_1434_BGLB000019     M~ENLRLLFCALLFSVTSS~~ELTIDIQPHGISLELTPKLVINCSITNKQ
13_tig_2094_BGLB007076     ~~~~~~~~~~~~~~~~~~~~~~LVIDVQPNVISAEITAQLVINCSITNNQ
14_tig_2094_BGLB000100     M~AFFLRLALWASLVFWSAS~ELVIDVQPNVILPEITAQLVINCSITNNQ
15_nsig_2162_BGLB000141    M~DFLKKAVIFLLTACFYVSAE~~~~~~~~~~~~~~~~~~~~~~~~~~~~
16_tig_2402_BGLB007591     ~~~~~~~~~~~~~~~~~~~~~~LIINVQPEVISPVFTSQLVINCSVTNNQ
17_tig_2702_BGLB000021     ~~~~~~~~~~~~~~~~~~~~~~LVIDVQPNVISAEITAQLVINCSITNNE
18_tig_2841_BGLB000096     M~ARLFPRLVLCVFVVPLISSELVIDVQPNVISSEITAQLVINCSITNNQ
19_tig_3569_BGLB009245a    ~~~~~~~~~~~~~~~~~~~~~~LVIEVQPNVISAEITAQLVINCSITNNQ
20_tig_3569_BGLB009245b    ~~~~~~~~~~~~~~~~~~~~~~LVIDVQPNVILPEITPHLVINCSITNNQ
21_tig_3807_BGLB000074     ~~~~~~~~~~~~~~~~~~~~~~LVIDVQPDVISAELTTQLVVNCSITNNH
22_tig_4104_BGLB000133     M~AFLLSLLLGVYLAPLALS~DLIINVQPEVISPVFTSQLVINCSVTNNQ
za_tig_4714                ~~~~~~~~~~~~~~~~~~~~~~~~~~~~~~~~~~~~~~~~~~~~~~~~~~
zb_tig_15796               M~AFFLLLALWASLVFWSAS~ELVIYVQPNVILPEITAQLVINCSITNNQ
x_tig_565_no-Cs            ~~~~~~~~~~~~~~~~~~~~~~~~~DVQPDVISPELTSQLLVNCSVSDNH
z_tig_6870_frameshifts     MCFYSSDV~~~~~~~~~~~~~ELVIDVQPSVISPEITAQLVINCSITNNQ

BgMFREP2_AY012700          ~~~~~~~~~~~~~~~~~~~~~~~~~~~~~~~~~~~~~~~~~~~~~~~~~~
FREP3.1_BB02BAC_AEO50745   VQHLEVIKSLTLSRYNEIIREFDELIALDSLTQNLKQFVRFKYSQISF~~
FREP3.2_BB02BAC_AEO50746   VQQLDLIKSLTLSRYNETIRDFDELIALDSLTLNLKQFVRFKYSQISF~~
FREP3.3_BB02BAC_AEO50747   VPGLDTINSLSLSRYNETKKEFDVLLSLDTHTLSLQQLVQFRHAQISF~~
BgMFREP4_AY012701          ~~~~~~~~~~~~~~~~~~~~~~~~~~~~~~~~~~~~~~~~~~~~~~~~~~
FREP7.1_AY028462           VQHLDVIRSLTLSRYNQTLRDFEDITALDLLTLNLKQLVKFKHSHISF~~
FREP12.1_AF515464          VQHLDVIRSLTLSRYDETLKEFLDLITLEAKTLNLSQLVQLHHAQISF~~
FREP13_AF515468            AQNIDVIKSLTLSRYNETIRDFEVMXXLDLLTLNLKQLVQFNYSLISF~~
FREP14_BB02BAC_AEO50744    ~~~~~~~~~~~~~~~~~~~~~~~~~~~~~~~~~~~~~~~~~~~~~~~~~~
FREP14_EF467292            ~~~~~~~~~~~~~~~~~~~~~~~~~~~~~~~~~~~~~~~~~~~~~~~~~~
1_tig_99_BGLB000116        VQQIDMIKSLSLARYNENIKEFEVLYYLEAATLNLTQHVQFQRSQIGF~~
1_sig_99_BGLB000152        ~~~~~~~~~~~~~~~~~~~~~~~~~~~~~~~~~~~~~~~~~~~~~~~~~~
3_sig_480_BGLB000179       EEVLNKTYFEFGVNVFIKECTEHQTKITTTATSTSTSETTTMSTSTRIEA
4_tig_565_BGLB000073       VQHLDVIRSLTLSCYNETLKEFFDLLTLEAKTLNLSQLVQLHHAQISF~~
5_tig_565_BGLB011626       VQQLDVIRSLTLSRYNETLKEFLDLLTLEAKTLNLSQLVQLHHAQISF~~
6_tig_990_BGLB014388       VQHLDVIKSLTLSRNNETLKEFEDLLVLNSSTLNLKQFKPLPNSQISF~~
7_tig 1199_BGLB000140      VQNIDVIKSLTLSRYNETIRDFEVMIALDLLTLNLKQLVQFKYSLISLS~
8_tig_1199_BGLB004529      VQHMEVIRSLTLSRYNQTLRDFKDITALDLLTLNLKQLVKFKHSQISF~~
9_tig_1199_BGLB004530      VQHLDVMNSLTLSRYDKTIRDFIALITLDSSTLNLKQLTKFSNSQVSF~~
10_tig_1199_BGLB000019     VQHIQVIKSLTLSRFNETIREFEDLVALDSSTLDLKKLMPFKYSQISF~~
11_sig_1434_BGLB000011     ~~~~~~~~~~~~~~~~~~~~~~~~~~~~~~~~~~~~~~~~~~~~~~~~~~
12_tig_1434_BGLB000019     VQHLQVIKSLTLSRYQESLKEFEILLSLEAKTRNLTQLVQLQRSQINV~~
13_tig_2094_BGLB007076     VQHLDVIRSLTLSRYNETLKEFFDLITLEAKTLNLSQLVQLHHAQISF~~
14_tig_2094_BGLB000100     VQQIEVIKCLTLSRYNETIKEFDDLFVLNSSTLDLKQLQQFQFSQISF~~
15_nsig_2162_BGLB000141    ~~~~~~~~~~~~~~~~~~~~~~~~~~~~~~~~~~~~~~~~~~~~~~~~~~
```

```
16_tig_2402_BGLB007591      VPNIDVIKSVSLSRYNETIKDFYVLLSLDTHTFNLQQFVQFRHAQVSF~~
17_tig_2702_BGLB000021      VQPLDVIRSLTISRYNETLKEFLDLLTLEAKTLNLSQLVQLHHAQISF~~
18_tig_2841_BGLB000096      VQYLDVIKSLTLSRYNETIRDFDELIALDSLTQNLKQFVRFKYSQISF~~
19_tig_3569_BGLB009245a     VQHLDVIRSLTLSRYNETRKEFFDLLTLESKTLNLSQLVQLHHAQISF~~
20_tig_3569_BGLB009245b     VQQLDLIKSLTLSRYNETSKEFQEMLTLDALPLILKLPTELNDSQIRF~~
21_tig_3807_BGLB000074      VAELDVIKYLTLSSYNESIKYFEDLLTLEANTFNLQKIRQFKHSQINS~~
22_tig_4104_BGLB000133      VPNIDVIKSVSLSRYNETIKDFYVLLSLDTQTFNLQQFVQFRHAQVSF~~
za_tig_4714                 ~~~~~~~~~~~~~~~~~~~~~~~~~~~~~~~~~~~~~~~~~~~~~~~~~~
zb_tig_15796                AQQLDVIKSLTLSRYNETITKYEDLFALNSSTLDLKQLLELKYSQISF~~
x_tig_565_no-Cs             VPGLIAINSLSLSIYNKTNNKFDVLLLLDTHKLSLRQLFQFQHAQISF~~
z_tig_6870_frameshifts      AQHIKVIKSLTLSRYNETIREFEDLFVLNTLTLILNQQTKLKVSQIRY~~

BgMFREP2_AY012700           ~~~~~~~~~~~~~~~~~~~~~~~~~~~~~~~~~~~~~~~~~~~~~~~~~~
FREP3.1_BB02BAC_AEO50745    ~GNLYITLTLPNPTQFDARIYRCNADGANSEGTNISLFTKKAVEYETNST
FREP3.2_BB02BAC_AEO50746    ~GNRYITLILHNPTQFDARIYKCNATGDNSEGANISLFAKKGVEYETNST
FREP3.3_BB02BAC_AEO50747    ~GNLYVTLTLRNPTQSDAKVYRCNVSGDDSLWKNITRVFKKEIKYETNLT
BgMFREP4_AY012701           ~~~~~~~~~~~~~~~~~~~~~~~~~~~~~~~~~~~~~~~~~~~~~~~~~~
FREP7.1_AY028462            ~GNVFISLTLLYPTQFDANVYRCSVKGGDPNNKDMSLFSKKTVEYETNST
FREP12.1_AF515464           ~GNLSISLTLHNPTQFDAKVYRCKVEGDKTNAASSSIVAKKEVEYRTNMT
FREP13_AF515468             ~GNVFISLTILHPTKSDAKVYRCSVKGDNSNERNISLFAKKAVEYETNST
FREP14_BB02BAC_AEO50744     ~~~~~~~~~~~~~~~~~~~~~~~~~~~~~~~~~~~~~~~~~~~~~~~~~~
FREP14_EF467292             ~~~~~~~~~~~~~~~~~~~~~~~~~~~~~~~~~~~~~~~~~~~~~~~~~~
1_tig_99_BGLB000116         ~GNKYITLTLQEPTQSDARVYRCNVIGDNAKVTNISVVSKKEVRYETIST
1_sig_99_BGLB000152         ~~~~~~~~~~~~~~~~~~~~~~~~~~~~~~~~~~~~~~~~~~~~~~~~~~
3_sig_480_BGLB000179        KTTTATEPTAVDATSPVTTTEPTTTTTMNTYSSLPPNTKATQDTPTSTNV
4_tig_565_BGLB000073        ~GNVYISLTLHNPTQFDAKVYRCKVEGDKTNAANSSIVAKKEVEYRTNTT
5_tig_565_BGLB011626        ~GNLSISLTLHTPTQFDAKVYRCKVEGDKTNAANSSIVAKKEVEYRTNTT
6_tig_990_BGLB014388        ~GNLYITLTLHNPTEFDAKVYKCNATGDNAEGTNISLFVLKAVEYETNST
7_tig_1199_BGLB000140       ~GNVFISLTILHPTKSDAKVYRCSVNGDNSNERNISLFAKKAVEYETNST
8_tig_1199_BGLB004529       ~GNVFISLTLLYPTQFDSNVYRCSVKGGDSNNKDMSLFSKKTVEYETNST
9_tig_1199_BGLB004530       ~GNLYLALTLHYPTQFDAKVYRCSVNGNNANGTDVSLSAKKGVEYETNST
10_tig_1199_BGLB000019      ~GNLYITLTLYNPTEFDAEVYRCMVNGDTSNWTNISLFAKKSVEHETNST
11_sig_1434_BGLB000011      ~~~~~~~~~~~~~~~~~~~~~~~~~~~~~~~~~~~~~~~~~~~~~~~~~~
12_tig_1434_BGLB000019      ~GDFYLALTLHNPSKFDARVYRCKAEGDTPQGTNSSSVVKTEVHYTINST
13_tig_2094_BGLB007076      ~GNLSISLTLHNPTQFDAKVYRCKVEGDKTNAENSSIVAKKEVEYRTNMT
14_tig_2094_BGLB000100      ~GNLYITLTLNNPSHFDAKVYRCNATGDNADGTSISLFAKMAVEYETSST
15_nsig_2162_BGLB000141     ~~~~~~~~~~~~~~~~~~~~~~~~~~~~~~~~~~~~~~~~~~~~~~~~~~
16_tig_2402_BGLB007591      ~ENLFLSLTVYNPIQSDAQAYRCNVDGDNSVQKNVSMKAKKEVRYEPNVT
17_tig_2702_BGLB000021      ~GNLSISLTLHNPTQFDAKVYRCKVEGDKTNAANSSIVAKKEVEYRTNTT
18_tig_2841_BGLB000096      ~GNLYITLTLHNPTQFDARIYRCNATGANSDGTNISLFAKKAVEYETNST
19_tig_3569_BGLB009245a     ~GNLYISLTLHNPTQFDAKVYRCKVEGDKTNAANSSIVAKKEVEYRTNTT
20_tig_3569_BGLB009245b     ~GNLYVTLTLHKPTLYDANVYRCNATGENANRTKKTVLAKKTVEFETNST
21_tig_3807_BGLB000074      ~GNLFITLTLQDPTQFDAKVYRCIVTGDASNATNISFVSKKEVKHETIST
22_tig_4104_BGLB000133      ~GNLFLSLTVYNPVQSDAQAYRCNVDGDNSVQKNVSMKAKKEVRYEPNVT
za_tig_4714                 ~~~~~~~~~~~~~~~~~~~~~~~~~~~~~~~~~~~~~~~~~~~~~KLYIY
zb_tig_15796                ~GNMYITLTLHNPTEFDAKVYRCHVTGDTAEGRNISLFAKKA~EYESNLT
x_tig_565_no-Cs             ~GNLYVSLIIYNPTESDARVYRCNVEGDTSFGKNISIVAKKTVEFRGNVT
z_tig_6870_frameshifts      ~GNSHITLTLHNPSKFDAKIYKCNATGDNAEGTNISLFAKKAVEYETNTT

BgMFREP2_AY012700           ~~~~~~~~~~~~~~~~~~~~~~~~~~~~~~~~SSWLNFTGNSETIRELIQ
FREP3.1_BB02BAC_AEO50745    ALIEEIRRIKKDE~~NKCSLKKDDLSDIKQ~~RWRVYFSESSKIIKERIE
FREP3.2_BB02BAC_AEO50746    ALIEEIRRIKKDE~~NYCSFKKDDLSDSKQ~~RSRVYFSGSSDIIKERIE
FREP3.3_BB02BAC_AEO50747    VLLEEIRRLREEK~~DRDQLSCQKEKLND~~~~SKLHFVGNSKVVKELFD
BgMFREP4_AY012701           ~~~~~~~~~~~~~~~~~~~~~~~~~~~~~~~~~~~LSFNANVEKINEVIQ
FREP7.1_AY028462            ALVEEIRRYKIDENKCLCSLASNDRTSTN~~KRLRVNFSGNSEIIKERVE
FREP12.1_AF515464           ALIEEIRRLKVVXKNDQCSLKXEELTSYHQ~~KTKLHFVGSSKVIKELIE
FREP13_AF515468             ALIEEIQRCKKVKSKCQCSLENNDSSSNYK~~RSRVHFSGSSEIIKERIE
FREP14_BB02BAC_AEO50744     ~~~~~~~~~~~~~~~~~~~~~~~~~~~~~~~~~PKLKFSGNAEIIKEFIE
FREP14_EF467292             ~~~~~~~~~~~~~~~~~~~~~~~~~~~~~~~~~PKLKFSGNAEIIKEFIE
1_tig_99_BGLB000116         PLIDEIRRLKKEADKNQCSSTNGEITDNKKKVRSKLSFVGSSEVINEFIE
1_sig_99_BGLB000152         ~~~~~~~~~~~~~~~~~~~~~~~~~~~~~~~~~~~LSFNANVEKINEVIQ
3_sig_480_BGLB000179        VNMATTNPPTKPNLDNTSSKTIKVR~~~~~~~~YLRLAVTPSAIELGKTE
4_tig_565_BGLB000073        ALIEEIRRLKVVEKNDQCSLEKEELTSYHQ~~KTKLHFVGSSKVIKELIE
5_tig_565_BGLB011626        ALIEEIRRLKIVEKNDQCTLEKKDLTASQQ~~RTKLHFVGSSKVIKELIE
6_tig_990_BGLB014388        ALIEEMRRRKKDENSRQCYFNKHESSDLNQ~~RSRVHFYGSSDIIKERVE
7_tig_1199_BGLB000140       ALIEEIQRCKKVESKCQCSLENNDSSSNYK~~RSRVHFSGSSEIIKERIE
8_tig_1199_BGLB004529       ALIEEIRRYKIDENKCLCSLTSNDRT~~~~~~RLRVHFSGNSEIIKESVE
9_tig_1199_BGLB004530       ALIEEIRRLKKDEDTYKCSFKKTDRPG~~~~~SRVHFFA~SSEIIKERIE
10_tig_1199_BGLB000019      ALIEKIRNFKKDEVNSKCTLQKNDHTG~~~~~SKVHFFA~SSDIFKESIE
11_sig_1434_BGLB000011      ~~~~~~~~~~~~~~~~~~~~~~~~~MRVIQ~~KPRLNFIANSDVIKELIQ
12_tig_1434_BGLB000019      ALVEEIRRLKEDENKDKCFPKKEEITGYYQ~~RSRLHFVASSRIVTELLD
13_tig_2094_BGLB007076      ALIEEIRRLKIVEKNDQCILEKKDLTASQQ~~ITKLHFVGSSKVIKELIE
14_tig_2094_BGLB000100      AFIEEIRRLKQDK~~NNCQSSLRSQ~~~~~~~KSRLQFDGSSEIIRELIE
15_nsig_2162_BGLB000141     ~~~~~~~~~~~~~~~~~~~~~~~~~~~~~~~KVTAEIAALPQKIRIGLTT
16_tig_2402_BGLB007591      ALIEKITRLMISKEIEQCSSMNVGISGYNN~IRSKLRFVASSEIVKELIE
17_tig_2702_BGLB000021      ALIEEIRRLKVVEKNDQCTLEKIELIASHQ~~KTKLHFVGSSKVIKELIE
18_tig_2841_BGLB000096      ALIEEIRRIKKDE~~NKCSLKKDDLSDIKQ~~RWRVYFSESSKIIKERIE
19_tig_3569_BGLB009245a     ALIEEIRRLKVVEKNDQCSLKNEKLTSHHQ~~KTKLNFVGSSKVIKEMIE
20_tig_3569_BGLB009245b     VLMEEMRRLKKNDNKCLCPLKKEERSDVIQ~~RSRIQFYESSEIIKEQLE
21_tig_3807_BGLB000074      TLLEQVRRLKKDIDRDQSPCKKEEPTVHQK~QKSKLDFVGSSEVIKELIE
22_tig_4104_BGLB000133      ALIEKITRLMISEDIEKCSLKNVELSGYNN~KISKLNFLGSSEIVKELIE
za_tig_4714                 YAILIFSFQLGIFQDKKCNNLFIKSLKSS~~~RSKVQFYRSSEIVQELIE
```

```
zb_tig_15796               ALIEELRRYKKNENFQCCLKKDEQSLVNQ~~~RSRVSFYGSLGIIRALIE
x_tig_565_no-Cs            ALVEEIRRLKTNYENDLCSLKRDELNSKNN~QRSRLLFVSNSKVIKELID
z_tig_6870_frameshifts     ALIEEIRRYKKNE~NFQCSLKKDEHSEVNQ~~RSRLHFYGSSEIITELIE

BgMFREP2_AY012700          PLKLTCTFQISKNDSDNDSQVLFMSIYHETKRVIASISKYQPVATSL~~~
FREP3.1_BB02BAC_AEO50745   PLTLKCTFQILTPDENETSRLQSLYILHESNGVVANINKDQAVITT~~~I
FREP3.2_BB02BAC_AEO50746   PLTLKCTFQVLKTDQNEISRLQSLYILHETKGVIAYVNKDQPVVTS~~~L
FREP3.3_BB02BAC_AEO50747   PLTLTCSIQDLMNDRNETSTVQSIYILHEANGIIATISKDQPVVTT~~~N
BgMFREP4_AY012701          PLMLTCSFEVSRNDSWQNTKVQLMYIMHETKGFVATITKDQNITGN~~~~
FREP7.1_AY028462           TLTLNCTFQVLNQDQNETSSLQSLYILHETNGVIANINKGQPV~~~~~~L
FREP12.1_AF515464          PLTLTCSLQNLDN~~~~NSTVQFMYILHESNGVIATINKDQPVVTT~~~K
FREP13_AF515468            TVTLNCTYQALKHQRNETS~LQSLYILHEANGVIANINKGQPVV~~~~~~
FREP14_BB02BAC_AEO50744    PLILSCSLKSFNNETNEHLKVHIMFIQHETNGVISTISKDQAVAVS~~~~
FREP14_EF467292            PLILSCSLKSFNNETNEHLKVHIMFIQHETNGVISTISKDQAVAVS~~~~
1_tig_99_BGLB000116        PFTLTCSCQVSNLHPDTNYTVEFMYILHETNGVIATINKGQLVVTA~~~I
1_sig_99_BGLB000152        PLMLTCSFEVSRNDSWQNTKVQLMYIMHETKGFVATITKDQNITGN~~~~
3_sig_480_BGLB000179       ALLIFC~SLPESSSTPFQNLVSL~KLSHSIDKDVYPF~KDLASINVADGR
4_tig_565_BGLB000073       PLTLTCSLQNLDH~~~~NSTVQFMYILHESNGVIATINKDQPVVTT~~~K
5_tig_565_BGLB011626       PLTLTCSLQDLDR~~~~NSTVQFMYILHEPIGVIATINKDQPVVTT~~~K
6_tig_990_BGLB014388       PWTLNCLYKFVNHDQKETPTLQSLFILHETNGVLAYINKGQPAVSV~~~I
7_tig_1199_BGLB000140      TVTLNCTYQALKHQRNETS~LQSLYILHEANGVIANINKGQPVV~~~~~~
8_tig_1199_BGLB004529      PLTLNCTFQVLNQDQNETSSLQSLYILHETNGVIANINKGQPV~~~~~~L
9_tig_1199_BGLB004530      PLTLNCSFQ~~~~~~~~~~~~~~~~~~ETNGVIATINKGQPVLTTI~HG
10_tig_1199_BGLB000019     PLTLKCSFQAIKQDENETSTLQSLYILHESNRIIANINKGQQVLTP~~~S
11_sig_1434_BGLB000011     PLVIRCSAQTFYVERNRSVTVTFMNIIHETIGSLATVSKIQQYDAS~~~~
12_tig_1434_BGLB000019     PLTLKCFFQVSNLDSVHNFTVQFLYILHETKGVVATMSKNQPVLTT~~~I
13_tig_2094_BGLB007076     PLTLKCSLQDLDR~~~~NSTVQFMYILHETNSVIATINKDQPVVTT~~~K
14_tig_2094_BGLB000100     PLTLNCSFKFSNDDNQETSTLQSLFILHETKGVIAYISKGQPVVTA~~~M
15_nsig_2162_BGLB000141    DFSIRCLAIPNQHDTGLSKILTLSIKKVQNDVPVTLAVVTPLSAAN~~~V
16_tig_2402_BGLB007591     PLTVKCSFPASNDSRYQDSVLQFMYILHETTGVIATISRDQPVTKS~~~G
17_tig_2702_BGLB000021     PLTLICSFQNQDH~~~~NSTVQFMYILHEPNDVIATINKYQPVVTT~~~K
18_tig_2841_BGLB000096     PLTLKCTFQILTPDENETSRLQSLYILHETNGVVANINKDQAVITT~~~I
19_tig_3569_BGLB009245a    PLTLTCSLQNLDH~~~~NSTVQFMYILHEPNGVIATINKDQPVVTT~~~K
20_tig_3569_BGLB009245b    PLTLTCVYKLSNQDSNETSRLQSLFILHETNGVIAYINKGQPVVTT~~~T
21_tig_3807_BGLB000074     PLTLACTYKVMNNDHNQNSTVQSLYIFHETNGIIATINKGETVATN~~~~
22_tig_4104_BGLB000133     PLTVKCSFLASNASPYKDSVLQFMYILHETNGVIATISRDQPVTKS~~~G
za_tig_4714                PLILNCSFKYLNYDKKEDYTLQSLFLLHESNGVIAYINKGQTVVTA~~~I
zb_tig_15796               PLTLKCVYKFVDHDSKEPSTIQYMFILHESKGLIAYIYKDQPVVTV~~~I
x_tig_565_no-Cs            SLTLTCSFQISDDISFENATIQSMHIFHESNGVIAMISGDQPIVRT~~~G
z_tig_6870_frameshifts     PLTITCLYKTLNQGEKETSALQSLYILHETNGVIAYINKQQPAITT~~~I

BgMFREP2_AY012700          YPSVTKVQGHIYHSNESKDSYLQVTWTHPKLSESGKYFCLAHAWNSTSQN
FREP3.1_BB02BAC_AEO50745   QGGNFENAQGEISGDQSKESYLQVTWSNLKHSDSGKYFCEAHVKHSKGKA
FREP3.2_BB02BAC_AEO50746   QGSHIQDVEGEIYDNAIKDSYLQVTWSNLKHTESGKYFCEAHNQYSEGRI
FREP3.3_BB02BAC_AEO50747   QDVNLLAVIGKLHDDSSKNSYLQVTWSNPKFSESGKYFCGAHANNAQGRN
BgMFREP4_AY012701          ADMTFSEGQGTLNNEIDNTSFXQVTWKNASNELSGKYICVVHATNAEGKV
FREP7.1_AY028462           KGSHLKDAQGNIFDSGLKDSYLQVTWSNVKLSDSGKYFCEANVKHSDGRA
FREP12.1_AF515464          QESNFNTAKGVLSDTKSKASFIEVSWSYIKSSESGNYFCGAHVMGPDGRS
FREP13_AF515468            QGSNLKNAEGEIFHNESKDSYLQVTWSNLKFSESGKYFCEANVKHSDGRA
FREP14_BB02BAC_AEO50744    ADQSSTHAHGKIYNKDLQDSYLQVIWKNPKISESGKYFCLAYAKNSTGQD
FREP14_EF467292            ADQSSTHAHGKIYNKDLQDSYLQVIWKNPKISESGKYFCLAYAKNSTGQD
1_tig_99_BGLB000116        QDFGNKNVKGELSDTKLQDSYLRVTWKDLKSSDSGKYFCGAHVIDAEGRS
1_sig_99_BGLB000152        ADMTFSEGQGTLNNEIDNTSFLQVTWKNASNELSGKYICVVHATNAEGKV
3_sig_480_BGLB000179       VNAHNNEGSGGGVISNTDEPFLSFLFPFPYFHMAGDYRCLAQGNTSDNQP
4_tig_565_BGLB000073       QESNFNMAKGEHSDTKTKASFIEVSWSYLKSSESGNYFCGAHVMGTDGRS
5_tig_565_BGLB011626       QESNFNMAKGKLSDTKTKASFIEVSWSYLKSSESGNYFCGAHVMEPDGRS
6_tig_990_BGLB014388       QGVHSKNVKGELYGNESVASYLQIMWSNLKISDSGKYFCEAHVKYSEGRS
7_tig_1199_BGLB000140      QGSKLKNAEGEIFDNESKDSYLQVTLSNLKFSDSGKYFCEANVKHSDGRA
8_tig_1199_BGLB004529      KGSHLKDAQGNIFDSGLKDSYLQVTWSNVKLSDSGKYFCEANVKHSDGRA
9_tig_1199_BGLB004530      GHSNSRNAKGEIFDNELKDSYLQVTLSNLQFSESGKYFCEAHVKRSQGGV
10_tig_1199_BGLB000019     QSDNSKNIQGEIHDDGSKDSYLQVTWSNVKSSESGKYFCEANVKHSDGRA
11_sig_1434_BGLB000011     PDVSLNKVQGYLSNQGSAESYLQITWAHPTPLQTGKYTCVAHTINYVGIV
12_tig_1434_BGLB000019     NENNFKNVKGELFENELNDSYIEVTWSHLKSSESGKYFCGAHVTDSKGTS
13_tig_2094_BGLB007076     QESNFNTAKGVLSDTKTKASFIEVSWSYLKSSESGKYFCGAHVMGPDGRS
14_tig_2094_BGLB000100     EEVKSKNVKGEIYQNKLKDSYLQVTWTNLKLSDSGKYFCEAHVQYLEERS
15_nsig_2162_BGLB000141    MPEARAAKAHGALDHST~~NYLNISWKNPESDLAGEYICEGIGVEASGKN
16_tig_2402_BGLB007591     QNLSSNTIQGELYHNLSKDSYLQVTWQNVKLFDSGKYFCGAHVNYVLGQK
17_tig_2702_BGLB000021     QESNFNMAKGELSDTKLQASYIEVSWSYIKSSESGNYFCGAHVMGPDGRS
18_tig_2841_BGLB000096     QGGNFENGQGEISGNQSKESYLHVTWSNLKHSDSGKYFCEAHVKHSKGKA
19_tig_3569_BGLB009245a    QESNLNMAKGKLSDTKLQASFIEVSWSYINSSESGKYFCGAHVMEPDGRY
20_tig_3569_BGLB009245b    KGENSKNVEGQIYDSELKESYLQVTWNNLIFSESGRYICEAYVKHSDGSY
21_tig_3807_BGLB000074     QEINSKNVKAELSSNESRDSYIQVTWSHLKLSEAGKYFCGASVKYSESKN
22_tig_4104_BGLB000133     QNLSSNTIQGELYHNLSKDSYLQVTWQNVKLSDSGKYFCGAHVNYVLGQK
za_tig_4714                KEITSQNVTGEIYDNKSIDSFLQVEWKNLKISDSGKYVCEAHVQYEEGKS
zb_tig_15796               QGVSAKHVKGQIYDNNFNDSYLQVTWTNLTLSDSGKYFCEAHVQYLEGRS
x_tig_565_no-Cs            LDITSPSIKGELYHNISKVTFLQLTWRKPTFLESGKYFCGAHVKISEKKS
z_tig_6870_frameshifts     QESNFKNVEGEIFDNESKDSYLQVTWNNLKHSESGNYFCEAHINRSDGRF

BgMFREP2_AY012700          SVFDADITVNVIKSSTDDLAVALSYIQDRL~D~K~~~~~~~~~~~~~~~~
FREP3.1_BB02BAC_AEO50745   ERLNEMLTIEVISPTIDDLMEVIQKLVTQV~D~GDKESLQDVKQNIMNIK
FREP3.2_BB02BAC_AEO50746   DKTSNMLTITVERPTFDDLVEAMHKLFTQV~D~GAKESLKAINQNIKNIN
FREP3.3_BB02BAC_AEO50747   EHWNEMLTITVERLQFDDIVKVMYDIQRQV~D~EDKKRLQTFHENLTNNF
```

```
BgMFREP4_AY012701           EFLSASLKVQVQKLEIADLAQYVVDLTARVKESDDKIQN~~YTRNVTSIK
FREP7.1_AY028462            ERLSEMLILTVVSPTVDDLVKVIEKLLGQV~D~EDTKHIQENKQNIKNIK
FREP12.1_AF515464           ERLNEMLAITVSNPTFDDVVKVIPKLLRQA~D~IEKENILENKQNIYDIK
FREP13_AF515468             ERLSEMLIITVVSPTFDDLVKVIEKLLGQV~D~GDTRHIQENNQSIKNIK
FREP14_BB02BAC_AEO50744     SVFQSTVTIKVLKPKADDLVQVLGQLLKRV~D~TLEQ~LLEGNETKLRGQ
FREP14_EF467292             SVFQSTVTIKVLKPKADDLVQVLGQLLKRV~D~TLE~QLLEGNETKLRGQ
1_tig_99_BGLB000116         ERLNEMVTVKVKLPTFDDLVNVIQKLLILV~Y~DDREILQDNEQNIKSIQ
1_sig_99_BGLB000152         EFLSASLKVQVQKLEIADLAQYVVDLTARVKESDDKIQN~~YTRNVTSIK
3_sig_480_BGLB000179        LT~~~~~~~~~~~LTAIGKVFIEYNSLVSI~SKTLRSLRLEQIQNENKTR
4_tig_565_BGLB000073        ELLNEVLAITVSNPTFDDLVKVIPKLLRQA~A~KDKENILENKHNIYHIK
5_tig_565_BGLB011626        ERLNEMLAITISNLTFDDLVKVIQKLLRQA~D~IEKEKILENKQNIYHIK
6_tig_990_BGLB014388        EKFNEMLTITVQSSTLNDLVNVIEKLITQV~D~EDKESIQVNKQNIENLK
7_tig 1199_BGLB000140       ERLSEMLILTVVSPTVDDLVKVVEKLLGQV~D~GDTKHIQENNQSIKNIK
8_tig_1199_BGLB004529       ERLSEMLILTVVSPTVDDLVKVIEKLLGQV~D~EDTKHIQENKQNIKNIK
9_tig_1199_BGLB004530       ERLNKMLAISVLSPTFEDLVKVIEKVLRKS~D~EDNIRIRENGQTLRNIQ
10_tig_1199_BGLB000019      ERLSEMLTITVVRPTFDDLVKVVEKLLEQF~N~EYKTHLQENVEKNRAML
11_sig_1434_BGLB000011      EKLEAALSIEVKRTSFDDLVDVVIDVLENL~D~DEKTKVKMLQQKVDKGQ
12_tig_1434_BGLB000019      ERLNEMLTITVTGPTLEDLVKVLQKLLAQK~D~EDKKIEKEN~~~~IKRI
13_tig_2094_BGLB007076      ERLNEVLTIIVLNPTFDDLVKVIPKLLRQA~D~KEKENILENKQNIYHIK
14_tig_2094_BGLB000100      EKFNEMLTITVQSPTLNDIVNVIQKLITQV~D~EDKESIQVSKQNIENIK
15_nsig_2162_BGLB000141     IAFSDSIFVSYEYASTEEYVSTLFQLHKEL~D~QSQAKLKAAYTAGNKTQ
16_tig_2402_BGLB007591      DRFQEQLVISVQRPTLDDLVKVVHDLQREV~G~EEKQRREISERNIMNIH
17_tig_2702_BGLB000021      ERLNEMLAIIVSNPTFDDLVKVIPKLLRQS~N~KDKENILENKQNIYNIK
18_tig_2841_BGLB000096      ERLNEMLTIEVISPTIDDLMEVIQKLVTQV~D~GDKESLQDVKQNIKNIK
19_tig_3569_BGLB009245a     ERLNEVLAIIVLNPTFDDLVKVIPKLLRQA~D~IEKENVLDNKNNIYHIK
20_tig_3569_BGLB009245b     DKLKEILTITVKSPTVDDLAKVVHMLLTQL~N~KDVSLTRSINY~~~~VK
21_tig_3807_BGLB000074      ENFNKMLTITITRPTFDDLVKVIQKILAKS~E~EDKELILGNIQS~~~IK
22_tig_4104_BGLB000133      DRFQEQLVISVQRPTFDDLVKVVHDLQREV~G~EEKQRREISERNIINI~
za_tig_4714                 EKVNEMLTITVQSPTLGDLVYVIQKLIAQA~D~EDKENVQASKQKIENIE
zb_tig_15796                EKD~~~~~~~~~~~~~~~~~~~~~~~~~~~~~~~~~~~~~~~~~~~~~~~
x_tig_565_no-Cs             DRLNKMLKIIVERPTIEDLFKSVFDLKTLS~D~EQNQSLHENEKKLKSIE
z_tig_6870_frameshifts      EKINEMLTIIVRGSTLHDLVKVIQKLQRQA~E~VDQESLRDNTQKLKTIK

BgMFREP2_AY012700           ~~~~~~~~~~~~~~~~~~~~~~~~~~~~~~~~~~~~~~~~~~~~~~~~~~
FREP3.1_BB02BAC_AEO50745    ED~~~LNTKEQNIISIKEDLNTKQQSIISIKEEFKTKQENIQ~~KD~VTI
FREP3.2_BB02BAC_AEO50746    KD~~~LDFKEQNITSIKEEVIRNQNNIQILSEDSNIKEQNMTSIREDLST
FREP3.3_BB02BAC_AEO50747    II~~~LNTNLQSIENVRRDVRTNQESINGIKDELLSNKQNIVNNKKDINT
BgMFREP4_AY012701           EE~~~LNALKENHLAALRSLDIIKK~~~~~~~~~~~~~~~~~~~~~~~~~
FREP7.1_AY028462            EE~~~LKTKEQNILSNTADLNSTQQTIRSMKEDIAINQHNMSSLKEFVDA
FREP12.1_AF515464           EY~~~FKSKQQNIISIKDGLNTNRHNIKSIADDLNVNKENIASHNDEINT
FREP13_AF515468             EE~~~LKIKEQNIISITADLNSTQQIISIIKEDITQNQQNISSMKEDLII
FREP14_BB02BAC_AEO50744     DD~~~RNVQITQKFSGLEALNLQRVNAD~~~~~~~~~~~~~~~~~~~~~~
FREP14_EF467292             DD~~~RNVQITQKFSGLEALNLQRVNAD~~~~~~~~~~~~~~~~~~~~~~
1_tig_99_BGLB000116         ND~~~LS~~~TNVLRINKDLDNYKENFIRINTDLDSKQQNIINIKQDLDN
1_sig_99_BGLB000152         EE~~~LNALKENHLAALRSLDIIKK~~~~~~~~~~~~~~~~~~~~~~~~~
3_sig_480_BGLB000179        QQDAIGTKQED~~~~~~~~~~~~~~~~~~~~~~~~~~~~~~~~~~~~~~~
4_tig_565_BGLB000073        ED~~~INSKQQNILSIKDGLDTNSQNIKSITDDLNANKQNIASHIDELNS
5_tig_565_BGLB011626        ED~~~INSKQQNIKSIKDGLDTNRQNINIIKDNLETSRQNIKSITDDLNA
6_tig_990_BGLB014388        ED~~~ANTKKQNLERIIEDIDTNKQNITSIKVVMDTNTRNIKKSRDEMD~
7_tig 1199_BGLB000140       EE~~~LKIKEQNIISITADLNSTQQIISIIKEDITQNQQNISSMKEDLII
8_tig_1199_BGLB004529       EE~~~LKTKEQNILSNTADLNSTQQTIRSMKEDIAINQQNISSIKTD~~~
9_tig_1199_BGLB004530       ED~~~SDTKEKNIKSIEEDLNTKQQNIISLKEDFNAFQRTMNTFREKLEV
10_tig_1199_BGLB000019      DR~~~NK~~~QTILLIKKDVLANQQSLQTMKEDWNSNQTNIISIKEELQS
11_sig_1434_BGLB000011      ~~~~~~~~~~~~~~~~~~~~~~~~~~~~~~~~~~~~~~~~~~~~~~~~~~
12_tig_1434_BGLB000019      DD~~~LETKQLNIISIKDDIDSYIQNMNKIKDELNSQKQSISSIRDDLRN
13_tig_2094_BGLB007076      VD~~~IKSKQENILSIKDELDTNSQNIKSITDDLNANKQSTLSYIDE~~~
14_tig_2094_BGLB000100      ED~~~VNTHHQDIKTIRTEKELNKENITSIKEVIDTITQNIMSIRVDTN~
15_nsig_2162_BGLB000141     SELVET~~~~~~~~~~~~~~~~~~~~~~~~~~~~~~~~~~~~~~~~~~~~
16_tig_2402_BGLB007591      EQNIEI~VKEDMKKHFRXKTNYTTLLQEI~~~~~~~~~~~~~~~~~~~~~
17_tig_2702_BGLB000021      ED~~~INSKQQNIISIKDGLDTNSQNINIIKDNLENSRQNIKNITDDLNA
18_tig_2841_BGLB000096      ED~~~LNTKEQNIISIKEDLNTKQQSIISIKEEFKTKQENI~~QKD~MTI
19_tig_3569_BGLB009245a     ED~~~INTKQQNIKSIKDGLDTNSQNIKSIKDGLDTNSQNIISIKDGLDT
20_tig_3569_BGLB009245b     ED~~~SDSNKENGKSKKEDRDTVKQN~~~IRDFQE~~~~~~~~~~~~~~~
21_tig_3807_BGLB000074      DD~~~LNANKQHIESVTEDLNDTKENMKNYKEEMKIIVANLSTTTTDISK
22_tig_4104_BGLB000133      ~~~~~~~~HEQNIENVKQDLKINQQNIKSVKEDVKINQQNIENVKEDVKI
za_tig_4714                 AD~~~LNTNNQKIQRIREEIDTNKQNITSIKEDINTNTQNVKSIKVNIDR
zb_tig_15796                ~~~~~~~~~~~~~~~~~~~~~~~~~~~~~~~~~~~~~~~~~~~~~~~~~~
x_tig_565_no-Cs             DD~~~FNKQADKLKQSLNDNE~~~QKLKSIEEDFNKQADKLKQS~~~LNA
z_tig_6870_frameshifts      ED~~~LDINIHNIINVKKDLNDK~~~~~~~~~~~~~~~~~~~~~~~~~~H

BgMFREP2_AY012700           ~~~~~~~~~~~~~~~~~~~~~~~~~~~~~~~~~~~~~~~~~~~~~~~~~~
FREP3.1_BB02BAC_AEO50745    NQQNIQKIKEELDSKEQSMIIIREDFSACQQNISAFKENIEIMFANLSTS
FREP3.2_BB02BAC_AEO50746    KQQTFLNIKEDVILNQQNIDKIKQDLNTYRHNM~SYIEEHLEVILAN~~~
FREP3.3_BB02BAC_AEO50747    AKESINVIKEELLSNKQNIVNNKRDIDTMEESINVIRHELLSNKQNIVNN
BgMFREP4_AY012701           ~~~~~~~~~~~~~~~~~~~~~~~~~~~~~~~~~~~~~~~~~~~~~~~~~~
FREP7.1_AY028462            NLESLQSIKEDLNIQQRNIISVKEDIAINQQNISSIKTDVAVNEENLINL
FREP12.1_AF515464           LRQMVNNVQSDLSICKKSIHNFSNDLDTKKQSIASHKDELNSFGQIVNSL
FREP13_AF515468             NQENLKNVKEDFNIQQRNILSLEKDFHTHQQNISNFQENLEIVLSNFSTA
FREP14_BB02BAC_AEO50744     ~~~~~~~~~~~~~~~~~~~~~~~~~~~~~~~~~~~~~~~~~~~~~~~~~~
FREP14_EF467292             ~~~~~~~~~~~~~~~~~~~~~~~~~~~~~~~~~~~~~~~~~~~~~~~~~~
1_tig_99_BGLB000116         QKLNIIRINEDLDSKEQQFTQFNIDLDSKQQTIIRIDKDLNATEHSIIRI
1_sig_99_BGLB000152         ~~~~~~~~~~~~~~~~~~~~~~~~~~~~~~~~~~~~~~~~~~~~~~~~~~
```

```
3_sig_480_BGLB000179        ~~~~~~~~~~~~~~~~~~~~~~~~~~~~~~~~~~~~~~~~~~~~~~~~~~
4_tig_565_BGLB000073        LKQMVNNVQGDISICKESIQRISNDLDTNKQSIERHNDELNSFGQIVNSL
5_tig_565_BGLB011626        NKQNIASHNNELNSLRQMVNNVQGDISICKESIHSISIDLDTNKQSIARH
6_tig_990_BGLB014388        TNITRIKEYMDINSRNMKMDID~~~~~~~~~~~~~~~~~~~~~~~~~~~~
7_tig 1199_BGLB000140       NQENLKNVKEDFNIQQRNILSLEKDFHTHQQNISNFQENLEIVLSNFSTA
8_tig_1199_BGLB004529       ~~~~~VAVNEENLIN~~~~~~LQKDFNIQQRNITSIKEDFNTFYKNTSSF
9_tig_1199_BGLB004530       ISANVSKIERERTE~~~~~~~DKE~~~~~~~~~~~~~~~~~~~~~~~~~~
10_tig_1199_BGLB000019      HRQNISTLKENFETVFSNFSTALIDIKNQIVKE~~~~~~~~~~~~~~~~~
11_sig_1434_BGLB000011      ~~~~~~~~~~~~~~~~~~~~~~~~~~~~~~~~~~~~~~~~~~~~~~~~~~
12_tig_1434_BGLB000019      NTQHMNSITDDLNVNGQNLKNIKDEININRQNISSIKADLNTNTQ~~~~~
13_tig_2094_BGLB007076      ~~~~~~~~~~~~~~~~~~~~~~~~~~~~~~~~~~~~~~~~~~~~~~~~~~
14_tig_2094_BGLB000100      NTITMLKTDMDTNAGNIEIFRVEINENITKLKEDLD~~~TNKQLLN~~~N
15_nsig_2162_BGLB000141     ~~~~~~~~~~~~~~~~~~~~~~~~~~~~~~~~~~~~~~~~~~~~~~~~~~
16_tig_2402_BGLB007591      ~~~~~~~~~~~~~~~~~~~~~~~~~~~~~~~~~~~~~~~~~~~~~~~~~~
17_tig_2702_BGLB000021      KKQNIASHNDELNTLRESVNSLETQ~~~~~~~~~~~~~~LANFSTALRQI
18_tig_2841_BGLB000096      NQQNIQKIKEELDSKEQSMIIIREVFSACQQNISAFKENIEIMFANLSNS
19_tig_3569_BGLB009245a     NSQNIKRIKDGLDTNSQNINIIKDDLETSRRNIKSITDDLNANKQSIASH
20_tig_3569_BGLB009245b     ~~~YPKLSVANISTSIVE~~~~~~~~~~~~~~~~~~~~~~~~~~~~~~~~
21_tig_3807_BGLB000074      ETDEERTEDKDKTY~~~~~~~~~~~~~~~~~~~~~~~~~~~~~~~~~~~~
22_tig_4104_BGLB000133      NQQNLKNVKEDLKINKDNIENVREEVKINQQNLTRLKETIDLLQ~YNLSN
za_tig_4714                 NITMLR~~ED~ILTNIH~~~DINEDLDTCKRDIGNIRMEMEGNTSMLKET
zb_tig_15796                ~~~~~~~~~~~~~~~~~~~~~~~~~~~~~~~~~~~~~~~~~~~~~~~~~~
x_tig_565_no-Cs             NGQKLKSIEEDFNKLADKQKQSLNDNEQKLKRIEED~~~LISNQQNLSIF
z_tig_6870_frameshifts      ENIIRINEN~~LDTKQQ~~~~~~~~DITCLKDNMNSTKQDIISLKDNMNS

BgMFREP2_AY012700           ~~~~~~~~~~~~~~~~~~~~~~~~~~~~~~~~~~~~~~~~~~~~~~~~~~
FREP3.1_BB02BAC_AEO50745    FMQVKTQI~~~~~~~~~~~~~~~~~~~~~~~~~~~~~~~~~~~~~~~~~~
FREP3.2_BB02BAC_AEO50746    ~~~~~~~~LSSASMKVKNQTDE~~~~~~~~~~~~~~~~~~~~~~~~~~~~
FREP3.3_BB02BAC_AEO50747    KKD~~~~~IHTTQESIIGILEE~~~LLRNQQNIVNNKKD~~~~~INTTQD
BgMFREP4_AY012701           ~~~~~~~~~~~~~~~~~~~~~~~~~~~~~~~~~~~~~~~~~~~~~~~~~~
FREP7.1_AY028462            QKD~~~~~FNIQQRNISSIKED~~~~~~~~~~~~~~~~~~~~~~~~~~~~
FREP12.1_AF515464           KDD~~~~~LRTNKQNLQSVTDE~~~ENTNKENINQLKED~~~~~FKSNKQ
FREP13_AF515468             LME~~~~~VKNQT~DKERKGDD~~~~~~~~~~~~~~~~~~~~~~~~~~~~
FREP14_BB02BAC_AEO50744     ~~~~~~~~~~~~~~~~~~~~~~~~~~~~~~~~~~~~~~~~~~~~~~~~~~
FREP14_EF467292             ~~~~~~~~~~~~~~~~~~~~~~~~~~~~~~~~~~~~~~~~~~~~~~~~~~
1_tig_99_BGLB000116         DKD~~~~~LNATEHSIIRINKD~~~LNATEHSIIRIDKD~~~~~LNATEH
1_sig_99_BGLB000152         ~~~~~~~~~~~~~~~~~~~~~~~~~~~~~~~~~~~~~~~~~~~~~~~~~~
3_sig_480_BGLB000179        ~~~~~~~~~~~~~~~~~~~~~~~~~~~~~~~~~~~~~~~~~~~~~~~~~~
4_tig_565_BGLB000073        KDD~~~~~LRTNKQSLQNITDV~~~VNTKKENINQLKED~~~~~LKSNKQ
5_tig_565_BGLB011626        NNE~~~~~LNSFRQKVNSLEDD~~~LRTNKQSLQSITYE~~~~~VNTNKQ
6_tig_990_BGLB014388        ~~~~~~~~~~~~~~~~~~~~~~~~~~~~~~~~~~~~~~~~~~~~~~~~~~
7_tig 1199_BGLB000140       LME~~~~~VKNQT~~DKERKGD~~~~~~~~~~~~~~~~~~~~~~~~~~~~
8_tig_1199_BGLB004529       QEIIIA~~NHSATLKKVKNETE~~~~~~~~~~~~~~~~~~~~~~~~~~~~
9_tig_1199_BGLB004530       ~~~~~~~~~~~~~~~~~~~~~~~~~~~~~~~~~~~~~~~~~~~~~~~~~~
10_tig_1199_BGLB000019      ~~~~~~~~~~~~~~~~~~~~~~~~~~~~~~~~~~~~~~~~~~~~~~~~~~
11_sig_1434_BGLB000011      ~~~~~~~~~~~~~~~~~~~~~~~~~~~~~~~~~~~~~~~~~~~~~~~~~~
12_tig_1434_BGLB000019      KID~~~~~LLTGNFNSNRLE~~~~~LMNIYKYLNSSQQSMTEFKLD~~~~
13_tig_2094_BGLB007076      ~~~~~~~~~~~~~~~~~~~~~~~~~~~~~~~~~~~~~~~~~~~~~~~~~~
14_tig_2094_BGLB000100      LQE~~~~~NLRMEVANISMD~~~~~LTKLKNKMDKVE~~~~~~~~~~~~~
15_nsig_2162_BGLB000141     ~~~~~~~~~~~~~~~~~~~~~~~~~~~~~~~~~~~~~~~~~~~~~~~~~~
16_tig_2402_BGLB007591      ~~~~~~~~~~~~~~~~~~~~~~~~~~~~~~~~~~~~~~~~~~~~~~~~~~
17_tig_2702_BGLB000021      KEE~~~~~IE~~~~~~~~~~~~~~~~~~~~~~~~~~~~~~~~~~~~~~~~
18_tig_2841_BGLB000096      FMQVKTQI~~~~~~~~~~~~~~~~~~~~~~~~~~~~~~~~~~~~~~~~~~
19_tig_3569_BGLB009245a     NDE~~~~~LNSLRQIVNSLKDD~~~LRTNKQSLQSIRDE~~~~~VNTNKE
20_tig_3569_BGLB009245b     ~~~~~~~~~~~~~~~~~~~~~~~~~~~~~~~~~~~~~~~~~~~~~~~~~~
21_tig_3807_BGLB000074      ~~~~~~~~~~~~~~~~~~~~~~~~~~~~~~~~~~~~~~~~~~~~~~~~~~
22_tig_4104_BGLB000133      YEG~~~~~~~~~KTNYTSLLQE~~~~~~~~~~~~~~~~~~~~~~~~~~~~
za_tig_4714                 ~~~~~~~~LNINTRNIKNLEVVID~~~~~~TSNTQIKKD~~~~~ININIQ
zb_tig_15796                ~~~~~~~~~~~~~~~~~~~~~~~~~~~~~~~~~~~~~~~~~~~~~~~~~~
x_tig_565_no-Cs             RNE~~~~~TKKLVAELSNSQIEWKGQIDKGSKPTFKFRMKALTSAVGWFT
z_tig_6870_frameshifts      TKQD~~~~IVSLKDNMNSTKQDIISLKDNMNSTKQDIVS~~~~~LKDNMN

BgMFREP2_AY012700           ~~~~~~~~~~~~~~~~~~~~~~~~~~~~~~~~~~~~~~~~~~~~~~~~~~
FREP3.1_BB02BAC_AEO50745    ~NKGNQED~~~~~~~~~~~~~~~~~~~~~~~~~~~~~~~~~~~~~~~~~~
FREP3.2_BB02BAC_AEO50746    ~~~~~~~~~~~~~~~~~~~~~~~~~~~~~~~~~~~~~~~~~~~~~~~~~~
FREP3.3_BB02BAC_AEO50747    SIREIQEE~~~~~LLRNQQNIENAKEE~~~~~LKSKQDSMKSIQDE~~~~
BgMFREP4 _AY012701          ~~~~~~~~~~~~~~~~~~~~~~~~~~~~~~~~~~~~~~~~~~~~~~~~~~
FREP7.1_AY028462            ~~~~~~~~~~~~~~~~~~~~~~~~~~~~~~~~~~~~~~~~FKTFYKNTSS
FREP12.1_AF515464           KIQNITQE~~~~~VNANRQNIINLNNTSQQSLSKLEADLETQLTNLSTAL
FREP13_AF515468             ~~~~~~~~~~~~~~~~~~~~~~~~~~~~~~~~~~~~~~~~~~~~~~~~~~
FREP14_BB02BAC_AEO50744     ~~~~~~~~~~~~~~~~~~~~~~~~~~~~~~~~~~~~~~~~~~~~~~~~~~
FREP14_EF467292             ~~~~~~~~~~~~~~~~~~~~~~~~~~~~~~~~~~~~~~~~~~~~~~~~~~
1_tig_99_BGLB000116         SIIRINKD~~~~~LDSKQQTIFSIQQDCKSTKKDFNEYKQNMSIFKETQE
1_sig_99_BGLB000152         ~~~~~~~~~~~~~~~~~~~~~~~~~~~~~~~~~~~~~~~~~~~~~~~~~~
3_sig_480_BGLB000179        ~~~~~~~~~~~~~~~~~~~~~~~~~~~~~~~~~~~~~~~~~~~~~~~~~~
4_tig_565_BGLB000073        KIQNITQK~~~~~VNTNRHSIINLNKD~~~~~~~~~~~~~~~~~~~~~~~
5_tig_565_BGLB011626        NINQLKED~~~~~LKSNEQKIQNITED~~~~VNTNRLNIMNINKD~LNTS
6_tig_990_BGLB014388        ~~~~~~~~~~~~~~~~~~~~~~~~~~~~~~~~~~~~~~~~~~~~~~~~~~
7_tig 1199_BGLB000140       ~ ~~~~~~~~~~~~~~~~~~~~~~~~~~~~~~~~~~~~~~~~~~~~~~~~~
8_tig_1199_BGLB004529       ~~~~~~~~~~~~~~~~~~~~~~~~~~~~~~~~~~~~~~~~~~~~~~~~~~
9_tig_1199_BGLB004530       ~~~~~~~~~~~~~~~~~~~~~~~~~~~~~~~~~~~~~~~~~~~~~~~~~~
10_tig_1199_BGLB000019      ~~~~~~~~~~~~~~~~~~~~~~~~~~~~~~~~~~~~~~~~~~~~~~~~~~
```

```
11_sig_1434_BGLB000011       ~~~~~~~~~~~~~~~~~~~~~~~~~~~~~~~~~~~~~~~~~~~~~~~~~~
12_tig_1434_BGLB000019       ~LETQLANLSTDLID~~~~~MKRNIDKDE~~~~~~~~~~~~~~~~~~~~~
13_tig_2094_BGLB007076       ~~~~~~~~~~~~~~~~~~~~~~~~~~~~~~~~~~~~~~~~~~~~~~~~~~
14_tig_2094_BGLB000100       ~~~~~~~~~~~~~~~~~~~~~~~~~~~~~~~~~~~~~~~~~~~~~~~~~~
15_nsig_2162_BGLB000141      ~~~~~~~~~~~~~~~~~~~~~~~~~~~~~~~~~~~~~~~~~~~~~~~~~~
16_tig_2402_BGLB007591       ~~~~~~~~~~~~~~~~~~~~~~~~~~~~~~~~~~~~~~~~~~~~~~~~~~
17_tig_2702_BGLB000021       ~~~~~~~~~~~~~~~~~~~~~~~~~~~~~~~~~~~~~~~~~~~~~~~~~~
18_tig_2841_BGLB000096       ~NKGNQED~~~~~~~~~~~~~~~~~~~~~~~~~~~~~~~~~~~~~~~~~~
19_tig_3569_BGLB009245a      NINQLKED~~~~~LKSNKQNTQNITED~~~~VNTNRLNIMNINKD~LNTS
20_tig_3569_BGLB009245b      ~~~~~~~~~~~~~~~~~~~~~~~~~~~~~~~~~~~~VKIPTDTDVD~~~~
21_tig_3807_BGLB000074       ~~~~~~~~~~~~~~~~~~~~~~~~~~~~~~~~~~~~~~~~~~~~~~~~~~
22_tig_4104_BGLB000133       ~~~~~~~~~~~~~~~~~~~~~~~~~~~~~~~~~~~~~~~~~~~~~~~~~~
za_tig_4714                  TIKQIRVD~~~~~MDTNITSLKKTMD~~~~~TNTLDIQNIKVDSD~~~~~
zb_tig_15796                 ~~~~~~~~~~~~~~~~~~~~~~~~~~~~~~~~~~~~~~~~~~~~~~~~~~
x_tig_565_no-Cs              RCPLKVRCRGTMFINPYTTGGLVCGTSPHRLCNIE*~~~~~~~~~~~~~~
z_tig_6870_frameshifts       STKQDIVS~~~~~LKDNMNSTKQDIIS~~~~~LKDNMNSTKQDIVS~~~~

BgMFREP2_AY012700            ~~~~~~~~~~~~~~~~~~~~~~~~~~~~~~~~~~~~~~~~~~~~~~~~~~
FREP3.1_BB02BAC_AEO50745     ~~~~~~~~~~~~~~~~~~~~~~~~~~~~~~~~~~~~~~~~~~~~~~~~~~
FREP3.2_BB02BAC_AEO50746     ~~~~~~~~~~~~~~~~~~~~~~~~~~~~~~~~~~~~~~~~~~~~~~~~~~
FREP3.3_BB02BAC_AEO50747     ~LLSNLHNMNISSIWKVLSNFSTAVMDMKDDIDKGKTE~~~~~~~~~~~~
BgMFREP4 _AY012701           ~~~~~~~~~~~~~~~~~~~~~~~~~~~~~~~~~~~~~~~~~~~~~~~~~~
FREP7.1_AY028462             FQEIILANL~SATLEKVKNETE~~~~~~~~~~~~~~~~~~~~~~~~~~~~
FREP12.1_AF515464            TQINEKIKK~~~~~~~~~~~~~~~~~~~~~~~~~~~~~~~~~~~~~~~~~
FREP13_AF515468              ~~~~~~~~~~~~~~~~~~~~~~~~~~~~~~~~~~~~~~~~~~~~~~~~~~
FREP14_BB02BAC_AEO50744      ~~~~~~~~~~~~~~~~~~~~~~~~~~~~~~~~~~~~~~~~~~~~~~~~~~
FREP14_EF467292              ~~~~~~~~~~~~~~~~~~~~~~~~~~~~~~~~~~~~~~~~~~~~~~~~~~
1_tig_99_BGLB000116          TVFLNLSSSLTEVKQEVE~~~~~~~~~~~~~~~~~~~~~~~~~~~~~~~~
1_sig_99_BGLB000152          ~~~~~~~~~~~~~~~~~~~~~~~~~~~~~~~~~~~~~~~~~~~~~~~~~~
3_sig_480_BGLB000179         ~~~~~~~~~~~~~~~~~~~~~~~~~~~~~~~~~~~~~~~~~~~~~~~~~~
4_tig_565_BGLB000073         MNTSKQYLSKLETD~~~~~LETQLTNLSTALTQINEKIE~~~~~~~~~~~
5_tig_565_BGLB011626         QQSMRKLETDLETQLTNLSTTLTSIQKEI~~~~~~~~~~~~~~EKGQVFF
6_tig_990_BGLB014388         ~~~~~~~~~~~~~~~~~~~~~~~~~~~~~~~~~~~~~~~~~~~~~~~~~~
7_tig 1199_BGLB000140        ~ ~~~~~~~~~~~~~~~~~~~~~~~~~~~~~~~~~~~~~~~~~~~~~~~~~
8_tig_1199_BGLB004529        ~~~~~~~~~~~~~~~~~~~~~~~~~~~~~~~~~~~~~~~~~~~~~~~~~~
9_tig_1199_BGLB004530        ~~~~~~~~~~~~~~~~~~~~~~~~~~~~~~~~~~~~~~~~~~~~~~~~~~
10_tig_1199_BGLB000019       ~~~~~~~~~~~~~~~~~~~~~~~~~~~~~~~~~~~~~~~~~~~~~~~~~~
11_sig_1434_BGLB000011       ~~~~~~~~~~~~~~~~~~~~~~~~~~~~~~~~~~~~~~~~~~~~~~~~~~
12_tig_1434_BGLB000019       ~~~~~~~~~~~~~~~~~~~~~~~~~~~~~~~~~~~~~~~~~~~~~~~~~~
13_tig_2094_BGLB007076       ~~~~~~~~~~~~~~~~~~~~~~~~~~~~~~~~~~~~~~~~~~~~~~~~~~
14_tig_2094_BGLB000100       ~~~~~~~~~~~~~~~~~~~~~~~~~~~~~~~~~~~~~~~~~~~~~~~~~~
15_nsig_2162_BGLB000141      ~~~~~~~~~~~~~~~~~~~~~~~~~~~~~~~~~~~~~~~~~~~~~~~~~~
16_tig_2402_BGLB007591       ~~~~~~~~~~~~~~~~~~~~~~~~~~~~~~~~~~~~~~~~~~~~~~~~~~
17_tig_2702_BGLB000021       ~~~~~~~~~~~~~~~~~~~~~~~~~~~~~~~~~~~~~~~~~~~~~~~~~~
18_tig_2841_BGLB000096       ~~~~~~~~~~~~~~~~~~~~~~~~~~~~~~~~~~~~~~~~~~~~~~~~~~
19_tig_3569_BGLB009245a      QQSLSKLEADLETQLANLSNTLTSIKEQV~~~~~~~~~~~~~~E~~~~~~
20_tig_3569_BGLB009245b      ~~~~~~~~~~~~~~~~~~~~~~~~~~~~~~~~~~~~~~~~~~~~~~~~~~
21_tig_3807_BGLB000074       ~~~~~~~~~~~~~~~~~~~~~~~~~~~~~~~~~~~~~~~~~~~~~~~~~~
22_tig_4104_BGLB000133       ~~~~~~~~~~~~~~~~~~~~~~~~~~~~~~~~~~~~~~~~~~~~~~~~~~
za_tig_4714                  ~TNITRLKQDMDTNTGNIKIIKMDMDANITNLKDNLETNKQMINNFKENL
zb_tig_15796                 ~~~~~~~~~~~~~~~~~~~~~~~~~~~~~~~~~~~~~~~~~~~~~~~~~~
x_tig_565_no-Cs              ~~~~~~~~~~~~~~~~~~~~~~~~~~~~~~~~~~~~~~~~~~~~------
z_tig_6870_frameshifts       ~LKDNMNSTKQDIVS~~~~~LKDNMNSTEQHIVS~~~~~IKKDLDTTQAN

BgMFREP2_AY012700            ~~~~~~~~~~~~~~~~~~~~~~~~~~~~~~~~~~~~~~~~~~~~~~~~~~
FREP3.1_BB02BAC_AEO50745     ~~~~~~~~~~~~~~~~~~~~~~~~~~~~~~~~~~~~~~~~~~~~~~~~~~
FREP3.2_BB02BAC_AEO50746     ~~~~~~~~~~~~~~~~~~~~~~~~~~~~~~~~~~~~~~~~~~~~~~~~~~
FREP3.3_BB02BAC_AEO50747     ~~~~~~~~~~~~~~~~~~~~~~~~~~~~~~~~~~~~~~~~~~~~~~~~~~
BgMFREP4_AY012701            ~~~~~~~~~~~~~~~~~~~~~~~~~~~~~~~~~~~~~~~~~~~~~~~~~~
FREP7.1_AY028462             ~~~~~~~~~~~~~~~~~~~~~~~~~~~~~~~~~~~~~~~~~~~~~~~~~~
FREP12.1_AF515464            ~~~~~~~~~~~~~~~~~~~~~~~~~~~~~~~~~~~~~~~~~~~~~~~~~~
FREP13_AF515468              ~~~~~~~~~~~~~~~~~~~~~~~~~~~~~~~~~~~~~~~~~~~~~~~~~~
FREP14_BB02BAC_AEO50744      ~~~~~~~~~~~~~~~~~~~~~~~~~~~~~~~~~~~~~~~~~~~~~~~~~~
FREP14_EF467292              ~~~~~~~~~~~~~~~~~~~~~~~~~~~~~~~~~~~~~~~~~~~~~~~~~~
1_tig_99_BGLB000116          ~~~~~~~~~~~~~~~~~~~~~~~~~~~~~~~~~~~~~~~~~~~~~~~~~~
1_sig_99_BGLB000152          ~~~~~~~~~~~~~~~~~~~~~~~~~~~~~~~~~~~~~~~~~~~~~~~~~~
3_sig_480_BGLB000179         ~~~~~~~~~~~~~~~~~~~~~~~~~~~~~~~~~~~~~~~~~~~~~~~~~~
4_tig_565_BGLB000073         ~~~~~~~~~~~~~~~~~~~~~~~~~~~~~~~~~~~~~~~~~~~~~~~~~~
5_tig_565_BGLB011626         ISSSSMLE~~~~~~~~~~~~~~~~~~~~~~~~~~~~~~~~~~~~~~~~~~
6_tig_990_BGLB014388         ~~~~~~~~~~~~~~~~~~~~~~~~~~~~~~~~~~~~~~~~~~~~~~~~~~
7_tig 1199_BGLB000140        ~ ~~~~~~~~~~~~~~~~~~~~~~~~~~~~~~~~~~~~~~~~~~~~~~~~~
8_tig_1199_BGLB004529        ~~~~~~~~~~~~~~~~~~~~~~~~~~~~~~~~~~~~~~~~~~~~~~~~~~
9_tig_1199_BGLB004530        ~~~~~~~~~~~~~~~~~~~~~~~~~~~~~~~~~~~~~~~~~~~~~~~~~~
10_tig_1199_BGLB000019       ~~~~~~~~~~~~~~~~~~~~~~~~~~~~~~~~~~~~~~~~~~~~~~~~~~
11_sig_1434_BGLB000011       ~~~~~~~~~~~~~~~~~~~~~~~~~~~~~~~~~~~~~~~~~~~~~~~~~~
12_tig_1434_BGLB000019       ~~~~~~~~~~~~~~~~~~~~~~~~~~~~~~~~~~~~~~~~~~~~~~~~~~
13_tig_2094_BGLB007076       ~~~~~~~~~~~~~~~~~~~~~~~~~~~~~~~~~~~~~~~~~~~~~~~~~~
14_tig_2094_BGLB000100       ~~~~~~~~~~~~~~~~~~~~~~~~~~~~~~~~~~~~~~~~~~~~~~~~~~
15_nsig_2162_BGLB000141      ~~~~~~~~~~~~~~~~~~~~~~~~~~~~~~~~~~~~~~~~~~~~~~~~~~
16_tig_2402_BGLB007591       ~~~~~~~~~~~~~~~~~~~~~~~~~~~~~~~~~~~~~~~~~~~~~~~~~~
17_tig_2702_BGLB000021       ~~~~~~~~~~~~~~~~~~~~~~~~~~~~~~~~~~~~~~~~~~~~~~~~~~
18_tig_2841_BGLB000096       ~~~~~~~~~~~~~~~~~~~~~~~~~~~~~~~~~~~~~~~~~~~~~~~~~~
```

```
19_tig_3569_BGLB009245a      ~~~~~~~~~~~~~~~~~~~~~~~~~~~~~~~~~~~~~~~~~~~~~~~~~~
20_tig_3569_BGLB009245b      ~~~~~~~~~~~~~~~~~~~~~~~~~~~~~~~~~~~~~~~~~~~~~~~~~~
21_tig_3807_BGLB000074       ~~~~~~~~~~~~~~~~~~~~~~~~~~~~~~~~~~~~~~~~~~~~~~~~~~
22_tig_4104_BGLB000133       ~~~~~~~~~~~~~~~~~~~~~~~~~~~~~~~~~~~~~~~~~~~~~~~~~~
za_tig_4714                  RAMLSNYSSDLEELKIQILE~~~~~~~~~~~~~~~~~~~~~~~~~~~~~~
zb_tig_15796                 ~~~~~~~~~~~~~~~~~~~~~~~~~~~~~~~~~~~~~~~~~~~~~~~~~~
x_tig_565_no-Cs              --------------------------------------------------
z_tig_6870_frameshifts       MKSLNEDFYTNIQNITS~~~IKKDMNTYKENILRIETDLDSNKQNILSIR

BgMFREP2_AY012700            ~~~~~~~~~~~~~~~~~~~~~~~~~~~~~~~~~~~~~~~~~~~~~~~~~~
FREP3.1_BB02BAC_AEO50745     ~~~~~~~~~~~~~~~~~~~~~~~~~~~~~~~~~~~~~~~~~~~~~~~~~~
FREP3.2_BB02BAC_AEO50746     ~~~~~~~~~~~~~~~~~~~~~~~~~~~~~~~~~~~~~~~~~~~~~~~~~~
FREP3.3_BB02BAC_AEO50747     ~~~~~~~~~~~~~~~~~~~~~~~~~~~~~~~~~~~~~~~~~~~~~~~~~~
BgMFREP4_AY012701            ~~~~~~~~~~~~~~~~~~~~~~~~~~~~~~~~~~~~~~~~~~~~~~~~~~
FREP7.1_AY028462             ~~~~~~~~~~~~~~~~~~~~~~~~~~~~~~~~~~~~~~~~~~~~~~~~~~
FREP12.1_AF515464            ~~~~~~~~~~~~~~~~~~~~~~~~~~~~~~~~~~~~~~~~~~~~~~~~~~
FREP13_AF515468              ~~~~~~~~~~~~~~~~~~~~~~~~~~~~~~~~~~~~~~~~~~~~~~~~~~
FREP14_BB02BAC_AEO50744      ~~~~~~~~~~~~~~~~~~~~~~~~~~~~~~~~~~~~~~~~~~~~~~~~~~
FREP14_EF467292              ~~~~~~~~~~~~~~~~~~~~~~~~~~~~~~~~~~~~~~~~~~~~~~~~~~
1_tig_99_BGLB000116          ~~~~~~~~~~~~~~~~~~~~~~~~~~~~~~~~~~~~~~~~~~~~~~~~~~
1_sig_99_BGLB000152          ~~~~~~~~~~~~~~~~~~~~~~~~~~~~~~~~~~~~~~~~~~~~~~~~~~
3_sig_480_BGLB000179         ~~~~~~~~~~~~~~~~~~~~~~~~~~~~~~~~~~~~~~~~~~~~~~~~~~
4_tig_565_BGLB000073         ~~~~~~~~~~~~~~~~~~~~~~~~~~~~~~~~~~~~~~~~~~~~~~~~~~
5_tig_565_BGLB011626         ~~~~~~~~~~~~~~~~~~~~~~~~~~~~~~~~~~~~~~~~~~~~~~~~~~
6_tig_990_BGLB014388         ~~~~~~~~~~~~~~~~~~~~~~~~~~~~~~~~~~~~~~~~~~~~~~~~~~
7_tig_1199_BGLB000140        ~ ~~~~~~~~~~~~~~~~~~~~~~~~~~~~~~~~~~~~~~~~~~~~~~~~~~
8_tig_1199_BGLB004529        ~~~~~~~~~~~~~~~~~~~~~~~~~~~~~~~~~~~~~~~~~~~~~~~~~~
9_tig_1199_BGLB004530        ~~~~~~~~~~~~~~~~~~~~~~~~~~~~~~~~~~~~~~~~~~~~~~~~~~
10_tig_1199_BGLB000019       ~~~~~~~~~~~~~~~~~~~~~~~~~~~~~~~~~~~~~~~~~~~~~~~~~~
11_sig_1434_BGLB000011       ~~~~~~~~~~~~~~~~~~~~~~~~~~~~~~~~~~~~~~~~~~~~~~~~~~
12_tig_1434_BGLB000019       ~~~~~~~~~~~~~~~~~~~~~~~~~~~~~~~~~~~~~~~~~~~~~~~~~~
13_tig_2094_BGLB007076       ~~~~~~~~~~~~~~~~~~~~~~~~~~~~~~~~~~~~~~~~~~~~~~~~~~
14_tig_2094_BGLB000100       ~~~~~~~~~~~~~~~~~~~~~~~~~~~~~~~~~~~~~~~~~~~~~~~~~~
15_nsig_2162_BGLB000141      ~~~~~~~~~~~~~~~~~~~~~~~~~~~~~~~~~~~~~~~~~~~~~~~~~~
16_tig_2402_BGLB007591       ~~~~~~~~~~~~~~~~~~~~~~~~~~~~~~~~~~~~~~~~~~~~~~~~~~
17_tig_2702_BGLB000021       ~~~~~~~~~~~~~~~~~~~~~~~~~~~~~~~~~~~~~~~~~~~~~~~~~~
18_tig_2841_BGLB000096       ~~~~~~~~~~~~~~~~~~~~~~~~~~~~~~~~~~~~~~~~~~~~~~~~~~
19_tig_3569_BGLB009245a      ~~~~~~~~~~~~~~~~~~~~~~~~~~~~~~~~~~~~~~~~~~~~~~~~~~
20_tig_3569_BGLB009245b      ~~~~~~~~~~~~~~~~~~~~~~~~~~~~~~~~~~~~~~~~~~~~~~~~~~
21_tig_3807_BGLB000074       ~~~~~~~~~~~~~~~~~~~~~~~~~~~~~~~~~~~~~~~~~~~~~~~~~~
22_tig_4104_BGLB000133       ~~~~~~~~~~~~~~~~~~~~~~~~~~~~~~~~~~~~~~~~~~~~~~~~~~
za_tig_4714                  ~~~~~~~~~~~~~~~~~~~~~~~~~~~~~~~~~~~~~~~~~~~~~~~~~~
zb_tig_15796                 ~~~~~~~~~~~~~~~~~~~~~~~~~~~~~~~~~~~~~~~~~~~~~~~~~~
x_tig_565_no-Cs              --------------------------------------------------
z_tig_6870_frameshifts       SEADTNKQDILRIKEDLDTKQQNMLSSRENVDTNRHNMSIFQEMVVANLS

BgMFREP2_AY012700            ~~~~~~~~DGVDSIQISRASPTLPESCRD~~~~~VISSEDRVVVTLASGL
FREP3.1_BB02BAC_AEO50745     ~~~~~~~~~~~~~~~~~~QHLIPKIICRD~~~~~VNSTDERVVVTLTSGL
FREP3.2_BB02BAC_AEO50746     ~~~~~~~~~~~~~~~GSTLSYPPRKSCRD~~~~~VNSTDERVVVTLTSGL
FREP3.3_BB02BAC_AEO50747     ~~~~~~~~~~~IKQNKSSITKLHPRSCRD~~~~~VNSTDDRVVVTLASGL
BgMFREP4_AY012701            ~~~~~~~~~VNKNLQLSCECLAKPTSCRD~~~~~VISTEDRVVVTLASGL
FREP7.1_AY028462             ~~~~~~~~~~~~~~~~~~~~LLHPTSCRK~~~~~VIYKEDRAIVTLASGL
FREP12.1_AF515464            ~~~~~~~~~~~~~~~~~~~~~GLPSSCRE~~~~~INSFQERVIVTLTSGL
FREP13_AF515468              ~~~~~~~~~~~~~~~~~~~~~~QITSCRD~~~~~VTSKDDRVVVTLASGL
FREP14_BB02BAC_AEO50744      ~~~~~~~~~~~~NILISTTHKTPFTSCRD~~~~~VNSSDERVVVTLASEQ
FREP14_EF467292              ~~~~~~~~~~~~NILISTTHKTPFTSCRD~~~~~VNSSDERVVVTLASEQ
1_tig_99_BGLB000116          ~~~~~~~~~~~~~~~~~~KGYKHIASCQV~~~~~RSIDSRVIVL~LISGL
1_sig_99_BGLB000152          ~~~~~~~~~VNKNLQLSCECLAKPTSCRD~~~~~VISTEDRVVVTLASGL
3_sig_480_BGLB000179         ~~~~~~~~~~~~VGGYNVITNRQPVSCRD~~~~~VNSTEDRMVVTLASGL
4_tig_565_BGLB000073         ~~~~~~~~~~~~~~~~~~~~KGPPISCRD~~~~~ISSTQNRVIVKLLSGL
5_tig_565_BGLB011626         ~~~~~~~~~~~~~~TNKYTFLVPPISCRE~~~~~IISTHNRVIVKLLSGL
6_tig_990_BGLB014388         ~~~~~~~~~~~TIITKLKKSLILLASCRY~~~~~VQSSEPRVIVTLTSGL
7_tig_1199_BGLB000140        ~~~~~~~~~~~~~~~~~~~~~YQITSCRD~~~~~VTSKDDRVVVTLASGL
8_tig_1199_BGLB004529        ~~~~~~~~~~~~~~~~~~~~LLHPTSCRK~~~~~VIYEEDRAIVTLASGL
9_tig_1199_BGLB004530        ~~~~~~~~~~~~~~~~~~~~~~~IASCRE~~~~~VKTREERVVVTLASGL
10_tig_1199_BGLB000019       ~~~~~~~~~~~~~~~~~RLGFKQVLSCRD~~~~~VRSIADRLVVFLTSGL
11_sig_1434_BGLB000011       ~~~~~~~~~~~~~~~~~~~~~~~~~SCLN~~~~~VRSIKARVFVILDSGL
12_tig_1434_BGLB000019       ~~~~~~~~~~~~~VLKRQFGFLQHTSCRD~~~~~ISSIQDRVIVTLVSGL
13_tig_2094_BGLB007076       ~~~~~~~~~LNSLRQTVNNIQGPPISFRD~~~~~ISSTQNHVIVKLLSGL
14_tig_2094_BGLB000100       ~~~~~~~~~~~~~~~~~TIILRTSTSCLD~~~~~VQSFEARVIVTLSSGL
15_nsig_2162_BGLB000141      ~~~~QTRLSRRQLPDDCIPVVSNATGCDDPKLTVGYHILEIVPKDGLGPI
16_tig_2402_BGLB007591       ~~~~~~~~~~~~~~~~~~QNTLIPQSCRG~~~~~IIYKKERVEVTLASGL
17_tig_2702_BGLB000021       ~~~~~~~~~~~~~~~~~~~~KGPPISCRQ~~~~~INCTHNRVNVKLLSGL
18_tig_2841_BGLB000096       ~~~~~~~~~~~~~~~~~~QHLIPQIICRD~~~~~VNSTDERVVVTLTSGL
19_tig_3569_BGLB009245a      ~~~~~~~~~~~~~~~~~~KGNVSSISCRD~~~~~TNSTHNRIIVKLLSGL
20_tig_3569_BGLB009245b      ~~~~~~~~~~~~~~KIHQKGNTAPKSCRY~~~~~VHSSDRRVIVTLASGL
21_tig_3807_BGLB000074       ~~~~~~~~~~~~~~~~~SVNRISPKSCRD~~~~~VNSTYDRVVVTLASGL
22_tig_4104_BGLB000133       ~~~~~~~~~~~~~~~~~FQKTLIPESCRG~~~~~IIHKKERVVVTLASGL
za_tig_4714                  ~~~~~~~~~~~~~~~V~~NSNIIPKSCRN~~~~~VTYPEAQVIVTLASGL
zb_tig_15796                 ~~~~~~~~~~~~~~FQYIKKNIFITSCLD~~~~~VQSFESRAIVMLSSGL
x_tig_565_no-Cs              --------------------------------------------------
z_tig_6870_frameshifts       TELKEVQNQIHKVNKLLQFLPEPPTTCRV~~~~~SSPQA~RAIVTLSSGL
```

```
BgMFREP2_AY012700          KVMCDTKTDGGGWIIFQRRINGYVDFYRGWKEYRDGFGDYDIG~EFYLGN
FREP3.1_BB02BAC_AEO50745   KVMCDTKTDGGGWIIFQRRINGKVDFYRGWKEYRDGFGDYTIG~EFYLGN
FREP3.2_BB02BAC_AEO50746   KVMCDTKTDGGGWIIFQRRISGNVDFYRGWKEYRDGFGDYNIG~EFYLGN
FREP3.3_BB02BAC_AEO50747   KVMCDTKTDGGGWIIFQRRINGSVDFYRGWQEYRDGFGDYNIG~EFYLGN
BgMFREP4_AY012701          EVMCDTTTDGGGWTIFQRRFNGSIDFYRDWKEYRDGFGDYNIG~EFYLGN
FREP7.1_AY028462           KVMCDTKTDGGGWIIFQRRVNGSVDFYRGWQEYRDGFGDYNIG~EFYLGN
FREP12.1_AF515464          KVMCDTKTDGGGWIIFQRRINGKVDFYRGWKEYRDGFGDYDIG~EFYLGN
FREP13_AF515468            KVMCDTKTDGGGWIIFQRRINGKVDFYRNWQVYRDGFGDYDIG~EFYLGN
FREP14_BB02BAC_AEO50744    KVMCDTKTDGGGWIIIQRRIMGYVDFYRGWKEYRDGFGDYNIG~EFYLGN
FREP14_EF467292            KVMCDTKTDGGGWIIIQRRIMGYVDFYRGWKEYRDGFGDYNIG~EFYLGN
1_tig_99_BGLB000116        KVMCDTKTDGGGWIIFQRRINGKVDFYRGWKEYRDGFGDYNIG~EFYLGN
1_sig_99_BGLB000152        EVMCDTTTDGGGWTIFQRRFNGSIDFYRGWKEYRDGFGDYNIG~EFYLGN
3_sig_480_BGLB000179       KVMCDTKTDGGGWIIFQRRINGKVDFYRGWKEYRDGFGDYNIG~EFYLGN
4_tig_565_BGLB000073       KVMCDTKTDGGGWIIFQRRINGSVDFYRDWKEYRDGFGDYDIG~EFYLGN
5_tig_565_BGLB011626       KVMCDTKTDGGGWIIFQRRINGKIDFYRGWQVYRDGFGDYSIG~EFYLGN
6_tig_990_BGLB014388       KVMCDTMTNGGGWIIFQRRINGKVDFYRGWKEYRDGFGDYNIG~EFYLGN
7_tig 1199_BGLB000140      KVMCDTKTDGGGWIIFQRRINGKVDFYRNWQAYRDGFGDYDIG~EFYLGN
8_tig_1199_BGLB004529      KVMCDTKTDGGGWIIFQRRINGKVDFYRGWKEYREGFGDYNIG~EFYLGN
9_tig_1199_BGLB004530      KVMCDTKTDGGGWIIFQRRTYGIVDFYRSWQEYRDGFGDVSIG~EFYLGN
10_tig_1199_BGLB000019     KVMCDTKTDGGGWLIFQRRINGKVDFYRGWKEYRDGFGDYDIG~EFYLGN
11_sig_1434_BGLB000011     EVMCDTKTDGGGWIIFQRRINGSVDFYRGWKEYRDGFGDYNIG~EFYLGN
12_tig_1434_BGLB000019     QVMCDTKTDGGGWIIFQRRINGNVDFYRGWKEYRDGFGDYNIG~EFYLGN
13_tig_2094_BGLB007076     KVICDTKTDGGGWIVSQRRINGKVDFYRDWKEYRDGFGDFNIG~EFYLGN
14_tig_2094_BGLB000100     KVMCDTKTDGGGWIIIQRRINGKVDFYRGWKEYRDGFGDYNIG~EFYLGN
15_nsig_2162_BGLB000141    KVLCHVRIQGEGWIVFQKRFDGSVDFYRNFRDYEVGFGTVSPTTEYWLGL
16_tig_2402_BGLB007591     KVMCDTKTDGGGWIIFQRRINGKVDFYRGWQEYRDGFGDYNIG~EFYLGN
17_tig_2702_BGLB000021     KVMCDTKTDGGGWIIIQRRINGKVDFYRGWKEYRDGFGDYNIG~EFYLGN
18_tig_2841_BGLB000096     KVMCDTKTDGGGWIIFQRRINGEVDFYRGWKEYRDGFGDYAIG~EFYLGN
19_tig_3569_BGLB009245a    KVMCDTKTDGGGWIIFHRRINGKVYFYRGWQEYRDSFGDYNIG~EFYLGN
20_tig_3569_BGLB009245b    TVMCDTQTDGGGWIVFQRRVNGMLHFYRNWTEYRDGFFDYDIG~EFYLGN
21_tig_3807_BGLB000074     KVMCDTKTGGGGWIVIQRRVNGKVHFFRDWSDYRNGFGDYNIG~EFYLGN
22_tig_4104_BGLB000133     KVMCDTKTDGGGWIIFQRRINGKVDFYRGWKEYRDGFGDYNIG~EFYLGN
za_tig_4714                KVMCDTKTDGGGWIIFQRRINGKVDFYRGWKEYRDGFGDFNIG~EFYLGN
zb_tig_15796               KVMCDTKTDGGGWIIFQRRINGKVDFYRGWKEYRDGFGDYNIG~EFYLGN
x_tig_565_no-Cs            --------------------------------------------------
z_tig_6870_frameshifts     EVMCDTKTDGGGWILIQRRINGKVDFYRDWKEYRDGFGDYNIG~EFYLGN

BgMFREP2_AY012700          ENIFKLTSSKKYD~~LRIDLEFNNTKYFAFYTRFEIL~GEQDYYKLQIGG
FREP3.1_BB02BAC_AEO50745   EYISKLTSTRNFD~~LRIDFKYNHKTYFVEYSDFRIL~NETNNYQLKIGK
FREP3.2_BB02BAC_AEO50746   ENIYMLTSTGQYD~~LRIDLKYKNNAFFAQYSGFKIL~SEKEKYKLNIGV
FREP3.3_BB02BAC_AEO50747   ENIYMLTSTGQYD~~LRIDLKYKNNSFFAQYSSFKIL~SEKEKYKLNIGV
BgMFREP4_AY012701          ENIFNLTSSRKYE~~LRFDLEYENKKYFAHYSDFKLL~DENNKYKLIIGS
FREP7.1_AY028462           ENIFKLTSTGKYN~~LRIDLVLKNQPYYAQYSIFQIL~SEKNHYKINVGG
FREP12.1_AF515464          ENIFGLISTGQYD~~LRIDLEFNNTNYFAQYENFKVL~SETEKYKLQTGT
FREP13_AF515468            ENVYNLTTSGNFD~~LRIDLKFNYQSYFAQYTHFKLL~SEKEFYRLKFGD
FREP14_BB02BAC_AEO50744    ENIHQLTSKKPHE~~LRIDLEFDNENYFAQYSSFLLL~GEEDLYKLQIGD
FREP14_EF467292            ENIHQLTSKKPHE~~LRIDLEFDNENYFAQYSSFLLL~GEEDLYKLQIGD
1_tig_99_BGLB000116        EIIFNLTSTGKYD~~LRIDLEFNNTKYFAQYKDFKVL~SETEKYKLQIGN
1_sig_99_BGLB000152        ENIFNLTSSRKYE~~LRFDLEYENKKYFAHYSDFQLL~DENNKYKLIIGS
3_sig_480_BGLB000179       ENIFMLTSGRQYE~~LRIDMEFKTKKYFAKYSRFKVL~SEANNYQLKIES
4_tig_565_BGLB000073       ENIFNLTSTGQYD~~LRIDLEYNDKKYFAQYKDFKIL~SETEKYXIQIGD
5_tig_565_BGLB011626       ENIFNLTSTGQYD~~LRIDLEFNNTKYFAQYENFKIL~SETEKYKLKIGN
6_tig_990_BGLB014388       EHIFKLTSSGQYD~~LRIDLEFKNKPYFAQYKDFKVL~SETEKYKLQIGQ
7_tig 1199_BGLB000140      ENVYNLTSSRNFD~~LRIDLKFNYQNYFAQYTNFKLL~SEQEFYRLKLGD
8_tig_1199_BGLB004529      ENIFKLTSTGQYD~~LRIDLVLKNQAYYAQYSIFQIL~SEKDNYKINVGG
9_tig_1199_BGLB004530      EHIYNLTSSAKYD~~LRIDIKYNRNSYYAQYSDFQIL~SEKDKYKLKIGA
10_tig_1199_BGLB000019     ENIFNLTSTGKYD~~LRIDLKYND~FYYAQYSNFQIL~SENDKYKLKIEA
11_sig_1434_BGLB000011     ENIFKLTSTGQYD~~LRIDLEFNNTKYFAQYENFKVL~SETEKYKLKIGK
12_tig_1434_BGLB000019     ENIFKLTSRRQFD~~LRIDLEFENAKYVAQYKDFKVL~GETEKYKLQIGK
13_tig_2094_BGLB007076     ENIFKLTSTEEYD~~LRIDLEFNNTKYFAQYKNFKVL~SETEKYKLQIGD
14_tig_2094_BGLB000100     ENIFKLTSDGIYD~~LRIDLEYNNKTYFAQYENFKVL~SETEKYKLQIGD
15_nsig_2162_BGLB000141    ENLRRLLVDNGDENGLRVDMTERGTAFNFTRIYPIFRVGPGDGYTLIADG
16_tig_2402_BGLB007591     ENIFSLTSTGQYD~~LRIDLEFNNTKYFAQYKDFKIL~SETEKYKLQIGK
17_tig_2702_BGLB000021     ENIFTLTSTEQYD~~LRIDLEFNNAKYFAQYENFKVL~IETEKYKLQIGT
18_tig_2841_BGLB000096     EYISKLTSTRNFD~~LRIDFKFNHKTYFVEFSDFRIL~NETNNYQLKIGK
19_tig_3569_BGLB009245a    ENIFKLTSTGQYD~~LRIDLEFNNTKYFAQYENFKVL~SETEKYKLQIGK
20_tig_3569_BGLB009245b    ENISRLTSTGQHE~~LRIDLEFNKTTYFAHYNGFKVL~GESEKYKVMVGV
21_tig_3807_BGLB000074     ENIFKLTSCGQYD~~LRIDLKYNNQSYYAQYTDFQIS~SENDNYKLKIKS
22_tig_4104_BGLB000133     ENIFKLTSTGQYD~~LRIDLEFNSTKYFALYNNFKVL~SETEKYKLKIGT
za_tig_4714                EHIFKLTSAGEYD~~LRIDLEYNNKSYFAQYEDFKVL~SETEKYKLQIGD
zb_tig_15796               ENIFKLTSTGQYD~~LRIDIQFSKSPYFAQYENFKVL~SETEKYKLHIGN
x_tig_565_no-Cs            --------------------------------------------------
z_tig_6870_frameshifts     ENIFKLTSTGQYD~~LRIDLEYNNKTYFAQYENFKVL~SETEKYKLKIGX

BgMFREP2_AY012700          YSGNAGDALTNIHN~DKFFSTYDKDNDLGTSGSCAVTHK~GAWWYRD~CY
FREP3.1_BB02BAC_AEO50745   YKGNASDEFSYHNN~MQFSTFDRDNDVDKR~NCALQFT~GAWWYHS~CHY
FREP3.2_BB02BAC_AEO50746   YSGNAGDSFSIHNN~SFFTTFDRDNDENSS~NCAVDYT~GAWWYQSGCHS
FREP3.3_BB02BAC_AEO50747   YSGNAGDSFSRHNN~SFFTTFDRDNDENSS~NCAVVYT~GAWWYKRGCHH
BgMFREP4_AY012701          YSGNAGDSMRRHVN~KFFTTFDKDNDDSPNDNCAIIRR~GAWWYQN~CAD
FREP7.1_AY028462           YSGNAGNSLSYHND~MYFSTFDNDNDAYSGVNCAVKYH~GAWWYKT~CYE
FREP12.1_AF515464          YSGNAGDDLSPHNN~KFFSTFDRDNDISSSTNCAEYNS~GAWWYES~CHH
```

```
FREP13_AF515468           YSGNAGDSLSYHKD~MYFSTFDKDNNIDSR~NCATEYW~GAWWYRS~CHY
FREP14_BB02BAC_AEO50744   YSGNAGDSLAYQKN~MSFSTYDRDNDNASGENCAVSYS~GAWWYNA~CHM
FREP14_EF467292           YSGNAGDSLAYQKN~MSFSTYDRDNDNASGENCAVSYS~GAWWYNA~CHM
1_tig_99_BGLB000116       YLGNASNQLFYHNN~TFFTTYDSDNDESRNVNCAVDSS~GAWWYYW~CHD
1_sig_99_BGLB000152       YSGTAGNSMKRHVN~KFFTTFDKDNDESPNENCAIIRR~GAWWYQN~CAD
3_sig_480_BGLB000179      FSGNAGDSLTYHND~QFFSTFDRDNDRQNRSNCAVTYL~GAWWYNA~CLY
4_tig_565_BGLB000073      YSGNAGDDLLYHNN~MYFSTFDRDNDVHSDTNCADYSS~GAWWYAD~CHR
5_tig_565_BGLB011626      YSGNAGDNLSYHNN~MFFSTFDRDNDNSGT~NCAEYSS~GAWWYED~CHR
6_tig_990_BGLB014388      YLGDAGDGLKNQNN~MFFTTFDRDNDIWSIDNCAKFYL~GAWWHHG~CHL
7_tig 1199_BGLB000140     YSGNAGDSLFNHRD~LYFSTYDKDKNIDSR~NCATEYR~GAWWYRS~CHT
8_tig_1199_BGLB004529     YSGNAGDSLSYHND~MYFSTYDKMSG~~~~~NCAVEKH~GAWWYKN~CYD
9_tig_1199_BGLB004530     YSGNAGDSLSYHNN~MFFSTYDKDNDLYGS~NCATNYL~SAWWYNS~CHN
10_tig_1199_BGLB000019    YSGNAGDSLSYHND~AHFSTYDQDNDNTSI~NCASTVS~GAWWYKS~CHH
11_sig_1434_BGLB000011    YSGNAGDELSYHND~EFFSTFDRDNDVHGDLNCAGFSS~GAWWYQS~CFR
12_tig_1434_BGLB000019    YSGNASDGLNVHNN~MFFSTFDRDNDVDSGRNCAERCS~GAWWYKG~CLQ
13_tig_2094_BGLB007076    YSGNAGDDLSPHNN~MFFTTFDRDNDVASDLNCAEQCS~GAWWYKS~CHD
14_tig_2094_BGLB000100    YSGNAVDSLINHKN~MFFTTFDRDNDRDRNKNCAQHYS~GAWWYNN~CHP
15_nsig_2162_BGLB000141   YS~DGGKGLSENSG~AKFSTYDNDHSLG~~~~CPSSLRLAGWWFHEGCGF
16_tig_2402_BGLB007591    YSGNAGDSLSLHNN~KFFTTFDRDNDVESDLNCAEDSS~GAWWYRS~CHS
17_tig_2702_BGLB000021    YSGNAGDDLSYHNN~MYFSTFDRDNDVHSGTNCAEHSS~GAWWFRE~CQY
18_tig_2841_BGLB000096    YKGNTSDDFSYHNN~MKFSSFDRDNDVDTR~NCALQYT~GAWWYNS~CHF
19_tig_3569_BGLB009245a   YSGNATEDLSRHNN~MFFTTFDSDNDIHSSGNCAEYSS~GAWWYAN~CHD
20_tig_3569_BGLB009245b   YSGNATDSFKISNN~MSFTTFDRDNDNIAFEQCARYNK~GAWWYDK~CVE
21_tig_3807_BGLB000074    YSGNASDSLSCHNG~GPFSTVDNDNDGSSA~NYAAVYI~GAWWYHPGCYH
22_tig_4104_BGLB000133    YSGNAGDELSVHNN~FYFSTFDRDNDVHSDVNCADYSS~GAWWYES~CNN
za_tig_4714               YSGNADDSLRRHNN~MFFSTFDRDNDIESF~NCAVESS~GAWWYYH~CST
zb_tig_15796              YSGNARDSLSYNNN~MFFSTFDRDNDKSSL~NCAQHYS~GAWWYNA~CHS
x_tig_565_no-Cs           --------------------------------------------------
z_tig_6870_frameshifts    YSGNATDSLSDHXN~MFFSTFDKDNDKKSG~NCAEMYS~GAWWYNA~CHQ

BgMFREP2_AY012700         DSNLNGKWGSDRVNWSKLTGITKS~~~~~VTFTEMKIREIELN*---------
FREP3.1_BB02BAC_AEO50745  SNLNGQWGSKEYSKGANWESITGYEAS~VSFVEMKIRER*-------------
FREP3.2_BB02BAC_AEO50746  CNLNGKWGSTDYGKGVNWYTLSTFYSS~LSFTEMKIREI*-------------
FREP3.3_BB02BAC_AEO50747  SNLNGKWGSSDHGKGVNWHVVSNFDSS~VSFTEIKIREI*-------------
BgMFREP4_AY012701         VNLNGNWGRGEPDGVFWDNITVWES~~~VSFSEIKIREIDKEKNKS*------
FREP7.1_AY028462          SNLNGKWGSSERDKDLNWNTLLQTAHVGVSFTEMKIRERE*------------
FREP12.1_AF515464         SNLNGQWGRTSN~KGMNWFKLTRGSNS~VSFTEMKIREREKNYFH*-------
FREP13_AF515468           SHLNGVWGGKDP~KGLIWDKVTNYEAS~VSFTEMKIREKS*------------
FREP14_BB02BAC_AEO50744   SNLNGDWGNDAYGKGVNWDGVTGLHDS~VVFSEMKLRELD*------------
FREP14_EF467292           SNLNGDWGNDAYGKGVNWDGVTGLHDS~VVFSEMKLRELD*------------
1_tig_99_BGLB000116       SNLNGKWGSKLYKQGMNWYGVTEDSNS~VSFTEMKIRERE*------------
1_sig_99_BGLB000152       VNLNGNWGRGEPDGAFWDNITVWES~~~VSFSEIKIREID*------------
3_sig_480_BGLB000179      ANLNGKWGSREYGKGLIWLGFTSPTES~VTFSEMKMRETSRQ*----------
4_tig_565_BGLB000073      SNLNGKWGSTLDHKGMNWAHLTGWIKS~VSFSEMKIREREKKTQFKSQLRSK*
5_tig_565_BGLB011626      SNLNGQWARTSYPKGMIWYQLTGSSNC~VSLSEMKIREREKNYFH*-------
6_tig_990_BGLB014388      SNLNGKWGSKSYGEGLNWADLTGYHDG~VSFSEMKIRERT*------------
7_tig 1199_BGLB000140     SHLNGVWGGKDS~KGLIWDKVTNYEAS~VSFTEMKIREKR*------------
8_tig_1199_BGLB004529     SNLNGQWGSSERGKDVNWNTLTETARVGVSFTDMKIRERE*------------
9_tig_1199_BGLB004530     SNLNGQWGSKVNSNGVIWNTLTKGESA~~SFTEIKIREK*-------------
10_tig_1199_BGLB000019    VNLNGRWRSKESGKSVIWRALTGEHES~VLYSEMKIREKD*------------
11_sig_1434_BGLB000011    SNLNGLWGRTSFHKGMNWLHLTGQSNS~VSFSEMKIREREKKLF*--------
12_tig_1434_BGLB000019    SNLNGKWGSTLYDRGLNWRDVTGWTKS~VSFSEMKIRETE*------------
13_tig_2094_BGLB007076    SNLNGQWGRNSYKGMNWEQLTRY~SNS~VSFSEMKIREREKNYFN*-------
14_tig_2094_BGLB000100    FTLNGKWGSKAGGYGLHWDGLTGYYNS~VSFSEMKLRERK*------------
15_nsig_2162_BGLB000141   VNLNGLWGLASGEASMFWYEIYNHPTHSMQATEMKFRPFPSPVRV*-------
16_tig_2402_BGLB007591    SNLNGPWGSTSYSKGMIWYKLTGGSKS~VSFSEMKIRERK*------------
17_tig_2702_BGLB000021    SHLNGQWGSTSHEKGMNWYKLTRHSNS~VSFSEMKIREREKNYFK*-------
18_tig_2841_BGLB000096    SNLNGQWGSKEYSKGANWESITGYGAS~VSFVEMKIRER*-------------
19_tig_3569_BGLB009245a   SNLNGQWGRTSYSKGMNWYELTGYSNS~VSFSEMKIRER*-------------
20_tig_3569_BGLB009245b   SNLNGLWNKTSYEGMNWKYLTGLSL~S~VTFSEMKIRERLKPIY*--------
21_tig_3807_BGLB000074    SNLNGKWGTTEYAKGLQWYSLTSYSTT~VSFTEMKIREIETDDYLTLLNK*--
22_tig_4104_BGLB000133    SNLNGQWGSTVYDQGMNWPHLTDYTNS~VSFSEMKIRERK*------------
za_tig_4714               SNLNGKWENDTYARRLHWDSLPGFHR~~ISFSEMKIRERK*------------
zb_tig_15796              YTLNGKWGGRSGGKGLNWSGLTDYDTS~VTFSEMKLRKRK*------------
x_tig_565_no-Cs           -----------------------------------------------------
z_tig_6870_frameshifts    SNLNGKWGSTSPSVGLNWYLLTGYSSS~VSFSEMKIKERE*------------
```

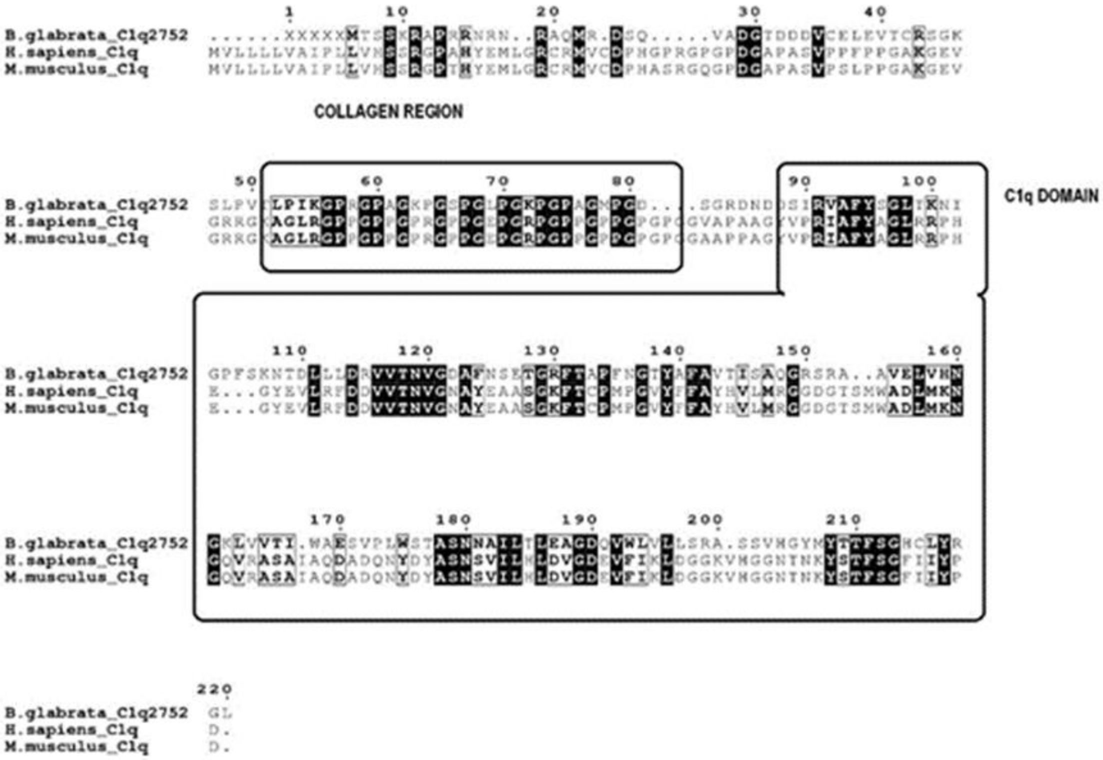

Supplementary Figure 28. *B. glabrata* C1q-1 amino acid alignment.
B. glabrata C1-1 sequence aligned to *H. sapiens* complement C1q (GenBank Accession X03084) and *M. musculus* C1q (GenBank Accession AAA37335). The conserved collagen region and C1q domain Boxed regions are boxed. Amino acid residues in dark boxes are conserved in the three sequences, while the amino acid residues in clear boxes are conserved in two out of the three sequences.

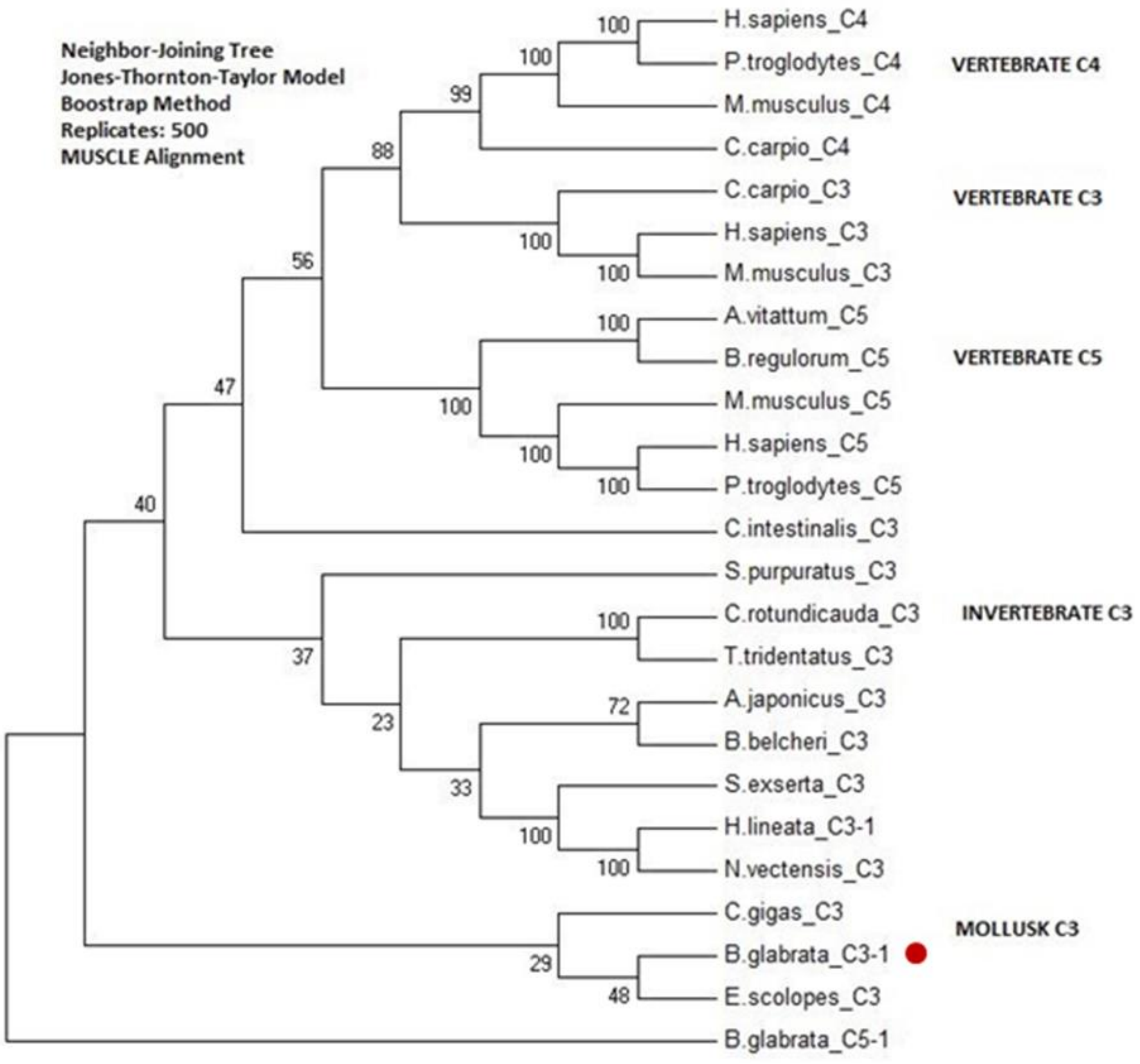

Supplementary Figure 29. Phylogenetic tree Complement C3 sequences.
Sequences (gaps removed) were aligned with MUSCLE to construct a phylogenetic tree using the Neighbor-joining Tree method with the James-Thornton-Taylor model, and the bootstrap method via 500 replicates. The sequences used in the construction of this phylogenetic tree were as follows: *Biomphalaria glabrata* C3-1, C3-5 (*B.glabrata*_C3-1, *B.glabrata*_C5-1), *Strongylocentrotus purpuratus* C3 (S.purpuratus_C3/AAC14396), *Swiftia exserta* C3 (S.exserta_C3/AAN86548), *Venerupis decussatus* C3 (V.decussatus_C3/ACN37845), *Euprymna scolopes* C3 (E.scolopes C3/ACF04700), *Carcinoscorpius rotundicauda* C3 (C.rotundicauda C3/AAQ08323), *Tachypleus tridentatus* C3 (T.tridentatus_C3,BAH02276), *Apostichopus japonicus* C3 (A.japonicus_ C3/ADN97000), *Branchiostoma belcheri* C3 (B.belcheri_C3/BAB47146), *Ciona intestinalis* C3 (C.intestinalis_C3/NP_001027684), *Nematostella vectensis* C3 (N.vectensis_C3, BAH22724), *Homo sapiens* C3, C4, C5 (H.sapiens_C3/P01024, H.sapiens_C4/P0C0L5, H.sapiens_C5/AAA51925), *Mus musculus* C3, C4, C5 (M.musculus_C3/AAH43338, M.musculus_C4/P01029, M.musculus_C5/P06684), *Cyprinus carpio* C3-H1 (C.carpio_C3/BAA36618), *Pan troglodytes* C3, C5 (P.troglodytes_C3/XP_003311220, P.troglodytes_C5/XP_520228), *Lepisosteus oculatus* C4 (L.oculatus_C4/ XP_006628287), *Cyprinus carpio* C4 (C.carpio_C4/ BAB03284), *Apaloderma vittatum C5* (A.vitattum_C5/ KFP85189), *Balearica regulorum gibbericeps* C5 (B.regulorum_C5/ KFO11286).

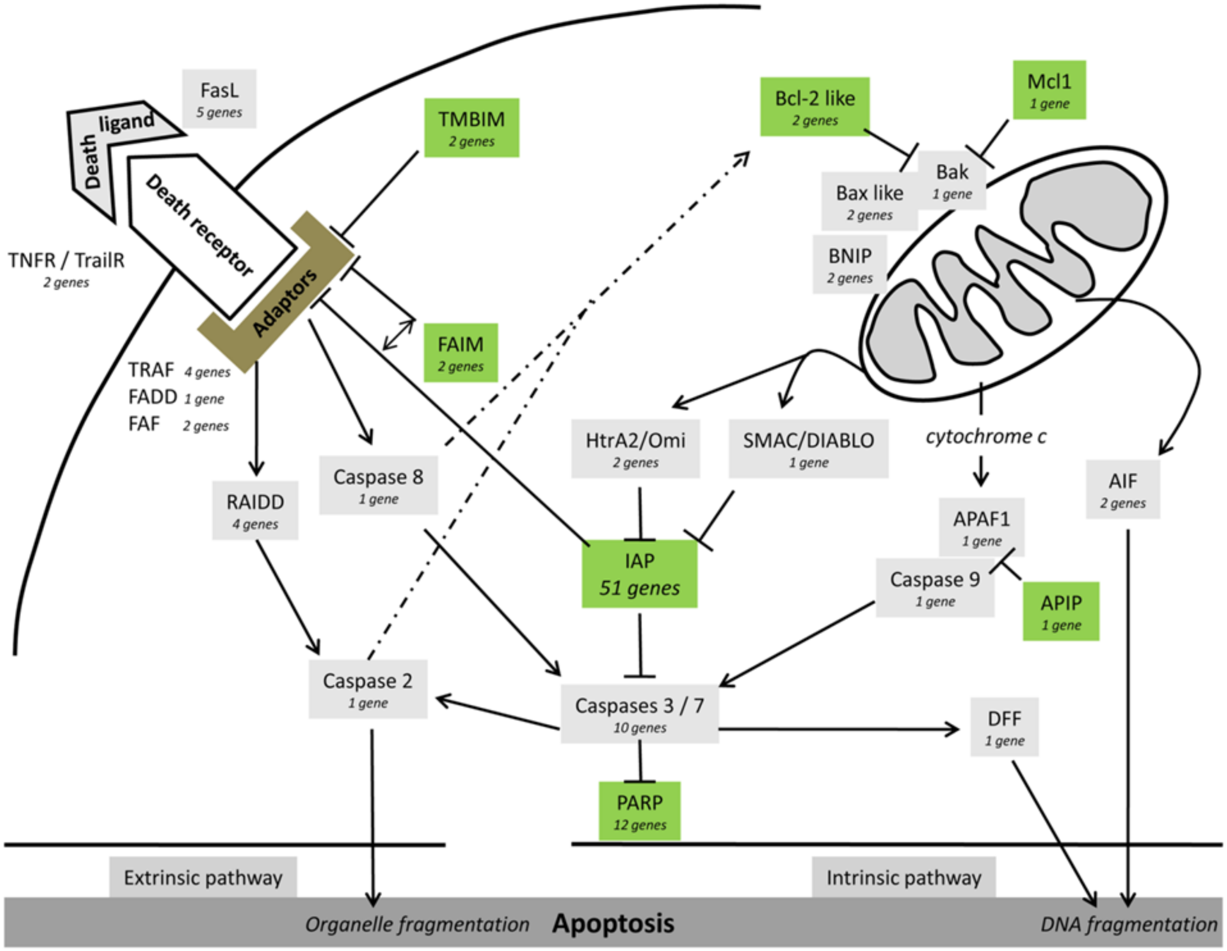

Supplementary Figure 30. Apoptotic signaling pathways in *Biomphalaria*. The abbreviations used in this schematic representation are as follows: FADD (Fas-Associated protein with Death Domain), TNFR (Tumor Necrosis Factor Receptor), TRAF (TNF Receptor-Associated Factor), FAF (Fas-Associated Factor), FasL (Fas Ligand), TrailR (TNF-Related Apoptosis-Inducing Ligand Receptor), TMBIM (Transmembrane Bax Inhibitor-1 Motif containing), FAIM (Fas Apoptotic Inhibitory Molecule), RAIDD (RIP Adaptor with a Death Domain), BNIP (BCL2/adenovirus E1B 19 kDa protein-interacting protein), IAP (Inhibitor of Apoptosis), APIP (Apaf-1 Interacting Protein), DFF (DNA Fragmentation Factor), PARP (Poly (ADPribose) Polymerase), SMAC/DIABLO (Second Mitochondria-derived Activator of Caspases), APAF1 (Apoptotic Peptidase Activating Factor 1), AIF (Apoptosis Inducing Factor), Mcl1(induced Myeloid leukemia cell differentiation protein), Bax (Bcl-2–associated x protein), Bak (Bcl-2 homologous antagonist/killer), Bcl (B-cell lymphoma 2).

Proteins involved in anti- or pro-apoptotic signaling pathways are shown in green or grey respectively.

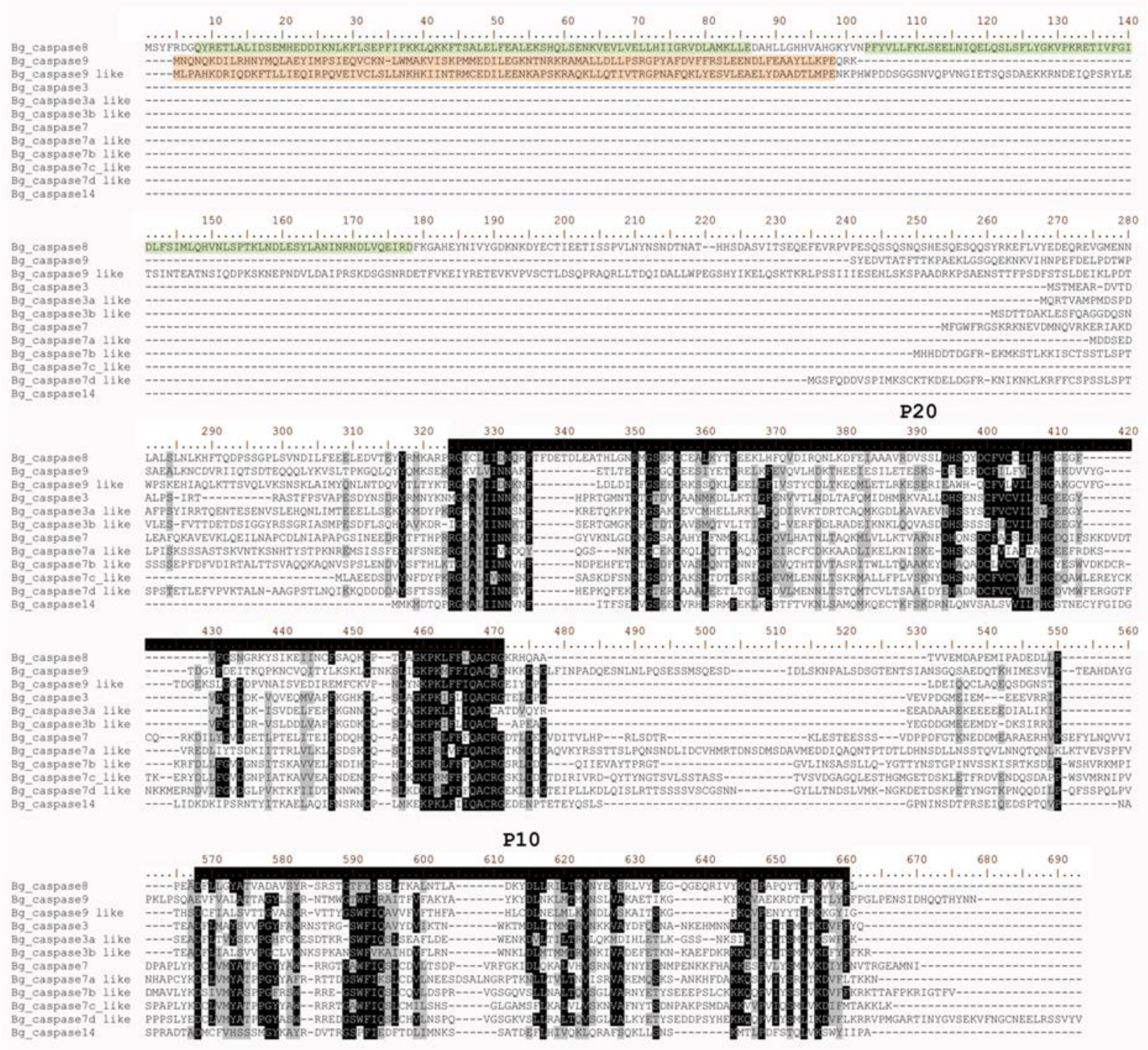

Supplementary Figure 31. Alignment of full length caspase proteins from *B. glabrata*. Identical and similar residues in this multiple alignment (ClustalW) are shown in black and grey background, respectively. CARD (Caspase Activation and Recruitment Domain) and DED (Death Effector Domain) are highlighted in orange or in green. The p20 domain and p10 domain predicted with PROSITE are highlighted by lines below the alignment. The conserved caspase family cystein active site motif QACRG was found in most of the caspase sequences from *B. glabrata*. To classify caspases containing a short prodomain located before the p20 subunit, a PSI-BLAST was performed with amino acid residues positioned between the p20 and p10 domains. Whole genome and transcriptome screening by use of the Interproscan database revealed 15 genes encoding proteins associated with a caspase domain (Interproscan number : IPR011600). Three of these are partial, the corresponding scaffolds did not include ORFs beginning with a start codon.

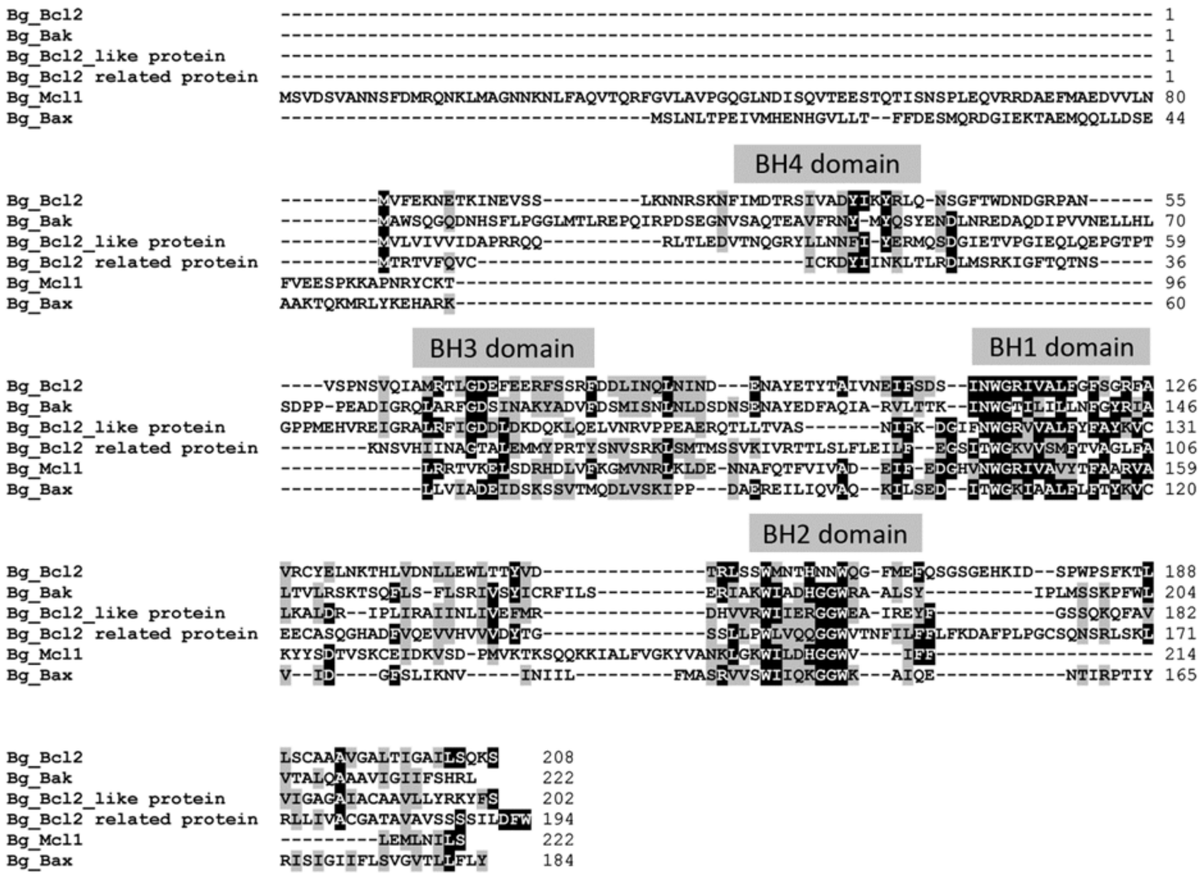

Supplementary Figure 32. Bcl2-family members in *B.glabrata*. Similar or identical residues in at least half of the compared sequences are shaded respectively in grey or in black. The conserved BH1, BH2, BH3 and BH4 domains are indicated in brown. Six core Bcl-2 family proteins were characterized. Two of them harbour three BH domains (BH1, BH2 and BH3 domain), Bg_Mcl1 and Bg_Bax, anti- and pro-apoptotic factors respectively. These others related members share the four BH domains found commonly in anti-apoptotic Bcl2 family proteins. BLASTP analysis of Bg_Bak and Bg_Bcl2 related protein revealed a high similarity with pro-apoptotic factor from two marine molluscs (Accession numbers EKC19322, *Crassostrea gigas* and *Aplysia californica*, XP_005094538, respectively)

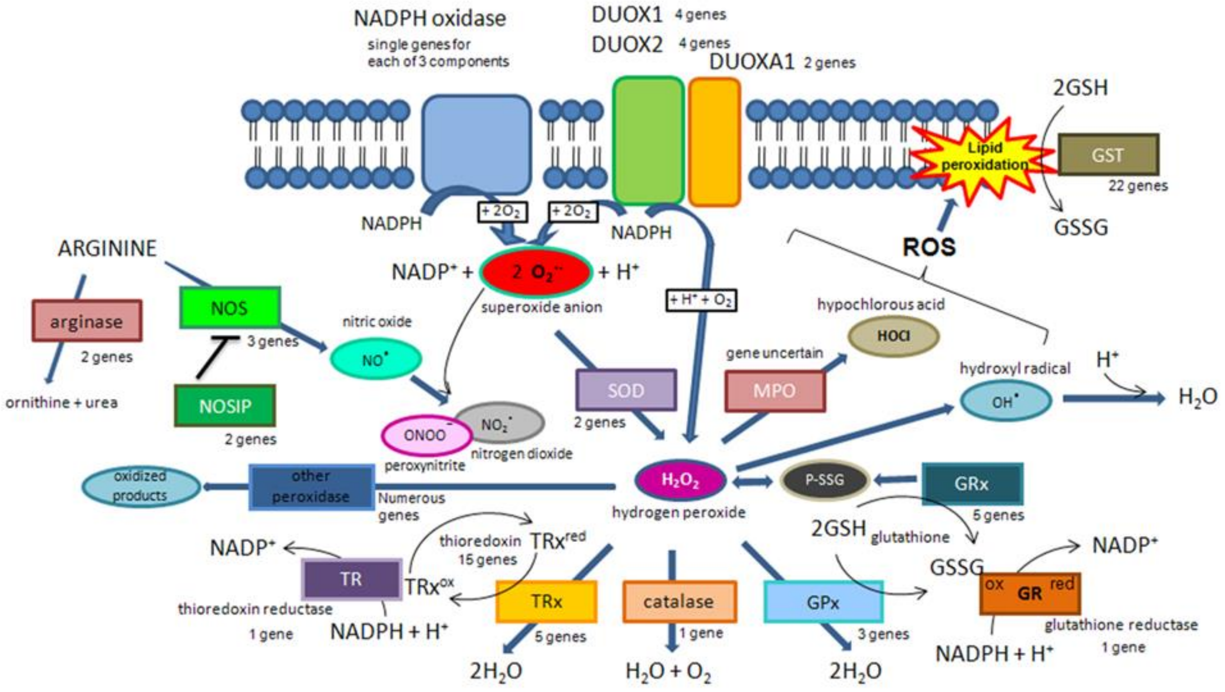

Supplementary Figure 33. Production and metabolism of reactive oxygen species and nitric oxide. The figure shows the network of interactive *B. glabrata* genes that support the capacity to generate and metabolize reactive oxygen species (ROS) and nitric oxide (NO). See Supplementary Note 15 and Supplementary Data 20 for details.

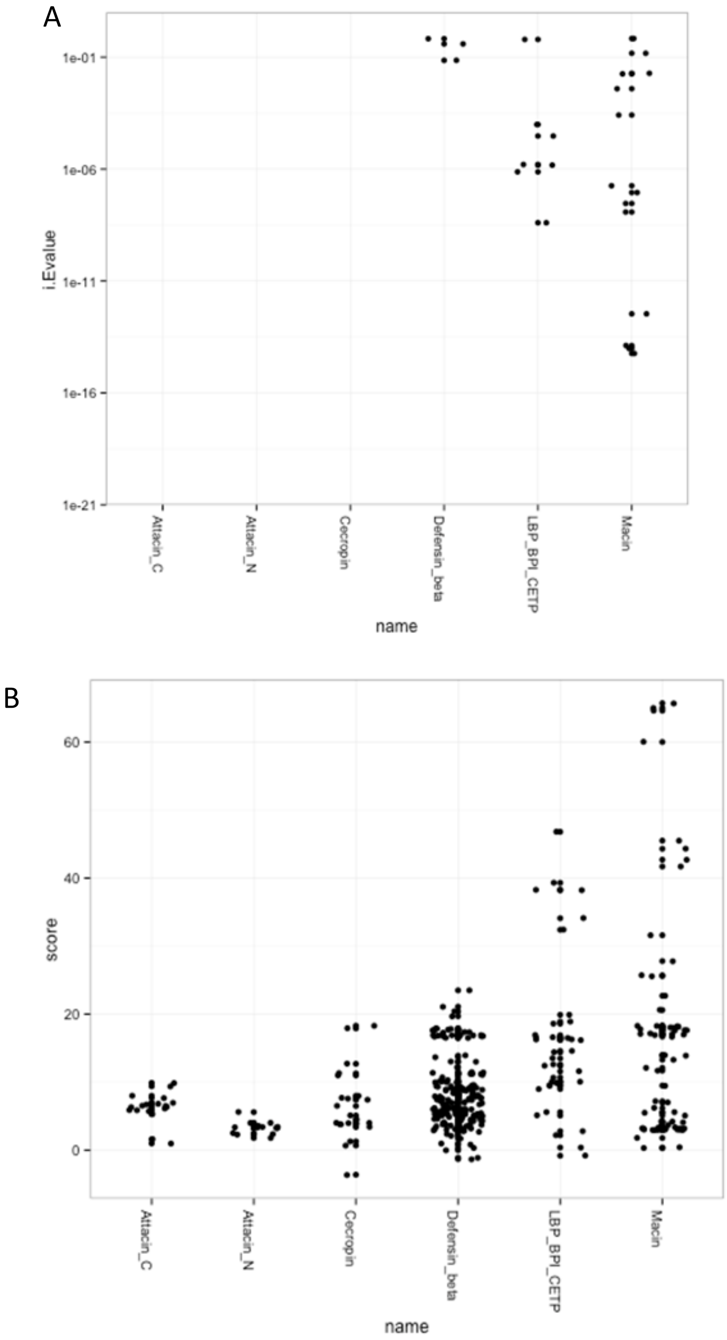

Supplementary Figure 34. ***B. glabrata*** **assembly lacks common AMPs. Additional tests were performed to investigate the apparent absence of common categories of antimicrobial peptides (AMPs) such as Attacins, Cecropins and Defensins, as indicated by BLAST searches of the** ***B. glabrata*** **assembly. A.** Independent E-values for available antimicrobial profile hidden Markov models obtained using HMMer3. **B.** Match scores for available antimicrobial profile hidden Markov models obtained using HMMer3. The results of these analyses support the findings that were obtained with alignment-based BLAST searches that genes encoding cecropins, attacins and defensins are not present in the *B. glabrata* genome assembly.

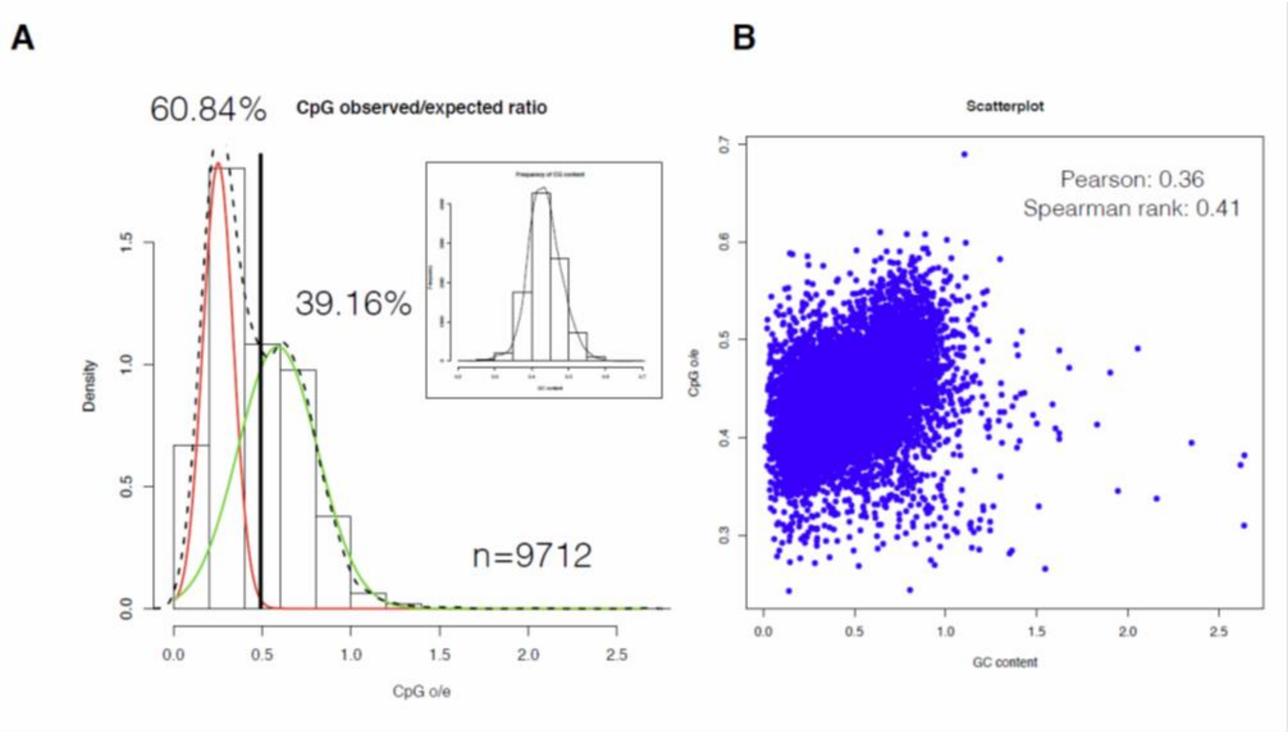

Supplemental Figure 35: **CpG observed/expected (o/e) ratio in coding regions.** (A) Frequency distribution of predicted coding regions of 9,712 genes. Frequency distribution shows bimodality with an estimated 61% of genes showing CpG o/e ratio <0.5 (red, presumably high methylation) and 39% that are probably not methylated (green, CpG o/e ≥0.5). No such bimodality was evident for the GC content (insert). (B) CpG o/e ratio and GC content of genes are statistically not correlated.

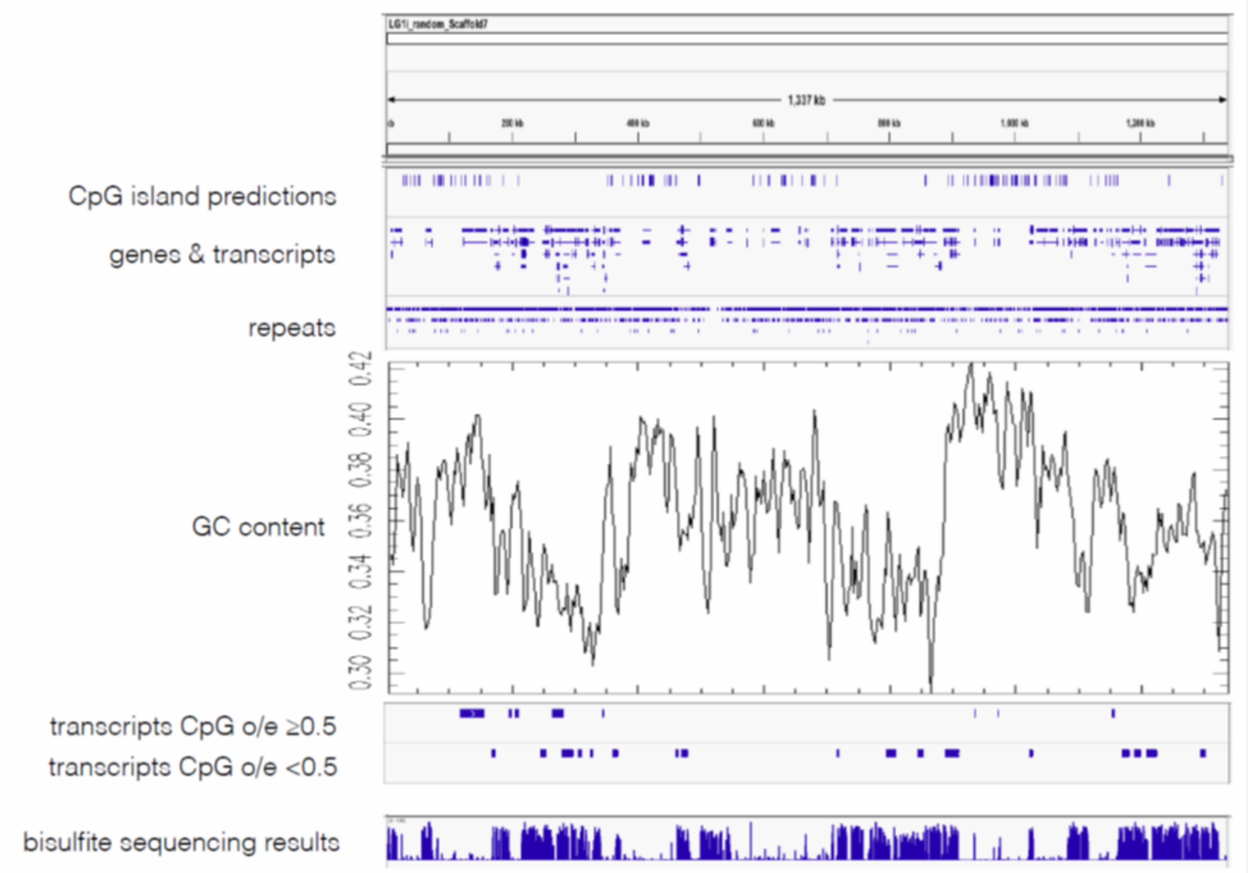

Supplemental Figure 36: **LG1i_random_Scaffold7, sequence content and CpG composition.** This schematic representation of one of the largest scaffolds depicts (Top panel) position in bp on top, CpG islands, predicted genes and predicted repeats superposed on GC content and location of transcripts with CpG observed/expected ratio <0.5 (presumably high methylation) and ≥0.5 (probably not methylated). Bottom panel: histogram of BSMAP results for bisulfite sequencing (BS-Seq), y-axis indicates degree of methylation (0 to maximum 1).

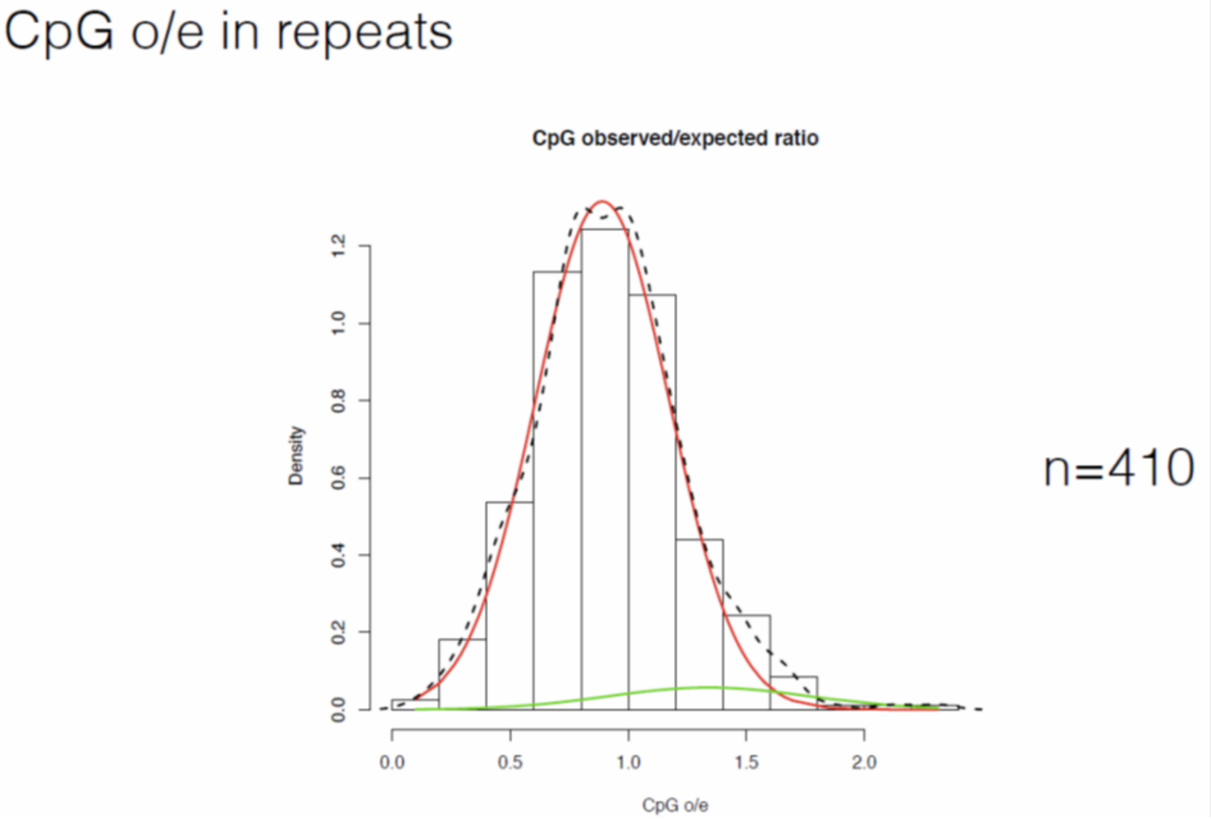

Supplemental Figure 37: **CpG observed/expected (o/e) ratio in repetitive sequences**. Frequency distribution of 410 predicted repeat consensus sequences. Frequency distribution shows unimodality with a mean value close to 1 suggesting that repeats are in general not methylated.

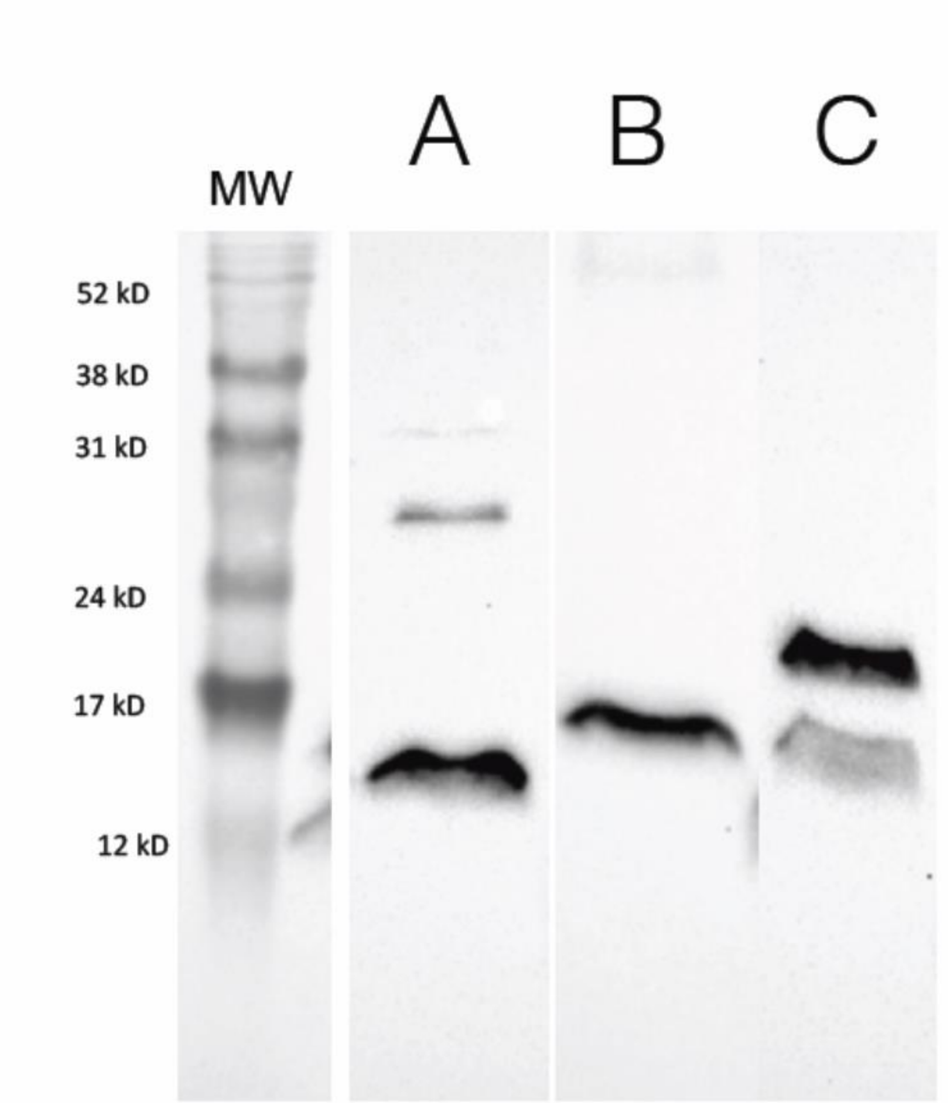

Supplemental Figure 38: **Western Blots show histone modifications**. Western blots were performed as previously described (Azzi *et al*., 2009). Tissue material was taken from the headfoot region of *B. glabrata* snails derived from Guadeloupe and maintained at the University of Perpignon. Western blotting confirmed the presence of histones H4 and H3 and their most prominent modifications, (A) H4K20me3, (B) H3K27me3, (C) H3K9ac. me=methylation, ac = acetylation.

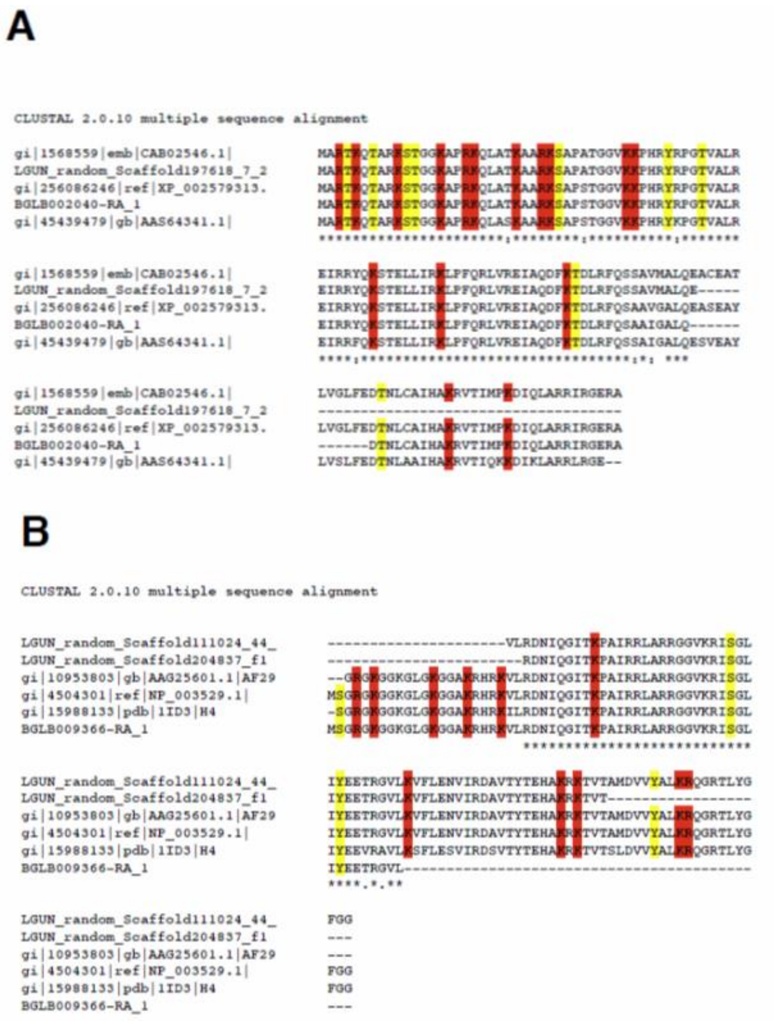

Supplemental Figure 39: **ClustalW alignment of amino-acid sequences of histones H3 and H4.** The ClustalW alignments show canonical acetylation/methylation sites in red and phosphorylation sites in yellow.

(A) H3: gi|45439479|gb|AAS64341.1| histone H3 [*Saccharomyces cerevisiae*], gi|256086246|ref|XP_002579313.1| histone H3 [*Schistosoma mansoni*], gi|1568559|emb|CAB02546.1| histone H3 [*Homo sapiens*], BGLB002040 and LGUN_random_Scaffold197618:7:291:1 [*B. glabrata*].

(B) H4: gi|15988133|pdb|1ID3|H4 [*Saccharomyces cerevisiae*], gi|10953803|gb|AAG25601.1|AF297469_1 histone H4 [*Schistosoma mansoni*], gi|4504301|ref|NP_003529.1| histone H4 [*Homo sapiens*], BGLB009366 and LGUN_random_Scaffold111024:44:289:-1, LGUN_random_Scaffold204837:1)[*B. glabrata*]

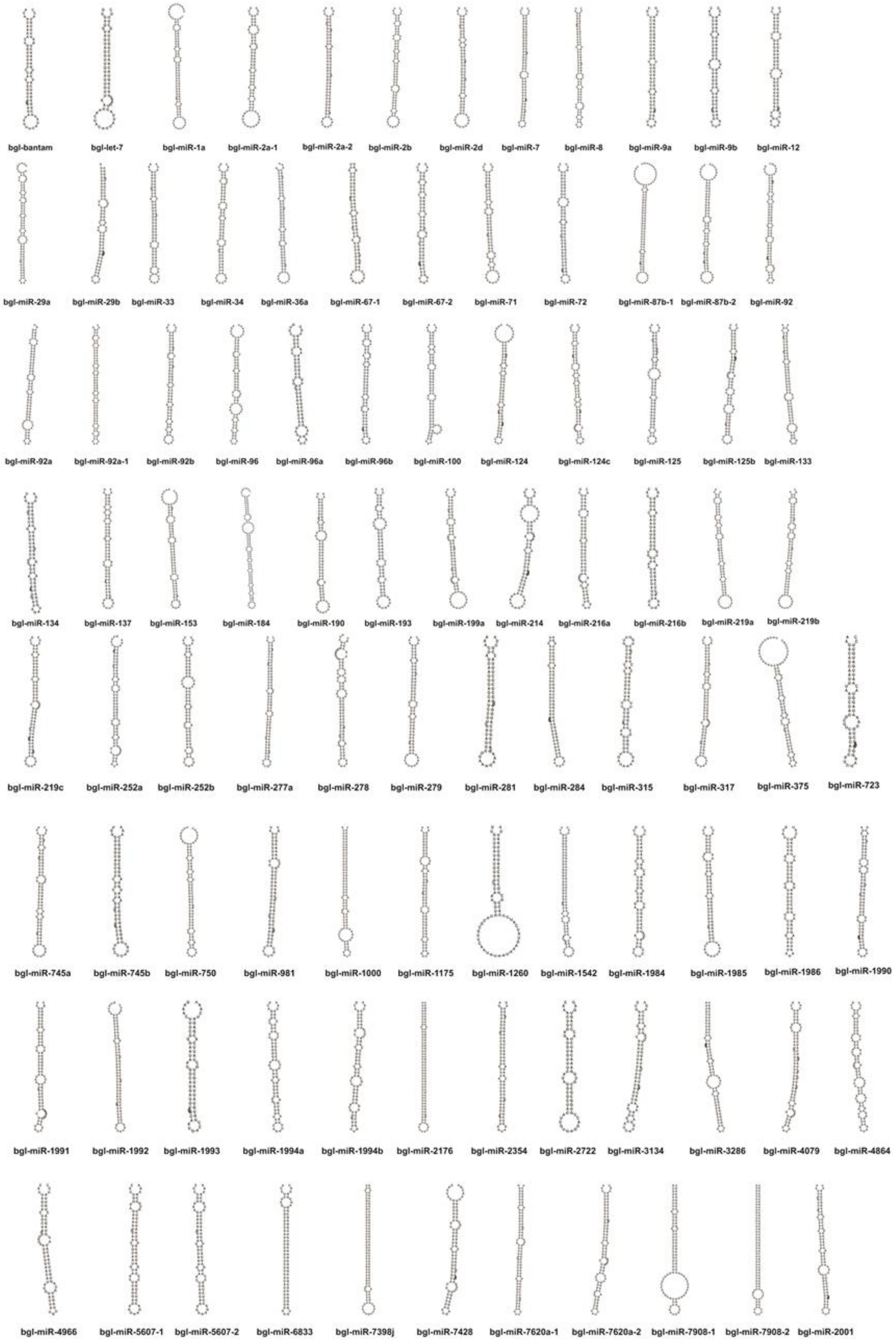

Supplementary figure 40: Secondary structures of *B. glabrata* pre-miRNA sequences

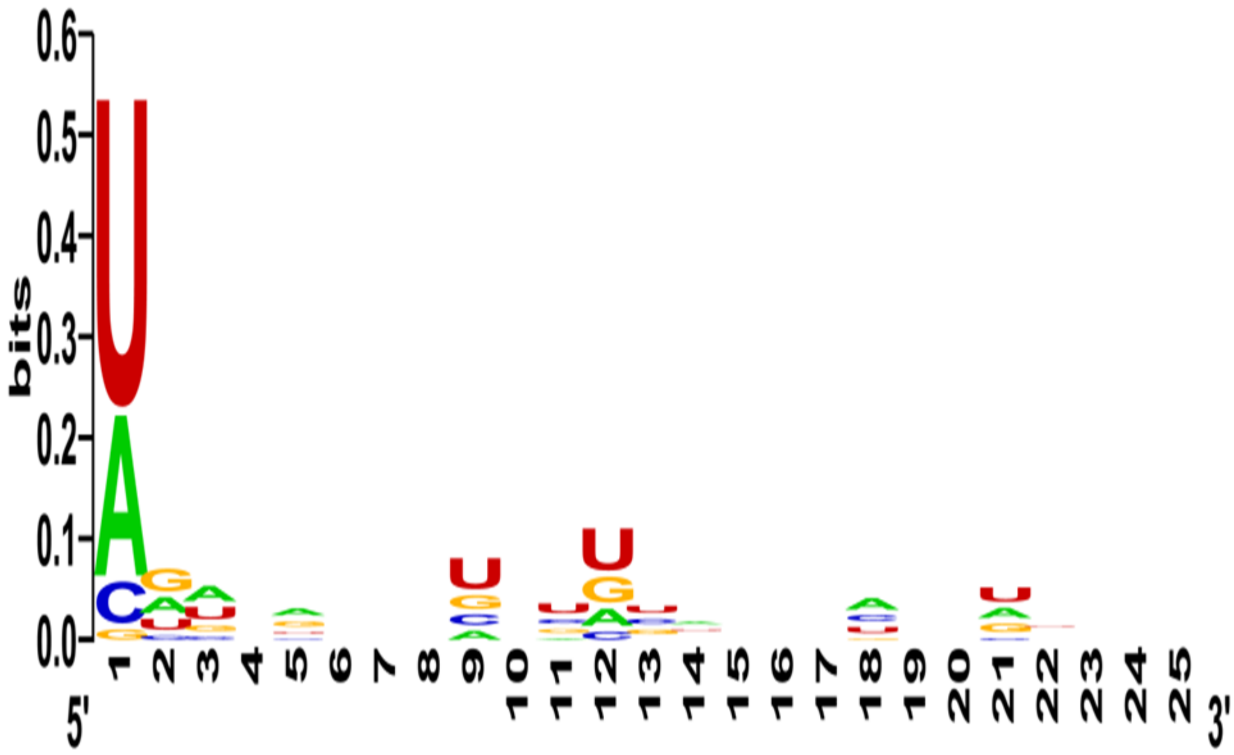

Supplementary figure 41: Weblogo of *B. glabrata* mature miRNAs. This sequence logo indicates relative frequencies of nucleotides at several positions along the length of sequence, evident from the aligned *B. glabrata* mature miRNAs.

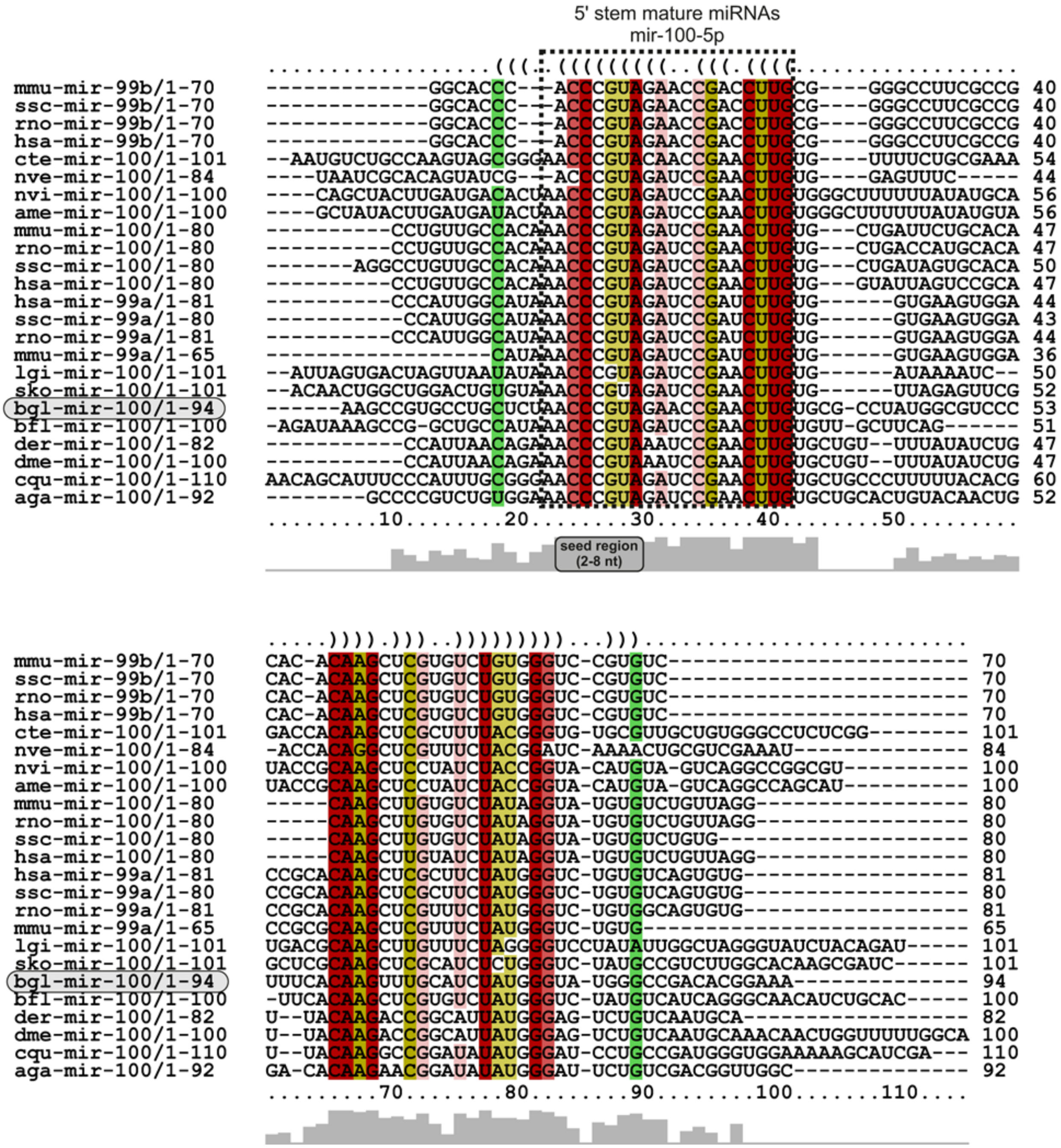

Supplementary figure 42: Alignment of bgl-mir-100 with animal homologs. Brackets and colors identify matching residues in 5’ and 3’ stems of hairpin structures. Level of nucleotide identity is indicated below the alignment.

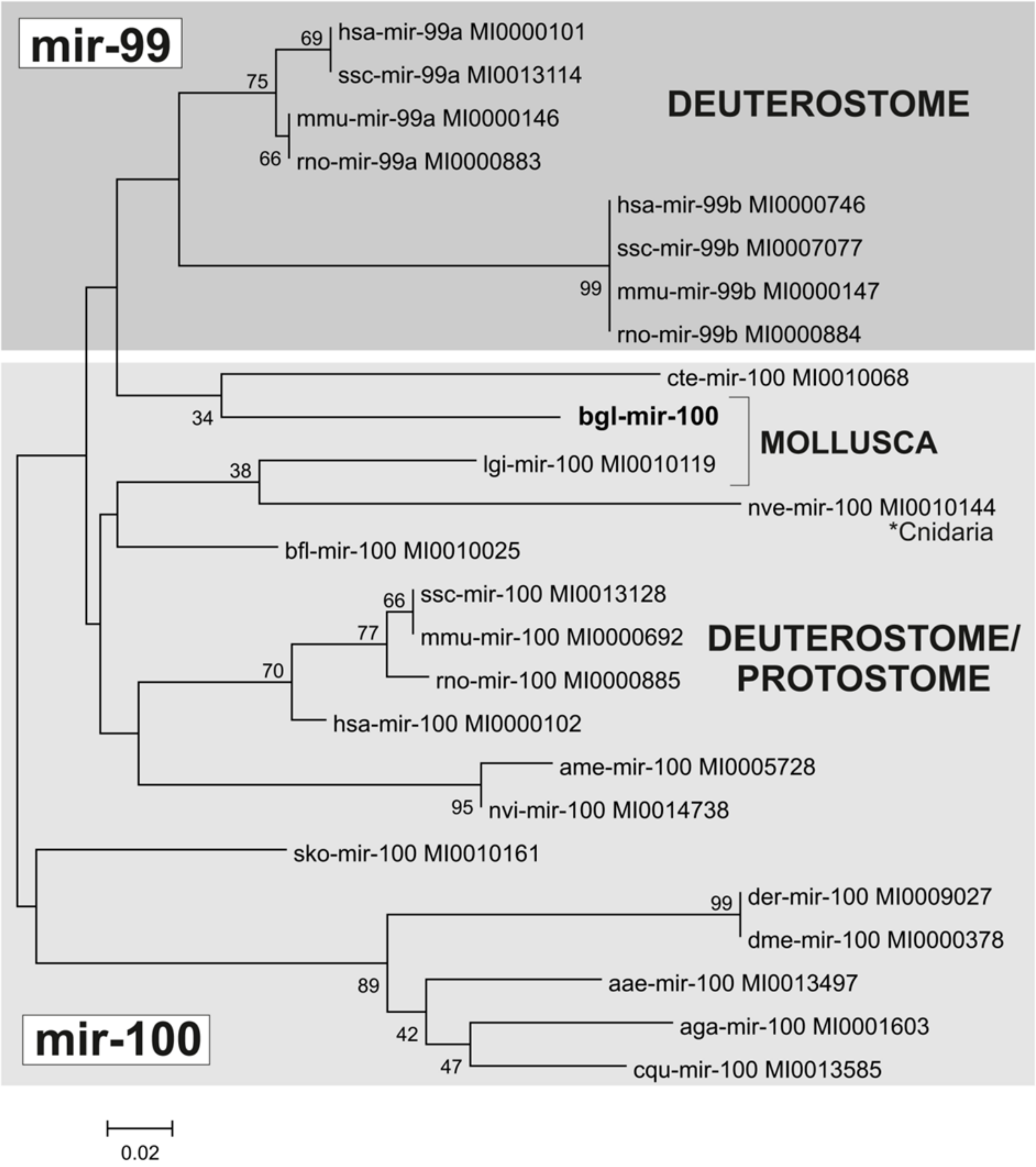

Supplementary figure 43: Phylogenetic tree of bgl-mir-100 (bold) with animal homologs and miR-99 precursors.

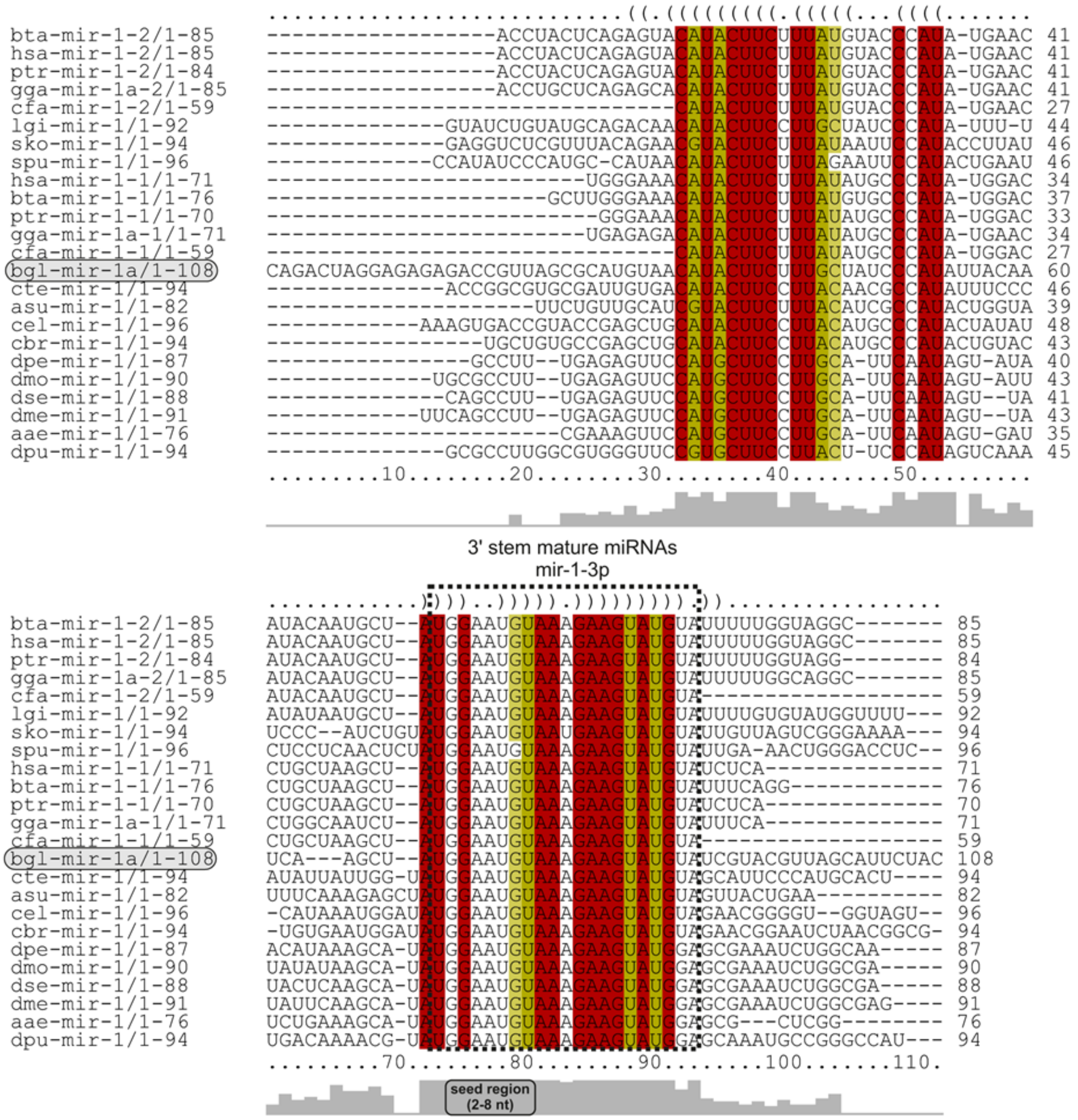

Supplementary figure 44: Alignment of the bgl-mir-1a with homologs from Bilateria. Brackets and colors identify matching residues in 5’ and 3’ stems of hairpin structures. Level of nucleotide identity is indicated below the alignment.

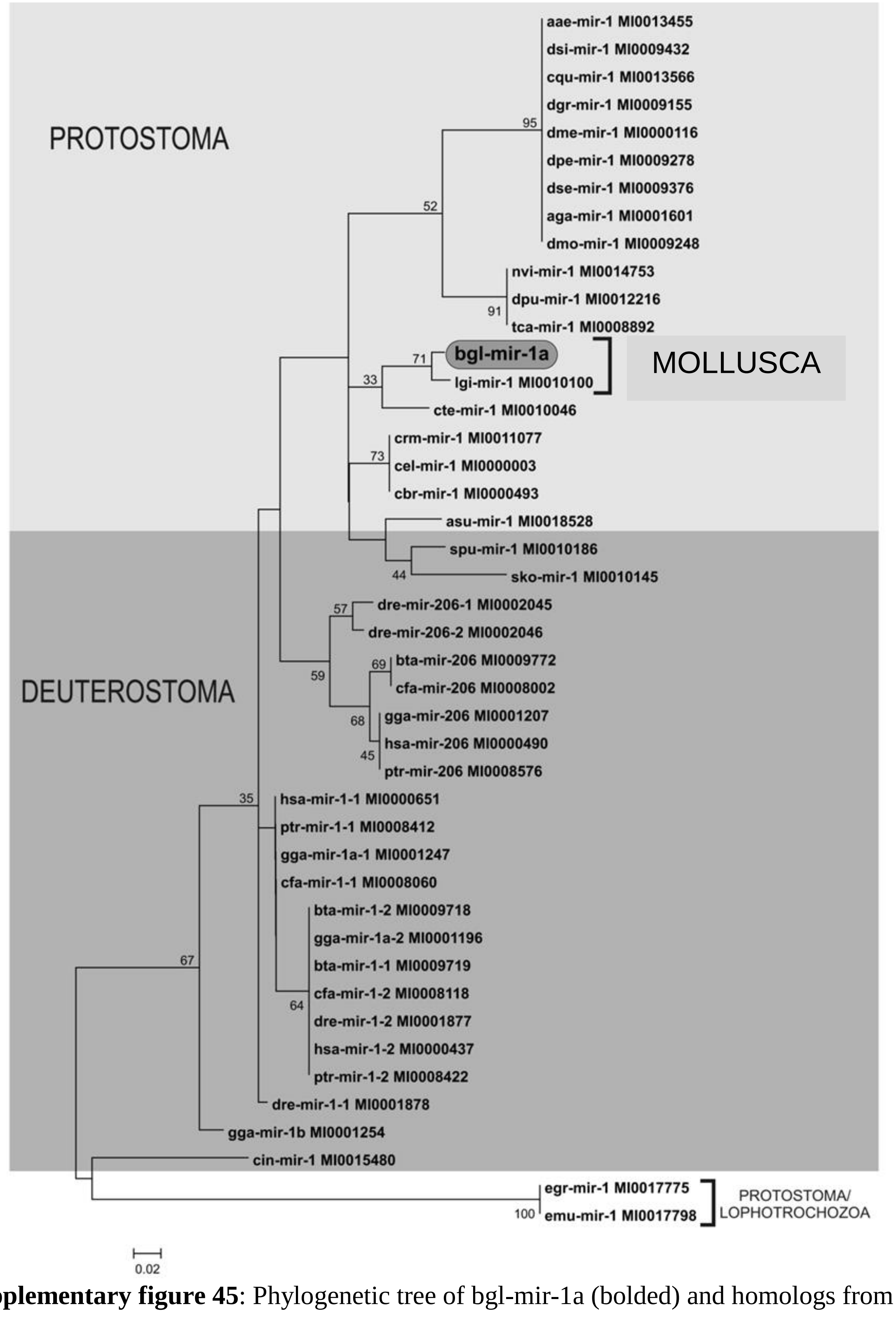

Supplementary figure 45: Phylogenetic tree of bgl-mir-1a (bolded) and homologs from Bilateria.

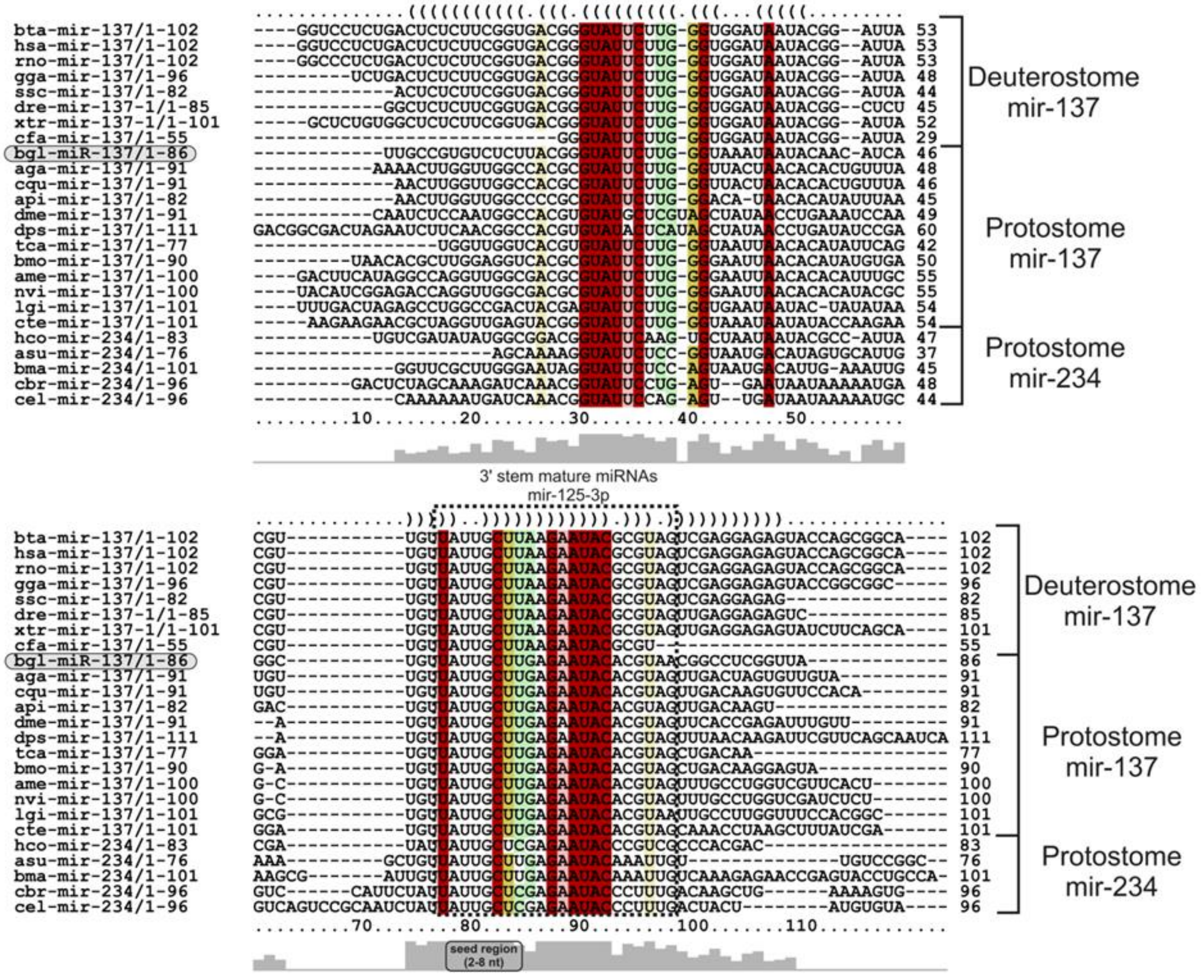

Supplementary figure 46: Alignment of bgl-mir-137 with homologs from Bilateria. Brackets and colors identify matching residues in 5' and 3' stems of hairpin structures. Level of nucleotide identity is indicated below the alignment.

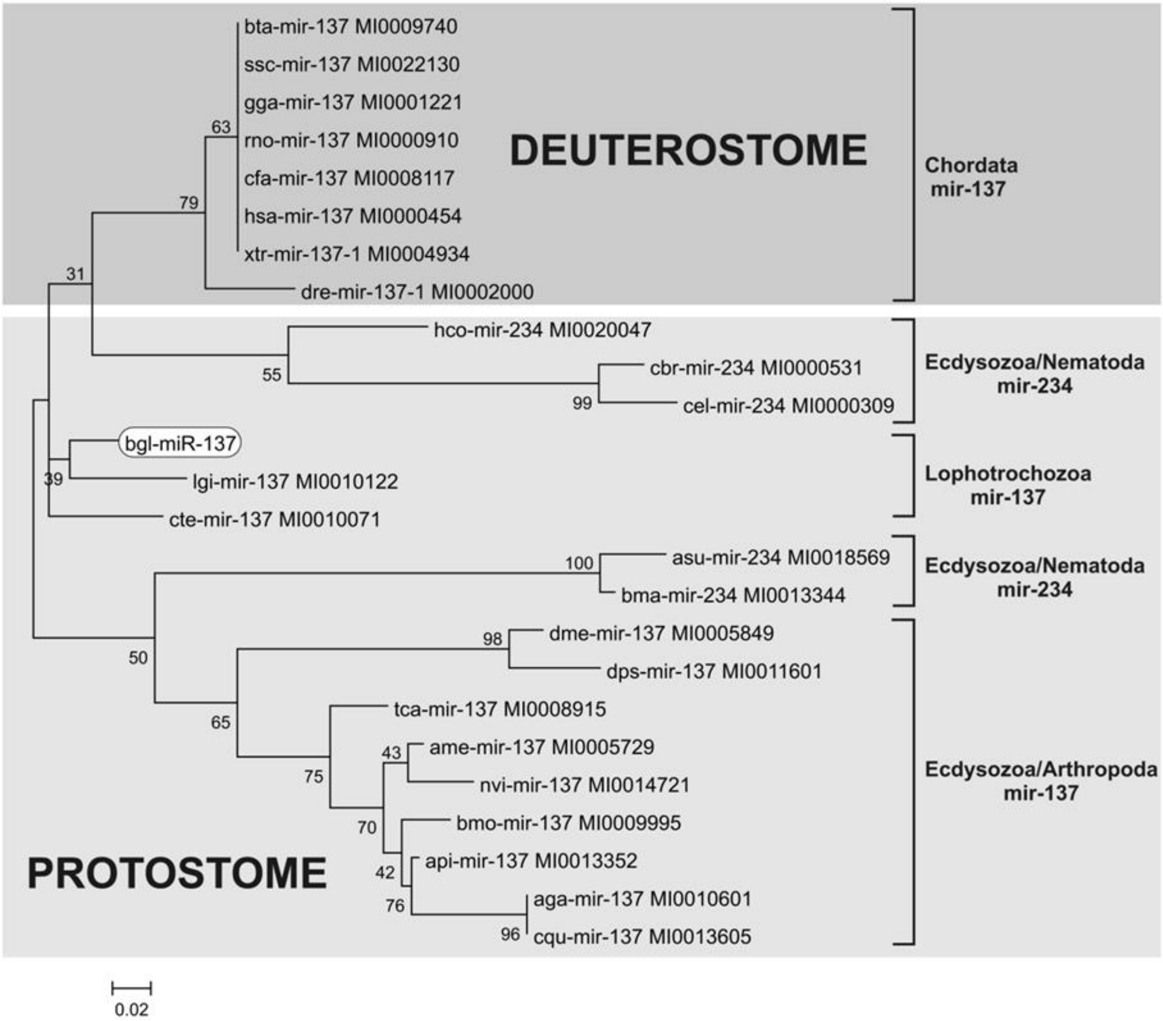

Supplementary figure 47: Phylogenetic tree of bgl-mir-137 (boxed) and homologs from Bilateria.

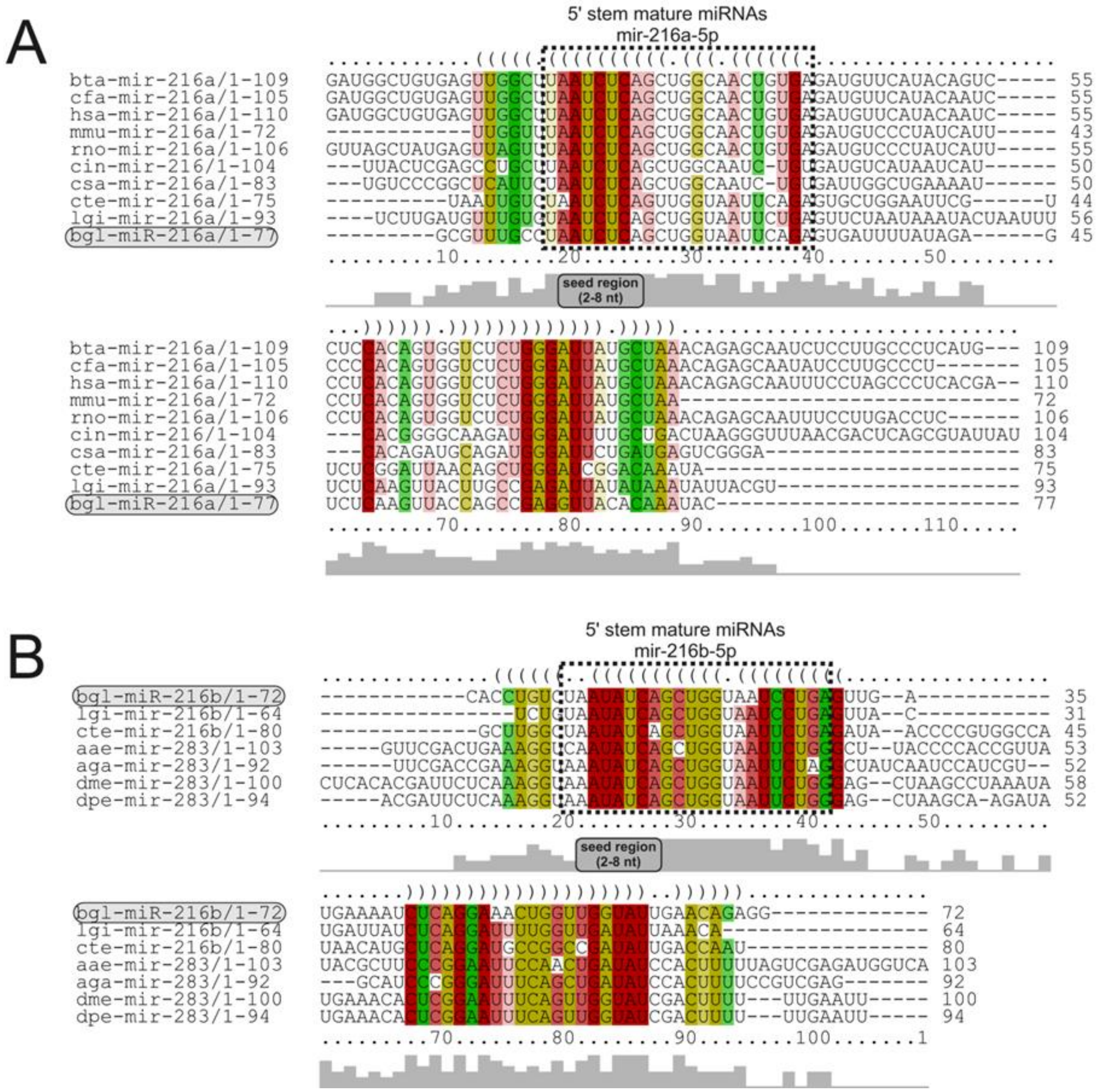

Supplementary figure 48: Alignment of (A) bgl-mir-216a and (B) bgl-mir-216b with homologs from Bilateria. Brackets and colors identify matching residues in 5' and 3' stems of hairpin structures. Level of nucleotide identity is indicated below the alignment.

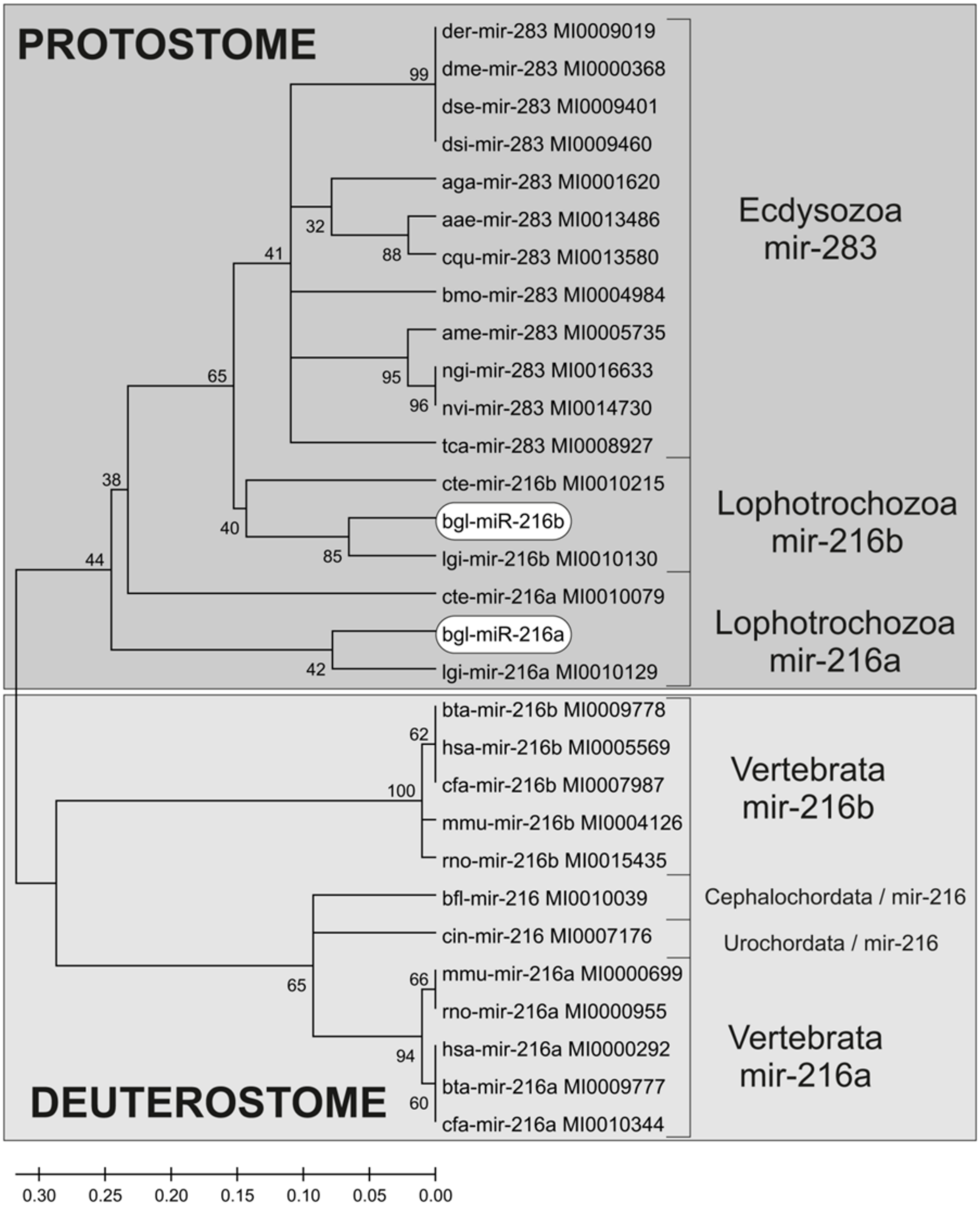

Supplementary figure 49: Phylogenetic tree of mir-216 family, bgl-mir-216a/216b (boxed), and homologs from Bilateria and mir-283 homologs.

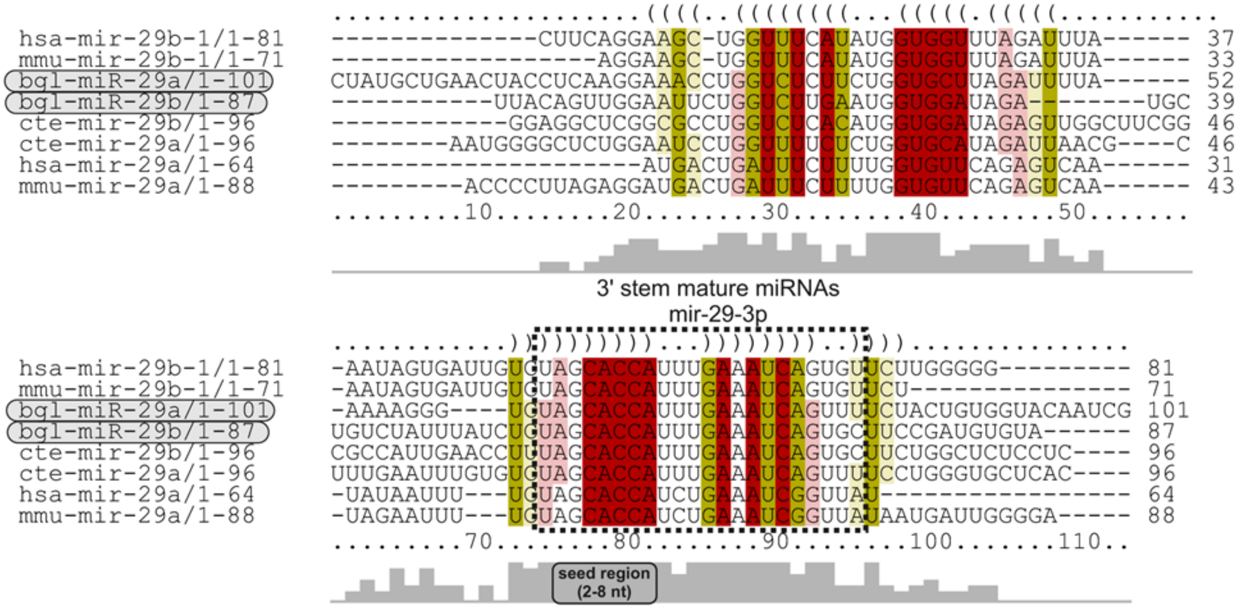

Supplementary figure 50: Alignment of bgl-mir-29a and bgl-mir-29b with homologs from Bilateria. Brackets and colors identify matching residues in 5’ and 3’ stems of hairpin structures. Level of nucleotide identity is indicated below the alignment.

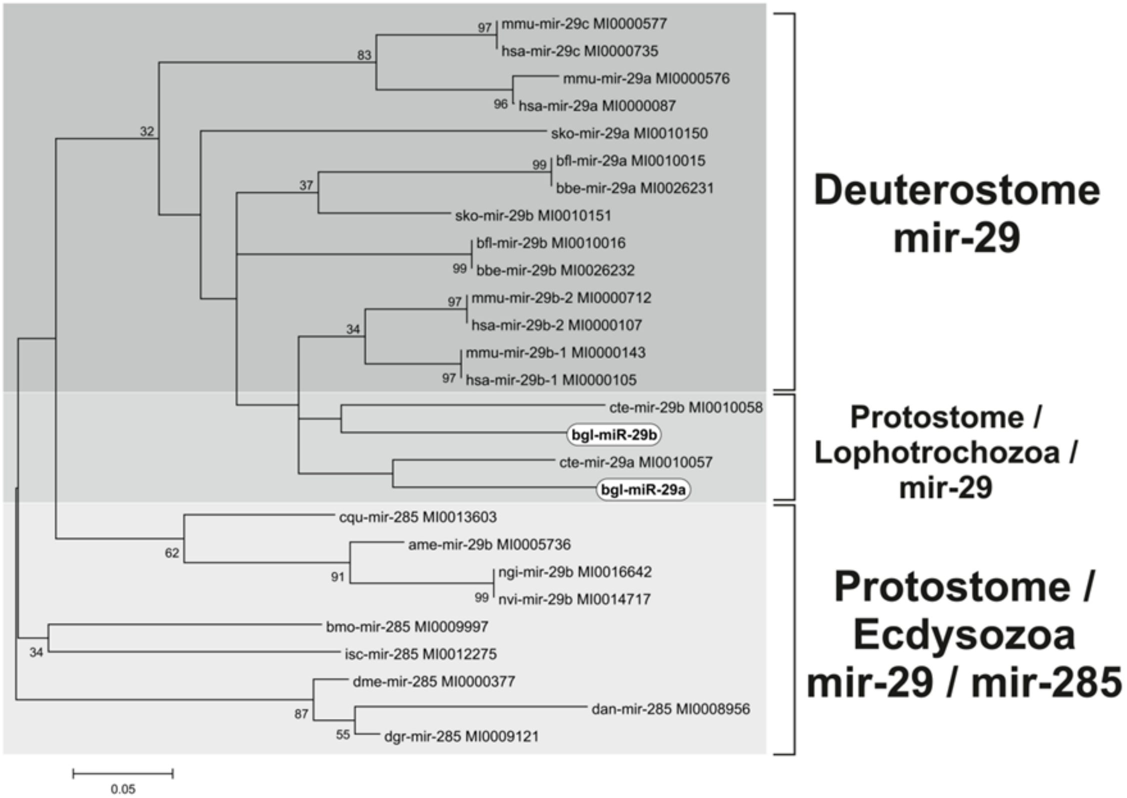

Supplementary figure 51: Phylogenetic tree of bgl-mir-29a and bgl-mir-29b with homologs from Bilateria and mir-285 homologs.

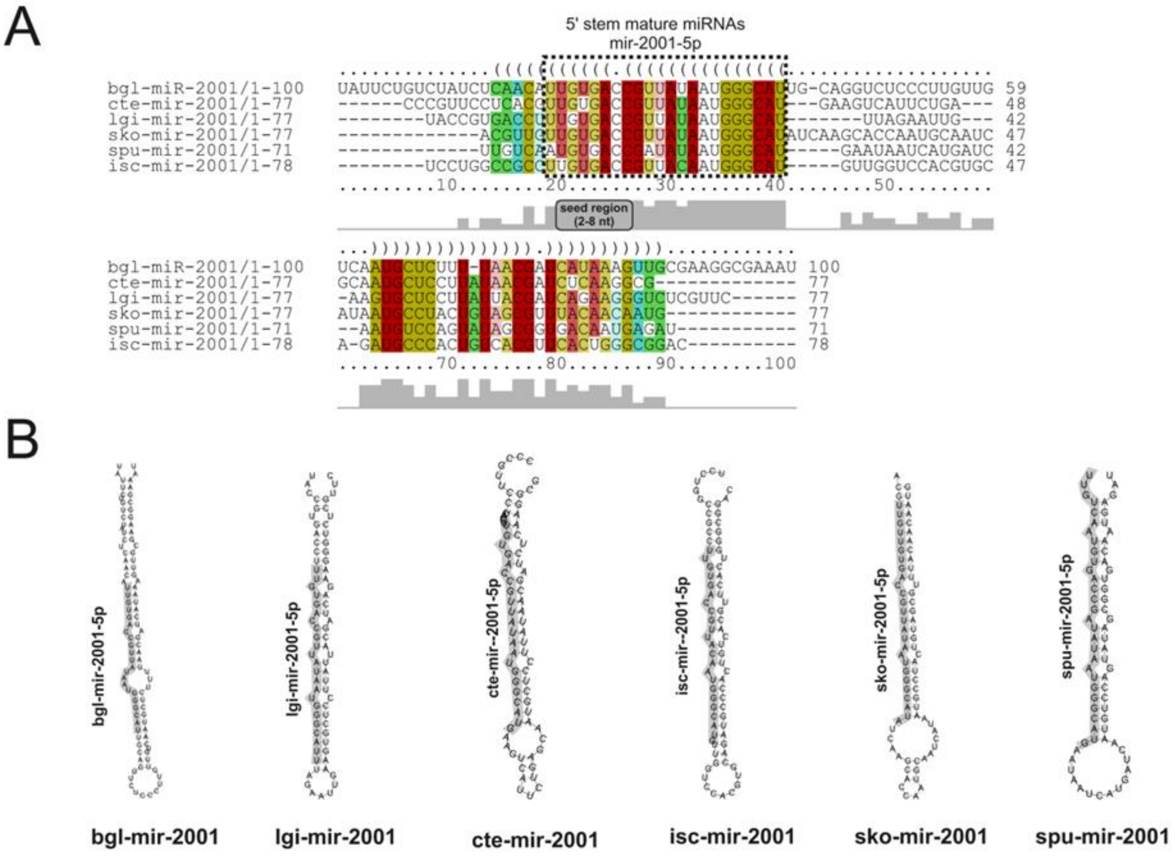

Supplementary figure 52: (A) Alignment of bgl-mir-2001 with homologs from Bilateria (colors and brackets identify matching residues in 5’ and 3’ stems of hairpin structures, Level of nucleotide identity is indicated below the alignment) and (B) secondary structure highlighting the mature miRNAs.

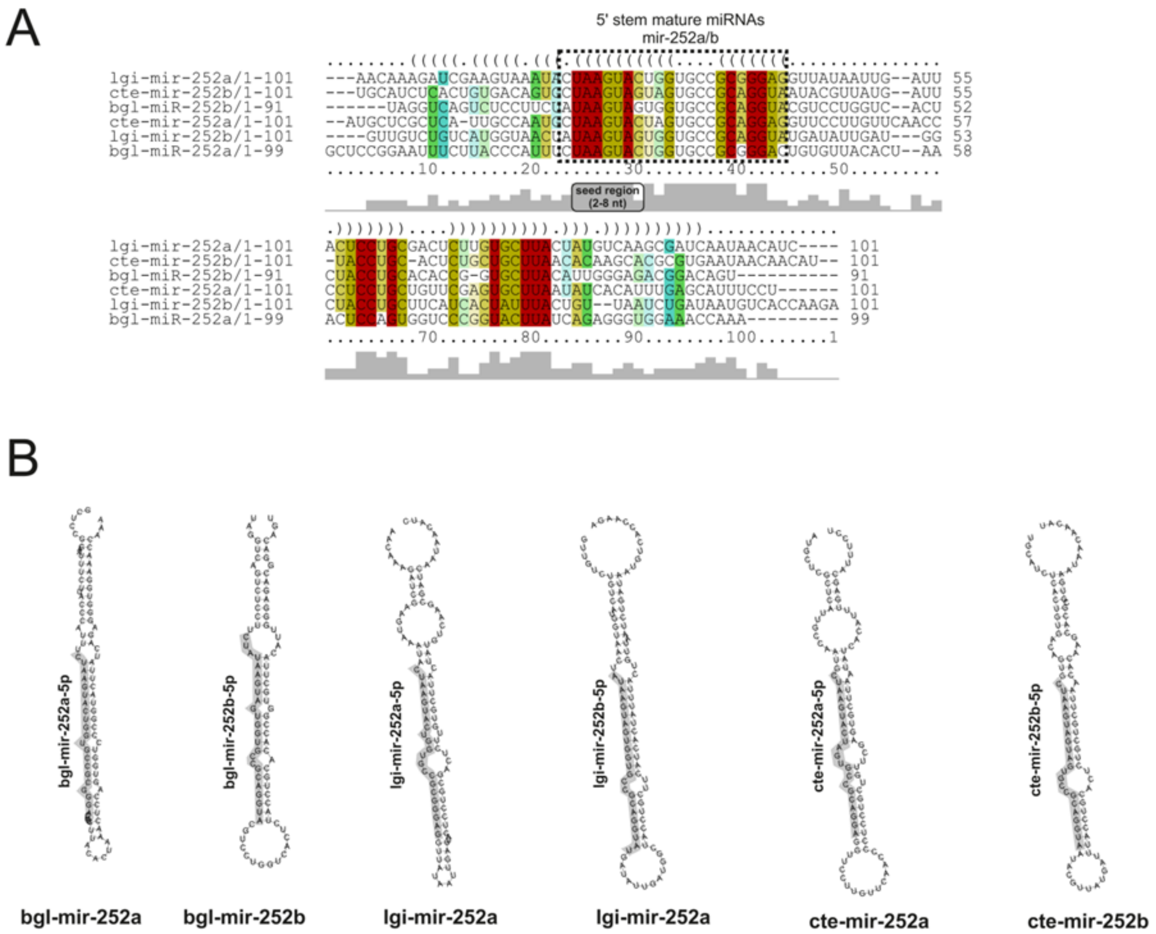

Supplementary figure 53: (A) Alignment of bgl-mir-252a and bgl-mir-252b with homologs from Bilateria (colors identify matching residues in 5’ and 3’ stems of hairpin structures) and (B) secondary structure highlighting the mature miRNAs.

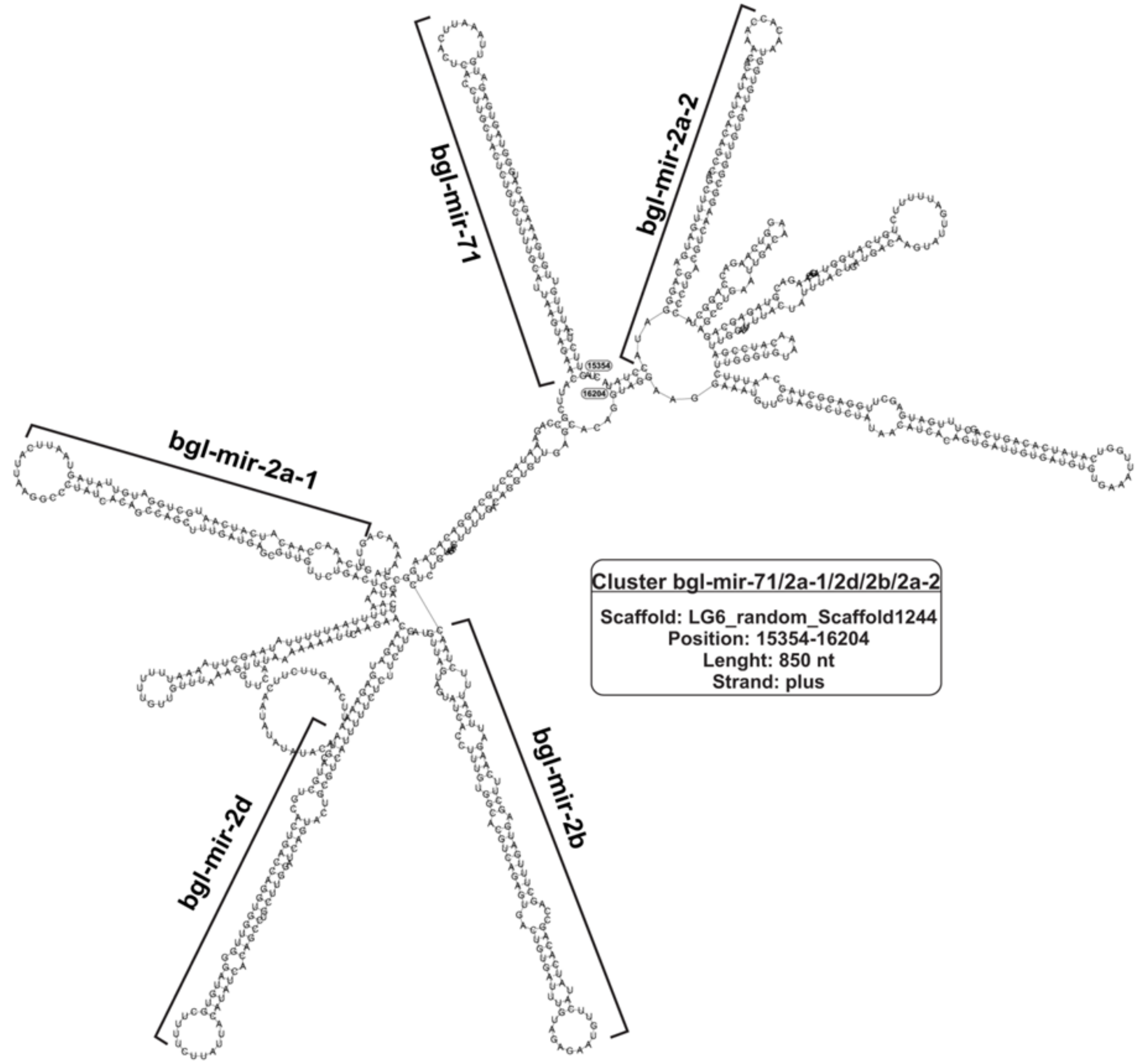

Supplementary figure 54: RNA secondary structure of the cluster bgl-mir-71/2a-1/2d/2b/2a-2

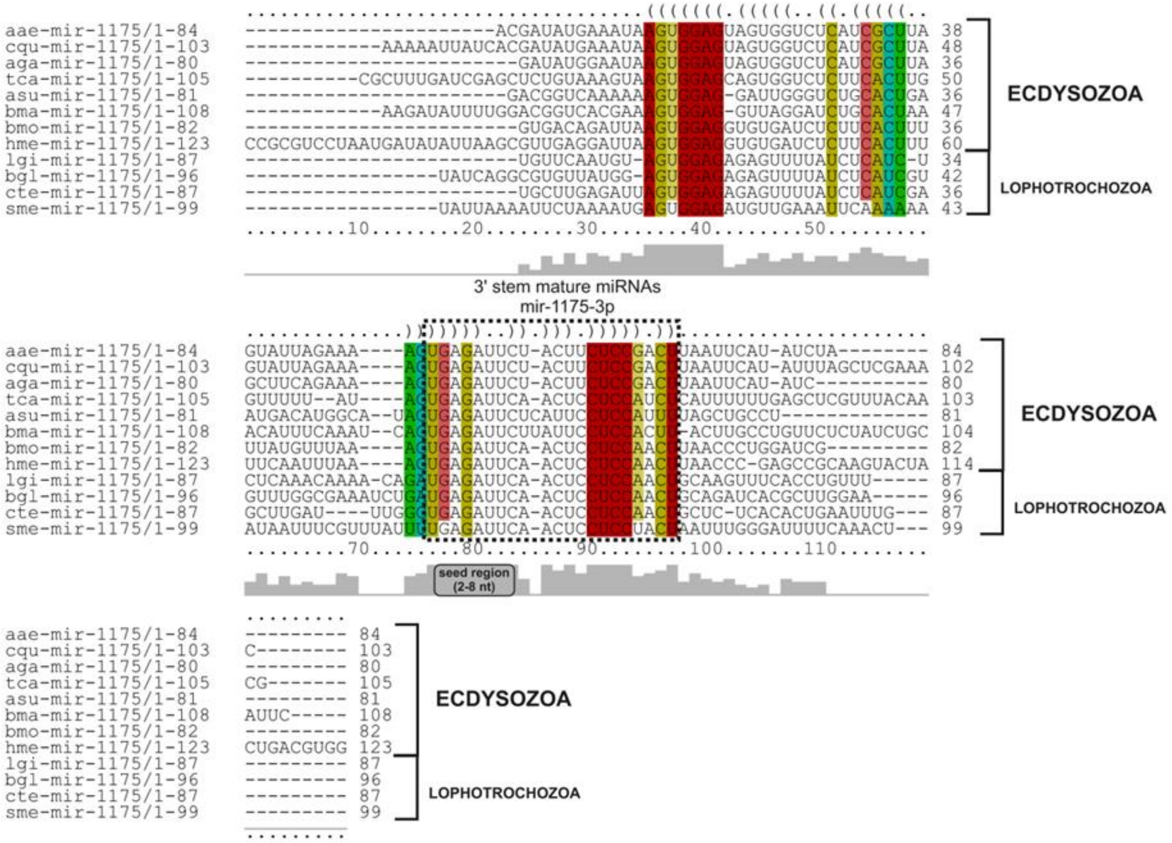

Supplementary figure 55: Alignment of the bgl-mir-1175 with homologs from Protostomia. Colors identify matching residues in 5’ and 3’ stems of hairpin structures.

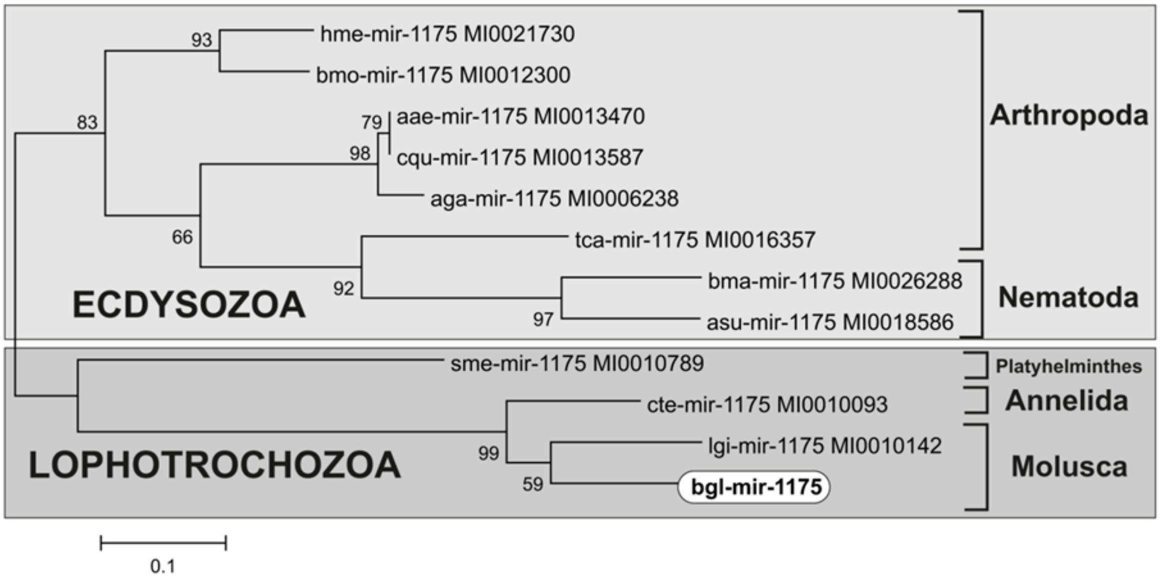

Supplementary figure 56: Phylogenetic tree of bgl-mir-1175 (boxed) and homologs from Protostomia

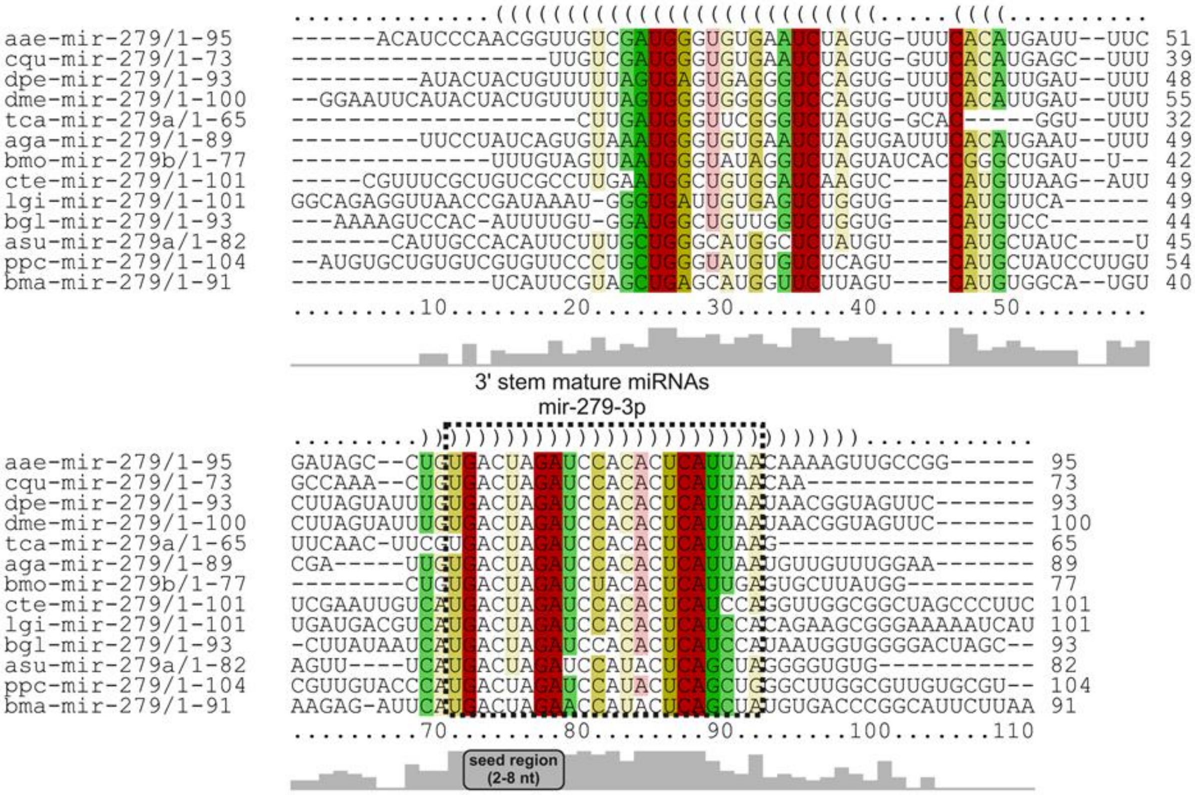

Supplementary figure 57: Alignment of bgl-mir-279 with homologs from Protostomia. Colors identify matching residues in 5' and 3' stems of hairpin structures.

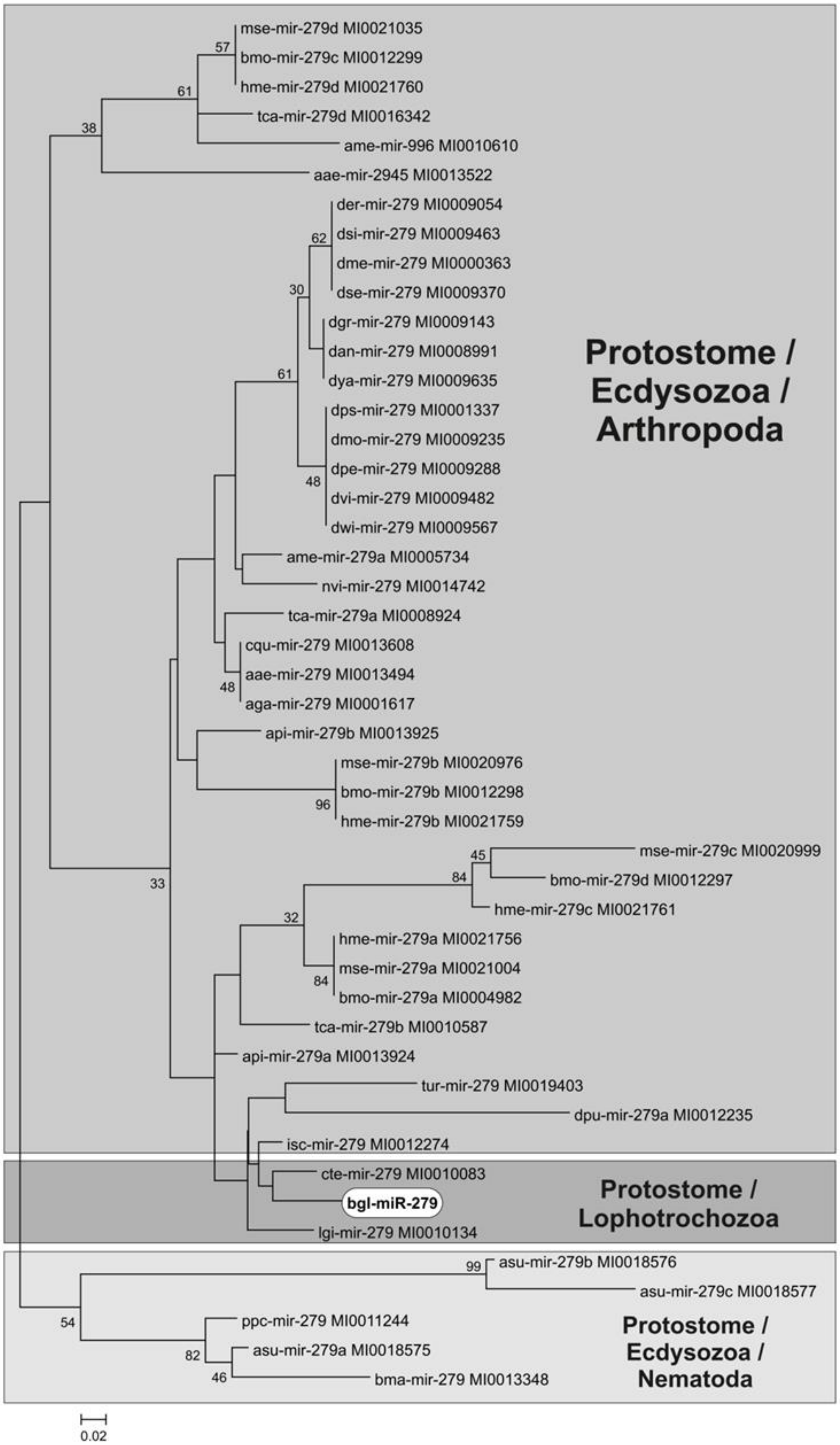

Supplementary figure 58: Phylogenetic tree of bgl-mir-279 (boxed) and homologs from Protostomia

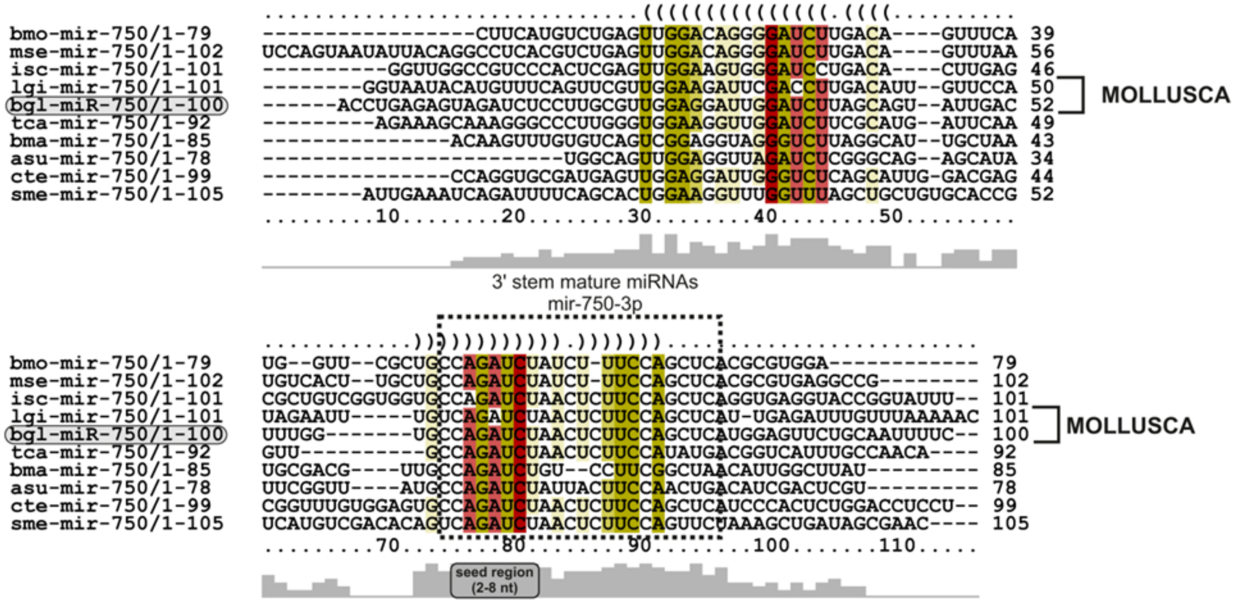

Supplementary figure 59: Alignment of bgl-mir-750 with homologs from Protostomia. Colors identify matching residues in 5’ and 3’ stems of hairpin structures.

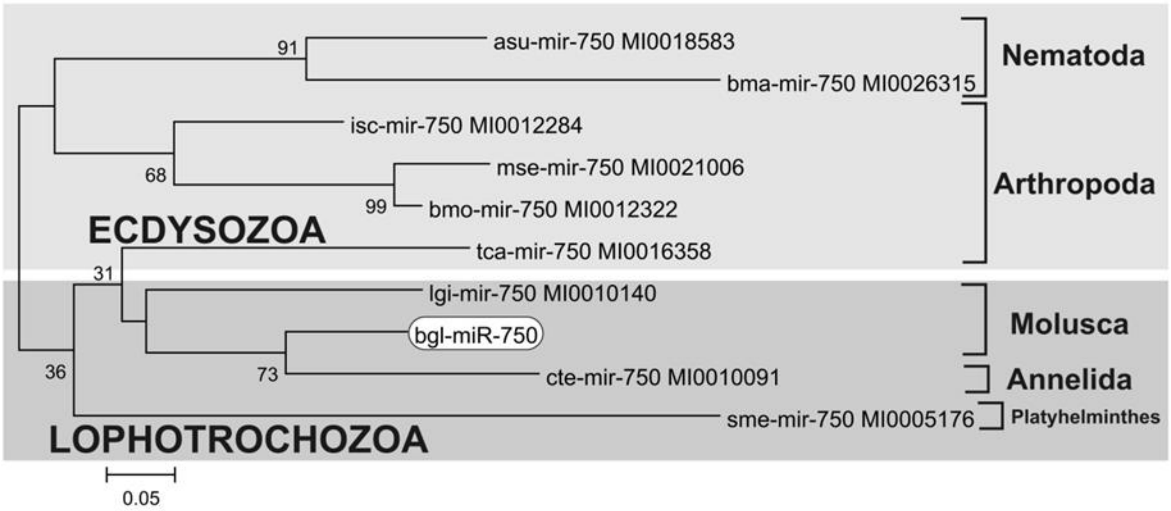

Supplementary figure 60: Phylogenetic tree of bgl-mir-750 (boxed)and homologs from Protostomia.

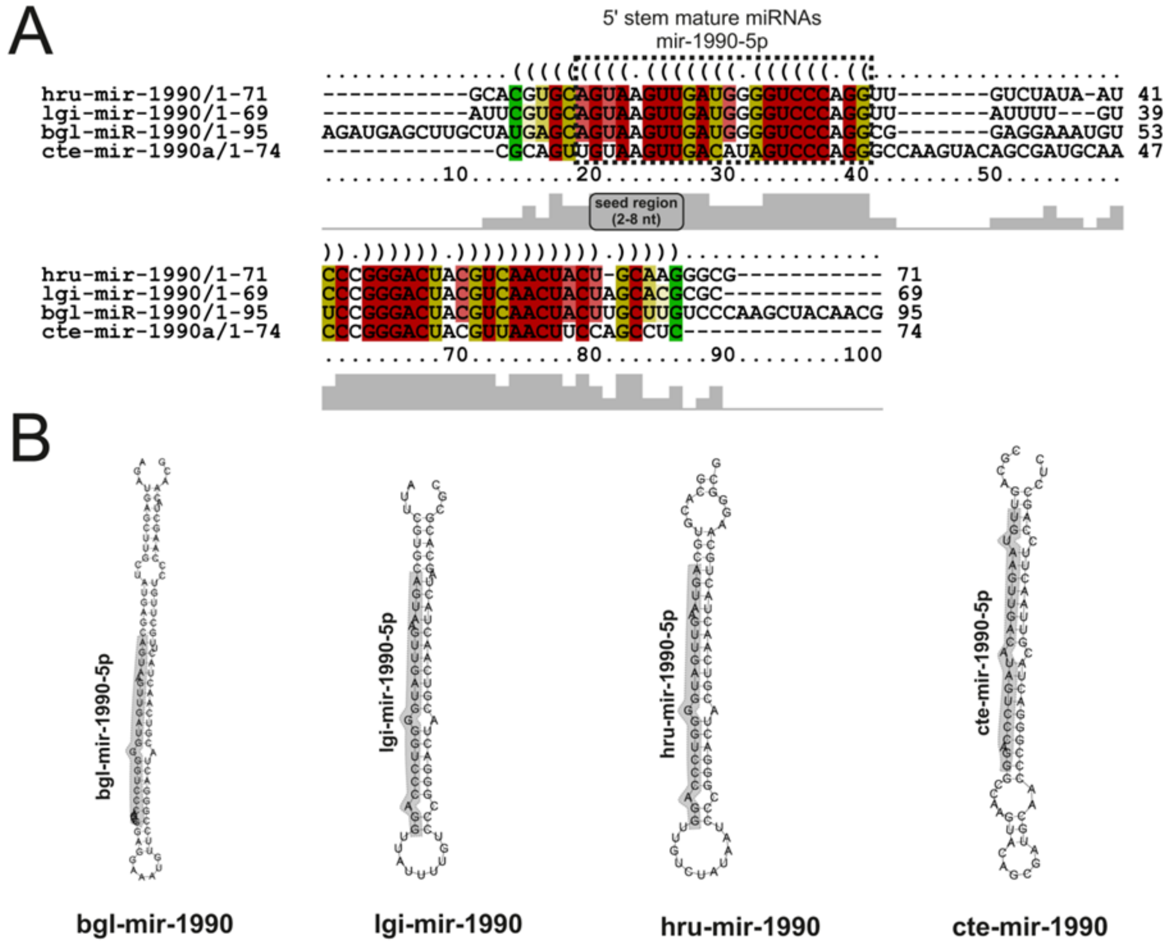

Supplementary figure 61: (A) Alignment of bgl-mir-1990 with homologs from Lophotrochozoa (colors identify matching residues in 5’ and 3’ stems of hairpin structures) and (B) secondary structure highlighting the mature miRNAs.

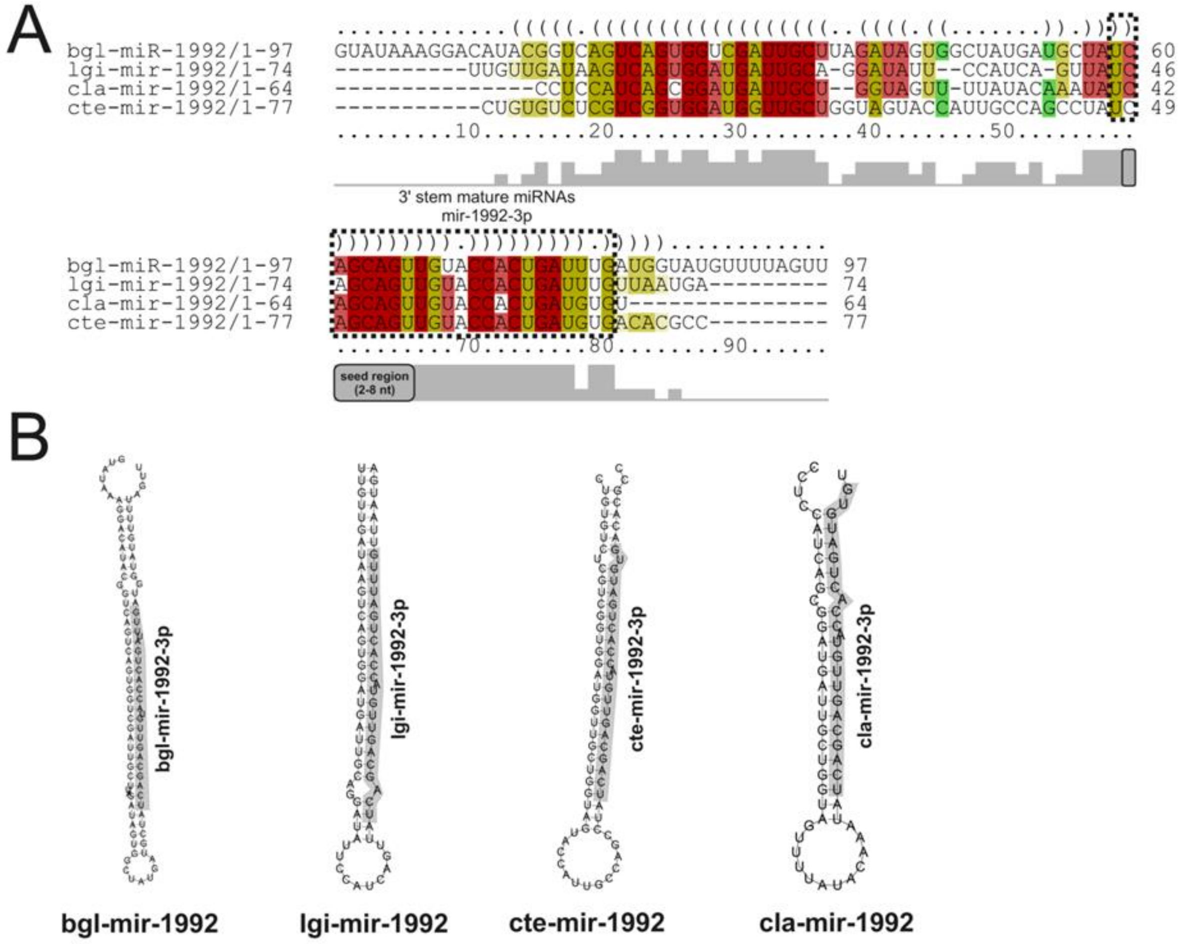

Supplementary figure 62: (A) Alignment of bgl-mir-1992 with homologs from Lophotrochozoa (clors identify matching residues in 5' and 3' stems of hairpin structures) and (B) secondary structure highlighting the mature miRNAs.

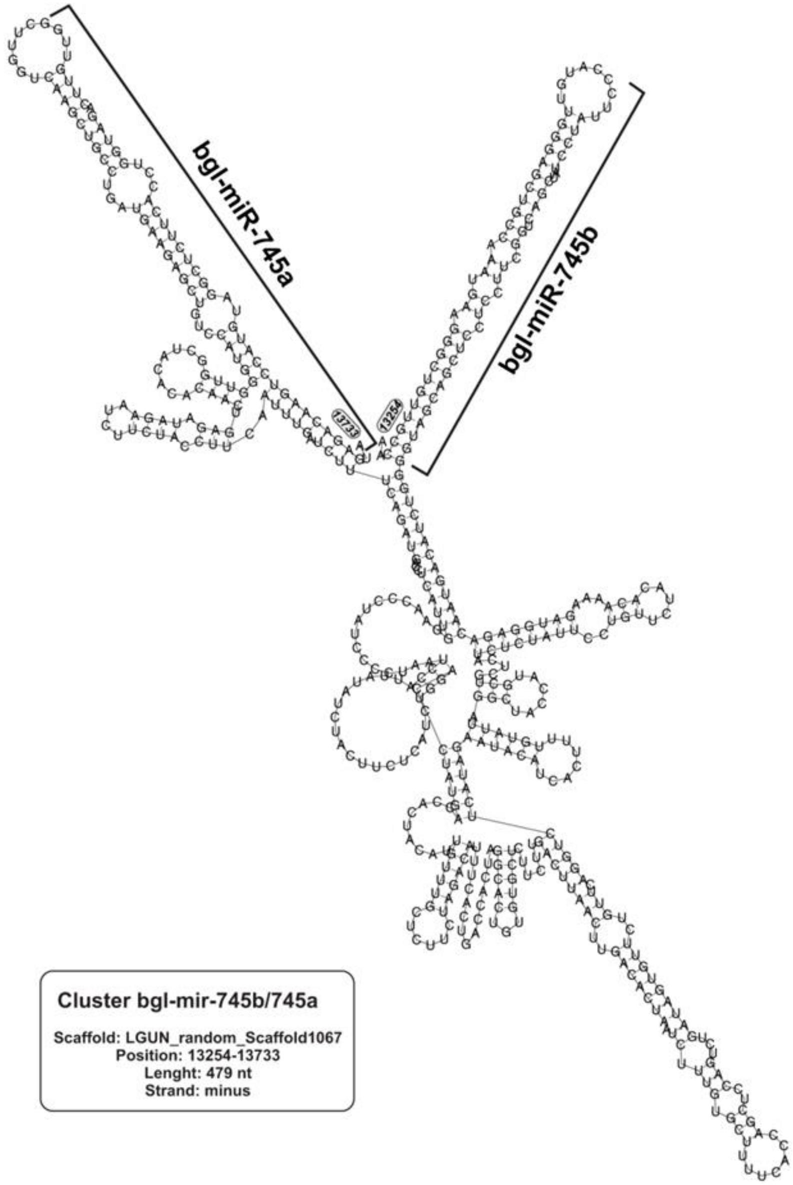

Supplementary figure 63: RNA secondary structure of the cluster bgl-mir-745a/745b.

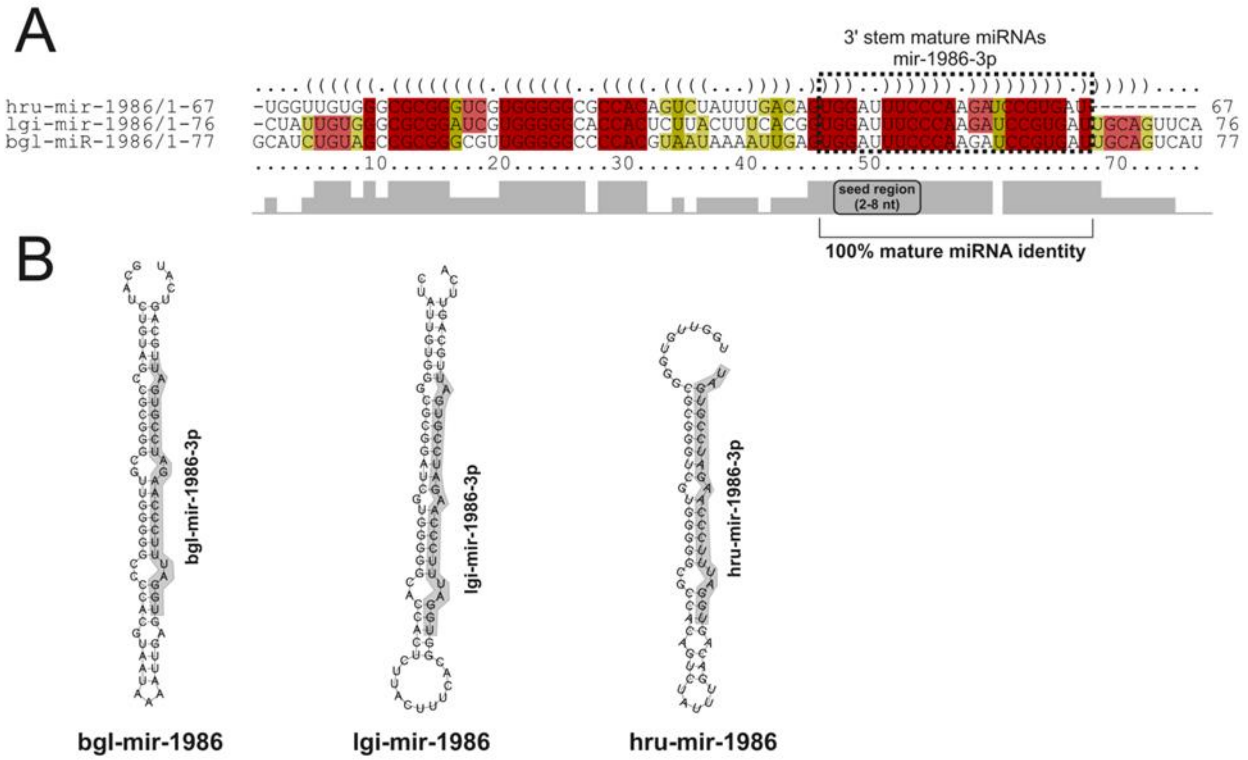

Supplementary figure 64: (A) Alignment of bgl-mir-1986 with homologs from Mollusca (colors identify matching residues in 5' and 3' stems of hairpin structures) and (B) secondary structure highlighting the mature miRNAs.

miR-1984

```
               ***********************
bgl-miR-1984   UGCCCUAUCCGUCAGGAACUGUG
lgi-miR-1984   UGCCCUAUCCGUCAGGAACUGUG
```

miR-1985

```
               ***********************
bgl-miR-1985   UGCCAUUUUUAUCAGUCACUGUG
lgi-miR-1985   UGCCAUUUUUAUCAGUCACUGUGA
```

miR-1994a

```
                ******************************
bgl-miR-1994a   UGAGACAGUGUGUCCUCCCUUG
lgi-miR-1994a   UGAGACAGUGUGUCCUCCCUUG
```

miR-1994b

```
                **********************
bgl-miR-1994b   UGAGACAGUGCGUCCUCCCUCA
lgi-miR-1994b   UGAGACAGUGUGUCCUCCCUCA
```

miR-1991

```
                ***********  *  *****
bgl-miR-1991   CUUACCCUGUUAUACUGAGAAGU
lgi-miR-1991   GUUACCCUGUUAAUCGAAGAAGU
```

miR-1992

```
               ***********************
bgl-miR-1992   UCAGCAGUUGUACCACUGAUUUG
lgi-miR-1992   UCAGCAGUUGUACCACUGAUUUG
```

miR-2001

```
               ***********************
bgl-miR-2001   UUGUGACCGUUAUAAUGGGCAUU
lgi-miR-2001   UUGUGACCGUU UAAUGGGCAUU
```

let-7

```
               ****************** ***
bgl-let-7-5p   UGAGGUAGUAGGUUGUAUUGUU
lgi-let-7      UGAGGUAGUAGGUUGUAUAGUU
```

miR-100

```
                  ********** ***********
bgl-miR-100-5p    AACCCGUAGAACCGAACUUGUGCG
lgi-miR-100       AACCCGUAGAUCCGAACUUGUG
```

miR-1993

```
               ********** ***********
bgl-miR-1993   UAUUAUGCUGCUAUUCACGAGA
lgi-miR-1993   UAUUAUGCUGAUAUUCACGAGA
```

miR-190

```
              **************** *****
bgl-miR-190   AGAUAUGUUUGAUAUAUUUGGUGG
lgi-miR-190   AGAUAUGUUUGAUAUACUUGGU
```

miR-216a

```
               ****************** ***
bgl-miR-216a   UAAUCUCAGCUGGUAAUUCAGAG
lgi-miR-216a   UAAUCUCAGCUGGUAAUUCUGAG
```

miR-281

```
                  **********************
bgl-miR-281       UGUCAUGGAGUUGCUCUCUUUA
lgi-miR-281-3p    UGUCAUGGAGUUGCUCUCUUUA
```

miR-216b

```
               ***********************
bgl-miR-216b   UAAUAUCAGCUGGUAAUCCUGAG
lgi-miR-216b   UAAUAUCAGCUGGUAAUCCUGAG
```

miR-29a

```
              **********************
bgl-miR-29a   UAGCACCAUUUGAAAUCAGUUU
lgi-miR-29    UAGCACCAUUUGAAAUCAGUUU
```

miR-71

```
             ********* *************
bgl-miR-71   UGAAAGACAUGGGUAGUGAGAUG
lgi-miR-71   UGAAAGACAAGGGUAGUGAGAUG
```

miR-745a

```
               *********************
bgl-miR-745a   AGCUGCCUGAUGAAGAGCUGU
lgi-miR-745a   AGCUGCCUGAUGAAGAGCUGU
```

miR-34

```
             ******************** **
bgl-miR-34   UGGCAGUGUGGUUAGCUGGUUGU
lgi-miR-34   UGGCAGUGUGGUUAGCUGGUAGU
```

miR-745b

```
               *********************
bgl-miR-745b   AGCUGCCAAAUGAAGGGCUGUU
lgi-miR-745b   AGCUGCCAAAUGAAGGGCUGUG
```

miR-7

```
            ***************** ******
bgl-miR-7   UGGAAGACUAGUGAUUUAGUUGUU
lgi-miR-7   UGGAAGACUAGUGAUUUUGUUGUU
```

miR-9a

```
                ***********************
bgl-miR-9a-5p   UCUUUGGUUAUCUAGCUGUAUGA
lgi-miR-9       UCUUUGGUUAUCUAGCUGUAUGA
```

miR-8

```
                ***********************
bgl-miR-8-3p    UAAUACUGUCAGGUAAAGAUGUC
lgi-miR-8       UAAUACUGUCAGGUAAAGAUGUC
```

miR-278

```
                  ******************** *
bgl-miR-278       UCGGUGGGACUUUCGUUCGUUU
lgi-miR-278       UCGGUGGGACUUUCGUUCGUCU
```

miR-375

```
                  **********************
bgl-miR-375-3p    UUUGUUCGUUCGGCUCGCGUUAU
lgi-miR-375       UUUGUUCGUUCGGCUCGCGUUA
```

miR-33

```
                  ******************** *
bgl-miR-33-5p     GUGCAUUGUAGUUGCAUUGCGUG
lgi-miR-33-5p     GUGCAUUGUAGUUGCAUUGCAU
```

miR-96b

```
                  ***********************
bgl-miR-96b       AUUUGGCACUUGUGGAAUAAUCG
lgi-miR-96b       AUUUGGCACUUGUGGAAUAAUCG
```

miR-96a

```
                  ****************** ***
bgl-miR-96a       CUUGGCACUGGCGGAAUAGUCA
lgi-miR-96a       CUUGGCACUGGCGGAAUAAUCA
```

miR-981

```
                  ********** ************
bgl-miR-981       UUCGUUGUCGUCGAAACCUGCCU
lgi-miR-981       UUCGUUGUCGACGAAACCUGCCU
```

miR-317

```
                  ********************** *
bgl-miR-317       UGAACACAGCUGGUGGUAUCUUAU
lgi-miR-317       UGAACACAGCUGGUGGUAUCUUCU
```

miR-87b

```
                  **********************
bgl-miR-87b-1/2   GUGAGCAAAGUUUCAGGUGUAU
lgi-miR-87        GUGAGCAAAGUUUCAGGUGUAU
```

miR-133

```
                  ***********  **********
bgl-miR-133-5p    AGCUGGUUGAAUCUGGGCCAAAU
lgi-miR-133-5p    AGCUGGUUGAAAUUGGGCCAAAU
```

miR-1a

```
                  **********************
bgl-miR-1a-3p     UGGAAUGUAAAGAAGUAUGUAU
lgi-miR-1         UGGAAUGUAAAGAAGUAUGUAU
```

miR-153

```
                  **********************
bgl-miR-153-3p    UUGCAUAGUCACAAAAGUGAUC
lgi-miR-153       UUGCAUAGUCACAAAAGUGAUC
```

miR-12

```
                  ********** ***********
bgl-miR-12        UGAGUAUUACUUCAGGUACUGAG
lgi-miR-12        UGAGUAUUACAUCAGGUACUGA
```

miR-252a

```
                  *********************
bgl-miR-252a      CUAAGUACUGGUGCCGCGGGA
lgi-miR-252a      CUAAGUACUGGUGCCGCGGGA
```

miR-252b

```
                  **********************
bgl-miR-252b      AUAAGUAGUGGUGCCGCAGGUA
lgi-miR-252b      AUAAGUAGUGGUGCCGCAGGUA
```

miR-1990

```
                  **********************
bgl-miR-1990      AGUAAGUUGAUGGGGUCCCAGG
lgi-miR-1990      AGUAAGUUGAUGGGGUCCCAGG
```

miR-2b

```
                  **********************
bgl-miR-2b        UAUCACAGCCAGCUUUGAUGAGCU
lgi-miR-2c        UAUCACAGCCAGCUUUGAUGAGU
```

miR-2d

```
                  ***********************
bgl-miR-2d        UAUCACAGCCUGCUUGGAUCAGU
lgi-miR-2d        UAUCACAGCCUGCUUGGAUCAGU
```

miR-67

```
                  *********** ***********
bgl-miR-67-1/2    UCACAACCUGCUUGAAUGAGGAC
lgi-miR-67        UCACAACCUGCAUGAAUGAGGAC
```

miR-2722

```
                  **********************
bgl-miR-2722      UGGCGCCGUGGAAACAUCUACC
lgi-miR-2722      UGGCGCCGUGGAAACAUCUACC
```

miR-92

```
                  **********************
bgl-miR-92        AAUUGCACUCGUCCCGGCCUGC
lgi-miR-92        AAUUGCACUUGUCCCGGCCUGC
```

miR-279

```
                  **********************
bgl-miR-279       UGACUAGAUCCACACUCAUCCA
lgi-miR-279       UGACUAGAUCCACACUCAUCCA
```

miR-1175

```
                  ***********************
bgl-miR-1175-3p   UGAGAUUCAACUCCUCCAACUGC
lgi-miR-1175-3p   UGAGAUUCAACUCCUCCAACUGC
```

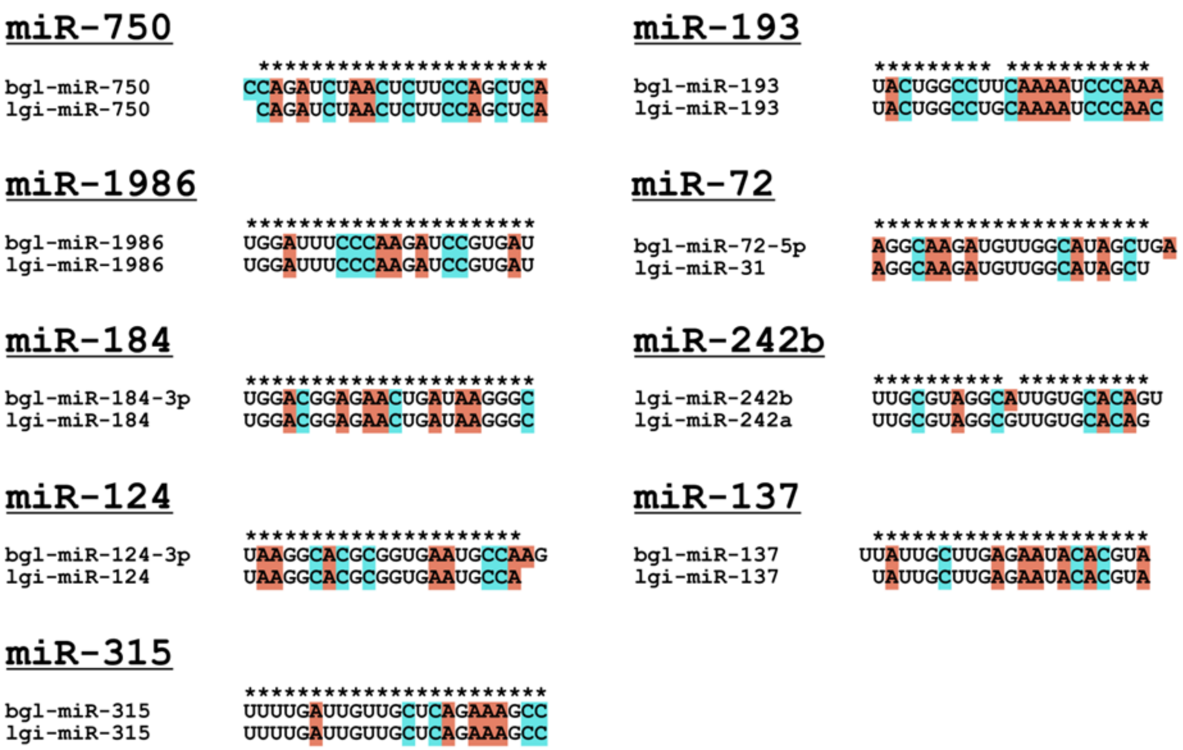

Supplementary figure 65: Alignment of *B. glabrata* mature miRNAs (bgl) with orthologs from *Lottia gigantea* (Lgi), * = identical nucleotides

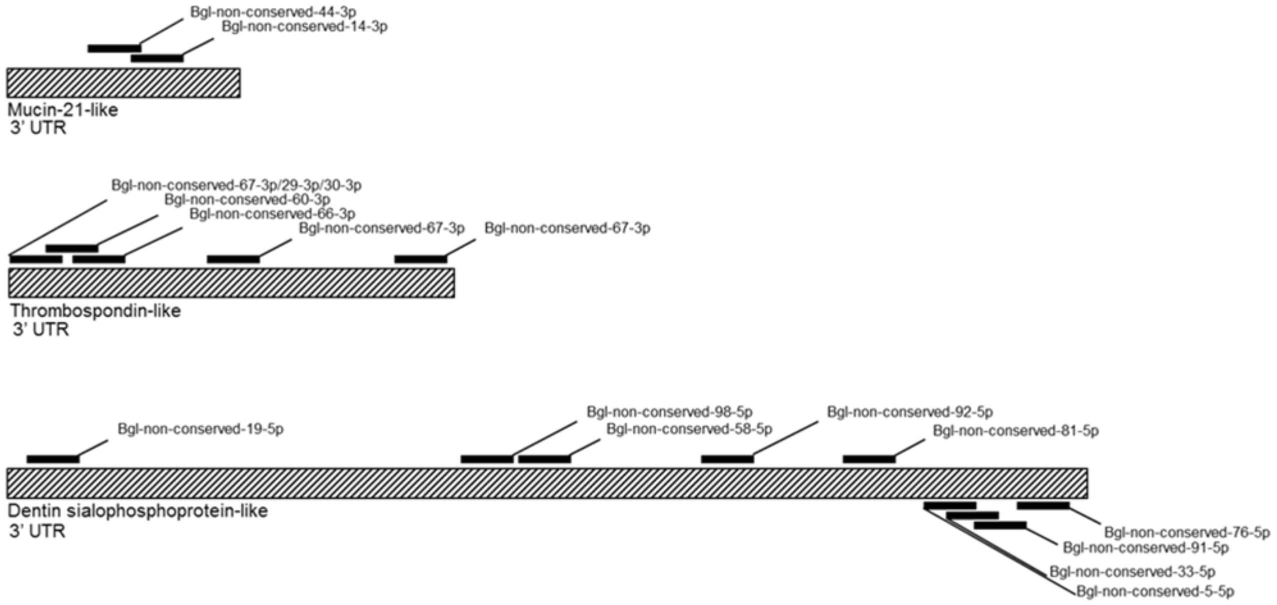

Figure 66. Identified binding sites of novel microRNAs from *B. glabrata*. MicroRNA binding sites were identified for novel predicted mature microRNA from *B. glabrata*. Targets were identified from the 3'UTR sequences of available *B. glabrata* transcripts.

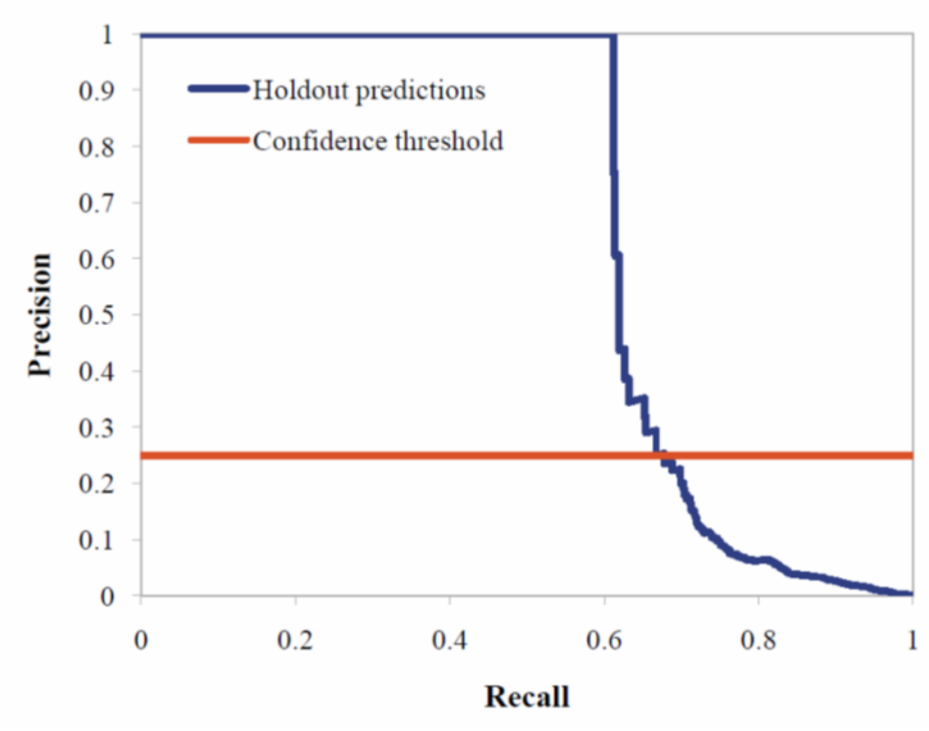

Supplementary figure 67. Precision-recall on *Anolis carolinensis* hold-out data.

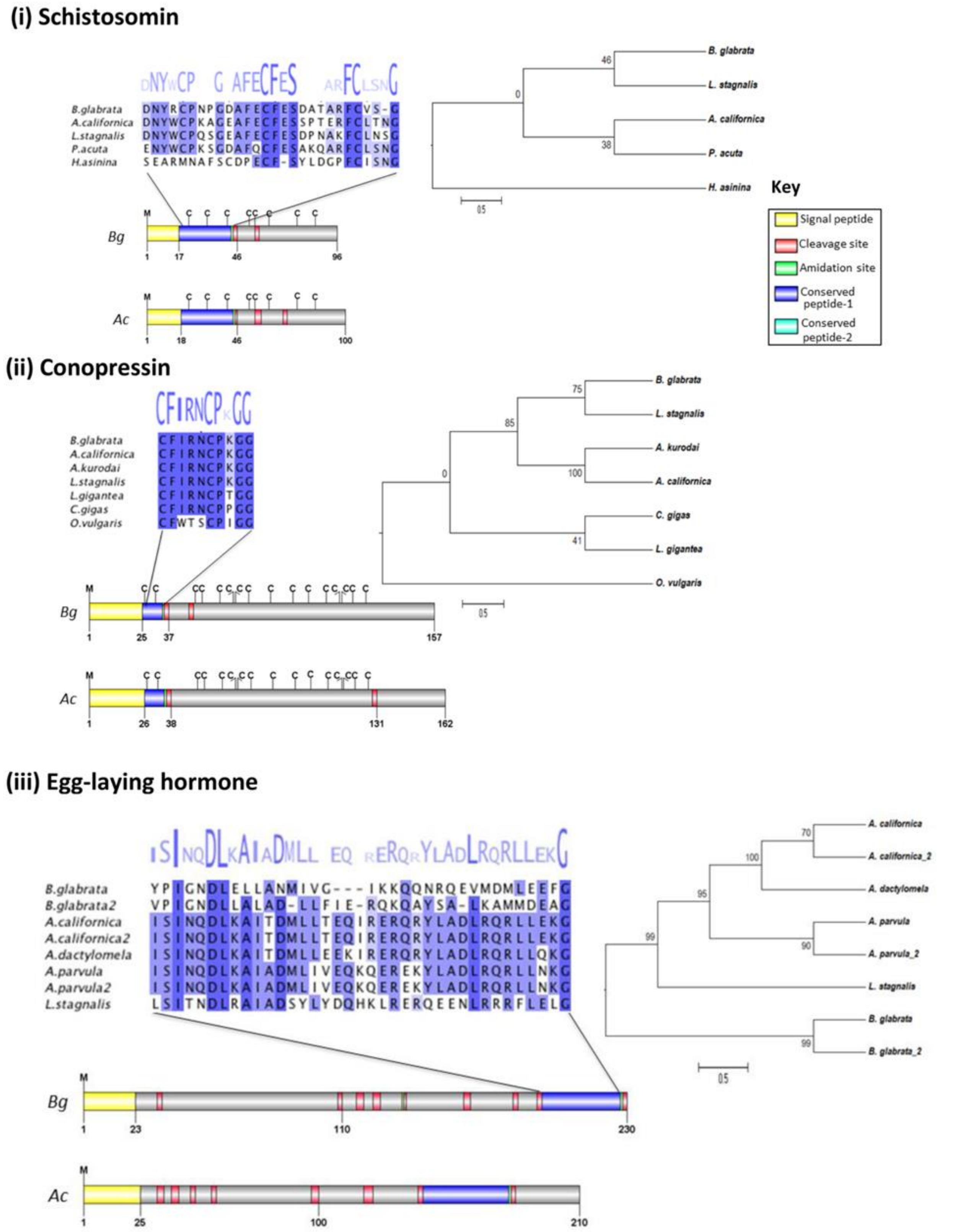

(**Supplementary Figure 68**. Characterisation of five *Biomphalaria glabrata* neuropeptides, legend next page)

(iv) Whitnin

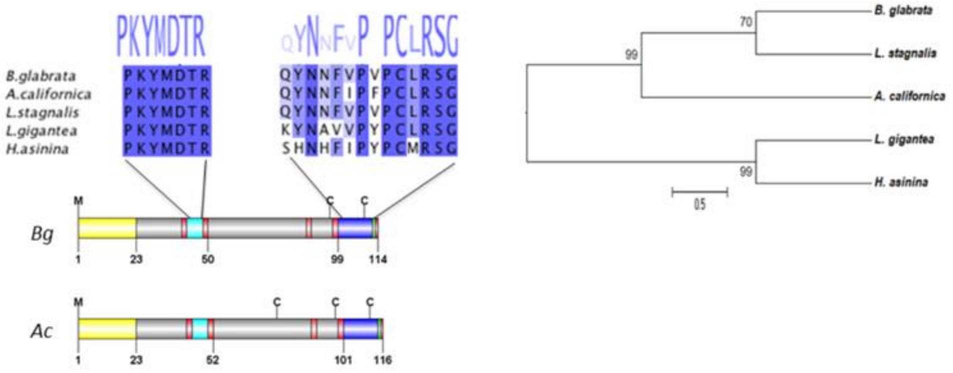

(v) Gonadotropin-releasing hormone

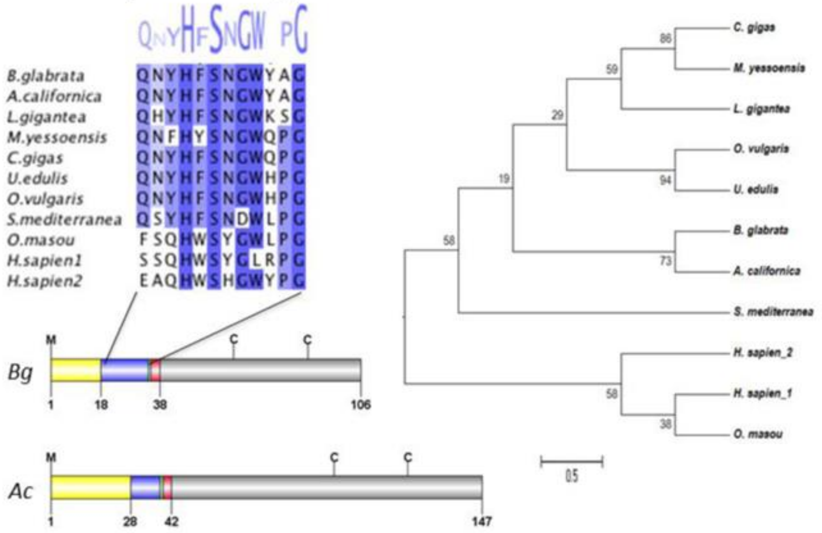

Supplementary Figure 68. Characterisation of five *Biomphalaria glabrata* neuropeptides. Multiple sequence alignment of *B. glabrata* neuropeptide with different species,alignments are shaded using the ClustalX coloring scheme (see Clamp *et al*. 2004). Also shown is comparative cross-species phylogenetic analyses using Maximum likelihood method with MEGA5, under Jones-Taylor-Thorton (JTT) substitution model (Jones *et al*., 1992) with a gamma distribution of rates between sites (eight categories, parameter alpha, estimated by the program). Schematics representation of neuropeptides precursors in *B. glabrata* (Bg) is compared to *A. californica* (Ac) showing signal peptide, cleavage sites, cysteine organization, amidation sites and peptides bearing sequence identity to known bioactive peptides (conserved peptides). The accession numbers of the different species used these analyses are given separately with the neuropeptides. (i) Schistosomin- *A. californica*: NP_001191584; *L. stagnalis*: P24471; P. acuta: BW986160; *H. asinina*: AEW67133. (ii) Conopressin- L. stagnalis: Q00945; *A. kurodai*: BAB40371; *O. vulgaris*:BAC82436; *C. gigas*: EKC41686; *A. californica*:NP_001191416*; L. gigantea*:ESO90380. (iii) Egg-laying hormone-*A. californica*: AAA62580; *A. californica*_2: AAA27748; *L. stagnalis*: P06308; *A. parvula*: P17686; *A. parvula*_2: P17685; *A. dactylomela*: AC095731. (iv) Whitnin- *L. stagnalis*: AAF36485; *A. californica*: NP_001191586; *L. gigantea*: ESP04260; *H. asinina*: AFN20273. (v) Gonadotropin-releasing hormone-A. californica: NP_001191482; *O. vulgaris*:BAB86782; *U. edulis*:BAH09303; *C. gigas*: ADZ17180; *M. yessoensis*:BAH47639; *L. gigantea*: FC805607; *O. masou*: P30973; *H. sapiens*: AAX36880 and AAC02981; *S. mediterranea*: ADC84439. The scale bar indicates the number of amino acid substitutions per site.

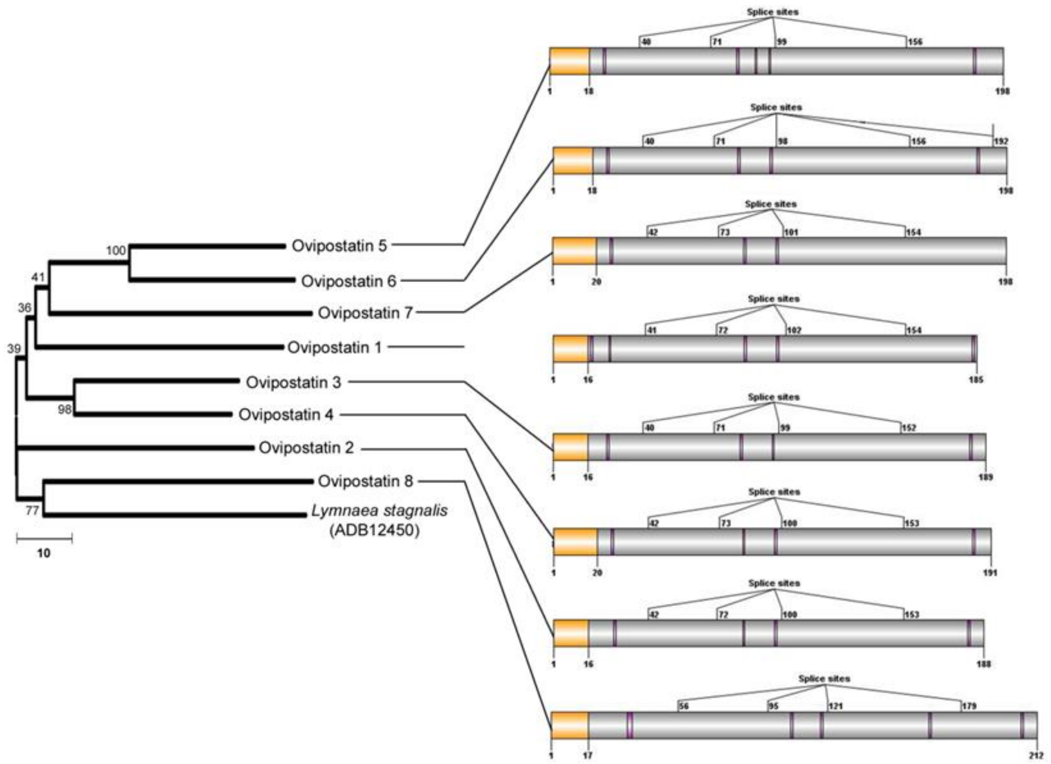

Supplementary Figure 69. Phylogenetic tree and schematic representation of Ovipostatins identified from the *B. glabrata* genome. See Supplementary Data 34 for details of *B. glabrata* ovipostatins 1-8

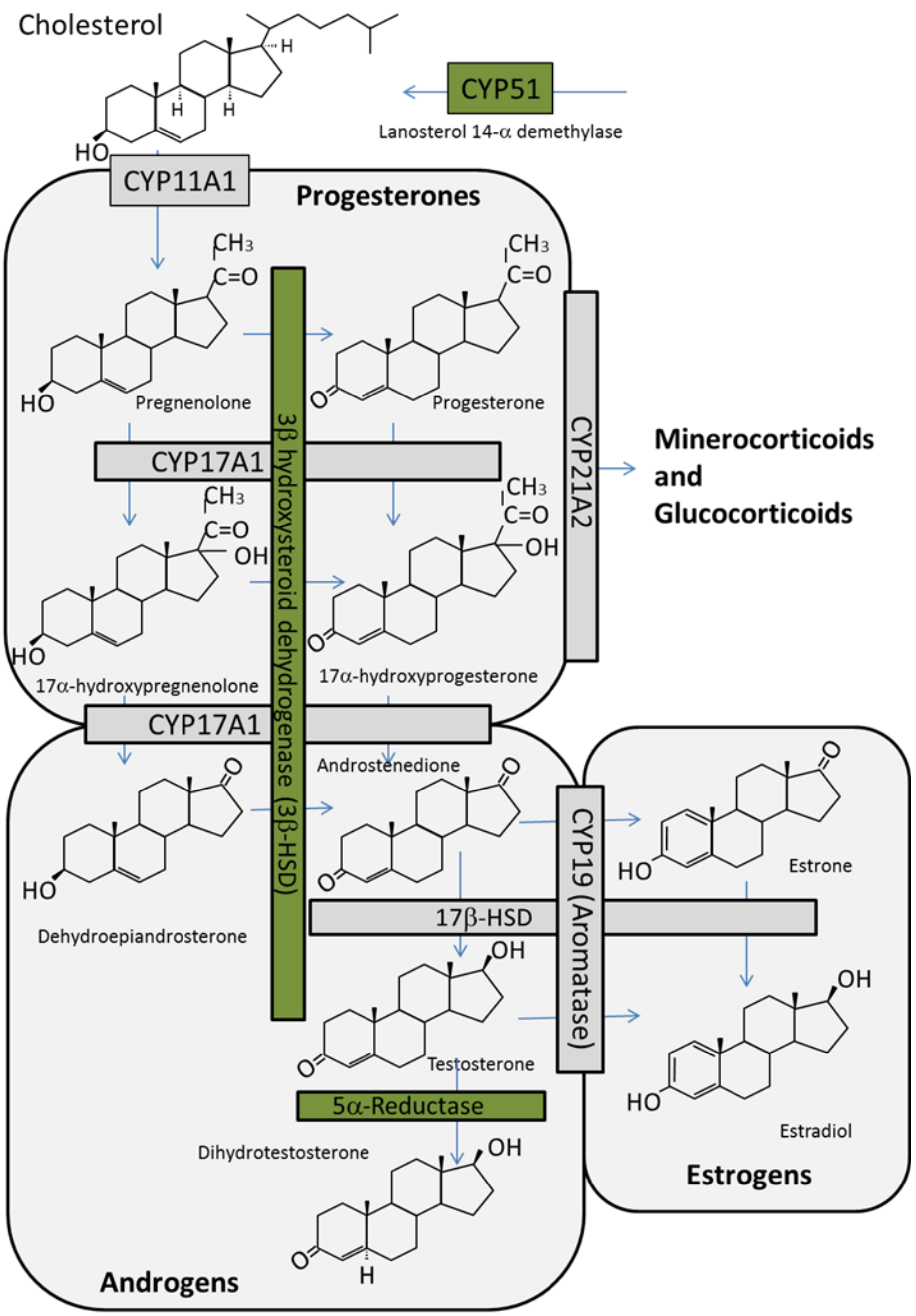

Supplementary Figure 70. Steroidogenesis. Arrows indicate enzyme catalyzed reactions. Boxes indicate genes involved in conversions. Genes identified from *B. glabrata* are boxed in green.

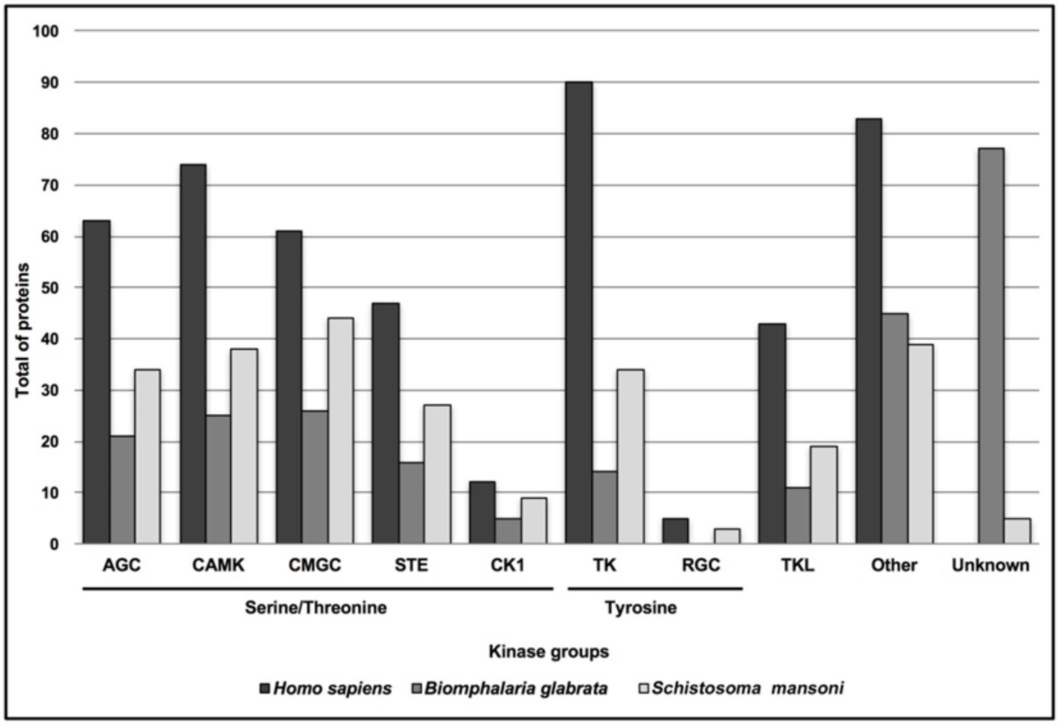

Supplementary Figure 71. Functional groups of eukaryotic protein kinases (ePKs) in *B. glabrata*, *S. mansoni* and *Homo sapiens*. The ePK groups include: AGC (cAMP-dependent protein kinase/protein kinase G/protein kinase C extended), CaMK (Calcium/Calmodulin regulated kinases), CMGC (Cyclin-dependent Kinases and other close relatives), STE (MAP Kinase cascade kinases), CK1 (Cell Kinase I), TK (Tyrosine Kinase), RGC (Receptor Guanylate Cyclases), TKL (Tyrosine Kinase Like), Other, and Unknown.

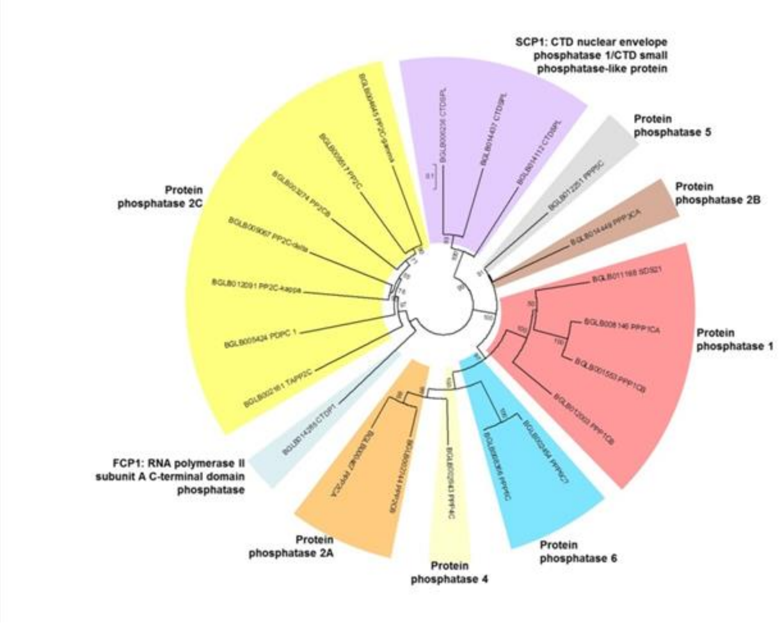

Supplementary Figure 72: Phylogenetic reconstruction of the protein serine/threonine phosphatases of *Biomphalaria glabrata*. The tree shown represents the best-supported phylogenetic analysis based on 500 bootstrap replicates with only those values >50 shown. The tree was constructed using neighbour-joining *p*-distance method. Nine discrete clades were identified were identified with a considerable amount of paraphyly shown throughout.

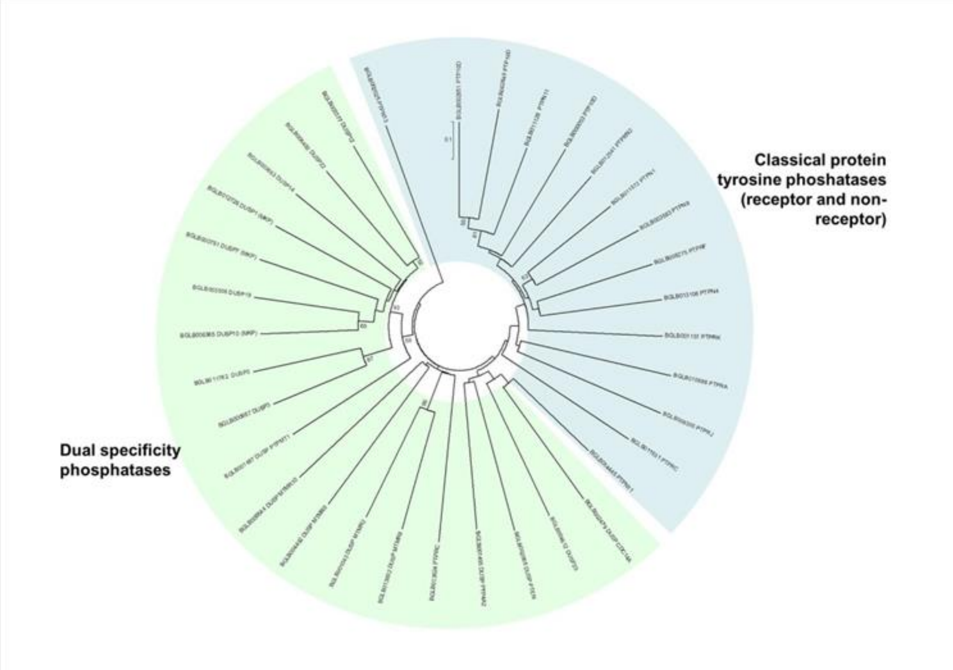

Supplementary Figure 73: Phylogenetic reconstruction of the protein tyrosine phosphatases (PTPs) of *Biomphalaria glabrata*. The tree shown represents the best-supported phylogenetic analysis based on 500 bootstrap replicates with only those values >50 shown. The tree was constructed using neighbour-joining *p*-distance method. This illustrates two discrete groups within the protein tyrosine phosphatases with the dual specificity phosphatases and the classical receptor/non-receptor type PTPs falling into discrete groups.

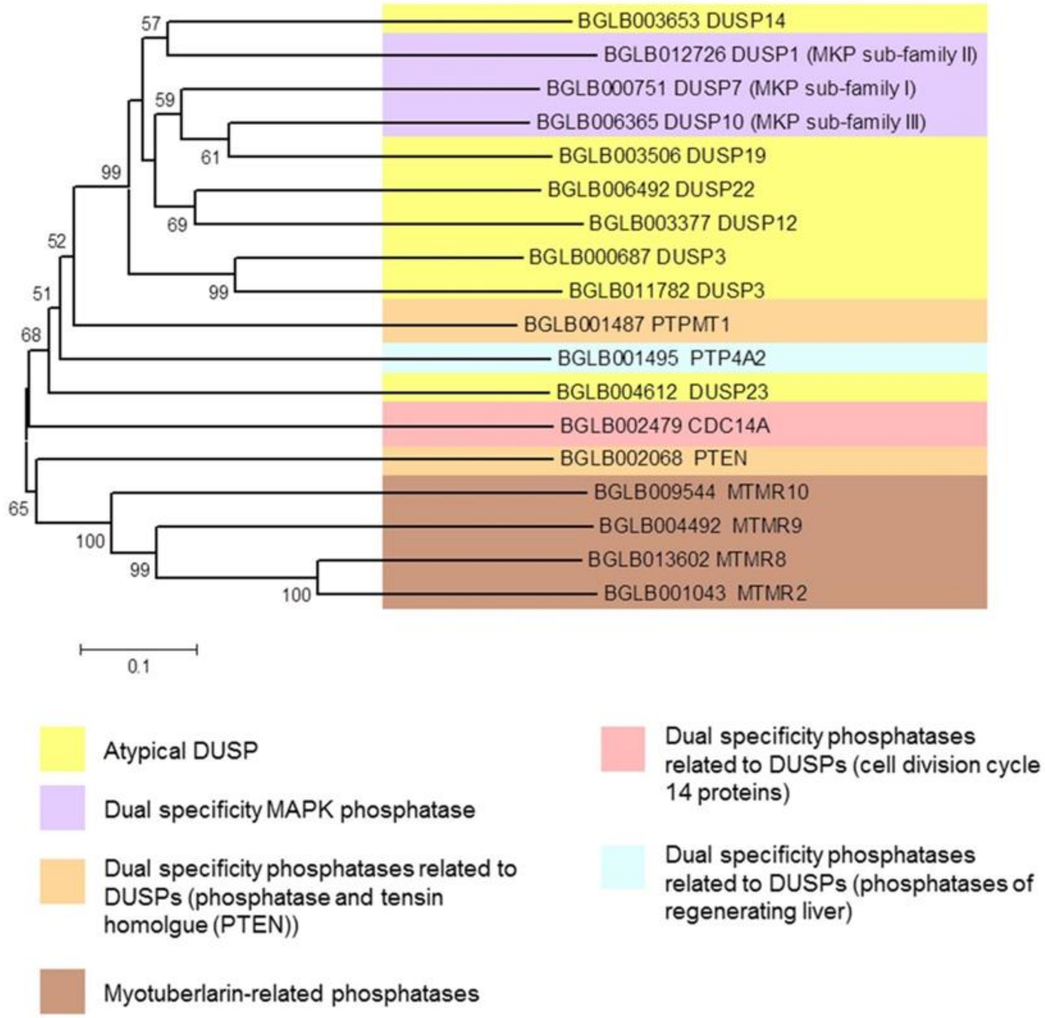

Supplementary Figure 74: Phylogenetic reconstruction of the dual specificity phosphatases (DUSPs) of *Biomphalaria glabrata*. The tree shown represents the best-supported phylogenetic analysis based on 500 bootstrap replicates with only those values >50 shown. The tree was constructed using neighbour-joining *p*-distance method. Considerable paraphyly was revealed in predicted function/classification across the DUSPs indicating the close relationships between these proteins.

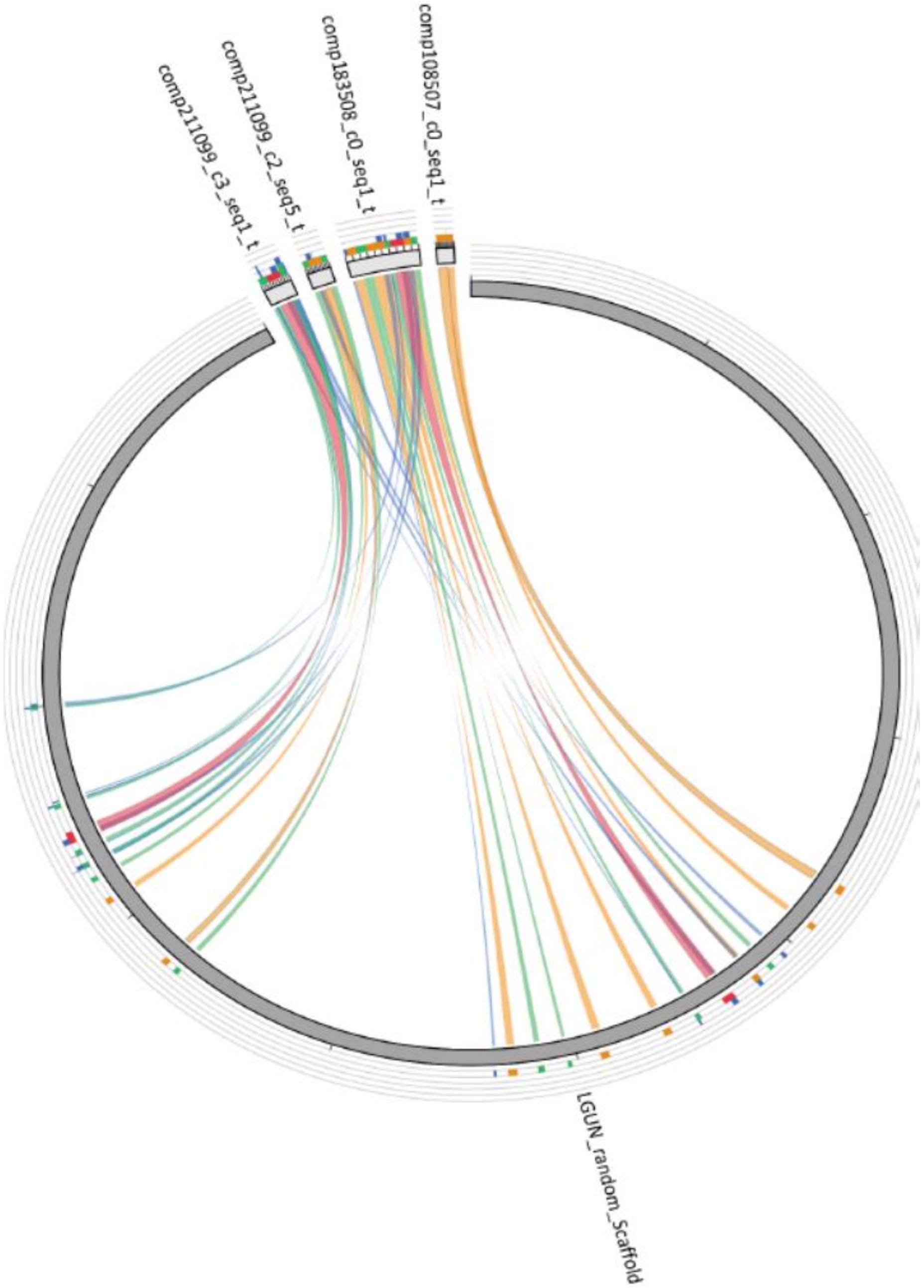

Supplementary Figure 75: Four tyrosinase transcripts map to LGUN_random_Scaffold2224. Circos diagram illustrating the genomic arrangement of 4 tyrosinase transcripts mapped to. Blue, Green, Orange and Red represent the four quartiles up to the maximum score of the local alignment with a score of 80% of the maximum score is red, while one with 20% of the maximum score is blue. Four arbitrarily named transcripts (top of the circus diagram), appear to derive from alternative splicing of exons on the scaffold.

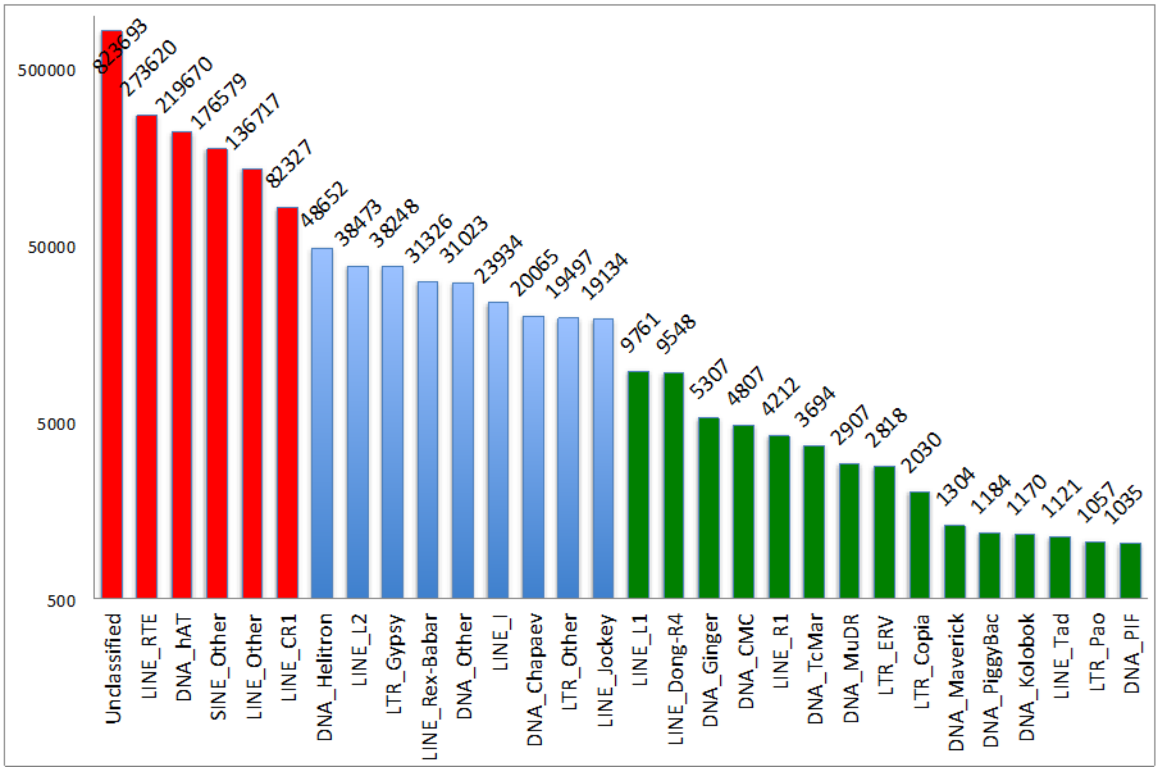

Supplementary Figure 76: Ranked high-copy TE abundance, as estimated with fragments numbers, within major classes of repeats summarized in Fig 6. Red >50K copies, blue 10-50K copies, green 1K-10K copies.

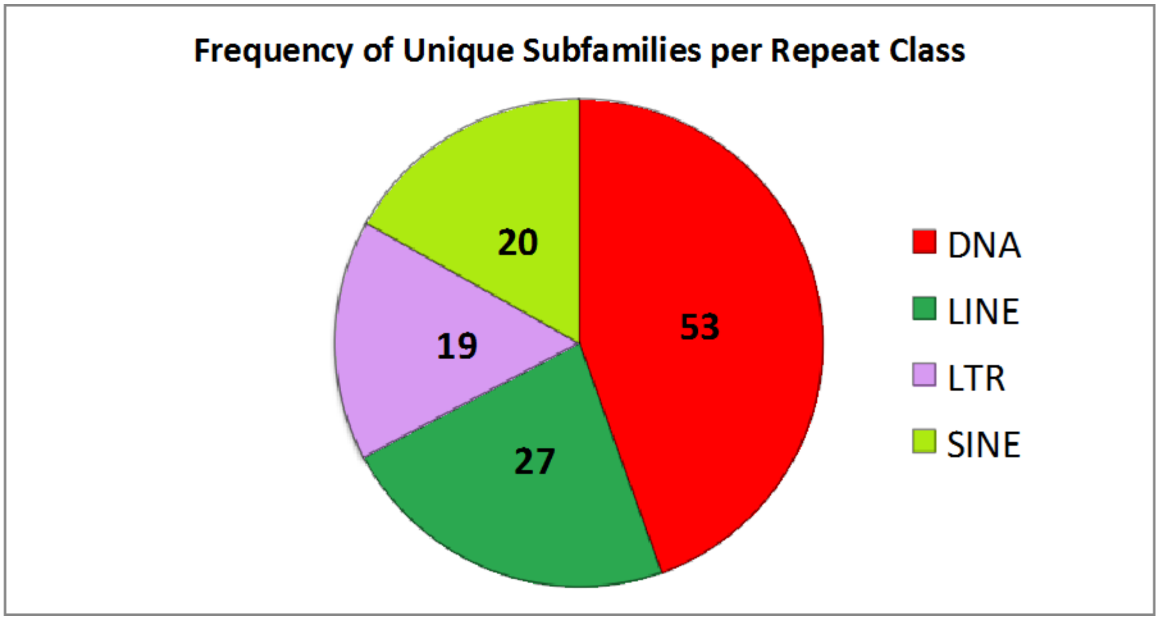

Supplementary Figure 77: Taxonomic diversity of interspersed repeats by major class indicated by colors in Fig. 1A. Diversity within each class is represented by the frequency of clade-specific subfamily annotation in a custom TE library and using RepeatMasker software.

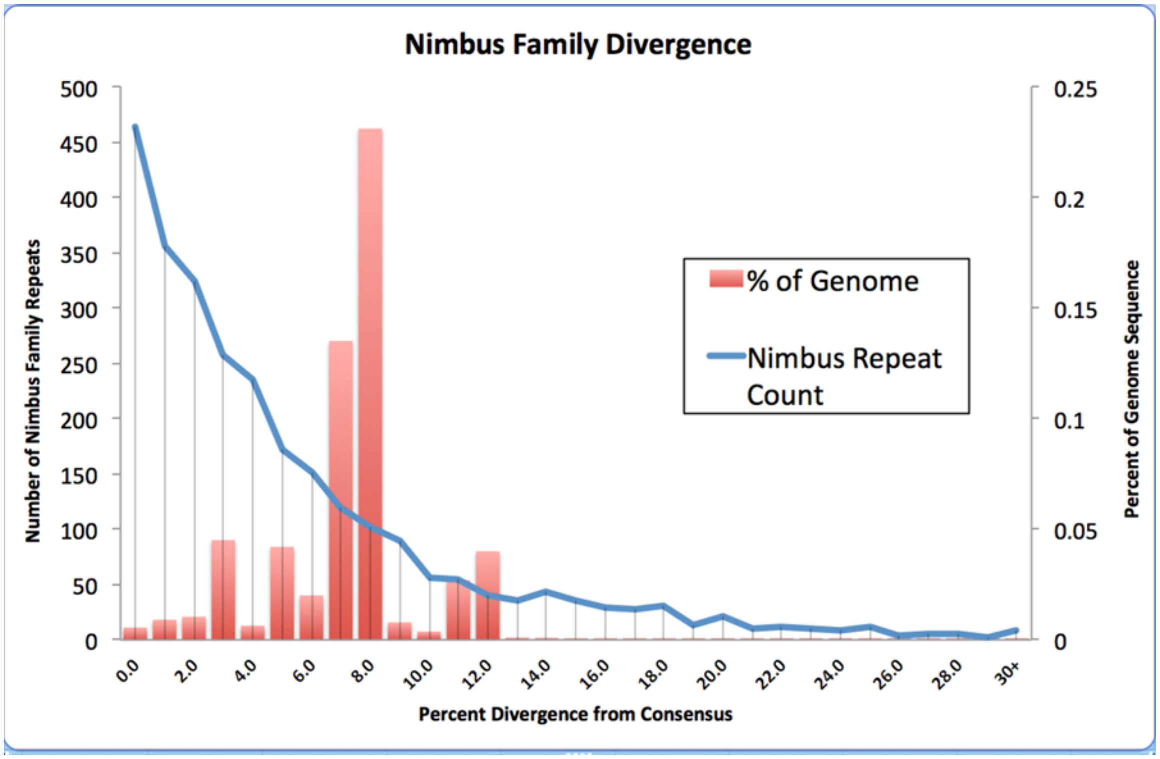

Supplementary Figure 78
Evolutionary profile of Nimbus LINE age reflected by the distribution of percent sequence divergence between individual elements and the consensus. Frequency per divergence category (left y axis) and percentage of genome sequence per category indicated by red bars (right y axis) are shown in function of the percentage of divergence to the consensus (by bins of 1%, first one being ≥0 and <1).

Supplementary Note 1. Karyotyping of the snail *Biomphalaria glabrata.*

A definitive karyotype of *Biomphalaria glabrata* has been problematic to attain due to the small size of its chromosomes, and the difficulties with obtaining quality metaphase spreads for cytogenetic analysis from cells containing large amounts of muco-polysaccharides which interfere with the spreading of chromosomes on microscope slides. The first reference in scientific literature regarding the *B. glabrata* karyotype employed the 'squash' technique to identify a haploid set of 18 chromosomes[1]. Several reports confirmed the presence of 36 chromosomes[2-4]. Raghunathan[5] obtained metaphase spreads from snail embryos and performed Giemsa staining to provide an improved karyotypic description for *B. glabrata* with the 18 pairs of chromosomes arranged into 6 groups, according to the morphology-based criteria of chromosome classification established by Levan *et al*. (1964)[6]. The establishment of a cell line from *Biomphalaria glabrata* named the Bge cell line (*B. glabrata* embryo)[7], and subsequent cytogenetic investigations also indicated a diploid set of 36 chromosomes[8]. It is of note that the genome of the Bge cell line is unstable; batches of Bge cell line and sublines from different laboratories showed karyotypic differences e.g. with 64 chromosomes in Bge1 and 67 in Bge2[9,10]. Thus, although these cells can be useful *in vitro* models, caution should be applied when they are used for genome studies. In 1984, Goldman *et al.*[11] isolated chromosomes from snail embryos and performed G-banding which allowed re-classification of the *B. glabrata* chromosomes into 9 groups. Finally, silver staining of *B. glabrata* chromosomes[12] revealed the nucleolar organizer regions and enabled analysis of the p and q arm ratios and centromere position. Inconsistencies in the karyotypic descriptions of *B. glabrata* produced previously are probably due to the different methodologies for preparing chromosome spreads, and inconsistencies in interpretation of the chromosome banding results. To resolve the above discrepancies, in aid of the genome sequencing effort[13] and supporting further studies of the snail genome[14-16] new protocols were developed to analyse *B. glabrata* chromosomes, and to provide the most detailed and accurate karyotype of *B. glabrata* to date, and the first ideogram for any snail chromosomes.

Chromosome preparation: We developed a fast, efficient and reproducible method to spread chromosomes from these snails, using BB02 strain *Biomphalaria glabrata*. This was achieved using the ovotestis of the snails, which contain good numbers of mitotic cells in addition to meiotic cells. Snails were exposed to 10μg/ml colcemid solution (3 hours, 27°C). The ovotestes were dissected and placed into 0.05M KCl and subsequently macerated. The sample was sieved to remove large tissue fragments and incubated in KCl for 20 more minutes. After centrifugation at 163g (5 minutes) cells were fixed in Carnoy fixative (methanol:acetic acid; 3:1 v/v). The cell suspension was dropped from approximately 1 meter onto damp glass microscope slides (Supplementary Figure 1).
Karyotyping through G-banding: The standard human G-banding technique was modified and adapted for the smaller and fragile snail chromosomes. Briefly, the chromosomes slides were incubated in 0.025% trypsin in 1X Earle's balanced salt solution (Invitrogen) for 30 seconds, incubated in Giemsa stain for 3 minutes (Karyomax, Invitrogen), plunged into 1% fetal bovine serum followed by a wash in 1X phosphate buffered saline and then dH_20. Chromosomes were mounted in DPX mountant for histology (Biotech Sciences Ltd) under 22 x 50 mm coverslips. The chromosome preparations were viewed with a Zeiss AxioImager Z2, imaged using a CoolCube 1m monochrome megapixel CCD camera and analysed with the Ikaros software (Metasystems). G-banding was performed on 30 good quality metaphase spreads, and the chromosomes on each spread were identified and grouped according to size, centromere position and banding pattern. Adobe Photoshop CS5 software was employed to magnify the karyotypes to measure the p and q arms. The measurements were converted to a percentage of the total haploid autosomal length (HAL) for each

cell. The HAL values were then averaged to construct an approximate scaled schematic representation for each chromosome. By conferring with principles outlined in the Human ISCN[17] each chromosome arm was divided into regions based on the prominent G-positive or G-negative bands. The landmark bands for each chromosome were then drawn to the schematic representations.

Fluorescence in situ hybridization: To map specific individual genes on to the chromosomes, to further allow their identification, we adapted the fluorescence in situ hybridization (FISH) protocol used for Bge cells[9]. Three *B. glabrata* BACs, containing seperately, the three genes *actin, ferritin* and *hsp70*[19] were used as FISH probes[18]. The BAC DNA was labelled with biotin-dUTP using a nick translation kit (Invitrogen). The BACs were co-precipitated with excess herring sperm DNA and *B. glabrata* genomic DNA, dried together and re-suspended in hybridization mix buffer containing 50% formamide, 2X SSC, 10% dextran sulphate and 1% Tween20 at pH7.0. The probes were denatured at 75°C for 5 minutes and repetitive sequences in the probes were allowed to anneal with repetitive elements in the genomic DNA for 2 hours. Before hybridization, the spread chromosomes were aged for 2 days at room temperature, dehydrated by passage through an ethanol series (70%, 90% and 100%) and denatured for 1.5 minutes at 70°C in 70% formamide, 2X SSC, pH7.0, followed by a further dehydration step using a further ethanol series. The probes were placed onto the denatured chromosomes to hybridize at 37°C for 4 days. The slides were washed (2X SSC, 3 minutes), placed in 1% bovine serum albumin in 2X SSC w/v) for 20 minutes and washed in 2X SSC for 3 minutes. Detection of the biotinylated probes was performed using cyanine 3 or fluorescein isothiocynate conjugated to streptavidin (Amersham Biosciences) (1:200 v/v) for 30 minutes at 37°C. The slides were then mounted in Vectashield containing 4’ 6 – diamidino-2-phenylindole (DAPI) (Vectalaboratories). The chromosomes and FISH signals were visualized using an AxioImager Z2, imaged using a CoolCube 1m monochrome megapixel CCD camera and Metafer software (Metasystems).

This study confirmed that the karyotype of *B. glabrata* is composed of 36 chromosomes. Based on size, centromere positioning and G-bands, the chromosomes were grouped into 18 pairs, 17 of which are homomorphic (homologues appear identical to each other) and 1 pair heteromorphic (homologues appear different from each other) (Supplementary Figure 2). Ideograms for the snail chromosomes were constructed (adhering to suggestions from Francke and Oliver[20]) thus providing a template for references purposes (Supplementary Figure 3). With the aim of identifying genomic ‘landmarks’ along the snail chromosomes which could be used eventually in addition to or as an alternative to G-banding for unequivocal chromosome identification and also in support of genomic studies; as a proof of principle we have mapped three specific genes by FISH. The *B. glabrata Actin* containing BAC was located on a group A chromosome which we identified as chromosome 5 (Supplementary Figure 3A). *Ferritin* (Supplementary Figure 3B) and *Hsp70* (Supplementary Figure 3C) containing BACs each mapped to different Group C chromosomes, as shown through dual-color FISH experiments (Supplementary Figure 3D).

This study provides, for the first time, a complete karyotype description for *B. glabrata*. The use of G-banding led us to identify a unique pattern within the p and q arms of each chromosome and construct for the first time an ideogram for *B. glabrata*. We also deployed gene-specific FISH mapping to establish further chromosomal landmarks to unequivocally link genetic and physical maps in this species. This development will open up the possibility of significantly advancing molluscan and comparative cytogenomic studies.

Supplementary Note 2. *Biomphalaria glabrata* linkage mapping.

For improved characterization of the *Biomphalaria glabrata* genome, linkage mapping data were used to assign scaffolds that resulted from *in silico* assembly to linkage groups.

Linkage mapping was performed with 13-16-R1 strain *B. glabrata* snails, maintained at Oregon State University, using two outbred unrelated parents and their 67 F1 offspring (NCBI BioProject Accession PRJNA288880), as well as snails from 19 inbred lines (NCBI BioProject Accession PRJNA270097). These snails were genotyped using RAD (Restriction site associated) markers[21] from *SbfI* digestion that were sequenced on the Illumina HiSeq 2000 at Oregon State University and aligned to *B. glabrata* genome assembly (Vectorbase: Assembly BglaB1, Genbank Accession Number APKA00000000.1) using BWA[22]. Raw sequence data have been uploaded to NCBI SRA (see BioProject Accessions above). Inbred snail line data are detailed in Tennessen *et al.* [23]. Both SNPs observed in the RAD tags and presence/absence of RAD tags were used as polymorphic markers. Mapping was done with OneMap[24] using a minimum LOD of 8. Initially, linkage maps were generated for each of the two parents in the cross. Then genotypes from the inbred lines were incorporated into these preliminary linkage maps to include additional markers that were not polymorphic in the initial cross. Each linkage group was given a name beginning with "LG." Linkage groups from the initial cross are named with LG and a number (e.g. LG1), while linkage groups formed entirely from inbred line data are named with a number and an "i" (e.g. LG1i). Linkage group numbers are arbitrary; e.g. there is no relationship between, LG1 and LG1i.

The linkage map of Parent 1 was used to assign scaffolds to linkage groups in the genome assembly. Any scaffold that appeared on exactly one linkage group was assigned to that linkage group. All linkage groups were classified as "random" meaning that we did not attempt to order scaffolds within linkage groups. For example, Scaffold10125 appears only on LG2, so its final designation in the genome assembly is LG2_random_Scaffold10125. Scaffolds with markers on more than one Parent 1 linkage group, as well as all scaffolds that did not have any markers in the Parent 1 linkage map, were designated as LGUN for "unknown" (e.g. LGUN_random_Scaffold16129). Most scaffolds were not represented in the linkage map and therefore are LGUN. (Also see http://www.ncbi.nlm.nih.gov/nuccore/APKA00000000.1)

The full linkage maps of both parents are presented in Supplementary Data 1: Bg linkage groups. For Parent 1 there are 118 linkage groups incorporating 1257 scaffolds and 1419 marker positions. For Parent 2 there are 128 linkage groups incorporating 1229 scaffolds and 1395 marker positions. The first column is either a number or the letter "I". If it is a number, it represents the map distance from the start of the linkage group in centimorgans, and it is followed by markers(s) which mapped to that site in the cross. If the first column is an "I", it is followed by markers from the inbred lines that are either in complete linkage disequilibrium (LD) with at least one of the markers in the previous line, or, in the case of linkage groups with an "i" in their name, are not in LD with any markers from the cross and constitute a unique linkage group. Marker names are the scaffold number, underscore, site rounded to 200,000bp. For example, "17.79 Scaffold4906_0" means that at a linkage map position of 17.79 cM, there is a marker on Scaffold4906 that is closer to physical position 0bp than to position 200,000bp (i.e. under 100,000).

Supplementary Note 3. RNAseq mapping, variant calling for annotated proteome and secretome

For improving our understanding of the biology of *B. glabrata* and for future exploration of potential interactions between *B. glabrata* as snail host and *S. mansoni* parasites, the RNAseq data obtained from 12 different BB02 *B. glabrata* snail organs/tissues (originating from 2 to 10 snails/sample) were mapped onto the genome assembly. In this way, RNASeq data revealed patterns of gene expression and aid gene modeling, as well as helped to derive sequence variation data by inspecting the pileup of RNAseq reads for synonymous and nonsynonymous variants along the expressed transcripts. The polymorphic genes were correlated to a diversity of metabolic pathways identified by KEGG analysis for the predicted proteome. Little is known about the diversity of secretome proteins overall, and regarding differential expression of secreted proteins from various *B. glabrata* tissues that could potentially interact with *S. mansoni* as these parasites develop within the snail host.This analysis was also performed for the subset of gene models that was predicted to represent secreted proteins. Secreted proteins are involved in vital biological processes such as cellular adhesion and migration, cell-cell communication, differentiation, proliferation and regulation of immune responses. Likely, these proteins are important for understanding host-parasite interactions. We predicted the whole set of secreted proteins, analyzed their diversity and annotated the putative secretome in terms of GO, Pfam domains and metabolic pathways. We clustered the secretome proteins according to tissue expression patterns.

Sequence mapping and variant calling: HiSeq Illumina mate-pair reads representing RNAseq data obtained from 12 different tissues of *B. glabrata* (see Supplementary Data 1) were screened to remove adaptors and primers (FASTX-toolkit, http://hannonlab.cshl.edu/fastx_toolkit/index.html). Reads were mapped onto the *B. glabrata* genome (Assembly BglaB1) available at VectorBase (https://www.vectorbase.org/organisms/biomphalaria-glabrata/bb02/bglab1) using TopHat2 (http://ccb.jhu.edu/software/tophat/index.shtml). Single Nucleotide Variations (SNVs) were identified using SAMtools mpileup pipeline (http://samtools.sourceforge.net/mpileup.shtml). SNVs were filtered according to the following criteria: (1) low quality variants were discarded (PHRED <20); (2) variants with a minimum rate of two equal reads covering the least frequent allele and nucleotide positions with more than two alleles were removed; (3) variants with too low or too high read depths were not considered, although variants in the interval between the minimum of four reads of depth and the maximum three standard deviations above the mean were kept; (4) SNVs within 5 bp of each other were discarded[25]. The set of selected SNVs were then annotated using SnpEff[26]. The final list of nonsynonymous SNVs were screened to identify variants in metabolic pathways (SIFT scores <0.05), due to the potential function of this class of variants. Gene pathway networks analysis were performed using the bi-directional best-hit (BBH) method in the KEGG Automatic Annotation Server (KAAS)[27]. Approximately 252 million Illumina raw reads from 12 different tissues of several individual BB02 *B. glabrata* snails were screened for the presence of adaptors and primer sequences as well as for low-quality sequences. More than 97% of the raw reads passed the filters, with the exception of the set of reads originated from albumen gland (AG) from which more than 15% of the reads were removed. Reads that lost their corresponding mate were still used, but mapped as fragment reads. After filtering, 55-60% of the reads from each tissue were mapped to genome and approximately 30-40% were concordant pair alignments. Mapping files were grouped in order to increase statistical support for variant calls. A total of 382,543 putative SNVs were discovered in the genome assembly. From the entire set, 85,790 SNVs occurred in 9,030 genes, corresponding to 60.5% of the predicted genes. Seventy-three percent of these genes had fewer than

10 SNVs per gene. The number of genes with 11-30 SNVs per gene was 1,843 and there were 516 genes contained more than 30 SNVs per gene (Supplementary Figure 4). Next, SnpEff was used to annotate all the SNVs and their potential mutational effects on associated genes. This analysis showed that 12,758 nonsynonymous SNVs (16.32%) in 5,397 genes.

Proteome annotation: The *in silico* prediction of proteins was performed using several bioinformatics tools and databases. InterProScan was used to assign domains to the predicted proteome of *B. glabrata*[27]. The output of InterProScan that integrated the following protein signature databases: BlastProDom, FPrintScan, HMM-PIR, HMM-Pfam, HMM-Smart, HMM-Tigr, ProfileScan, Pattern Scan, Superfamily, Gene3D and HMM-Panther was used to construct a relational database in Structured Query Language (SQL) used for querying and extracting information from the analysis using the graphical user interface DbVisualizer (http://dbvis.com/). Functional annotation using GO terms was also obtained using InterProScan[29]. Metabolic information was retrieved from KEGG and BRITE using the KAAS tool[27] with BBH (bi-directional best hit) assignment method and using eukaryotes as "representative set". Protein domain information was retrieved from InterProScan[28]. Secreted proteins were clustered based on RNAseq expression patterns, normalized by RPKM, according to all 12 sequenced tissues. Hierarchical Clustering was performed using the GenePattern web-base platform[30]. Clustering was performed with Euclidean distance measure and pairwise average-linkage. The same strategy was implemented for the both proteome and secretome, full results can be downloaded at: http://headnode.cebio.org/download/KAAS/B_glabrata/**.** The proteome was annotated using InterProscan (Supplementary Data 3). Information regarding the metabolic pathways supported by the genes from *B .glabrata* was obtained using the KEGG database. From a total of 5,368 expressed genes assigned to one or more KO categories, 1,825 contained nonsynonymous SNVs. These genes were mapped to 32 biochemical pathways of five top-level KO categories (Supplementary Data 4 and Supplementary Figure 5). The KO second-level categories with the highest number of polymorphic genes were membrane transport and metabolism of cofactors and vitamins categories with 56.5% and 51% of genes containing nonsynonymous SNVs, respectively. Second-level KO categories with the lowest number of polymorphic genes were immune system and nervous system with 21.2% and 23.3% of the genes with nonsynonymous SNVs, respectively. Regarding the number of nonsynonymous SNVs per gene, the genes related to the replication and repair categories showed the highest ratio (an average of 3 SNVs per gene) and the lowest ratio was observed for genes related to the overview category (an average of 1.7 SNVs per gene). Additionally, out of the 583 genes coding for the specific group of secreted proteins, 348 (60%) contained 2,232 SNVs. Moreover, from the set of 240 putative kinase genes, 200 (83%) present a total of 2,300 SNVs.

Secretome prediction: The *in silico* prediction of secreted proteins was performed using different bioinformatics tools and databases. SignalP 4.1[31] was used for identifying classical secretory proteins. All proteins identified as not having a signal peptide were analyzed with SecretomeP[32] for predicting non-classical secreted proteins. In this case, records with NN score ≥ 0.9 were considered as secreted proteins. Proteins predicted to be secreted were subsequently scanned for the presence of mitochondrial sequences by TargetP[33] and transmembrane helices by TMHMM[34]. We identified 583 secreted proteins corresponding to 4.1% of the *B. glabrata* proteome (Supplementary Data 5,6), 484 (3.4%) proteins possess a classical N-terminal signal peptide, 391 (2.7%) were identified as non-classically secreted. A total of 281 proteins were predicted to be anchored to the membrane by transmembrane domains. Additional of 11 proteins targeted to the mitochondria were removed from the analysis. Of these 583 genes coding for the specific group of secreted proteins, 348 (60%) contained 2,232 SNVs. Annotation was possible for 474 proteins (81% of the secretome) (Supplementary Data 7). The *B. glabrata* secretome included different functional

classes related with different process like digestive enzymes, protease inhibitors, hormones, lectins, kinases and antioxidant enzymes. We observed 238 Pfam domains[35] (Supplementary Data 8). Among the most abundant domains we found was the Proteinase inhibitor I15, antistatin-like domain (PF02822), the proteins that presented this domain are related with serine-type peptidase inhibitor activity (G0:0004867). The second most abundant domain present in the predicted secretome was the zinc-finger double domain (PF13465). Other domains frequently observed were peptidases such as peptidase 10 (PF00450), peptidase C1A (PF00112), peptidase S1 (PF00089). The Pfam domain analysis showed the diversity of this subproteome (secretome). The same diversity was obtained in the GO analysis (Supplementary Figure 6).

Proteins containing EGF domains (PF07645, PF00053, PF12661) play a role in extracellular protein-protein interactions such as adhesion, coagulation, and receptor ligand interactions. EGF modules have been described also to function in innate immunity. The hemolymph of *B. glabrata* contains lectins, represented in our secretome prediction as the PF00059 Pfam domain. These proteins have been demonstrated that precipitate polypeptides secreted by the trematode *Echinostoma paraensei* [36] and to be a crucial step to non-self recognition and a key defense molecule.

Secreted proteins were mapped onto KEGG pathways revealing a great deal of functional diversity. Most secreted proteins belonged to categories as carbohydrate metabolism (n=17), glycan biosynthesis (n=10), folding sorting and degradation (n=10), signal transduction (n=20) and transport and catabolism (n=18). According to BRITE there are serine peptidases (n=11), cysteine peptidases (n=5) and C-type lectins (n=3) present in the snail secretome. These results indicated that some metabolic pathways might be important the snail-trematode interaction. Further inspection of the peptidases predicted 163 peptidases belonging to five major classes such as aspartic (n=6), cysteine (n=48), metallo (n=57), serine (n=34) and threonine (n=17) and one peptidase of unknown catalytic type. The range of receptors, transporters or channel proteins identified are interesting because some compounds with molluscicidal activity bind such proteins[37]. Our predictions yielded 50 G protein-coupled receptors (GPCRs), 22 nuclear receptors and 56 channel proteins (including 21 voltage-gated and 12 related to voltage-gated cation channels). We also identified 122 transporters of which the most abundant were: 23 were mitochondrial carriers, 9 nucleoside sugar transporter and 8 were metal ion transporter.

In order to have a dynamic view of the secretome, all the RNAseq data for the 12 tissues were clustered according to the pattern of protein expression (Supplementary Figure 8). We found a similar expression pattern in foot and salivary glands and a different expression pattern in the ovotestis. Expression of secreted proteins at different sites could point to those that may be directly involved in snail-parasite interactions. Further study of the secretome may indicate relevant aspects of snail-parasite interactions that determine whether *B .glabrata* will defeat the infection or ultimately transmit schistosomiasis.

Computational annotation of the *B. glabrata* proteome provides a catalogue of gene functions and metabolic processes that function in aspects of the general biology of this snail, also in relation to the role of *B. glabrata* as intermediate host for parasites such as *S. mansoni*. Secreted proteins are important to understand parasite-host interactions and are involved in vital biological processes such as cellular adhesion, cell-cell communication, differentiation and proliferation. In order to have a dynamic view of the secretome, all the RNAseq data for the 12 tissues were clustered according to the pattern of protein expression (Supplementary Figure 8). Expression of particular (secreted) proteins at different anatomical sites could point to those that may be involved in snail-parasite interactions. Further study, especially of the secretome could indicate relevant aspects of snail-parasite interactions that determine whether *B .glabrata* will defeat the infection or ultimately

transmit schistosomiasis. Additional studies should be conducted to investigate if SNVs accumulation is related with specific characteristics of *B. glabrata*, and how these contribute to individually diverse response capabilities of *B. glabrata* snails to their environment.

There is a scarcity of data on SNVs in *B. glabrata* or any evolutionarily related species in the literature. One of the few studies using RNAseq to identify polymorphic sequences showed a high divergence of nonsynonymous SNPs between alleles of several genes with probable immunological roles[38], whereas this study indicates the opposite. This may be due to use of distinct *B. glabrata* isolates (BB02 versus field collected snails from Guadeloupe) and different analytic approaches (analysis of the full proteome versus restriction-site associated DNA (RAD) markers and specific loci).

The results presented here provide a broad overview of the distribution of *B. glabrata* SNVs and can be used to guide further research, for example, comparing SNVs in snail populations sensitive or resistant to molluscicides or pathogens such as *S. mansoni*.

Supplementary Note 4. Tissue-specific expression patterns (RPKM) in *B. glabrata*.

The following approach was taken as a means of evaluating gene expression among and within the tissues of *B. glabrata*, using the RNAseq data (paired ends) collected from a total of 12 tissues dissected from BB02 *B. glabrata* as part of the genome characterization project for analysis against the gene models predicted by Maker from the *B. glabrata* genome assembly.

Reads Per Kilobase per Million (RPKM) values provide a means for quantifying gene expression from RNAseq data by normalizing for total read length and the number of sequencing reads (see[39]). RNAseq (Illumina paired-end reads (see Methods, main paper; Supplementary Data 1 for Genbank accession numbers) were available from twelve different tissues/organs of adult BB02 *B. glabrata* (between 2 and 10 snails per sample to obtain sufficient RNA amounts): albumen gland (AG); buccal mass (BUC); central nervous system (CNS); digestive gland/hepatopancreas (DG/HP); muscular part of the headfoot (FOOT); heart including amebocyte producing organ (HAPO); kidney (KID); mantle edge (MAN); ovotestis (OVO); salivary gland (SAL); stomach (STO); terminal genitalia (TRG).

RPKM values were calculated for all the transcriptome data for all tissues using RNA-Seq analysis software from CLCBio (Qiagen, Waltham, MA) as in Young *et al*.[40]. Paired-end reads were imported into CLCBio, and trimmed using NGS coretools Trim reads option (primers and adaptors, as described in Supplementary **Note** 3). We only used reads that were above the quality limit (0.05) and that were 35 bps or longer. Both paired reads and "orphan" reads from broken pairs were run together in the analysis. Using the RNA-seq analysis option on CLC Bio, our selection parameters were as follows: Maximum number of hits for a read = 10; Strand specific = Both; Count paired reads as two = Yes; Create list of unmapped reads = Yes; Create report = Yes; Create fusion gene table = No; Expression value = RPKM; Calculate RPKM for genes without transcripts = Yes; Reference type = One reference sequence per transcript; Global alignment = No; Auto-detect paired distances = Yes; Similarity fraction = 0.9; Length fraction = 0.8; Mismatch cost = 2; Insertion cost = 3; Deletion cost = 3; Reference sequence was *Biomphalaria glabrata* _RNA_Gene_Mode

RPKM = $(10^9 \times C)/(N \times L)$ – (see details in Mortazavi *et al.* [39]) with
C = Number of reads mapped to a gene (or coding sequence [CDS] read count)
N = Total mapped reads in the experiment (total mapped read count)
L = gene length in base-pairs for a gene

Hierarchical clustering was performed using *Spotfire* (TIBCO Software Inc.), based on RPKM values. The algorithm used was a hierarchical agglomerative method (http://spotfire.tibco.com/; as in [41]). UPGMA (unweighted pair-group method of averages) clustering was used to calculate the row and column dendrograms together with euclidiean distance measure, and ordering weight was an averaged value. All transcriptome data was analyzed against the *Biomphalaria* gene models.

Comparison of RPKM values for individual transcripts across tissues reveals genes that are highly and differentially expressed in particular tissues, see Supplementary Data 9 and Supplemenary Figures 8,9.

Supplementary Note 5. Proteomic characterization of released proteins.

We collected *B. glabrata* proteins from snail conditioned water (SCW) and following electrostimulation (ES), which induces rapid release of proteins. NanoHPLC-MS/MS was used for characterization of these proteins.

Snails, collection of released proteins (mucus) of B. glabrata: BB02 strain *B. glabrata* were housed at QIMR Berghofer (Brisbane) and maintained in flow-through aquarium tanks in a constant temperature room set to 25C, and fed to satiety on lettuce. Snail conditioned water (SCW) - several batches of 20 snails were collected from different aquaria, washed with pH neutral distilled water, and kept in approximately 25 ml water for 3 h under 28C. Snails were then returned to their original aquaria, while 25 ml methanol was added into each beaker of SCW and mixed thoroughly, followed filtering through a 0.45 µm PVDF Millex-HV syringe filter to remove particles and microbial. The filtrate was snap frozen and lyophilised. Electro-stimulation (ES) – Snails were placed individually into a petri-dish containing a small amount of water to partly cover the shell. Two electrodes connected to a 3 volt battery were used to stimulate the snail by touching water each side of the snail for 2 s, 20 times at 1 s intervals. Water was then collected using a pipette, and transferred to 20% methanol. The final products were combined from 18 snails and the solution was filtered (0.45 µm PVDF filter) then lyophilised.

SDS-PAGE electrophoresis and trypsin digestion: The lysate proteins of larvae were size fractionated by 1D sodium dodecyl sulphate-polyacrylamide gel electrophoresis (SDS-PAGE) with a 4 – 12% polyacrylamide gradient gel (Amersham ECL Gel, GE Healthcare Life Sciences) according to manufacturer's instructions. The gel was stained with Coomassie Brilliant Blue R250 (Sigma-Aldrich). Lanes containing protein were sliced into 51 pieces and processed for LC-MS/MS. Briefly, proteins within each gel piece were subjected to reduction (10 mM DTT, 45 min at 56C) and alkylation (IAA, 30 min, room temperature, in the dark) followed by Sequencing Grade Modified Trypsin (Promega) for 16 h at 37C. Peptides were then extracted from the gel pieces as described and desalted as well as concentrated by Zip-tip C18 (Millipore).

NanoHPLC-ESI-TripleTOF Analysis: The extracts were analysed by LC-MS/MS on a Shimadzu Prominance Nano HPLC (Japan) coupled to a Triple Tof 5600 mass spectrometer (ABSCIEX, Canada) equipped with a nano electrospray ion source. Six µl of each extract was injected onto a 50mm x 300 µm C18 trap column (Agilent Technologies, Australia) at 30µl/min. The samples were de-salted on the trap column for 5 minutes using 0.1% formic acid (aq) at 30µl/min. The trap column was then placed in-line with the analytical nano HPLC column, a 150mm x 75µm 300SBC18, 3.5µm (Agilent Technologies, Australia) for mass spectrometry analysis. Linear gradients of 1-40% solvent B over 35 min at 300nL/minute flow rate, followed by a steeper gradient from 40% to 80% solvent B in 5 min were used for peptide elution. Solvent B was held at 80% for 5 min for washing the column and returned to 1% solvent B for equilibration prior to the next sample injection. Solvent A consisted of 0.1% formic acid (aq) and solvent B contained 90/10 acetonitrile/0.1% formic acid (aq). The ionspray voltage was set to 2400V, declustering potential (DP) 100V, curtain gas flow 25, nebuliser gas 1 (GS1) 12 and interface heater at 150C. The mass spectrometer acquired 500ms full scan TOF-MS data followed by 20 by 50ms full scan product ion data in an Information Dependant Acquisition, IDA, mode. Full scan TOFMS data was acquired over the mass range 350-1800 and for product ion ms/ms 100-1800. Ions observed in the TOF-MS scan exceeding a threshold of 100 counts and a charge state of +2 to +5 were set to trigger the acquisition of product ion, ms/ms spectra of the resultant 20 most intense ions. The data was acquired and processed using Analyst TF 1.5.1 software (ABSCIEX, Canada).

Protein identification: An in-house protein sequence database was constructed based on the twelve transcriptomes of *B. glabrata*. A composite target–decoy database was built with the forward and reverse sequences for calculating the FDR. Proteins were identified by database searching using PEAKS v6.0 (Bioinformatics Solutions Inc., Waterloo, ON, Canada) against the protein database. Search parameters were as follows: none digestion, variable modifications included amidation, methionine oxidation, conversion of glutamine to pyroglutamic acid, and deamidation of asparagine. Precursor mass error tolerance was set to 20 ppm and a fragment ion mass error tolerance was set to 0.05 Da. Maximum expectation value for accepting individual peptide ion scores [-10*Log(p)] was set to ≤0.01, where p is the probability that the observed match is a random event.

Prediction of secreted proteins: N-terminal signal sequences were predicted using the SignalP 4.1[31], Predisi[42] and TMHMM[34]. For SignalP predictions, positive identifications were made when both neural network and hidden Markov model algorithms gave coincident estimations. Herein, a protein was designated as secreted, only when it met the criteria of both SignalP and Predisi, and did not have a transmembrane domain predicted by TMHMM. To avoid false predictions, we used all protein sequences instead of choosing a representative sequence in the protein groups for secretion prediction. This condition was set because more than one-third of the identifications were of protein groups rather than single proteins. Proteins within the same protein group were expected to have similar characteristics, as these proteins are orthologs, paralogs, or members of the same protein family. Thus, if one or more protein sequences from a given protein group were predicted as secreted protein(s) then this protein group was annotated as secreted.

The proteins released by *B. glabrata* into the SCW and the EW samples that were identified by NanoHPLC-MS/MS are listed in Supplementary Data 10), Detection of an ortholog of temptin, a pheromone of *Aplysia*, suggests an operational pheromone sensory system in *B. glabrata*.

Supplementary Note 6. GPCRs, putative receptors for aquatic odor perception.

Aquatic animals must perceive a variety of water-soluble chemicals. Odor perception is initiated at the olfactory organs, where odorants are detected by specific chemosensory receptors, with G protein coupled receptors (GPCR) identified previously as putative molluscan chemoreceptors[43]. To gain further insight into the molecular mechanisms underlying odor perception in aquatic environments, we analyzed the *B.glabrata* genome for GPCR-like gene superfamily sequences.

To identify target sequences, the *B. glabrata* genome and gene model (CDS) databases were imported form Vectorbase into the CLC Genomics Workbench (v6.0; Finlandsgade, Dk). Previously identified molluscan putative chemoreceptor GPCR[44] were then used to query (TBLASTN and BLASTX) the databases. Open reading frames retrieved from the databases were translated and screened for the presence of recurrent transmembrane motifs using TMHMM. Multiple sequence alignments were created with the Molecular Evolutionary Genetics Analysis (MEGA) software version 5.1. Sequence presentation and shading of multiple sequence alignments was performed using the LaTEX TEXtopo package. Phylogenetic trees were constructed based on the amino sequences of the candidate olfaction genes and the collected data sets. Amino acid sequences were aligned using ClustalW2. Unrooted trees were constructed by the neighbor-joining method, with Poisson correction of distances, as implemented in MEGA5.1 software. Node support was assessed using a bootstrap procedure base on 1000 replicates. BB02 strain *B. glabrata* were housed at QIMR Berghofer (Brisbane) and maintained in flow-through aquarium tanks in a constant temperature room set to 25C, and fed to satiety on lettuce.

Tentacles were fixed with glutaraldehyde in phosphate buffer at 4°C overnight. Secondary fixation was carried out by immersion in 1% osmium tetroxide in sodium cacodylate, dehydration in a graded series of ethanol (20% to 100%), drying ina critical point drying machine, using liquid CO2 as a transitional medium and platinum-coating using an Eiko IB-5 Sputter Coater. The specimens were viewed using a Jeol 6300 field emission SEM (USC).

B. glabrata tentacles were homogenized in TriZol reagent (Invitrogen) and total RNA extractions were performed following the manufacturer's instructions. cDNA was synthesized using a first-strand cDNA Synthesis Kit (Fermentas) and used as a template in PCR with BgCR509a-specific primers (available upon request). A *B. glabrata* actin gene was used as control. PCR (REDTaq DNA polymerase, Sigma) was performed under the following conditions: 94℃ for 1 min, 36 cycles of 94℃ for 1 min, 45℃ for 2 min; 7℃ for 1 min, and 72℃ for 10 min. Amplicons were separated on a 2% agarose gel, and visualized using ethidium bromide.

Sense and antisense RNA probes will be prepared by *in vivo* transcription from BgCR509a-pGEM-T (Promega) cloned fragments using a digoxygenin labeling kit (Roche Molecular Biochemicals) with SP6 polymerase for antisense probes and T7 polymerase for sense riboprobes. *B. glabrata* tentacles were fixed in 4% paraformaldehyde and prepared for paraffin sectioning at 8 mm. Sections were de-paraffinized, rehydrated and probed as described in Cummins et al.[44]. We identified 241 seven transmembrane domain GPCR-like genes that belong to 14 subfamilies and cluster in the genome (Fig. 1, Supplementary Figs. 9,10, Supplementary Data 11). Through RT-PCR and *in situ* hybridization analysis we also confirmed that at least some of these genes are expressed within the animal's anterior tentacle (Fig. 1). In summary, we have identified putative chemoreceptor genes that are expressed within the olfactory organ sensory epithelia of *B. glabrata,* demonstrating their potential importance in odorant perception.

Supplementary Note 7. Genome of a mycoplasma or related mollicute associated with *Biomphalaria glabrata.*

Commensals have been found in both wild and laboratory strains of the snail *B. glabrata*, and a possibly complete genome sequence of a commensal was found in the snail genome assembly. These results potentially open up a dimension of the snail's biology that has currently received little attention.

For identification of the commensal whole genome, the NCBI nr database was searched using BLAST with all sequences from the *B. glabrata* genome assembly version 4.3. Using in-house python scripts, the results were filtered using a minimum BLAST bitscore of 50, the gene identifiers in the BLAST results were mapped against the NCBI taxonomy database, and finally *B. glabrata* scaffolds were sorted into taxonomic divisions (corresponding to bacteria, invertebrates etc) and then species within those divisions. The total amount of sequence (and the corresponding scaffolds) was allocated to each species. In this way a 647,978 base pair scaffold with only bacterial BLAST hits was identified (LGUN_random_Scaffold191). It comprises four contigs of lengths 526.5 kb, 65.4 kb, 52.6 kb and 3.4 kb. The ends of this scaffolds contained 163 bp of overlapping sequence, which is consistent with a circular prokaryotic genome. No other scaffolds or unplaced small contigs gave BLAST hits to organisms with "plasma" in their name (with a bitscore ≥ 200) – thus it is probable that the majority of the commensal genome has been assembled within this single 648 kb scaffold. This is well within the expected size range of the mycoplasmas, with *Mycoplasma genitalium* having a genome of 589 kb. Mycoplasma-like organisms have been known to infect a range of bivalves and crustaceans[46] and have previously been found in the metabolically active microbial community of the gastrointestinal tract of the Giant African snail, *Achatina fulica*[47]. Performing a BLAST of the commensal 16S sequence taken from the scaffold against the NCBI 16S ribosomal RNA sequences, identified it as an unknown mollicute, with 82% to 83% identity to a number of spiroplasmas, mesoplasmas and mycoplasmas. The highest hit was to *Spiroplasma lampyridicola* strain PUP-1 (isolated from a firefly beetle) with an identity of 83% and total score of 1249. We next verified the presence of this commensal in other individual snails derived from the same colony of the BB02 strain used for *B. glabrata* genome sequencing as well as checking whether similar commensals could be found in two different laboratory snail colonies. Here, *B. glabrata* gDNA was derived from the BB02 colony at the University of New Mexico, an Aberystwyth pigmented hybrid colony, or an Aberystwyth albino NMRI colony, and screened for the presence of potential commensal bacteria using a bacteria-specific 16S primer set (8F: 5′-AGAGTTTGATCCTGGCTCAG-'3; 1492R: 5′-GGTTACCTTGTTACGACTT-'3). A single 16S mycoplasma sequence with 98% similarity to the genome commensal (over 274 base-pairs) was identified from the BB02 snails while three different 16S mycoplasma sequences, significantly different to the genome commensal sequence, but with 98% similarity to other known mycoplasmas, were identified from the Aberystwyth pigmented hybrid snail colony and NMRI albino colony.

A phylogenetic tree (NJ) was built using MEGA6[48], see Supplementary Figure 10. It is constructed with the 16S sequences of the commensals, and also 16S sequences sampled from the top BLAST hits to the genome commensal sequence and our clones. The tree shows the two BB02 sequences, labeled (a) and (b) in a long separate branch, and the three Aberystwyth sequences (c) clustering together with a number of known mycoplasmas.

We analyzed the RNA-Seq reads that were obtained from 12 different tissues of *B. glabrata*. Our analysis of the RNA-Seq data indicated the presence of small transcripts in all tissues that map to the commensal genome. Whilst this analysis should be viewed with a high degree of caution, it does provide some evidence that the commensal could be present in the hemolymph. Most of the

RNA signal from the commensal is expected to have been lost for two reasons: (a) unlike eukaryotic RNA, prokaryotic RNA is generally not polyadenylated, apart from some small mRNAs, and thus it does not have the ploy(A) tail – the poly(A) tail is essential for the RNA to be picked up by standard eukaryotic sequencing protocols; and (b) without the poly(A) tail prokaryotic RNA degrades quicker than eukaryotic RNA and hence we would expect less of it to be present in the sample.

The RNA reads mapping to the commensal sequence are probably polyadenylated prokaryotic small mRNAs. Generally these are anomalous reads (i.e. only 1 read in the pair is actually sequence, and then the sequence is typically around 15-20 bp in length). When performing a BLAST with longest of these reads against the NCBI nr database, the best hits are bacterial, and often include mycoplasma or spiroplasma ribosomal sequences.

The commensal genome has been deposited at NCBI with accession number CP013128 (under the same bioproject identifier as the *B. glabrata* host, PRJNA12879). Annotation was provided with the NCBI Prokaryotic Genome Annotation Pipeline version 3.0, and consists of 595 genes, 555 coding sequences, 11 pseudo genes, 26 tRNAs, and the 5S, 16S and 23S ribosomal RNAs. In addition, gene annotation was provided with GLIMMER version 3.02 [49], using GLIMMER's shell script, g3-iterated.csh. This script was modified to use the NCBI Genetic Codes (number 4) for the Mycoplasma/Spiroplasmas. It discovered 578 genes. Of these genes, 328 were annotated with Gene Ontology (GO) terms and/or enzyme codes using Blast2Go[50] and UniProt. See Supplementary Data 12 for a list of coordinates of the predicted genes and their corresponding annotations.

Supplementary Note 8. Virus discovery from *Biomphalaria glabrata*

To date, no viruses have been reported from any species of schistosome-transmitting snails. Identification of viruses from sequence data from *Biomphalaria* may indicate novel pathogens of snails and have potential application toward development of molecular tools that can be applied for genetic modification of *B. glabrata.*We analyzed transcriptome sequences and contigs assembled from DNA sequences from BB02 *B. glabrata* generated in this study for the presence of virus-derived sequences We also examined RNA-seq data from the Brazilian BgBREstrain of *B. glabrata* (NCBI SRA database Accession: SRX327185) for identification of viral sequences. Sequence reads were assembled with either Newbler (454 sequencing data), or Trinity (v2.0.6)[51]. The assembled contigs were initially annotated by BLASTX against the NCBI nr data base[52]. Contigs that hit viral sequences were selected for further study to identify actual viral sequences based on viral sequence structure. The virus encoded proteins were further analyzed by BLASTP annotation. No sequences from DNA viruses were identified from *B. glabrata* sequence data. Contigs with sequence similarity to disrupted RNA viral genomes were identified from the transcriptome, suggesting potential integration of RNA viral sequence into the *B. glabrata* genome[53].

Three near complete viral genomes of novel RNA viruses were assembled from RNA sequence data. Sequence analysis of the viral genomes and encoded genes revealed that these viruses are similar to viruses of insects or other animals suggesting that these viruses, BGV1, BGV2 and BGV3, are likely to infect *B. glabrata*. Here we describe the genomes of these three novel viruses. Please note, NCBI categorized the submission of the below viral sequences as Third Party Annotation (TPA) Accordingly, sequence data are available from the authors upon request, pending release of GenBank accessions after publication of this study.

***Biomphalaria glabrata* virus 1 (BGV1):** The assembled BGV1genome has 9,008 nt, which encodes a 2,669 aa polyprotein (Supplementary Fig 13A). BLASTP results showed that the polyprotein sequence has about 25% similarity to insect small RNA viruses, specifically to iflaviruses, but the position of the non-structural and structural proteins is switched relative to iflaviruses.

***Biomphalaria glabrata* virus 2 (BGV2):** The second virus discovered from B. glabrata has an assembled genome of 16,477 nt (including a polyA tail), which is near full-length. BGV2 is unique for small RNA viruses in that it encodes three ORFs, with ORF1 and ORF2 being identical and encoding nonstructural proteins, and ORF3 encoding structural proteins (Supplementary Fig 13B). The RNA-dependent RNA polymerase (RdRP) domain shows 26% sequence similarity to Nora virus. The non-structural protein contains a new domain, the MCP domain, which may be involved in transmembrane function. A 3C-like protease domain (GxCG) was also identified. The putative coat proteins of BGV2 do not have sequence similarity to any known viruses but contain two capsid protein domains that are common in Picornavirales (rhv_like, cd00205; Rhv, pfam00073).

***Biomphalaria glabrata* virus 3 (BGV3):** The third near full-length virus assembled from the B. glabrata transcriptome is a novel virus of 10,047 nt in length. The genome encodes a single polyprotein of 2,852 aa (Supplementary Fig 13C). The arrangement of the polyprotein is similar to that of insect iflaviruses with the capsid proteins located at the N'-terminus, and the non-structural proteins at the C'-terminus. The polyprotein of BGV3 has sequence similarity to those of three small RNA viruses (encephalomyocarditis virus, enterovirus and heptitis A virus), which infect animals and humans. BGV3 have 26-27% similarity to other small RNA viruses. Hence BGV3 is also a novel type of unclassified virus.

Analysis of snail genomic DNA sequence data did not reveal any sequences derived from DNA viruses. Three near complete, novel viral genome sequences were identified from *B. glabrata* transcriptome sequence data. The genome structures and the protein annotation results suggest that these three viruses are animal small RNA viruses with limited homology to known viruses. These novel viruses may be snail-specific. Viruses of *B. glabrata* may be developed as tools for genetic manipulation and/or provide for novel approaches for the management (control) of snail vectors of schistosomiasis.

Supplementary Note 9. HSP annotation and expression in *B. glabrata*.

Heat shock proteins (HSPs) perform an essential biological role in protecting the cell from an array of insults, including stress from heat, cold, infection, injury or proteotoxic stresses. In response to these environmental and cellular perturbations, HSPs prevent intracellular protein aggregation and mis-folding, preserving the tertiary structure of cytoplasmic proteins, thereby allowing cellular functions to progress during recovery from the stress. The long-term health of the cell is determined by how efficient protein homeostasis can be maintained. Some HSPs, such as the HSP70 multi-family are both inducible and constitutively expressed in the cell. These well known proteins are highly conserved throughout evolution, from unicellular microorganisms, *e.g.* bacteria, to complex multicellular eukaryotes. The number of genes coding for the diverse HSP family members varies widely in different organisms. For classification, we followed the nomenclature based on the guidelines assigned by the HUGO Gene Nomenclature Committee[53] and used in the National Center of Biotechnology Information Entrez Gene database for the heat shock genes in annotating HSP sequences that were found in the *Biomphalaria glabrata* draft genome assembly (version 4.3). Accordingly, we have identified in *B. glabrata*, members of the following HSP families: HSPA (HSP70), HSPC (HSP90), DNAJ (HSP40), HSPB (small HSP, HSP 20), and the chaperonin (HSP60). We did not identify any members of the HSPH (HSP110) family. ***1) occur in the genome of B. glabrat:*** A local version of the UCSC Genome Browser[54] and the Blat alignment program[55] were used to search and visualize the *B. glabrata* genome draft assembly (version 4.3) . The mining of *conserved B. glabrata* heat shock protein (Hsp) sequences was conducted by searching the draft assembly against previously characterized HSP sequences from various molluscs, including *B. glabrata*, *Lottia gigantea* and *Aplysia californica*. Results from our annotation efforts to identify stress response genes such as heat shock proteins and the non-LTR retrotransposon *Nimbus* (*BgI*) like elements[56] and the relevance of these data in the ability of *B. glabrata* to act as the obligate intermediate snail host that transmits schistosomiasis are presented below (see headings 1-4). We performed a Blat search of the *B. glabrata* v4.3 genome draft assembly with query sequences/proteins of known HSPs from *B. glabrata* and other molluscs such as *A. californica* and *L. gigantea*, that were available in GenBank. We also used the RNA sequence data sets (12 tissues) available through the *B .glabrata* genome project. Our analysis identified five major families, namely HSP 20 (small HSPs), HSP40 (DNAJ), HSP60 (chaperonin), HSP70 (HSPA), and HSP90 (HSPC) in multiple scaffolds displaying significant sequence identity with known eukaryotic and prokaryotic heat shock proteins. Supplementary Data 13 shows the major conserved classes of HSPs that are represented in the snail genome, as well as the number and location of the nearest, well characterized snail non-LTR retrotransposon *nimbus* (*BgI*) element are provided. The *B. glabrata* HSP sequences were also annotated based on the presence of heat shock domains and motifs of other known HSPs identified in the Conserved Domain Database (CDD[57]. Some of the HSP families included pseudogenes. Supplementary Figure 14 shows the gene organization of members of all the identified HSP families thus far. The HSPA (HSP70) family is the largest, consisting of 6 members with multi-exon genes, 5 with single exons and more than 10 pseudogenes. The conserved N-terminal nucleotide - binding domain (NBD) of HSPA proteins was present (at least partially) in the majority of the proteins as was the signature sequence of the dnaK molecular chaperone. A previously identified *B. glabrata* HSP70 gene and its HSP70 promoter[58] were represented in entirety on LGUN_random_Scaffold 1164, yet due to inaccuracies in the assembly, both the gene and its promoter were split and located on either end of the contig.

The DNAJ (HSP40) family is represented in *B. glabrata* by a single gene (2 exons and 1 intron) that has the conserved J-domain known to be responsible for HSPA recruitment and for

stimulation of HSPA ATPase activity that is also required for interaction between HSP40 and HSP 40-like proteins with their partner HSP70s[59]. While DNAJ (HSP40) family is the largest in humans it appears to be the smallest in *B. glabrata*.

The HSPB family (small HSPs), are characterized by the conserved alpha crystallin domain (cd06464) and, in *B. glabrata* encompasses four different members and multiple pseudogenes. Unlike other HSPs, members of this family, including the pseudogenes (or possible gene duplications), appear to cluster in close proximity within the genome.

The HSPC (HSP90) family in the *B. glabrata* genome has two members that vary considerably in gene structure (one genes has 5 and the other has 13 exons) but encode similary sized proteins of 727 and 686 amino acids, respectively. The HSP90 gene reveals the signature histidine kinase-like ATPase ATP binding domain characteristic of HSP90s and other topoisomerases, and DNA repair proteins.

HSP60 belonging to the chaperonin family HSPD (an ortholog of the *E. coli* GroEL) is represented by 2 members in the genome. Both genes exhibit the characteristic GroEL- like type I chaperonin signature but one gene is organized as 12 exons (encoding a 571 aa protein) while the other has a single exon that yields a protein with 553 amino acids.

Members in these highly conserved HSP families show diversity in gene organization and size of expressed protein that may have resulted from duplications and retrotransposition (indicated by the presence of the large number of non-LTR retrotransposon *nimbus* (*BgI*) throughout the genome (Supplementary Figure 15) in addition to other mobile genetic elements [MGEs]).

The genes of the five HSP families identified from the *B. glabrata* assembly were compared with HSP sequences from other molluscs (*A. californica*, *L. gigantea*), human and schistosomes (compatible parasite- *Schistosoma mansoni* or incompatible parasites- *S. japonicum* and *S. hematobium*). However, since our primary interest is studying the host parasite interaction, we chose to determine the evolutionary relationship of the orthologous HSP sequences between the human host (*Homo sapiens*), the schistosome parasites (mainly *S. mansoni* and *S. japonicum* where genome sequences are better assembled/ annotated compared to *S. haematobium*) and the intermediate snail host *B. glabrata*. Protein sequences from *B. glabrata*, *H. sapiens*, *S. mansoni*, *S. japonicum* and *S. hematobium* were aligned using Phlogeny.fr[60] (Supplementary Figure 16, see legend for details of the analysis). Our analysis indicates that HSPs have diversified into 2 distinct clades with the DNAJ (HSP40) and the chaperonins (HSP60) deriving from one ancestral group and the HSPA (HSP70), HSP90 and HSP20 from the other. The HSP families then group into their specific clades and within each family the genes are more conserved between the two hosts (human and snail) than to the schistosomes. Except for HSP70 we found no homologs for the remaining HSPs in *S. haematobium*. Of the three medically significant schistosome species whose genomes have recently been sequenced, *S. mansoni* and *S. japonicum* are the best annotated, and could explain why *B. glabrata* HSP 90, 60, 40 and 20 orthologs were detected in these two, but not in *S. hematobium*.

Blat analysis of the *B. glabrata* genome was performed using the full-length sequence of *nimbus* (*BgI*, a non LTR retrotransposon;Genbank Accession EF413180) and with motifs encompassing, ORF1 (gag-like protein), ORF2 (pol-like protein) and the pol sub-domains expressing Endonuclease (APE), Reverse Transcriptase (RT) and Ribonuclease H (RNase H) as the query sequences. Supplementary Figure 15 shows the results from this analysis. There are a large number of *Nimbus* (*BgI*, 5869 bp) sequences interspersed throughout the *B. glabrata* genome (n=4495) of which n=60 have 50% of the bases and n=2 have 90% of the nucleotides aligned to the target sequence in the genome. We performed a similar analysis with the individual components of the *Nimbus* (*BgI*) sequence namely ORF1 (gag), ORF2 (pol) and the sub domains of ORF2 (endonuclease-APE, reverse transcriptase-RT and ribonuclease H- RNaseH). The results indicated

that ORF1, ORF2, APE, RT and RNaseH while also being highly represented (n=569, 1901, 399, 380 and 545 respectively), show higher representation of the 3'terminal end of the element, namely towards the RNaseH domain of ORF2 and ORF2 itself. This phenomenon was documented previously when a BAC clone that was rich in mobile genetic elements was sequenced[61]. We also observed a significant number of partial *Nimbus* (*BgI*) sequences in the proximity of heat shock proteins (data not shown) but documented only those that were identified by the repeat masker program. More data on the repetitive landscape of the genome are discussed in the Supplementary section 31.

Transposable elements are ubiquitous in eukaryotes and happen to be one of the key molecular evolutionary forces shaping the genome[62] and can effect this change by disrupting genes, inducing genomic rearrangements, influencing gene expression and mobilizing various types of non-autonomous sequences. The high occurrence of the *Nimbus* (*BgI*) sequences within the assembled genome, especially in scaffolds that also contain HSPs sequences, and evidence of co-expression of *Nimbus* (*BgI*) RT and HSP70 transcripts in infected snails[63] could indicate that regulation of *B. glabrata* HSPs, together with the wide variations we have observed in the members of the HSPs family, might be affected by MGEs in the snail genome.

The possible involvement of HSPs and MGEs is highlighted in Supplementary Figure 16. Here, we show results from real time qPCR analysis of RNA from *B. glabrata*, *Oncomelania hupensis hupensis*, and *Bulinus truncatus* with, and without, exposure (according to [63]) to compatible and incompatible schistosome species, S. *mansoni*, *S japonicum* and *S. haematobium*, respectively. Our results show that there is a 3.34 fold induction of HSP70 and 11.97 fold induction of *Nimbus* (*BgI*) RT transcripts, respectively, upon exposure of *B. glabrata* to *S. mansoni*, but not to *S. hematobium or S. japonicum*. Thus, the co-induction of RNA corresponding to both HSP70 and the *Nimbus* (*BgI*) RT following infection of the snail host with the parasite is a species-specific response and their co-expression and up-regulation points to their regulatory role during stress such as exposure to a compatible parasite.

This is a first attempt to characterize and organize the various members of HSPs in *B. glabrata*. Members in these evolutionarily conserved HSP families in *B. glabrata* show diversity in gene organization and size of expressed protein that may have resulted from duplications and retrotransposition. The repeat rich genome of the snail is interspersed with retroelements, such as SINEs and LINEs and these repeats may drive aspects of the diversity that we have observed in the HSPs sequences. How MGEs, especially *Nimbus* (*BgI*) are related to mechanisms of stress responses involving HSPs in *B. glabrata* and to the biology of why this snail acts as the intermediate host for *S. mansoni* remains to be studied further.

Supplementary Note 10. A proteogenomic approach to annotating the *B. glabrata* genome

Expression of (a) specific protein(s) within cells or tissues of an organism can be considered a functional "phenotype" reflecting ongoing biochemical or molecular processes. In an effort to identify and characterize the proteins, and their encoding genes, involved in the complex molecular interactions between early larval stages of the blood fluke *Schistosoma mansoni* and its snail intermediate host *Biomphalaria glabrata*, we are taking a proteogenomics approach [64] in which parasite-reactive snail proteins are isolated/enriched, subjected to shotgun proteomic analyses (including peptide sequencing, matching to the translated *B. glabrata* protein database, and identifying encoding genes and their genomic location). This approach to identifying genes encoding expressed proteins with potential functional relevance has been applied to an experimental schistosome-snail interactive system to identify *B. glabrata* plasma proteins that bind to surface tegumental proteins of *Schistosoma mansoni* primary sporocysts or those contained in larval transformation products (LTP) released during *in vitro* miracidium-to-sporocyst development.

In addition, because of the importance of the *B. glabrata* embryonic (Bge) cell line in serving as an *in vitro* cellular model for molecular and biochemical pathways involved in diverse snail functions, we also have initiated a comparative proteogenomic analysis of Bge cell proteins and their encoding genes with those of the snail. Since the establishment of the Bge cell line by Hansen almost 40 years ago[7], this cell line has been used as an *in vitro* snail cell model to investigate schistosome-snail immune interactions[65,66], as well as investigating regulation of cellular or molecular processes and the development of gene manipulation/ transfection methodologies[10,14,67,68]. However, to date no systematic attempts have been made to identify the genes encoding the Bge cell proteome, nor to compare expressed proteome-encoded genes to those of *B. glabrata* snails. Because this cell line originally was derived from *B. glabrata*, it would be extremely valuable to know if, and to what extent, Bge cells and *B. glabrata* snails have diverged in both gene structure and expression patterns. This information also would be important to validate this *in vitro* host-parasite system as an accurate reflection of molecular events occurring in the snail host. To begin addressing this deficiency, we conducted proteomic analyses on Bge cells to identify protein-encoded genes in this cell line for comparison with those found in the *B. glabrata* genome. Since heat shock proteins (HSPs/chaperonins) represent a highly conserved group of proteins functioning in multiple cellular/physiological processes, we focused our comparison on the HSPs constitutively expressed in Bge cells, again using a proteogenomic approach, showing that some are highly conserved (e.g., HSP60), while others are quite divergent (HSP70).

Two-day cultured *S. mansoni* primary sporocysts, obtained by *in vitro* cultivation of freshly hatched miracidia[69], were prelabeled with biotin and separated into 4 fractions using a modification of the ProteoExtract Subcellular Proteome Extraction Kit protocol (EMD Chemicals)[70]. This process involved the successive treatment of sporocysts in a series of extraction buffers that yielded fractions enriched in soluble cytosolic (F1), membrane (F2), nuclear (F3) and cytoskeletal (F4) proteins. The membrane-enriched fraction F2 was dialyzed against PBS, concentrated and reacted with streptavidin-agarose beads to create the F2-fraction affinity column. Isolated plasma (cell-free hemolymph) from susceptible (NMRI) and resistant (BS-90) *B. glabrata* strains were passed through the strepavidin-F2 column, followed by elution of bound plasma proteins from columns with a low pH acetate buffer. Isolated proteins were subjected to shotgun proteomic analyses involving in-liquid tryptic digestion followed by nanoLC/MS-MS (LTQ-Orbitrap) as described previously[71]. Deconvoluted ion spectra data were submitted for peptide mapping and MS/MS ion search analyses against a 6-frame translation of the *B. glabrata* genome (VectorBase; <https://www.vectorbase.org/>) to identify scaffolds encoding the peptide sequences. Peptide

fragment "hits" (exact sequence matches) to the translated snail genome typically yielded longer encoded protein sequences, which were then subjected to BLASTP searches of the nonredundant NCBI protein database using an in-house licensed MASCOT search engine for putative gene identification. Cells of the Bge cell line were similarly subjected to proteomic analyses as described above as part of a larger comparative study of the Bge cell proteome.

To illustrate the utility of the proteogenomics approach to functional gene discovery, we targeted the fibrinogen-related protein (Frep) family of sporocyst-reactive plasma proteins from susceptible (NMRI) and resistant (BS-90) *B. glabrata* strains to identify Freps with binding reactivity to sporocyst tegumental proteins and LTP of *S. mansoni* (NMRI strain). Predicted Freps representing different subfamilies (Frep 2, 3, 5, 7, 12 and 13) were identified based on peptide matching to the translated *B. glabrata* genome (VectorBase) followed by BLASTP query of the non-redundant NCBI proteomic db to identify gene predictions. Many of the resulting sequence "hits" represented new Frep variants, with most being identified in both snail strains. In plasma eluates of BS-90 only, several peptides originally identified as belonging to the Frep3 subfamily (LGUN_random_Scaffold18083/ LGUN_random_Scaffold47310) were subsequently found to align with high identities to a newly described galectin-related protein (Grep)[72] (Supplementary Fig. 18; Supplementary Data 14). PCR-generated amplicons specific to the identified ORFs of these scaffolds were produced only from cDNA synthesized from BS-90 *B. glabrata* headfeet, and not NMRI cDNA (Supplementary Fig. 19), thus corroborating the differential expression of this Grep-like protein only in the BS-90 plasma eluates. We are currently in the process of annotating the Grep gene, although initial evaluation of its location within the *B. glabrata* genome indicates that it is scattered across several scaffolds. In summary, using this proteogenomic approach, not only were we able to identify a number of larval-reactive novel Frep-like plasma proteins for annotation in the *B. glabrata* genome, but also functionally relevant lectins (e.g., Greps) that appear to be differentially expressed in the plasma of resistant snails.

Significant findings from use of the *B. glabrata* embryonic (Bge) cell line as *in vitro* model for schistosome-snail interactions are summarized as follows:

(1) HSPs/chaperonins identified as belonging to the HSP70, HSP60 and HSP90 family clusters are abundantly represented as constitutively expressed proteins in Bge cells.

(2) Of the 21 *B. glabrata* HSP70 genes currently annotated in VectorBase, Bge cell peptides map to 10 of these with varying degrees of coverage (1.8-43%; Supplementary Data 15), although three HSP70 members predominate: HSP70 (including cognate4), HSP70/cognate5 and 78-kDa glucose regulated protein (also known as Binding immunoglobulin Protein or BiP). Even though there is strong evidence that the Bge cell genes encoding these dominant HSP70s are identical to those of *B. glabrata*, our finding of sequence-matching HSP70 peptides with single or multiple amino acid (aa) differences (Supplementary Data 15; Supplementary Fig. 20) suggests the presence of possible genetic isoforms of these HSP70 proteins in the Bge genome.

(3) The Bge cell HSP60 chaparonin is highly conserved with peptides matching with 100% identity to *B. glabrata* HSP60 (Supplementary Data 15). It is the most abundant of constitutively expressed of HSPs in Bge cells. However, a related HSP60 family protein, T-complex protein 1 (TCP-1), also expressed in Bge cells, is encoded by genes found in several different scaffolds that likely correspond to distinct genes for different TCP-1 subunits (α, γ, ε). Although these are partially annotated in the *B. glabrata* genome, multiple aa sequence differences (Supplementary Data 15; Supplementary Fig. 21) suggest either the existence of other snail TCPs that have not yet been identified/annotated, or that Bge cell express unique variants of this protein family.

(4) Of the 3 HSP90 family members annotated in the *B. glabrata* genome, only 2 Bge-HSP90s were identified in this study; namely HSP90/84 and 94-kDa glucose regulated protein

(Supplementary Data 15). The large number of identical peptides accounting for 20% coverage of the snail HSP90/84 indicates that the Bge cell and snail proteins, and their genes, are likely to be identical. However, as with the other HSPs, the presence of several single aa substitutions may suggest the presence of genetic variants in Bge cells (Supplementary Data 15; Supplementary Fig. 22). The 94-kDa glucose regulated protein, in addition to being less abundant than HSP90/84, also appeared to have greater variability in aa sequence compared to the snail homologue. However, one of the peptides: TVWDWELMN**SVKPIWTR**, (see Supplementary Data 15), which originally was found to be only 58% identical in annotated LG4_random_Scaffold285, actually was found to be a 100% identical match in the same scaffold. The reason for this discrepancy appears to be due to the misplacement of an exon/intron boundary (exon 6/intron 6-7) during annotation that identified the 5'end of the coding sequence as an intron. Thus both HSP90 family members in Bge cells appear to be very similar, if not identical, to their *B. glabrata* counterparts.

By employing a proteogenomics approach, snail plasma proteins (e.g., novel Freps, Greps) engaged in snail cell-larval binding interactions were identified by proteomic analyses and their encoding gene sequences identified in *the B. glabrata* genome database (VectorBase). We anticipate that this approach will provide a critical assessment of the parasite-snail host protein interactome (and their encoding genes) that will provide valuable insights into our understanding of the mechanisms regulating schistosome-snail compatibility, as well as other biological processes impacted by larval infection. In addition, using the group of highly conserved HSP family proteins, we have shown that, despite a significant expansion of chromosomal content in the Bge cell line maintained in several laboratories[9], Bge cells still encode and express genes that are identical, or very similar, to those found in the *B. glabrata* genome. Given that the Bge cell line is the only existing cell line in the Phylum Mollusca, and a large repertoire of proteins encoded in and expressed by these cells are very similar to those of *B. glabrata* (unpublished data), there is ample justification to continue the use of this cell line as an *in vitro* model system for investigating snail-schistosome interactions or other important biological processes.

Supplementary Note 11. Cytochrome P450 (CYP) genes in *Biomphalaria glabrata*

CYP genes form a large superfamily of genes coding for heme-thiolate enzymes called cytochromes P450. The P450 enzymes are involved in a variety of biological processes, from the biosynthesis and metabolism of signaling molecules (such as hormones) to the metabolism and detoxification of foreign molecules (xenobiotics such as pollutants, pesticides and drugs). In addition, induction of P450 enzymatic activity by pollutants is often used in biomonitoring, yet little is known of mollusc P450 enzymes.

The genome was extensively mined for P450 sequences using the signature sequence of the heme binding motif as well as similarity to P450 sequences from other animals[73]. Transcriptome data (RNAseq data from 12 tissues, see Methods) were used to confirm or correct gene models. Comparative phylogenetic analysis was used to classify the CYP genes into clans and families and to highlight significant features of the snail CYPome.

The genome assembly contained sequences for about 99 CYP genes (Supplementary Data 17). The approximation is due to uncertainties regarding probable pseudogenes, truncated genes and "loose exons" requiring further resolution. Very few of these genes have close homologs in vertebrates or in insects, but the major animal P450 clans are represented in the genome. These are the microsomal CYP2 clan (about 33 genes), the CYP3 clan (about 35 genes), the CYP4 clan (about 7 genes) and the mitochondrial clan (about 18 genes). Several CYP3 and CYP2 clan P450 genes are present in genomic clusters of recently duplicated genes, as is typical of all CYPomes[74,75]. For instance, a tight cluster of nine CYP2 clan genes are found in on LG20_random_Scaffold442.

The CYP51 clan (absent from arthropods and nematodes) is represented by an ortholog of the vertebrate CYP51A1 gene, indicating that snails can demethylate precursor sterols at the 14-alpha position (and therefore presumably can synthesize sterols *de novo*). The CYP26 clan, also absent from arthropods and nematodes, is represented by two homologs of human CYP26A1, suggesting that molluscs can metabolize retinoic acid. The CYP20 clan is represented by one gene, an "orphan" gene whose function remains unknown even for the human gene.

We searched for clear orthologs of the genes involved in steroidogenesis in vertebrates and in arthropods. There are no orthologs of the vertebrate CYP11 (encoding the cholesterol side-chain cleavage enzyme) in the mitochondrial clan, nor CYP17 and CYP21 genes in the CYP2 clan. The genome also lacks the CYP19 gene (aromatase), found only in chordates[76]. Furthermore, there are no orthologs of the "Halloween" genes of insects encoding the P450 enzymes that are involved in ecdysteroid biosynthesis[74] (also see Supplementary Note 27). Therefore, it is likely that if snails utilize steroids as hormones, these would prove to be structurally different from either vertebrate or insect steroid hormones. Such a view is in accordance with the analysis of Scott[77].

The large number of P450 genes of the mitochondrial clan suggests that molluscs like arthropods but unlike vertebrates, utilise mitochondrial P450s in detoxification functions (Feyereisen, 2011). However, of those genes, one is a likely orthologue of the mitochondrial vitamin D metabolizing enzymes (CYP24/CYP27). Furthermore, there are two genes of the CYP7 clan, and those observations taken together suggest that sterol hydroxylation may occur in snails, perhaps leading to yet undiscovered signaling molecules.

Some genes are closely related to previously isolated mollusc P450s, all of unknown function. At least two genes encode homologs of a mitochondrial clan P450 expressed in dorsal bodies of *Lymnea stagnalis* (CYP10) and originally suggested to be involved in gonadotropic hormone biosynthesis[78]. However, these genes are highly expressed in all tissues, so it unlikely that they play a role in mollusc endocrinology. Transcriptome data indicate that about fifteen P450 genes are expressed in just one tissue (e.g. four in ovotestis and five in the CNS; see Supplementary Data

9) suggesting that these P450 genes are prime candidates for a molluscan-specific physiological function.

Lophotrochozoan CYPomes have remained unexplored until now. Our study provides the first systematic survey of P450 genes in this important group of metazoa, and provide comparative insights with arthropods, nematodes and deuterostomia whose CYPomes are well described. It also sets the groundwork for the rational design of selective molluscicides, for instance by using *B. glabrata*-specific P450s for bioactivation of pro-molluscicides, or by targeting for inhibition those P450 involved in essential signaling processes.

Supplementary Note 12. Pattern recognition receptors (PRRs) and cytokine in the *Biomphalaria glabrata* genome

The freshwater snail *Biomphalaria glabrata* serves as an intermediate host for several species of trematode parasites that infect humans and have severe consequences for worldwide human health. Understanding the snail immune response to these parasites helps interpret susceptibility/resistance phenotypes of the snail for parasite infection and thus transmission of infectious disease such as human schistosomiasis. We investigated two categories of regulatory factors of snail immunity encoded in the *B. glabrata* genome sequence: pattern recognition receptors (PRRs), which are responsible for pathogen detection, and cytokines, which communicate information about the immune state across the organism.

To identify PRRs and cytokines within the *B. glabrata* genome sequence, we used a previously outlined domain-based search[79]. Immune genes are often excluded from gene model predictions due to their high multiplicity and rapid evolution. Consequently, we performed HMMER searches[80] on both the complete set of gene models, as well as all of the open reading frames (ORFs) within the genome sequence. The set of ORFs (≥ 75 amino acids) was generated in all six frames using the transeq package within Expasy[81]. Hidden Markov Models (HMMs) for domains identified within *B. glabrata* were obtained from PFAM as follows: Amidase_2 domains, which are present in IL-17, PF06083.4; Mif, PF01187.11; PGRPs, PF01510.18; NACHT domains, which typify NLRs, PF05729.4; SEFIR, PF08357.4; SRCR, PF00530.10; TIR domains, which mark TLRs, PF01582.12; TNF, PF00229.11.

Pathogen recognition receptors in the *B. glabrata* genome sequence: Toll-like receptors (TLRs) are a well-characterized class of PRRs. The prototypical member, Toll, was first described in *Drosophila*, where it controls dorsoventral patterning in embryonic development. In adult flies, it became apparent that Toll mutants exhibit a severely impaired antifungal response[82]. Soon after, mammalian Toll homologs were identified and linked to LPS recognition[83,84]. In both mammals and fruit flies, TLRs are encoded as small gene families comprised of approximately 10 to 20 genes where they bind to panels of molecules that are broadly conserved among pathogens (i.e., LPS, peptidoglycan, dsRNA). In contrast, several examples have recently emerged in which the TLR gene families are expanded (reviewed in [85,86]). Within the invertebrate deuterostomes, the purple sea urchin *Strongylocentrotus purpuratus* genome encodes 253 TLRs [87,88], and the amphioxus *Branchiostoma floridae* genome contains 72 TLRs[89]. The protostome annelid *Capitella capitata* genome sequence encodes 104 TLR genes[90]. Although the function of these multigene families has not been directly established, their expression profiles and sequence characteristics are consistent with a novel immune strategy in which large panels of receptors are capable of recognizing an expanded or refined suite of antigens. Although the genome sequences of several other lophotrochozoans are available (the California sea hare *Aplysia californica,* the Pacific oyster *Crassostrea gigas*, and the owl limpet *Lottia gigantean* [91], the molluscan TLR repertoire has not yet been thoroughly analyzed.

Given the rapidly diversifying nature of immune receptors, we use a combinatorial domain-based strategy to identify *B. glabrata* TLRs from the genome sequence[79]. Structurally, TLRs contain an N-terminal signal peptide, an ectodomain that consists of a series of leucine-rich repeats (LRRs), a transmembrane domain, and an intracellular Toll/Interleukin-1 receptor (TIR) domain that mediates downstream signaling. This domain combination is unique to TLRs and can be used to identify TLRs with divergent sequence. Proteins containing TIR-domains were identified from among both the predicted peptides (Bgla1.0) as well as the translated genome sequence (BglaB1). In total, we have identified 27 complete TLRs, 9 partial TLR genes, and 20 pseudogenes

(Supplementary Data 17). Partial genes are either truncated by the end of a scaffold or ambiguous sequence. Pseudogenes are typically complete, but contain mutations that lead to stop codons or frame shifts. Given the potential for assembly errors, it is possible that some of the partial and pseudogenes may in fact be complete genes. In addition, there are 53 short fragments (< 300 nt) that exhibit sequence similarity to molluscan TLRs and may be highly degenerate remnants of TLR genes. It is notable that only four of TLRs were identified in the gene model predictions, which underscores the importance of searching the genome sequence directly for these types of molecules.

Phylogenetic analysis of the *B. glabrata* TLR (BgTLR) genes identified seven strongly supported classes (1-7; see Figure 2 in the main manuscript). The most common of these, class 1, contains 13 complete genes and eight pseudogenes. We also analyzed the phylogenetic relationship of the BgTLRs with respect to molluscan TLRs as well as TLRs from vertebrates and fruit flies (data not shown). Although the quickly diversifying nature of these immune genes interferes with robust sequence analysis, it is clear that several *B. glabrata* TLR classes are unique to this lineage (classes 1-3). This specificity may point to recent duplication events to generate these genes, and may highlight an important, species-specific role. The *A. californica* genome sequence contains orthologs of classes 4-6, as well as a small group of genes that forms a strongly supported cluster within the 1/2/3 clade. A single ortholog of the class 6 gene, BgTLR36, is also present in *L. gigantea*. The class 7 genes are present in all available molluscan genomes, and homologs are also found in the Hawaiian bobtail squid *Euprymna scolopes*.

TLRs are divided into two structural categories based on the domain architecture of the ectodomains: the single-cysteine cluster TLRs (sccTLRs), and the multiple-cysteine cluster TLRs (mccTLRs[92]). In sccTLRs, the ectodomain consists of 18-22 tandem LRR cassettes flanked by specialized cysteine-rich domains known as N-terminal LRR (LRRNT) and C-terminal LRR (LRRCT) domains. In contrast, in the mccTLRs, the LRRs are interrupted by an LRRCT and LRRNT domain, with an additional LRRCT just preceding the transmembrane domain (see Figure 2b of the main manuscript). The mccTLR-type has been lost within the vertebrate lineage, as no examples of this domain type have been identified in vertebrates. In contrast, mccTLRs predominate within the protostomes analyzed to date (reviewed in [93]). Notably, however, the TLR gene family expansions have all occurred within the sccTLRs. For example, the *Capitella capitata* genome sequence encodes a single mccTLR and 104 sccTLR genes[90]. This pattern also holds in the sea urchin and amphioxus genome sequences (the purple sea urchin has 250 sccTLRs and 3 mccTLRs; in amphioxus, 60 of the 72 TLR genes are sccTLRs; summarized in [87]). The *B. glabrata* genome sequence encodes both mccTLRs and sccTLRs. As in the other species, the gene family expansions occur within the sccTLRs. Of the 27 complete *B. glabrata* TLR genes, two are of the mccTLR type (Figure 1b). These two genes are also phylogenetically distinct from the sccTLRs and form the Class 7 clade (Figure 1a).

The *B. glabrata* sccTLR ectodomains are characterized by the presence 10-20 LRR cassettes as well as a distinct variable region in the central portion of the ectodomain (Figure 1b). In general, the BgTLRs have shorter ectodomains than TLRs in other species, which tend to have >18 LRRs. This unique variable region can be seen in the Class 1 BgTLRs where most of the ectodomain could be classified into defined LRR cassettes but the sequence between LRR11 and LRR12 of unclear structure. This region does not exhibit similarity to internal LRRs or LRRCT domains and is highly variable with respect to both sequence and length. Unlike the rest of the molecule, in which there are very few insertion/deletions, the sequences in this region varied in length by as much as 113 amino acids. BgTLR9 contains eight repeats of a unique 12 amino acid sequence (LDTLRKR[R|L]EV[A|T]G) in this region. This highly variable region appears to be under strong evolutionary pressure to diversify and may therefore be involved in either ligand binding or TLR

dimerization, as has been observed in other systems[94]. The single Class 6 gene, BgTLR36, appears to have a truncated ectodomain that consists of only 10 LRRs. A transcript was identified that corresponds to the N-terminal portion of this TLR and extends beyond the BgTLR36 start of translation predicted by the genome sequence. This transcript also encodes a signal peptide, suggesting that this may in fact be the complete sequence and that the lack of signal peptide in the genome sequence is an assembly error.

Peptidoglycan recognition proteins (PGRPs) are pattern recognition molecules that bind to peptidoglycan, a central component of bacterial cell walls. These proteins are characterized by the presence of PGRP domains, which are homologous to the bacteriophage T7 lysozyme, and, in some cases, exhibit catalytic amidase activity[95]. These proteins are important in both the human and insect immune responses, although they play different roles in these lineages. Mammals have four secreted PGRPs (PGLRYP1-4) that are primarily expressed in epithelial tissues and are largely bactericidal [96]. In contrast, the 13 *Drosophila* PGRP genes are transcribed into 19 proteins that are either secreted, transmembrane, or intracellular (reviewed in [97]). These proteins have acquired a variety of functions, including positively regulating immunity by signaling through the Toll and Imd pathways, as well as important roles for the catalytic PGRPs in negative regulation of the immune response[98]. Within molluscs, PGRPs have been characterized in the bobtail squid where they are expressed on epithelial surfaces and are involved in the control of the symbiotic *Vibrio fischerii* [99].

Prior to the availability of the genome sequence, four full-length PGRP cDNAs were isolated from *B. glabrata* (PGRP-LA, PGRP-LA1, PGRP-LA2, and PGRP-SA[100]). Here, we report the identification of a total of eight *B. glabrata* PGRP genes (Supplementary Data 17). Consistent with the original report, PGRP-LA, PGRP-LA1, and PGRP-LA2 appear to be alternatively spliced isoforms of a single gene (BgPGRP-8; BGLB010280). Furthermore, gene model BGLB000840 corresponds with the previously described PGRP-SA (BgPGRP-5). Only a single PGRP (BgPGRP-2) does not contain any of the five conserved residues that form the catalytic site[96]. One PGRP (BgPGRP-1) encodes a protein with two PGRP domains. None of these proteins have predicted signal sequences, which is most likely due to incomplete gene models.

Other pattern recognition receptors. The remaining well-characterized PRR families include the scavenger receptors that contain scavenger receptor cysteine-rich (SRCR) domains, Nod-like receptors (NLRs), and Gram-negative binding proteins (GNBPs). Each of these families plays unique roles in immunity in different taxa. We did not identify any NLRs in the *B. glabrata* genome, as evidenced by the lack of NACHT domains in both the gene models and the translated genome. This is consistent with a loss of the NLRs in protostome lineages. A single GNBP was identified within the gene models (BGLB004123), which corresponds to the previously described GNBP from this species (Genbank EF452345.1;[100]). The *B. glabrata* genome also encodes a suite of 31 SRCR domains that are distributed among 17 gene models. Although there are a total of 88 open reading frames within the genome sequence that encode SRCR domains, these domains are distributed throughout 63 scaffolds, and there is no evidence of transcripts encoding multiple SRCR domains within the transcriptome. Thus, while it is not likely that multi-domain SRCR proteins are present within *B. glabrata*, some of the single SRCR domain proteins may be important in the snail immune response.

Homologs of vertebrate cytokines are particularly difficult to identify in distant taxa. This is largely due to the rapid diversification of this class of genes that are typically small, secreted molecules with little domain structure. Some families, however, are easier to identify than others. We searched the *B. glabrata* genome and gene models for homologs of all known vertebrate interleukins and cytokines. Genes from three families of cytokines are present within the snail genome: IL-17, Macrophage inhibitory factor (Mif), and Tumor Necrosis Factor (TNF).

IL-17 has been well-characterized in vertebrates as an inflammatory cytokine that is expressed by lymphocytes[101] and in barrier epithelia[102,103]. IL-17 homologs have been identified in several invertebrates, including the sea urchin[88], amphioxus[89], and two species of oysters[104,105]. Within the *B. glabrata* genome sequence, we have identified 12 IL-17 domains. Because these genes are small and are typically encoded by more than one exon, most of these open reading frames are likely to be portions of IL-17 genes. A single IL-17 transcript (Locus_1289) was isolated from the terminal genitalia and can be used to identify the complete coding sequence for two closely related IL-17 genes. The IL-17 receptor (IL-17R) is typically characterized by an intracellular Sef/IL-1R (SEFIR) domain that is similar to TIR domains. We were unable to confidently identify any transmembrane proteins that contain a SEFIR domain. However, given the similarity between the SEFIR and TIR sequence, it is possible that a divergent TIR domain-containing protein mediates IL-17 signaling in the snail.

Additionally, we have identified a small set of four homologs of Mif, and 11 homologs of TNF (Supplementary Data 17). One of the Mif molecules, termed here BgMif-1, was described previously, where it is expressed in circulating hemocytes and appears to be involved in the host response to *Schistosoma mansoni*[106].

Supplementary Note 13. Toll-like receptor signaling pathway in *B. glabrata*.

Toll-like receptors (TLRs) are a family of conserved pattern recognition receptors (PRRs) that have been found in nearly all metazoan phyla including the phylum Mollusca[93,107-110]. Therefore it is not surprising that TLRs were also identified in *B. glabrata* (see Supplementary **Note** 12). To identify signaling proteins that may function downstream of TLRs, the *B. glabrata* genome was searched using homologs of established TLR-associated signaling proteins using BLAST. Gene predictions were confirmed using RNAseq data acquired through the genome project. Putative protein sequences were analyzed in InterPro (http://www.ebi.ac.uk/interpro/) for the presence of signature motifs, such as transmembrane, Toll/interleukin-1 receptor (TIR), and Death domains.

This approach revealed a comprehensive TLR signaling network in *B. glabrata* (Supplementary Fig 23, Supplementary Data 18) that includes myeloid differentiation primary response 88 (MyD88), interleukin-1 receptor-associated kinase (IRAKs), TNF receptor-associated factor (TRAFs), nuclear factor of kappa light polypeptide gene enhancer in B-cells (NF-κB), inhibitor of NF-κB (IκB) and IκB kinase (IKKs). We discovers additional, less well known participants in TLR signaling, such as evolutionarily conserved signaling intermediate in the Toll pathway (ECSIT) and sterile alpha and HEAT/Armadillo motif containing protein (SARM). TLR-induced signaling can lead to gene transcriptional regulation through NF-κB transcription factors, and homologs of these are already known in *B. glabrata* (Zhang & Coultas, 2011). Additional transcription factors associated with TLR signaling were identified such as activator protein 1 (AP1), interferon response factor (IRFs) and lipopolysaccharide-induced TNF factor (LITAFs). Interestingly a number of Toll/interleukin-1 receptor (TIR) domain containing adaptor proteins were not found in the *B. glabrata* genome, including TIR-containing adapter protein (TIRAP), TIR-containing domain adapter inducing interferon–β (TRIF) and MyD88 adapter like (MAL). However, these TIR domain containing proteins have also not yet been identified in other molluscs.

The precise roles of TLR signaling in *B. glabrata* remain to be determined, especially regarding the alternative cytoplasmic factors that are involved in particular responses and which downstream processes are regulated. Already there are indications that TLR-induced signaling regulates molluscan immune responses, based on reports of increased TLR transcription following exposure to bacteria and/or viruses in *Crassostrea gigas*, *Mytilus galloprovincialis*, *Haliotis discus discus*[107-109] and schistosome infection in *B. glabrata*[111]. Typical ligands recognized by TLRs include pathogen associated molecular patterns (PAMPs) like LPS, flagellin, double stranded DNA and zymosan[112]. It is of interest to investigate which and how schistosome-derived antigens may trigger TLR-mediated signaling to activate immune responses in *B. glabrata*. Given the activation of mammalian TLRs by helminth-derived products[113] it is not unlikely that a similar scenario might exist in *B. glabrata*.

Supplementary Note 14. *Biomphalaria glabrata* FREP genes.

Biomphalaria glabrata responds to parasite infection by increasing in abundance the expression of parasite-reactive plasma lectins termed fibrinogen-related proteins (FREPs). These lectins comprise one or two upstream Immunoglobulin (IG) domains and a C-terminal fibrinogen-like (FBG) domain[36]. FREPs belong to a large gene family, and individual snails express unique, highly diversified repertoires of FREP sequences due to somatic mutation of the germline encoded genes[114-115]. Functional transcriptomics showed that FREPs contribute to anti-trematode resistance[115-116], FREPs are thought to provide a polymorphic non-self recognition system that co-determines immunological compatibility with trematode parasites capable of antigenic variation[117]. The genome assembly was examined for the number and diversity of germline FREP genes, as well as the genomic organization of FREP genes.

The assembly was queried for FREP gene sequences using TBLASTN (protein queries versus translated nucleotide sequences) with all previously characterized FREP genes, especially FREP3 and FREP4. Genomic scaffolds with regions showing BLAST similarities were inspected by eye for presence of FREP genes, and full length genes (exon 1 encoding the SP was not always evident) were annotated for domain organization and intron-exon structure, using RNAseq data available from *B. glabrata* and SMART (http://smart.embl-heidelberg.de/). FREP genes were aligned (equivalent codons) by eye with previously characterized FREP genes (Bioedit, http://www.mbio.ncsu.edu/BioEdit/bioedit.html) to construct a gene tree (Mega v.6,[118]).

BLAST searches with previously characterized FREP gene sequences identified multiple hits, especially involving FBG-related sequences, including unrelated genes, isolated (single) FREP-like exon and sequence fragments and bona fide FREP genes consisting of IgSF and FBG domains (note that the leading exons encoding signal peptides were not always represented in the assembled data). In total 22 FREP genes were identified, with 19 FREPs comprising two Ig domains and 3 FREPs with a single IG domain. Additional FREP genes may be present in the genome, for instance, coding regions with high similarity to FREP2 were distributed over several scaffolds but these could not be interpreted as a complete gene and accordingly such sequences were not reported. The genome contained FREPs with high sequence similarity to previously characterized FREP genes, possibly representing allelic versions of gene loci that differ among strains of *B. glabrata*. For example FREP3.1, characterized from M line *B. glabrata* in 2001[119], shared 94% amino acid sequence identity (97% similarity) with the gene designated 18 tig 2841 1832 from the BB02 strain *B. glabrata* genome assembly. A novel type of FREP was found, encoding for N-terminal sequence with similarity to a PAN_AP like domain (SMART accession SM00473) preceding a single IG and an FBG domain at the C-terminus (Supplementary Figure 24). PAN_AP domains may mediate carbohydrate or protein interactions leading to protein dimerization or act as ligand for attachment to cells[120].

Inspection of the genome revealed novel intron-exon architectures for single IG domain FREPs, additional to that reported previously[121]; the IG domain may be encoded across two or three two exons. Another FREP gene uniquely consisted of two exons with the first encoding the signal peptide and the second combining the IG, ICR and FBG domains (compare A,B,C in Supplementary Figure 25). All FREP genes with two IG domains have the same uniform intron-exon organization as reported previously[119]. The intron/exon structures of the different categories of FREP genes from *B. glabrata* differ consistently in positions of splice sites relative to the encoded domains from AcFREP1 from *Aplysia californica* (AcFREP2 is a putative retrogene and lacks introns)[122]. This suggests different evolutionary history of the dual IG FREP genes between *B. glabrata* and *A. californica*, panpulmonate and euphistobranch gastropods, respectively.

: Many of the FREP genes are clustered on scaffolds, frequently in proximity to isolated fragments of FREP-like sequences that resemble complete or partial exons. Sequence comparison of intact FREP genes showed instances of highest similarity among genes from the same scaffold (Supplementary Figure 26). Likely, these FREP sequences resulted from gene duplications. The presence of many genes and gene fragments suggests great gene plasticity and may provide sequence for diversification through gene conversions.

RNAseq cDNA align to FREP genes with high confidence, showing polymorphic positions within the FREP sequences (not shown). Dheilly *et al*.[76] have shown that RNAseq data reveal patterns of single nucleotide polymorphisms that are the same as those shown by targeted Sanger sequencing of subcloned variant sequences and as such, reveal aspects of nucleotide diversification of FREP genes (Zhang *et al*., 2004).

The genome provides a previously unattainable, improved view regarding number and diversity of FREP genes of *B. glabrata*. In total, 22 intact genes were identified (Supplementary Fig 26). Variations in intron-exon structure suggest high plasticity of these genes, and suggests differences in evolutionary history of FREP genes between families of gastropods.

This analysis provides an indication of the number of germline genes that is at the basis of the individually diverse FREP sequence repertoires evident from distinct *B. glabrata* snails. A FREP sequence with a N-terminal PAN_AP domain provides a novel domain organization, future analysis may provide us with an expanded understanding of the functional role of FREPs. Finally, upstream sequence and intergenic regions are now available to explore regulatory sequences towards study of regulation of FREP gene expression in *B. glabrata*.

Supplementary Note 15. Complement-like sequences of *Biomphalaria glabrata*

The snail *Biomphalaria glabrata* serves as an intermediary host for the blood fluke *Schistosoma mansoni*, and although *B. glabrata* is essential in the transmission cycle for the human disease schistosomiasis, the immunological interactions between snail host and parasite are still not fully understood. Using the *B. glabrata* genome assembly available at Vectorbase.org, we have identified several sequences containing complement-like domains in *B. glabrata*. The complement system is an important component of the innate immune response and it has been involved in key defense responses such as pathogen opsonization, inflammation, recruitment of immune cells, and direct cell lysis[123,124]. A search of the genome sequence identified several predicted genes with the alpha-2-macroglobulin, complement component C1q, and complement control protein (CCP) domains. In addition, using the *B. glabrata* genome and the RNAseq transcripts in combination we were able to extend the coding region of these sequences. We have identified RNA transcripts with sequences similar to C1q, C3, and CCP domain containing proteins.

Hypothetical transcripts and genomic regions of interest in *B. glabrata* were identified by obtaining complement-related amino acid sequences and aligning these to the *B. glabrata* genome via the BLAST function at Vectorbase.org. RNAseq sequence files were provided for twelve tissue types by the Snail Genome Project Consortium. Using NCBI BLAST+ (2.2.29+), these twelve RNAseq files were converted into individual BLAST databases, and then, local BLAST was performed via using the previously identified hypothetical transcript as query for each tissue database.

The output files from local BLAST gave hits to independent transcripts on each database. These RNA transcripts were translated and their amino acid sequences scanned using the NCBI BLASTP Suite at blast.ncbi.nim.nih.gov. The highest matching BLAST hit from BLASTP was subsequently used as query against the RNAseq databases in an effort to obtain the most complete sequences for each identified homologue. The resulting transcript hits were translated, and then aligned with the homologue-fragment(s) we had previously identified.

The RNAseq data for twelve *B. glabrata* tissues were used as followss: 1) Cufflinks (version 0.0.6) was used to assemble the transcripts to the reference *B. glabrata* genome using the reference annotation file hosted at Vectorbase.org; 2) Cuffmerge (version 0.0.6) was used to merge the assemblies obtained from Cufflinks; 3) Gffread was used to extract the nucleotide sequences obtained from the merged transcript file obtained from Cuffmerge[125]. The resulting FASTA files were analyzed to construct the most complete RNA sequence for each transcript of interest.

The transcript nucleotide sequences were aligned against the *B. glabrata* genome via BlastN at Vectorbase.org. To refine our annotations, the resulting hits were used to locate the nucleotides of the coding region in the transcripts relative to the genomic scaffolds.

Four complement-related and four TEP-related sequences were identified using the *B. glabrata* genome BLAST and RNAseq-Local BLAST. Among these, there were sequences similar to complement C1q, complement C3, and to CCP domain containing proteins. The *B. glabrata* sequences identified via genome browsing and BLAST analysis of the FASTQ tissue files are summarized on Supplementary Data 19. The sequences have been annotated in the *B. glabrata* genome at https://www.vectorbase.org (see Supplementary Data 19 for genome location and sequence names).

We identified a hypothetical transcript (BGLB000101) in the *B. glabrata* genome that partially coded for a C1q-domain; C1q is the initiator of the classical pathway of complement[123], and in some organisms may serve as a lectin in the lectin pathway[126]. Using BGLB000101 as query, we found a complete transcript coding for a 220 amino acid protein with the C1q domain and homology

to previously reported C1q sequences (see Supplementary Data 19). Interestingly, we found that there is a very similar unannotated sequence in *Aplysia californica* (XP_005101350) Alignment of the *B. glabrata* C1q-like with C1q sequences from human and mice showed high similarity in terms of protein size, amino acid residue identity, and location of the C1q domain (Supplementary Figure 28).

The hypothetical transcript BGLB000031 was identified having alpha-2-macroglobulin (A2M) domains, and had NCBI BLAST homology hits to known C3 proteins (Supplementary Data 19). C3 is not only the initiator of the alternative pathway of complement, but an important molecule required for the progression of the complement cascade and its immune activity[123]. We identified multiple RNA transcripts fragments that, when aligned with a C3 homologue, coded for a sequence of 1753 amino acids (comparable in size to *C. gigas's* C3, which is 1744 amino acids). This sequence matched BGLB000031 in the *B. glabrata* genome and found matches to C3 sequences by NCBI BLAST. Furthermore, BGLB000031 contained all common C3-protein domains, and organized in a similar manner as those reported in C3 sequences from other organisms.

Because C3 is part of the TEP protein family (consisting of the TEP, A2M and C3 groups)[126,127], we performed a phylogenetic analysis to determine in which TEP-subgroup the *B. glabrata* C3-like sequence aligned. The selected C3, C4, and C5 sequences formed distinct groups and *B. glabrata* C3-like sequence aligned with in the C3 group (Supplementary Figure 29).

The hypothetical transcript BGLB000132 from the *B. glabrata* genome contains CCP domains (Supplementary Data 19). Using the RNAseq data, we were able to identify a matching transcript coding for a 3011 amino acid protein with a VWFA domain at the 5' end, followed by three CCP domains, three calcium-binding EGF domains, and a pentraxin domain towards the 3' end. Furthermore, other organisms also code for similar uncharacterized proteins, as found by BLAST analysis (Supplementary Data 19).

Hypothetical transcript BGLB014448 also contained complement control protein (CCP) domains. Using this sequence as a query, we were able to identify RNA transcripts that when aligned together coded for a putative protein of 4847 amino acids. This transcript sequence contained 52 CCP domains, multiple calcium-binding EGF-like domains, and a pentraxin and Von Willebrand factor type A domains too (Supplementary Data 19). Similarly to the previously described *B. glabrata* CCP-1 sequence, CCP-2 was closely related to other proteins that are yet to be characterized themselves (Supplementary Data 19).

Based on the multiple CCP domains found in this sequence, its size and the sequential order of its domains, it suggests that this protein could be related to Cr1 (GenBank Y00816), a C3b complement receptor in humans (Morley & Walport, 2000). Cr1 is a large protein (2044 amino acids) composed of thirty CCP domains at the 5' end, and a transmembrane and cytoplasmic region towards the 3' end (Morley & Walport, 2000).

The hypothetical transcript BGLB000135 coded for a sequence containing a CCP domain and a serine protease inhibitor (KU) domain (Supplementary Data 19). We obtained a matching RNA transcript coding for a product with 309 amino acids. Unfortunately the BLAST results obtained do not provide sufficient evidence to permit us to speculate regarding the possible identity and function of this protein ((Supplementary Data 19). Nonetheless, based on its size and functional domains present, it could be function as a secreted serine protease inhibitor. Small protease inhibitors and serpins have been reported to be involved in the regulation of complement in other organisms[128-130].

The identification of complement-like factors in *B. glabrata* indicates the potential existence of novel and unexplored immune pathways in this molluscan species. The study of snail-complement could launch new venues of research, especially in relation to the expression of these

proteins in response to schistosome infections. Complement research is an expanding area of investigation, as even in the best studied vertebrate systems, the complement system still remains to be fully characterized; for example, recent studies have associated complement components to novel functions such as development and tissue regeneration[131]. Our analysis did not reveal from *B. glabrata* the gene homologs for the complement-like factors (C5b, C6, C7, C8, C9) that could form a complement membrane attack complex (MAC) and effect lysis of target cells, rather the particular complement factors described here are putatively able to opsonize targets, marking these for elimination by phagocytic cells. Elucidation of functional roles of complement-like factors in *B. glabrata* could offer new strategies to target immune activation in parasite-susceptible snails.

Supplementary Note 16. The *Biomphalaria* apoptotic network.

In most invertebrates, apoptosis plays a crucial role in immune defense against pathogens such as parasites and bacteria but can also be involved in adaptation to environmental stressors such as exposure to UV light or heat[132-134]. For example, apoptosis of virus-infected cells is considered a innate cellular response to block viral infection within the host [135-138]. In contrast, programmed cell death of hemocytes can be also induced by many pathogens to weaken the host immune system's ability and facilitate invasion[139,140]. Apoptotic control pathway may therefore be crucial in host-pathogen interactions[141].

The different apoptotic pathways are well documented and characterized in invertebrate model animals like *C. elegans* and *Drosophila* [142,143,145,146] and also for animal of economic interest like oyster[146]. However, for *Biomphalaria glabrata*, despite several comparative molecular approaches (transcriptomic, proteomic studies) no description of the apoptotic pathways has been available until now[147-151].

In depth analysis of the *B. glabrata* genome assembly and associated RNAseq data using BLAST with apoptosis homologs described from other invertebrates (see references above) and Interproscan analyses reveals a high degree of conservation in key apoptotic genes involved in both the extrinsic and the intrinsic signaling pathways (Supplementary Data 20).

Molecular determinants of the extrinsic pathway have been identified *in silico*, such as death ligands (FAS), receptors (TNFR, TrailR) and adaptors (FADD, TRAF, FAF, RAIDD). Genes involved in the intrinsic mediated cell death pathway like Bcl-2 family proteins (Bax, Bak, Bcl-2), releasable mitochondrial factors and caspases have been also found (Supplementary Fig. 30). Indeed, caspase domain signature recognition (IPR002398 and IPR011600) using Interproscan software revealed the presence of 15 caspases positioned in 10 different genomic scaffolds. Three of the caspases possess long prodomains that contain either a death effector domain (DED) or a caspase recruitment domain (CARD) and can be classified as initiator caspases. The others can be considered as effector caspases characterized by a short prodomain the activation of which will cause death by cleaving different cellular proteins such as the DNA repair enzyme poly(ADP-ribose) polymerase (PARP) or the endonuclease involved in DNA fragmentation (DFF) (Supplementary Fig. 31). The number of caspase genes is different from those found in invertebrates like the nematode *C. elegans*, the silkworm *Bombyx mori*, the fruit fly *Drosophila melanogaster*, the sea anemone, the oyster, the urchin and in vertebrates such as fishes and mammals with 4, 5, 7, 10, 24, 31 and 12, 13, respectively[146,152-154].

Interestingly, the apoptosome formed by the complex caspase 9 and APAF1 was found thus reinforcing the notion of a conserved basic machinery required for the activation of caspases in the intrinsic pathway. Moreover, a homolog of APAF-1 interacting protein (APIP) known as an apoptosomal regulator was identified. A caspase 8-like protein was identified, strongly suggesting a conserved cross talk capability between the extrinsic and intrinsic pathways even if caspase 8 substrates have not been characterized. Mitochondria are at the crossroads of different apoptotic signaling pathways and the preservation of their membrane integrity is highly controlled by the Bcl-2 family proteins to inhibit the release of pro-apoptotic factors such as cytochrome C. Six members have been characterized and classified in pro- or anti-apoptotic factors according to the Bcl2 homology (BH) domain number and their similarity (Supplementary Fig. 32). Some others factors phylogenetically close to the Bcl2 family members have been identified such as the anti-apoptotic TMBIM (Transmembrane BAX Inhibitor-1 Motif-containing) proteins and the pro-apoptotic Bnip (BCL2/adenovirus E1B 19 kDa protein-interacting protein), molecules which are potential targets for caspases[155,156].

Surprisingly, the *Biomphalaria* genome has about 50 genes located on 26 genomic scaffolds encoding BIR domain-containing proteins that are potent caspase inhibitors. The BIR motif associated with the RING domains characterizes all IAP protein family members. 9, 12 and 3 IAP full length sequences containing one, two or three BIR motifs have been identified respectively. The large number of genes is consistent with that found in other molluscs (17 for *Lottia gigantea* and 48 for *Crassostrea gigas*) while their number is limited in other clades (2 for *Caenorhabditis elegans*, 3 for the sea anemone, 4 for *Bombyx mori* and *Drosophila melanogaster*, 7 for the urchin and 8 for human). This family expansion in *Biomphalaria* suggests an important role in the regulation of apoptosis revealing a sophisticated apoptotic system but also in innate immunity in response to pattern recognition receptor stimulation[157]. Two major inhibitors of IAP activity released from mitochondria during apoptosis, Smac/DIABLO and two serine proteases similar to HtrA2, were also identified[146,152-154].

Supplementary Note 17. Production of reactive oxygen species and protection against oxidative damage.

Biomphalaria spp. are excellent subjects for studies on genes encoding redox-relevant proteins. As aquatic pulmonates, these snails breathe both with lungs and trans-dermally, and hemoglobin comprises over 95% of the plasma proteins[158]. Yet, implying a capacity to tolerate hypoxia, some species can survive weeks of aestivation when their habitats dry up[159]. As a variably susceptible lophotrochozoan intermediate host that may transmit the important lophotrochozoan human parasite, *B.glabrata*'s immune mechanisms are of obvious relevance to human health. These mechanisms rely heavily on reactive oxygen and nitrogen intermediates (ROS and RNS) produced by circulating phagocytic defense cells (haemocytes) [160-163]. ROS and RNS are highly reactive and can effect irreversible cell damage.

In the *B. glabrata – S. mansoni* parasitism, snail populations exhibit a wide spectrum of levels of resistance to the parasite. The capacity of the snail's haemocytes to generate ROS and RNS in response to parasite stimuli is documented[164]. In at least some cases, snail resistance mechanisms include oxidative stresses imposed on parasites by host snail haemocytes. *In vitro* studies have revealed that H_2O_2 and NO[162,164] contribute to the *B. glabrata* haemocyte-mediated killing of schistosome larvae. In fact, H_2O_2 is essential for the efficient killing of *S. mansoni* sporocysts. This may depend on its stability and its capacity to cross cell membranes[165]. Furthermore, hemocytes from some *S. mansoni*-resistant snails generate significantly more H_2O_2 than hemocytes from a susceptible snail strain, perhaps due at least in part to the former having constitutively elevated levels of the mRNA encoding the copper/zinc superoxide dismutase (CuZn SOD) [166-168]. In eukaryotes, production of ROS occurs in both phagocytic and non-phagocytic cells. Studies in non-lophotrochozoans have shown that the production of ROS typically begins with the synthesis of superoxide anion ($O_2.^-$), and continues with its contribution (by dismutation) to the production of H_2O_2. A relatively long-lived, highly diffusible and potentially damaging molecular species, H_2O_2 is soon either scavenged (metabolized to innocuous oxygen species) or contributed toward to the production of even more toxic ROS. Other important enzymes are those that either yield such additional destructive ROS or that 'scavenge' ROS by conversion to more innocuous chemicals. Production of reactive nitrogen intermediates begins with nitric oxide synthase.

ROS and RNS are chemically reactive molecules containing oxygen and nitrogen, respectively. Examples include oxygen ions, peroxides and hypohalous acids, nitric oxide and peroxynitrite. These are natural byproducts of the normal metabolism of oxygen and are generated by exogenous sources such as ionizing radiation. In addition to their roles as defense molecules, they have important regulatory roles in transcription, cell signaling and homeostasis, including apoptosis[169], platelet traffic (in vertebrates[170]), and induction of host immunity[171]. During times of environmental stress (e.g., exposure to UV, heat, or pathogens), levels of ROS in tissues can increase dramatically[172]. This can damage cell structures as a consequence of oxidation of nucleic acids, proteins and lipids. Cumulatively, this is known as oxidative stress.

To limit the deleterious effects of ROS on self, in eukaryotes adaptations have evolved to restrict the spread of ROS within and between cells, and to regulate their levels. These are well illustrated in the context of immune responses against pathogens. First, phagocytosis ensures the production of ROS in phagolysosomes of phagocytic cells. Second, the encapsulation of bigger parasites restricts ROS to the cellular capsule surrounding the intruder. Third, cells defend themselves against of the threat of ROS-mediated oxidative damage using enzymes such as catalases, lactoperoxidases, glutathione peroxidases and peroxiredoxins. Some vertebrate glutathione-S-transferases repair oxidative damage. Small molecule antioxidants such as ascorbic

acid (vitamin C), α-tocopherol (vitamin E), uric acid, thioredoxin and glutathione are additional intracellular antioxidants. Similarly, polyphenol antioxidants assist in preventing ROS damage by scavenging free radicals. In vertebrates, antioxidant activity in the extracellular space relies on α_1-microglobulin and uric acid. Other known antioxidants include melatonin. In *B. glabrata* a robust capacity for protection of self from oxidative damage is implied by the observation that, in the 15% of the proteome that is represented by the secretome, 'antioxidant activity' is one of three main terms under the GO umbrella of 'molecular function' (Supplementary section 3). Indeed most of the genes known to be involved in protection from oxidative stress have now been identified in the *B. glabrata* genome.

The BB02 *B. glabrata* genome assembly was extensively mined for genes encoding enzymes involved in the generation and scavenging of ROS and RNS. We used complete sequences, signature sequences, and/or conserved domains of molecules previously identified in related species or in an annotated *de novo* assembled transcriptome of *B. glabrata* (archived at the website, http://2ei.univ-perp.fr/?page_id=89, scroll to file Reference transcriptome). Transcriptome data were used to confirm, correct or complete gene models. Expression of candidate genes was analyzed using the RNAseq data obtained from other Brazilian isolates of *B. glabrata* at the Laboratory for Interactions-Hosts-Pathogens-Environments (I.H.P.E.) at the University of Perpignan, see the work of Deleury *et al.* [151].

Interrogation of the *B. glabrata* genome has revealed a comprehensive capacity to both make and metabolize reactive oxygen species (ROS) and nitric oxide (NO) (Supplementary Fig. 33, See Supplementary Data 21 for gene details).

The biogenesis of ROS begins with superoxide-generating systems, notably the NADPH oxidase (NOX) complex or dual oxidases (DUOX). This family of proteins is named the NOX/DUOX family. NADPH oxidase is a membrane-bound enzyme complex, and generates superoxide by transferring electrons from NADPH inside the cell across the membrane and coupling these to molecular oxygen to produce superoxide anions. In *B. glabrata*, functionally essential proteins of this complex are *nox2* (JZ482247;[173]), *phox22/ cytochrome245* (JZ482252), and potentially a gene product (JZ482248) with features indicative of relationship to *phox49*. Dual oxidases are oxidoreductase enzymes that catalyze the synthesis of two essential ROS molecules: the anion superoxide ($O_2.^-$) and hydrogen peroxide (H_2O_2). In humans, two isoforms of dual oxidase occur encoded by the DUOX1 and DUOX2 genes. In *B. glabrata* the two isoforms are also identified; DUOX1 seems to be encoded by 4 genes and DUOX2 by 4 genes (Supplementary Figure 33). To be activated, DUOX enzymes need to be acted on by DUOX maturation factor (DUOXA). This allows ER-to-Golgi transition, maturation, and translocation to the plasma membrane of functional DUOX. Two DUOXA have been identified in human: DUOXA1 arranged head-to-head to and co-expressed with DUOX1, and DUOXA2 that was similarly linked to DUOX2[174]. In *B. glabrata,* two DUOXA1 genes were identified. A putative homolog of the DUOXA2 gene was not identified even though DUOX2 seems to be present. When produced, $O_2.^-$ is an unstable radical that spontaneously dismutates to H_2O_2, yet enzymes that speed this up, particularly superoxide dismutases, are universally present in the Metazoa. In *B. glabrata*, both *sod1* and *sod 2* are present. Hydrogen peroxide is both more persistent than $O_2.^-$ and better able to cross membranes, and is capable of inflicting damage directly to organic molecules. Unsurprisingly, it is the substrate for many additional enzymes involved in either biosynthesis of additional oxidizing compounds or in detoxification. The snail genome encodes all of those that we sought: glutathione peroxidases (GPx; 3 genes), glutathione reductases (GR; 1 gene) and glutathione S transferases (GST; 22 genes), thioredoxin peroxidases (TRx; 5 genes) and reductases (TR; 1 gene), many additional peroxidases

(>38 potential loci), catalase (1 gene), arginases (2 genes) and nitric oxide synthases (3 genes) (see Table 30 for gene description).

The three main pathways for the elimination of reactive oxygen species (ROS) involve reduced glutathione (GSH), thioredoxin and catalase[175]. Thioredoxin (15 genes in *B. glabrata*), a protein that reduces ROS levels, can be regenerated by thioredoxin reductase (TR) using the metabolite NADPH. GSH, which can also be regenerated by NADPH via glutathione reductase (GR), is derived from the metabolites glutamate and cysteine. GSH acts directly, eliminating ROS through the action of glutathione peroxidase (GPx) and glutathione *S*-transferase (GST). Catalase also acts directly on ROS and resides predominantly in peroxisomes. Whereas both thioredoxin and GSH pathways rely heavily on NADPH production for sustaining their activity, catalase acts independently of NADPH. Glutathione *S*-transferases (GSTs) are best known for their ability to catalyze the conjugation of the reduced form of glutathione (GSH) to xenobiotic substrates for the purpose of detoxification. The GST superfamily consists of three families: the cytosolic, mitochondrial, and microsomal[176-178]. Cytosolic GSTs are divided into 13 classes based on their structure: alpha, beta, delta, epsilon, zeta, theta, mu, nu, pi, sigma, tau, phi, and omega. Mitochondrial GSTs are in class kappa[179]. Glutathione is involved in ROS detoxification and its increase may be part of an adaptive response to oxidative stress. Glutathione metabolizing enzymes including glutathione-S-transferase (GST) and glutathione reductase (GR) play key roles in these processes[180]. In *B. glabrata*, 22 genes of GST were identified. All three families - cytosolic (20 genes), mitochondrial (1 gene) and microsomal (1 gene) - are present in *B. glabrata.* Among the cytosolic GSTs, we identified 6 different classes: zeta, theta, mu, pi, sigma, and omega. Nitric oxide is involved in signaling and thereby serves to maintain homeostasis, but, when levels become high, it reacts with the anion superoxide yielding destructive peroxynitrite and nitrogen dioxide. In this way, it can enhance the defensive potency of leukocytes. *B. glabrata* encodes at least three nitric oxide synthases (NOS). And since arginine serves as substrate for NOS, regulation of its levels will influence NO production. It was clear that the snail also encodes arginase. NOS enzymes depend on nitric oxide interacting protein (NOSIP) to be active. Two genes of NOSIP were identified in the *B. glabrata* genome (Supplementary Data 21). It remains to be ascertained which cellular functions are served by which ROS and RNS, but it is probable that both homeostatic and aggressive defense pathways will be affected.

Supplementary Note 18. Antimicrobial peptides and proteins in *Biomphalaria glabrata.*

Along with phagocytosis (and associated hydrolytic enzymes) and the production of a variety of intermediate reactive oxygen species, antimicrobial peptides (AMPs) and proteins are important effectors of innate immune systems that are found in virtually all kingdoms of life. To date, 2600 AMP molecules are present in the Antimicrobial Peptide database (APD, available at http://aps.unmc.edu/AP/main.php/). Most of them are small cationic (less than 10 kDa) amphipathic, gene-encoded peptides that differ considerably in amino acid sequence and structural conformation[181,182]. They share the ability to interact with microbial membrane and promote pore formation, membrane thinning or lipid bilayer disruption[183].

To date, investigations of mollusc AMPs mainly focused on marine bivalves and resulted in the characterization of several AMP families. In mussels, cysteine-rich families of peptides were identified. They were called defensins, mytilins, myticins and mytimycins[184,1854]. In oysters, defensins, big defensins and proline-rich AMPs were characterized[186]. The presence of AMPs in *B. glabrata* and more generally in gastropods had not previously been reported, while several studies did identify and functionally characterize antimicrobial proteins. In particular, a LBP/BPI (BgLBP/BPI1) was identified[187] and shown to display an antibacterial and a potent anti-oomycete activity[188]. A β-pore forming toxin named biomphalysin with hemolytic and anti-schistosomal activities was characterized[189]. In addition, pre-genomic gene identification studies[190] also identified mRNAs showing similarities with achacin from the giant African snail *Achatina* *fulica*[191] and aplysianin from *Aplysia* *kurodai*[192,193]. These molecules shared 48% identity and displayed antimicrobial activities and an amino-oxidase domain (pfam01593). It was demonstrated that the antimicrobial activity of achacin is associated to an L-amino acid oxidase activity[194]. In the present work we searched the *B. glabrata* genome for putative AMPs as well as putative family members of proteins previously shown to display antimicrobial activities.

Amino acid sequences representative of all families of invertebrate AMPs (http://aps.unmc.edu/AP/main.php) were applied query all *B. glabrata* transcriptomic databases available using local TBLASTN software. This first step allowed the identification of different transcripts encoding precursors for AMPs of the macin family that combine antimicrobial and nerve repair activities[195]. Considering these similarities, these putative AMPs were called biomphamacins. The *B. glabrata* transcripts encoding putative achacins/aplysianins were identified using BLASTX. The corresponding precursors were named BgAchacins. The genome was then extensively mined for genes encoding biomphamacins, BgAchacins and genes encoding the different molecules previously identified (BgLBP/BPI, biomphalysin). Exhaustive BLAST searches suggested that achacins, LBP/BPIs, macins and biomphalysins are present in the genome sequence, while cecropins, attacins and defensins were absent (see Supplementary Note 18). To improve the sensitivity of these genome searches, profile hidden Markov models from the Pfam database representing known anti-microbial peptide domains were used to search predicted open reading frames using HMMer3 (version 3.0), according to Morris *et al*. [196]. Models for Attacin_C (PF03769), Attacin_N (PF03768), Cecropin (PF00272), Defensin_beta (PF00711), LBP_BPI_CETP (PF01273), and Macin (PF14865) based on full alignments were used. Achacins and biomphalysins do not have models in Pfam and no searches were performed for these peptides.

Tissue-specific transcriptomic databases were mined to assess tissue localization of gene transcripts. Expression of candidate genes was analyzed by Q-RT-PCR in bacterial Gram-negative, Gram-positive or fungi immune-challenged snails[151].

Extensive search of the *B. glabrata* genome for antimicrobial peptides and proteins revealed the existence of multigenic families of the previously documented proteins LBP/BPIs, achacins and biomphalysins and allowed identification of a family of macin-type AMPs. Five LBP/BPIs positioned on 4 different scaffolds as well as two achacins positioned on two different scaffolds were annotated. Similarly, while a single biomphalysin was recently characterized[189], the genome revealed the existence of 21 biomphalysins positioned on 19 different scaffolds. Regarding AMPs, only a single gene family of the macin type was encountered, comprised of seven members named biomphamacins. Supplementary Data 22 lists the different scaffolds identified for the corresponding genes.

Profile hidden Markov Models searches for divergent anti-microbial peptides using HMMer3 with Pfam models representing known anti-microbial peptide domains obtained matches with small E-values (independent E-value threshold < 0.01) only for LBP_BPI_CETPs (6 hits) and Macins (10 hits; Supplementary Figure 34a). Several of these matches were located in clusters on scaffolds LG18i_random_Scaffold2999, LGUN_random_Scaffold1023, LGUN_random_Scaffold3131, and LGUN_random_Scaffold4830), consistent with location in gene clusters. In contrast, the smallest E-value for defensin was 0.07. This prediction of a defensin on LGUN_Random_Scaffold157236 contained just 3 cysteine residues and thus did not match the expected six-cysteine residue pattern. Additionally, the corresponding bit scores were small (Supplementary Figure 34b). All other E-values were larger than 0.01.

As expected, transcripts of most of these candidate genes are present in various organs including the immune-relevant albumen gland and hematopoietic organ (Supplementary Data 23). Substantial changes in expression levels after an immune challenge were observed for a subset of these candidates including all LBP/BPIs, three biomphamacins, and nine biomphalysins (Supplementary Data 23), further supporting their involvement in antimicrobial response.

The genome analysis reveals that the *B. glabrata* antimicrobial toolbox presents particular features as compared with other mollusc or invertebrate species. Firstly, the diversity of molecules identified in *B. glabrata* is much lower than in bivalve molluscs in which numerous families of AMPs (mytilin, myticin, defensin, big defensins, mytimacin, …) were characterized[184-186]. We identified a multigenic family of at least seven members encoding cysteine rich cationic peptides of the macin family that we named biomphamacins. Because antimicrobial molecules are known to show poor primary structure conservation across animal phyla, it may be challenging to identify AMPs using sequence similarities. Consequently, HMMer3-based searches were developed and applied (by the group of Papenfuss in Australia) and this complementary bioinformatics approach confirmed the initial results (from the group of Mitta in France) by indicating a lack of AMP diversity in *B. glabrata*. Finally, we also used a biochemical approach dedicated to the research of such molecules. This classical procedure for the identification of cationic AMPs was successful in numerous animal models[184,185,197-199] but confirmed the absence of additional peptides in *B. glabrata* (G. Mitta, personal communication).

In contrast to this reduced arsenal in AMPs, the *B. glabrata* genome contains multigenic families of antimicrobial proteins. We identified five members of the potent anti-bacterial and anti-oomycete proteins LBP/BPI[188]. An extensive search of invertebrate genomes and transcriptomes reveals that LBP/BPIs are absent from important invertebrate phyla such as arthropods, and present in molluscs (gastropods, bivalves and cephalopods), annelids, echinoderms, cnidarians, nematods and poriferans. Interestingly, most species, including molluscs possess one or two LBP/BPIs, suggesting that the five LBP/BPI encoding genes of *B. glabrata* result from recent serial duplications.

We also identified a multigenic family encoding aerolysin-like molecules which belong to the pore forming toxin superfamily. These molecules usually produced by bacteria were probably acquired by horizontal transfer in a few animal and vegetal species[189,200-203]. They were present in other gastropods like *Aplysia* but have not been identified in bivalve molluscs. Indeed, a similarity search and an Interproscan screening on aerolysin domain reveal that they are absent from genomic and transcriptomic databases from *Crassostrea gigas* and *Mytilus* databases but present in the *Aplysia* genome. This suggests that aerolysin-like molecules were acquired during evolution and conserved in some animal species in which gene expansion occurred to acquire new potential functions. This is probably the case in snails and more particularly in *B. glabrata* in which biomphalysins clearly plays a role in anti-trematode defense[189] and could represent a new immune repertoire against snail pathogens.

Altogether these results suggest that while the arsenal of small antimicrobial peptides (AMPs) appears reduced in *B. glabrata* as compared with other invertebrate species, the antimicrobial arsenal also comprises several multigenic families encoding antimicrobial proteins that appear to have diversified or to have been acquired during evolution.

Supplementary Note 19. Characterizing unknown transcripts from *B. glabrata*

A number of studies have made use of microarrays to assess the large-scale expression patterns of *B. glabrata* snail transcripts associated with exposure and infection by *Schistosoma* mansoni and other digenean trematodes[115,150,204]. While these studies have formed the foundation for numerous investigations into those factors that define compatibility between snails and trematodes, the array (GEO platform GPL9483) holds probes that are not currently associated with any defined gene or protein. This fact makes these unknown factors interesting, as they potentially represent unique molecules that are in some way important for snail resistance to infection. We focused on expanding our understanding of transcripts that appeared on microarray analyses of responses of snails resistant to trematodes and, when assessed for homology to known sequences, returned a result of 'unknown'. We were particularly interested in those that displayed altered expression following trematode challenge. In total, we assessed 243 unknown transcripts associated with a custom microarray designed to assess immune factors expressed by *B. glabrata*[150].

In order to assign new identity to these unknown transcripts we used a combination of BLASTX and BLASTN of existing KEGG databases. Of the total transcripts analyzed, 42 previously unknown transcripts were assigned new, 'known' identifications based on improved BLAST search results of the fully or partially annotated genome sequence. Fifty-two transcripts were fully or partially annotated but continued to return BLAST results of hypothetical protein or predicted protein with no known function. Twenty-eight transcripts were fully or partially annotated that shared the highest sequence identity with bacterial genes. The remaining 121 transcripts were only partially annotated, and all returned BLAST results of 'no known similarity'.

Analysis of the 42 newly-identified transcripts against past microarray results[115,150,204] allowed for assessment of the expression of each transcript in M-line and BS-90 *B. glabrata* snails following digenean trematode challenge with either *S. mansoni* or *Echinostoma paraensei,* over the entire course of intramolluscan development of each parasite species (32 days).

Supplementary Data 24 contain the original microarray transcript ID, the microarray probe used, and the new BLAST results colour coded to correspond to the 42 identified transcripts (purple), the hypothetical proteins (green) and the definitive bacterial transcripts (orange), unidentified transcripts remain in white. Under the conditions used in the original microarray analyses (fold change in expression > 1.5), many of these transcripts do not significantly change in expression following challenge with either parasite, in either snail strain. However, a number are significantly increased or decreased in expression over time in response to trematode challenge or infection. For example, a TLR 13-like transcript (BGC04545), a leucine-rich-repeat-containing transcript (BGC00588) and a hemagglutinin/amebocyte aggregation factor (BGC02817) all display high homology to known immune factors in invertebrates, and each is increased in expression in response to trematode challenge, particularly in BS-90 strain *B. glabrata* (Supplementary Data 25). The complete list of newly identified transcripts from these array studies spans a number of physiological and metabolic processes. When taken in the context of this genome annotation, these results provide comprehensive assessments of the expression of previously unknown features. The original microarray studies were designed to facilitate longitudinal studies of transcript expression during the trematode development within in the snail, as well as during the initial stages of challenge in which a snail may successfully defend itself against infection (BS-90 *B. glabrata -S. mansoni*). Thus, novel gene annotation information is now accessible with respect to the transcript identified as part of this re-analysis of the microarray features.

Supplementary Note 20. Epigenetic toolbox of *B. glabrata.*

In parallel with genetic inheritance that is based on the sequence of nucleotide bases in DNA, probably all eukaryotes have a second inheritance system that transmits epigenetic information that is not encoded in the DNA sequence. Here we focus on a relatively narrow definition of epigenetics i.e. all information on the status of gene activity that is heritable, for which changes in this information are reversible and not based on the DNA sequence. Epigenetic inheritance is based on at least four information carriers: chemical modifications of DNA (such as cytosine methylation), modifications of histones and other chromatin proteins, non-coding RNA and the location of a gene in the nucleus. Covalent modifications of DNA and chromatin proteins are called a "chromatin-marking system"[205].

Coding sequences were extracted from the genomic sequence Biomphalaria-glabrata-BB02_SCAFFOLDS_BglaB1.fa using the annotation gff file Biomphalaria-glabrata-BB02_BASEFEATURES_BglaB1.0.gff3 (both from https://www.vectorbase.org/download/). Repeats in "biomphalaria-glabrata- bb02repeatslibmphalaria-glabrata-bb02repeatslib.fasta" downloaded from https://www.vectorbase.org/download/biomphalaria-glabrata-bb02repeatslib. Western blots were performed as previously described[206]. Tissue material was taken from the headfoot region of *B. glabrata* snails derived from Brazil (different from BB02) and maintained at Perpignan. BS-Seq was done according to the Lister protocol[207] (sequence 2 forward strands only) by GATC-biotech and data were analyses with BSMAP 1.0.0[208], and custom scripts. Annotation files are available at http://ihpe.univ-perp.fr/acces-aux-donnees/, scroll to "Reference transcriptome" (http://2ei.univ-perp.fr/telechargement/transcriptomes/transcriptsBre1et2.zip)

DNA methylation: The presence of 5-methyl-cytosine and traces of 5-hydroxy-methyl-cytosine was described earlier[209,210]. Homology searches revealed the presence of homologues to DNA methyltransferases (DNMT) 1 and 2, and methyl-CpG-binding domain protein 2/3 (MBD2/3) (Supplementary Data 26). The full-length sequences of DNMT2 and MBD2/3 were confirmed by PCR amplification as well as the catalytic domain of DNMT1. No homologues to Dnmt3 or Ten-eleven translocation methylcytosine dioxygenase (TET) could be detected confirming earlier results[209]. Due to spontaneous deamination of cytosine, methylation of CG dinucleotides leads in evolutionary time scale to underrepresentation of these CpG pairs in the genome. Only in regions devoid of methylation or with less methylation, CpG pairs occur in the statistically expected frequency. We have used this feature previously to analyse the Bg ESTs and found that there are two types of genes, those with high and those with low methylation. This was confirmed using the genomic sequences of predicted exons in the genome (Supplementary Fig. 35) and it is similar to what is found in other molluscs. GC content and CpG observed/expected (CpGo/e) ratios are not statistically significantly correlated (Supplementary Fig. 35) but genes whose coding regions possess high CpGo/e and those with low CpGo/e are clustered in the genome (Supplementary Fig. 35). Using a sliding window analysis we detected 78,460 presumably unmethylated CpG islands (CGI). In contrast to many other organisms, these CGI are atypical and not preferentially located in the 5' region of genes. They appear to be clustered in high GC content (>35%) regions ("isochores") (Supplementary Fig. 36). In many species, transposable elements and other repetitive sequences are heavily methylated. We scanned the predicted repeats of the Bg genome for traces of methylation using the CpG observed/expected ratio and surprisingly found that most of the repeats are likely to be unmethylated (Supplementary Fig. 37). To confirm our prediction, we performed whole-genome bisulfite-sequencing (BS-Seq) for pooled DNA of foot tissue of ten adult *B. glabrata* individuals. For further analysis we considered only those contigs of the Bg genome assembly that were at least 5 kb in length. Methylation is predominantly found in CpG pairs. 4.3% of CpG sites are methylated

with 0.5% showing complete methylation (Supplementary Fig. 37). Average methylation in CpG sites is 13.5%. We arbitrarily defined “high-methylated” regions as those regions in which there were at least 10 Cs no more than 2 kb apart, each at least 80% methylated, and covered by at least 8 reads. They cover only 20.2% of the genome but contain 37,673 (57%) of the 66,475 annotated genes confirming our CpG o/e based prediction (61%). Highly-methylated regions are extremely poor in repeat content (0.02% of the repeats are located there). Highly conserved proteins CEGMA core 458 data (http://korflab.ucdavis.edu/datasets/cegma/core/core.fa)[211] contain 2,748 proteins sequences for which we found a total of 371 matches in *B. glabrata*. 81% of these matches are located in high methylated regions. We then analysed RNA-Seq data for 12 tissues provided by the genome initiative and used Normfinder[212] to calculate range score for 63,030 genes. When this score is low, gene expression is stable suggesting house-keeping function. A high score value indicates tissue-specific differences. A Kolmogorov-Smirnov test showed that genes in highly methylated regions (33,975) have a more stable expression (p value < 0.0001, alpha = 0.05). Taken together, *B. glabrata* genomic methylation shows a mosaic type DNA methylation with a highly methylated compartment and a lowly methylated compartment. The highly-methylated compartments is essentially free of repeats and contains roughly 60% of the known genes, which probably do not evolve rapidly and have in general a stable expression pattern among tissues.

Histones and Histone modifying enzymes: The occurrence of core histones and a linker histone was deduced from the presence of genes coding for these proteins (Supplementary Data 26). We confirmed the presence of histones H4 and H3 and their most prominent modifications (H3K27me3, H3K9ac and H4K20me3) by Western blots (Supplementary Fig. 38). H3K9me2 and H3K4me3 have been shown to be detectable by immunostaining before[9]. Based on the genomic sequence and RNA-Seq data, the deduced amino acid sequence of the linker histone has a moderate similarity to histones H1/H5 of other species. The core regions of putative histones H2A are conserved but no H2B could be found. Three orthologues of H3 were identified but only one spans the canonical H3 sequence and all possess the expected modification sites (Supplementary Fig. 39). CENP-A is a specific H3 isoform found in the constitutive heterochromatic centromere. No gene for a putative CENP-A was identified in *B. glabrata*. Histone H4 is highly similar to other species but only one full-length cDNA was identified (Supplementary Fig. 39). Homologues of most histone modifying enzymes such Histone deacetylases (HDAC), Histone acyltransferases (HAT), Histone methyltransferases and demethylases (HMT/HDMT) can be found (Supplementary Data 26) but complexity is surprisingly low and for some enzyme classes such as Class III HDACs (Sir2-likes) for instance, no gene seems to be present.

Chromodomain proteins: Histone methylation is known to create binding sites for a number of non-histone chromatin proteins. The chromodomain of heterochromatin proteins 1 (HP1), for instance, binds to methylated H3K9[213]. The three known HP1 isoforms have numerous functions, including participation in transcriptional elongation, centromeric sister chromatid cohesion, telomere maintenance, and DNA repair, however, HP1 is primarily known for being a hallmark of transcriptional inactive heterochromatin[214]. A homologue of HP1-alpha can be found among the predicted *B. glabrata* genes (Supplementary Data 26). HP1 is particularly interesting because it provides a link between chromatin structure and the RNAi pathway. In model organisms, disruption of RNAi leads to changes in HP1 deposition, histone methylation and heterochromatin formation[215].

Non-coding RNA: Recent work in model organisms such as *Schizosaccharomyces pombe, Arabidopsis thaliana* and *Drosophila melanogaster* has shown that non-coding RNA (ncRNA) can provide a framework for the recruitment of chromatin-modifying factors. In these model organisms, ncRNA are processed through the RNAi machinery. Disturbing the RNAi pathway leads to changes in heterochromatin formation[215]. The classical RNAi pathway is present in *B. glabrata*[68]. One of the

best-analyzed complexes that links ncRNA to heterochromatization is the RNA-induced transcriptional silencing (RITS) complex in fission yeast that contains the Chp1, Ago1 and Tas3 proteins[216]. In this model, Rdp1-containing RNA-dependent RNA polymerase complex (RDRC) and Dicer (Dcr1) process repeat transcripts into small-interfering RNA (siRNA) that bind to Ago1 and probably direct RITS to nascent repeat transcripts, recruiting H3K9 methylase[217]. Homologues to Ago1, but not to *S. pombe* chromodomain protein Chp1 or RITS complex subunit 3 (tas3) that link Ago1 to the RITS complex are present in *B. glabrata*. Also homologues of *Drosophila melanogaster* vasa or piwi that are classes of proteins linked to RNAi via binding and cleaving of RNA seem to be absent from *B. glabrata*. These proteins are essential for germ cell development and ES cell maintenance[216,218] and it is tempting to speculate by what genes these functions might be replaced in *B. glabrata*.

Taken together, the epigenetic toolbox of *B. glabrata* appears to be surprisingly simple. That might indicate that epigenetic pathways are less redundant compared to other species and could constitute a chokepoint for vector control, or that orthologues for conserved gene functions have genetically diverged so much that they cannot be identified by homology-based and/or conserved domain searches.

Supplementary Note 21. miRNA/piRNA pathway genes and conserved miRNAs in *B.glabrata*

Increased insights into regulation of gene expression through small non-coding RNAs like microRNA (miRNA) have led to the realization that miRNAs also function in establishing and maintaining parasite infections in their hosts[219]. Parasite-mediated modification of host miRNA levels, or parasite contributed miRNA can manipulate the normal regulation of host biology to benefit the parasite, and determine success of failure of the infecting parasite. In consideration of the importance of miRNA for regulation of the general biology of *B. glabrata* and for shaping responses to changing environmental conditions, as well as the potential for modification of miRNA regulation by parasites like *S. mansoni* to hijack the host biology and achieve host compatibility, we investigated the miRNA and piRNA pathway genes in the *B. glabrata* genome and characterized *in silico* conserved *B. glabrata* miRNAs (mature and precursors) and their target genes in *B. glabrata* genome and transcriptome.

Prediction of miRNA and piRNA pathway genes in B. glabrata genome: We screened the *B. glabrata* genome (BB02 strain genomic scaffold sequences, BglaB1 assembly) for miRNA and piRNA pathway gene candidates using the BLASTP tool. The query sequences used for the genome screening were conserved genes involved in miRNA and piRNA biogenesis from *D. melanogaster* (FlyBase), *A. californica* (NCBI) and *L. gigantea* (http://genome.jgi-psf.org/Lotgi1/Lotgi1.home.html). Once putative proteins were found, the respective genome sequences and protein sequences that matched were retrieved from BglaB1.0 geneset. To search putative orthologues the BLASTP was performed against NCBI non-redundant proteins sequences (nr) database using the amino acid sequences of the proteins. : Prior to searching for miRNAs in the *B. glabrata* genome, we identified putative proteins involved on biogenesis of the miRNA and piRNA based on sequence similarity. Previously characterized proteins involved in predicted miRNA and piRNA pathways (amino acid sequences) from *D. melanogaster*, *L. gigantea and A. californica* were used for BLAST searches of the *B. glabrata* genome database and ESTs deposited in the NCBI database. This yielded 9 sequences with high similarity to orthologs representing putative miRNA and piRNA pathway proteins (Supplementary Data 27).

Computational prediction of B. glabrata microRNA genes: We used an integrated approach to search the potential conserved miRNAs in available *B. glabrata genome* and transcriptome sequences. This approach was based on previous method used successfully to find putative miRNAs in *S. mansoni* genome[220]. Briefly, we retrieved sequences that can form hairpin-like structures from *B. glabrata* genome (Biomphalaria-glabrata-BB02_SCAFFOLDS_BglaB1.fa) and transcriptome using Einverted (from EMBOSS) and BLASTn tools. The parameters for Einverted program were maxrepeat 95 nucleotides and score threshold 25. We also used Blastn to search hairpin-like sequences using all animal pre-miRNA sequences deposited in miRBase version 20.0 as queries. The e-value threshold used was 0.001, minimal match length 25 nucleotides and 80% of identity. We collected sequences with the length between 60 and 110 nt. To select for real miRNAs, the set of hairpin-like sequences were filtered in the following steps i.e MFE (Minimal free energy) filter, GC content filter, mature sequence homology filter, protein coding genes filter, noncoding RNAs filter and finally miPred classifier. The putative hairpin-like sequences obtained from EMBOSS and BLASTn tool were filtered using MFE(s) determined via RNAfold (Vienna RNA Package) with the following parameters: RNA secondary folding energy threshold -20 kcal/mol and with the options "-p -d2 -noLP"[221]. Secondly, these structures were filtered with GC content ranging from 30% to 65%. Additionally, all animal mature miRNAs deposited in miRBase (Version 20.0) were aligned with the sequence,accepting no more than 4 mismatches in whole mature miRNAs and 0 mismatches in the seed region (2–8 nt). In order to remove sequences similar to known protein-coding sequence we

compared the sequences with the transcripts (Biomphalaria-glabrata-BB02_TRANSCRIPTS_BglaB1.0.fa) using Blast. Other classes of non-coding RNAs (i.e., rRNA, snRNA, SL RNA, SRP, tRNAs, and RNase P) were removed by screening against the Rfam microRNA Registry (version 10.0)[222]. And finally, we used miPred to remove pseudo and false precursor miRNAs and retain only sequences classified as true microRNA precursors[223].

For further analysis in the identified *B. glabrata* pre-miRNAs we analyzed the following set of structural characteristics and thermodynamic parameters: Minimal Free Energy (MFE), Adjusted Minimal Free Energy (AMFE), Minimal Free Energy Index (MFEI), length, A content, U content, C content, G content, GC content, AU content, GC ratio, AU ratio, Minimal Free Energy of the thermodynamic ensemble (MFEE), Ensemble Diversity (Diversity), and frequency of the MFE structure in the ensemble (Frequency). The parameter adjusted MFE (AMFE) was defined as the MFE of a 100 nucleotide length of sequence and the minimal folding free energy index (MFEI) that was calculated by the following equation: $MFEI = [(AMFE) \times 100] / (G\% + C\%)$[224]. The diversity, MFE and frequency of the ensemble were measured using RNAfold as well as MFE of the secondary structures. The GC content and other structural characteristics were measured using Perl scripts.

Trinity software (version 20131110; Broad Institute and Hebrew University of Jerusalem;[125] was used as tool for reconstruction of transcriptomes from RNA-seq data with (Minimum assembled contig length to report 70) of *B. glabrata* organs: Ovotestis, Digestive gland, Stomach, Albumen gland, Heart/ APO, Terminal genitalia, Mantle edge, Kidney, Headfoot, Salivary glands, Central nervous system and Buccal mass (see Supplementary Data 1 for accession numbers of these data sets). Trinity combines three independent software modules: Inchworm, Chrysalis, and Butterfly, applied sequentially to process large volumes of RNA-seq reads. To identify miRNAs within the *B. glabrata* genome, we employed a similar approach as used by de Souza Gomes[220] which had identified conserved and non-conserved miRNAs in *S. mansoni* parasite. We identified a set of conserved miRNAs from the *B. glabrata* genome (Biomphalaria-glabrata-BB02_SCAFFOLDS_BglaB1) including clustered miRNAs, duplicated miRNAs, intronic miRNAs and intergenic miRNAs. Putative hairpin-like sequences in the *B. glabrata* genome, identified by using inverted EMBOSS and Blastn were used as the basis to uncover *B. glabrata* miRNAs with the conserved approach. From initially thousands of hairpin-like candidate sequences, this approach identified 95 pre-miRNAs and 102 mature miRNAs. This set of *B. glabrata* miRNAs genes was larger than the set of miRNAs of 60 pre-miRNAs and 64 mature miRNAs genes identified from another mollusc, *L. gigantea*, (JGl1 genome), previously deposited in miRBase (version 20.0). We expect that the number of miRNAs in *B. glabrata* will increase with analyses of future versions of *B,. glabrata* genome assemblies and additional high-throughput next generation sequencing datasets. The structural and thermodynamic characteristics of the *B. glabrata* miRNA are conserved and similar when compared to other miRNAs (Supplementary Data 28). Previous studies showed that the structural and thermodynamic features importantly facilitate prediction of miRNA and aid in distinction of true and false pre miRNAs. For instance, the 95 *B. glabrata* precursors miRNAs identified in this study folded into stem-loop structures that like previously characterized true miRNAs displaying Minimal Free Energy ranging from -21,26 kcal/mol to -70,70 kcal/mol (Supplementary Data 28 and Supplementary Fig. 40).

Our analyses show that *B. glabrata* miRNAs were distributed across intronic and intergenic regions of the genome. Of the 95 *B. glabrata* precursor miRNAs, 15 were locate to intronic regions (bgl-miR-71, bgl-miR-2a-1, bgl-miR-2a-2, bgl-miR-2b, bgl-miR-2d, bgl-miR-3286, bgl-miR-153, bgl-miR-7398j, bgl-miR-92a-1, bgl-miR-190, bgl-miR-92a, bgl-miR-7908-1, bgl-miR-7908-2, bgl-

miR-96 and bgl-miR-214) while the remaining 80 were distributed across intergenic regions (Supplementary Data 28).

The location of *B. glabrata* miRNA bgl-mir-190 in the second intron of BGLB001984, a putative talin gene seems a conserved feature: mir-190 miRNA hasoccurs in introns of talin genes across deuterostome[230] and protostome species including *L. gigantea*, another mollusc. Similarly, we located bgl-miR-153 in an intronic region of BGLB012541, a putative phosphatase gene present in *B. glabrata* genome. This miRNAs is present in an intronic region of the tyrosine phosphatase (PTPRN2) genes of some Deuterostome species, such as *Danio rerio*, *Anolis carolinensi, s Equus caballus*, *Canis familiaris* and humans,[231]. Supplementary Figure 40 shows the secondary structure of the miRNA genes in the clusters bgl-miR-71/2a-1/2d/2b/2a-2 and bgl-miR-745a/745b respectively. Thirtythree *B. glabrata* miRNAs were identified as part of the gene clusters such as bgl-miR-100, bgl-let-7, bgl-miR-125, bgl-miR-1175, bgl-miR-750, bgl-miR-124, bgl-miR-124c, bgl-miR-1990, bgl-miR-1986, bgl-miR-1994a, bgl-miR-1994b, bgl-miR-216a, bgl-miR-216b, bgl-miR-12, bgl-miR-219a, bgl-miR-219b, bgl-miR-29a, bgl-miR-29b, bgl-miR-71, bgl-miR-2a-1, bgl-miR-2a-2, bgl-miR-2b, bgl-miR-2d, bgl-miR-745a, bgl-miR-745b, bgl-miR-87b-1, bgl-miR-87b-2, bgl-miR-92a, bgl-miR-92a-1, bgl-miR-92, bgl-miR-92b, bgl-miR-9a and bgl-miR-9b. In the majority of primary structures of *B. glabrata* mature miRNAs, the nucleotide Uracil was present in the first position of 5' stem. This characteristic has been shown in mature miRNAs in general since this nucleotide plays an important role in recognition of the molecule by the RISC complex (RNA-induced silencing complex) (Supplementary Figure 41, Supplementary Data 29).

Alignment and Phylogenetic analysis of miRNAs: The pre-miRNA sequences were aligned using ClustalX 2.0 and RNAalifold[221,225]. To perform the alignments of the pre-miRNAs, parameters were adjusted to: gap opening 22.50 and gap extension 0.83. The mature miRNA sequence logos were generated using WebLogo 2.8.2 at http://weblogo.berkeley.edu/logo.cgi[226]. The phylogenetic trees were inferred using the Neighbor-Joining method[227]. The percentages of replicate trees in which the associated taxa clustered together in the bootstrap test (2000 replicates) are shown next to the branches. The tree is drawn to scale, with branch lengths in the same units as those of the evolutionary distances used to infer the phylogenetic tree. The evolutionary distances for pre-miRNAs sequences were computed using the Kimura 2-parameter method and are in the units of the number of base substitutions per site. The phylogenetic analyses were conducted in MEGA5[58]. This section presents alignments and phylogenetic analysis for *B. glabrata* miRNAs and the orthologous miRNAs from animal species, such as e.g. *L. gigantea* (Mollusca). We consider the conservation of sequence and evolutionary distribution of pre-miRNAs. Moreover, we analyzed the conservation of their primary and secondary structure highlighting the precursors, the seed region and mature sequences.We also compared the structures of the pre-miRNAs with their closest orthologs and the position of the mature miRNAs in the precursor sequences. We separated the *B. glabrata* miRNAs in groups based on their conservation in different levels in the animal phylogeny/evolution: Animal-specific miRNAs, Bilateria-specific miRNAs, Protostome-specific miRNAs, Lophotrochozoa-specific miRNAs and Mollusca-specific miRNAs. Some *B. glabrata* miRNAs were not studied in this manner due to evidence of highly complex evolutionary history (bgl-miR-134, bgl-miR-1542, bgl-miR-2354, bgl-miR-3134, bgl-miR-3286, bgl-miR-4079, bgl-miR-4864, bgl-miR-4966, bgl-miR-5607-1, bgl-miR-5607-2, bgl-miR-6833, bgl-miR-723, bgl-miR-199a, bgl-miR-214, bgl-miR-7398j, bgl-miR-7428, bgl-miR-7620a-1, bgl-miR-7620a-2, bgl-miR-7908-1, bgl-miR-1260 and bgl-miR-7908-2).

Animal-specific miRNAs: In this group we found a single miRNA, bgl-mir-100. This miRNA gene is conserved in the cnidarian species *Nematostella vectensis* and also in Bilateria, including deuterostome and protostome species. In bilateria species, this miRNA is part of the miR-

let-7/100/125b cluster which contains the miRNAs bgl-mir-let-7, bgl-mir-100, bgl-mir-125b. See Supplementary Figures 42,43.

Bilateria-specific miRNAs: Thirtysix *B. glabrata* miRNas were found in this category, including: bgl-let-7, bgl-miR-125, bgl-miR-125b, bgl-miR-137, bgl-miR-153, bgl-miR-184, bgl-miR-190, bgl-miR-193, bgl-miR-1a, bgl-miR-216a, bgl-miR-216b, bgl-miR-29a, bgl-miR-29b, bgl-miR-33, bgl-miR-375, bgl-miR-92, bgl-miR-92a, bgl-miR-92a-1, bgl-miR-92b, bgl-miR-9a, bgl-miR-9b, bgl-miR-124, bgl-miR-124c, bgl-miR-133, bgl-miR-219a, bgl-miR-219b, bgl-miR-219c, bgl-miR-34, bgl-miR-7, bgl-miR-72, bgl-miR-2001, bgl-miR-252a, bgl-miR-252b, bgl-miR-281, bgl-miR-71 and bgl-miR-278. See Supplementary Figures 44-54 for the phylogenetic distribution of the *B. glabrata* miRNA precursors and bilaterian orthologs as well as conservation of primary and secondary structures.

Protostome-specific miRNAs: This group encompasses 18 *B. glabrata* miRNAs bgl-bantam, bgl-miR-1175, bgl-miR-279, bgl-miR-2a-1, bgl-miR-2a-2, bgl-miR-2b, bgl-miR-2d, bgl-miR-315, bgl-miR-317, bgl-miR-750, bgl-miR-981, bgl-miR-1000, bgl-miR-12, bgl-miR-277a, bgl-miR-284, bgl-miR-8, bgl-miR-87b-1 and bgl-miR-87b-2. See See Supplementary Figures 55-60 for the phylogenetic placements of of the *B. glabrata* miRNA precursors and conservation of primary and secondary structure.

Lophotrochozoa-specific miRNAs: In this group we found 14 *B. glabrata* miRNAs: bgl-miR-36a, bgl-miR-67-1, bgl-miR-67-2, bgl-miR-96, bgl-miR-96a, bgl-miR-96b, bgl-miR-1990, bgl-miR-1992, bgl-miR-1994a, bgl-miR-1994b, bgl-miR-745a, bgl-miR-745b, bgl-miR-1993 and bgl-miR-2176. See See Supplementary Figures 61,62 for the phylogenetic distribution of the *B. glabrata* miRNA precursors and conservation of primary and secondary structures.

Mollusca-specific miRNAs: In this group we found 5 *B. glabrata* miRNAs: bgl-miR-1984, bgl-miR-1985, bgl-miR-1986, bgl-miR-1991 and bgl-miR-2722. See See Supplementary Figures 63,64 for the phylogenetic placement of these *B. glabrata* miRNA precursors and conservation of primary and secondary structures.

Conservation of mature miRNAs: *B. glabrata* versus *L. gigantea:* Comparative analyses of the 64 *L. gigantea* mature miRNAs and 60 *L. gigantea* precursor miRNAs, present in miRBase showed 53 highly similar *B. glabrata* mature miRNAs. Most , displayed similarity with L. gigantea mature miRNAs. Most of these displayed over 90% sequence identity, and 25 were 100% identical. See Supplementary Figures 65. Identification of conserved *B. glabrata* precursor miRNAs from RNA Sequencing: The identification of miRNAs from high-throughput next generation sequencing data has emerged as a vital approach to discover (non-)conserved miRNAs in various organisms. We identified 36 conserved precursor miRNAs in the RNAseq libraries from several organs of *B. glabrata* (See methods). Some miRNAs, such as miR-96, were encountered in transcriptomes of all tissues/organs, others, like bgl-miR-71 and miR-87b-1 precursor miRNAs were only encountered from the RNAseq library derived from the terminal genitalia. Supplementary Data 30 shows the distribution of precursor miRNAs amog the 12 different *B. glabrata* organs/tissues.

We collected 3′ UTR sequences from GFF3 file Biomphalaria-glabrata-BB02_BASEFEATURES_BglaB1.0.gff3 in VectorBase to search the predicted microRNA target genes in *B. glabrata* genome. The miRanda software (version 3.3a, 2010) was used with the following parameters and conditions: a gap opening penalty of − 8, a gap extension penalty of − 2; match with minimum score threshold 110, target duplex with maximum threshold free energy – 15 kcal/mol, scaling parameter 3 for complementary nucleotide match score, counting from the miRNA 5′ end, and demand strict 5′ seed pairing on between 2 and 9 nucleotides[228,229]. *B. glabrata* miRNAs may play crucial roles in regulation of gene expression in many biological processes of the snail. The *B. glabrata* genes targeted by bgl-miRNA genes were predicted using the software miRanda,

using similar parameters, but with less stringent criteria to those used to predict miRNA target genes for *S. mansoni* and human since the 3' UTR sequences are not well annotated in the current version of the *B. glabrata* genome (265 3' UTR)[220,229]. The adjusted miRanda parameters maintained 100% complementarity between the seed region of each bgl-miRNA gene and the 3′ UTR sequence targeted. Fortyfive bgl-miRNAs targeted at least one 3′ UTR sequence; a number of miRNAs had a multiple target sites within the same target gene (e.g. bgl-miR-1993 targeted different positions in the 3′ UTR sequence of BGLB008897 gene); and a number of 3′ UTR sequences were targeted by more than one mature bgl-miRNA (e.g. bgl-miR-124-3p, bgl-miR-125, bgl-miR-284 and bgl-miR-29b-3p which targeted 3′ UTR sequence of BGLB002915 gene) (Supplementary Data 43).

In summary, Screening of the miRNA and piRNA pathway genes in B. glabrata genome identified nine putative proteins involved on biogenesis of the miRNA and piRNA (Bgl-Argonaute, Bgl-Dicer, Bgl-Drosha, Bgl-Fmr1, Bgl-Partner-Dicer, Bgl-TDRD1, Bgl-PIWI, Bgl-Tudor-SN and Bgl-SPN-E) with high similarity with their orthologs from from *D. melanogaster*, *L. gigantea* and *A. californica.* Additionally, we identified conserved *B. glabrata* miRNAs (mature and precursors) and their target genes in *B. glabrata* genome and transcriptome, including clustered miRNAs, duplicated miRNAs, intronic miRNAs and intergenic miRNAs. Among thousands of the hairpin-like sequences we identified 95 pre-miRNAs and 102 mature miRNAs. The set of *B. glabrata* miRNAs genes discovered was larger than the set of miRNAs of identified genes in another mollusc, *L. gigantea*, which has deposited in miRBase up to date (version 20.0). The structural and thermodynamic characteristics of the *B. glabrata* miRNAs displayed very similar and conserved values compared to other miRNAs. Fifteen *B. glabrata* miRNAs were distributed across intronic regions and 80 across intergenic regions. One of the intronic miRNAs, bgl-mir-190 was identified in the second intron of the BGLB001984-RA mRNA, a putative talin gene, corroborating with the mir-190 distribution in other protostome species such as *L. gigantea*. Thirtythree *B. glabrata* miRNAs were part of gene clusters. Most *B. glabrata* miRNAs displayed conservation in primary (Uracil in the first position of 5' stem of *B. glabrata* mature miRNAs) and secondary structure involving the main part of the precursors, mature sequences and the seed region. Most of the *B. glabrata* precursor miRNAs demonstrated conserved phylogenetic distribution with their orthologs from animal species. Among all *B. glabrata* mature miRNAs identified, 53 displayed high similarity (25 showed 100% sequence identity) with mature miRNAs from *L. gigantea*. 36 different conserved precursor miRNAs were identified in 12 B. glabrata organ RNAseq libraries (Ovotestis, digestive gland, stomach, albumen gland, heart/ APO, terminal genitalia, mantle edge, kidney, headfoot, salivary glands, central nervous system and buccal mass). The *B. glabrata* genes targeted by bgl-miRNA genes were predicted; 45 bgl-miRNAs targeted at least one 3′ UTR sequence, a number of miRNAs had a multiple target sites within the same target gene and a number of 3′ UTR sequences were targeted by more than one mature bgl-miRNA.

The presence and conserved aspect of an miRNA regulatory system, as indicated by genome analysis further increases our options to study and manipulate snail biology and to investigate how how this system figures in the interactions between *B. glabrata* and *S. mansoni.*

Supplementary Note 22. Identification of novel *B. glabrata* microRNA and prediction of associated targets.

It is well established that in response to external stress stimuli, cellular modifications involve transcriptional, translational, post-translational, allosteric and microRNA regulation[232,233]. microRNA (miRNA) are short (18–23 nt), non-coding RNAs that are known to have central roles in regulating the post-transcriptional expression of mRNA transcripts. To date, more than 1200 human miRNA species have been predicted, many of which are conserved throughout evolution[233]. Current estimates indicate that miRNA may regulate up to 20–30% of an organisms genome, suggesting enormous potential that miRNA may hold widespread control of gene expression, including manipulation of this system by parasites[218]. We utilized a newly developed pipeline to predict both known (conserved) and novel miRNA sequences in the *B. glabrata* genome. Identification of these miRNA may provide information on the conservation of miRNA and their influence on translational regulation of gene expression in this snail species, potentially the miRNA regulatory system is targeted by parasites like *S. mansoni* to alter the biology of the snail in order to achieve an parasite-supportive environment inside the snail host.

To extend the repertoire of 95 identified conserved small regulatory RNAs (see Supplementary **Note** 21) from the *B. glabrata* genome, we performed an *in silico* prediction of miRNA within the *B. glabrata* genome scaffolds. We leverage a novel approach to miRNA classifier construction in which models are built dynamically and are targeted toward the species being studied. This differs from current precursor miRNA prediction methods, which attempt to produce generalized models, which are applicable to a large number of species. In order to generate a targeted model for a species, our precursor miRNA prediction approach generates data sets of real- and pseudo- miRNA from species that are closely related to the targeted species.

Our species-specific positive training datasets are built using version 19 of the miRBase database (http://www.mirbase.org). This database, and thus our overall set of positive training patterns, contains 20982 miRNA sequences across 193 species. Redundant sequences and sequences containing non-AGCU characters were removed from the dataset, resulting in a subset containing 19161 sequences. CD-hit[234] was then used to generate clusters of sequences within this dataset. Clusters were generated using a threshold of 80% sequence similarity, using default CD-hit parameters. Using these parameters, CD-hit generated 11668 clusters, 2607 of these clusters contain more than one sequence, while the largest cluster contains 34 sequences. In spite of the use of the lowest possible sequence similarity threshold that the CD-hit algorithm will allow, approximately half of all miRNA sequences in our dataset are not clustered with any other sequence.

Using the miRBase sequence dataset and the clusters described above, we develop positive training datasets for miRNA classification, which are targeted toward arbitrary species. These datasets were developed as follows: For a given positive integer ‘n’, the largest ‘n’ clusters are chosen from the CD-hit clustering results, then a representative sequence is chosen from each of these ‘n’ clusters. Each representative sequence is the sequence whose species is nearest our target species in terms of phylogenetic classification. In the event of multiple sequences within a cluster whose species are equally close to our target species in terms of phylogenetic classification, the sequence among these candidate representative sequences whose length is closest to the mean length of sequences within the cluster is chosen as the representative sequence for the cluster.

Negative training datasets are generated from the coding regions of a species' genomes. For a given target species, coding region sequence data from closely related species are downloaded from the European Nucleotide Archive, http://www.ebi.ac.uk/ena/. From these coding regions, sequences that resemble miRNA are extracted. Sequences are considered to resemble miRNA if they fold into a

hairpin secondary structure containing at least 18 stem pairs, a maximum base pair separation of 120, and a minimum free energy of at most -20 kCal/mol. Secondary structures are determined using the RNAfold package[221]. These folding criteria are commonly used for the determination of miRNA hairpin candidates[235].

For our MiRNA classification pipeline we have chosen the HeterMirPred[235] feature set for classification of *B. glabrata* miRNA using our targeted positive and negative training sets. HeteroMirPred is a highly cited example of a modern machine learning-based miRNAclassifier, which is available for download under an open source license. The feature set consists of 20 sequence and structural features. We have chosen to use a support vector machine (SVM) as our classifier as opposed to the ensemble classifier, which was found to be optimal by the original HeteroMirPred study, due to the ability of a SVM to provide clear probability estimates along with its determinations of real or pseudo-miRNA. These estimates allow us to rank results based on the probability that they are real or pseudo-miRNA and to parameterize our model for arbitrary recall thresholds, which is important for successful experimental validation of miRNA prediction results, as miRNA prediction class imbalance is on the order of 1:1000. We have used the LibSVM library[236] to parameterize and generate our SVM classification models.

We chose a prediction confidence threshold of 0.98 for classification of miRNA in the snail genome. In a 10-fold cross validation experiment using *Anolis carolinensis* as a hold-out species, the 0.98 confidence threshold represented a specificity of 99.8% and a sensitivity of 67.3% (Supplemental figure 67). We estimate a 1000:1 class imbalance ratio during the prediction of miRNA; at this level of class imbalance, a specificity of 99.8% produces a precision rate of approximately 0.25. Based on these results, we feel that the 0.98 confidence threshold provides a reasonable tradeoff between sensitivity and precision, retrieving the majority of true miRNA from a dataset while achieving a high precision rate in the face of the large class imbalance. Supplemental figure 67 demonstrates the average precision-recall tradeoff of 10 classifiers on *A. carolinensis* hold-out data.

To help validate predicted precursor miRNA above the 0.98 cut-off value, we used RNAseq data obtained from total RNA pools of mantle tissue to determine which sequences were actively transcribed in *B. glabrata*. We used Blast+ (v.2.2.29) to determine the presence of predicted precursor sequences within the RNAseq database. We used the following alignment criteria determine successful identification: (1) >95% alignment, (2) alignment of RNAseq data to the full length of the predicted precursor miRNA (+/- 2nt from either the 5' or 3' end), (3) the absence of gaps or mismatches, (4) no positive identification of the predicted precursor miRNA to the NCBI nucleotide database. For positive identification, all predicted miRNA met or exceeded each of these criteria.

Additional validation was carried out on predicted precursor miRNA by ensuring that each sequence was capable of producing mature miRNA. The prediction of mature miRNA was carried out using MiRdup (v.1.2), a newly developed prediction pipeline[236]. For these predictions, both mature miRNA and associated precursor miRNA sequences were downloaded from miRbase (http://www.mirbase.org/) (v.19). Training classifiers were generated specifically from sequences extracted from the Mollusca phylum of miRbase. For the purpose of training classifiers, negative sets of non-miRNAs were generated as follows. For each positive example (pair of miRNA and precursor miRNA), a negative example was generated by randomly relocating the miRNA along the same precursor miRNA sequence, preserving the miRNA's length, but excluding the exact position of the true miRNA or of any other known miRNA. Each Mollusca-specific training example was represented as a set of 100 features. To perform the ranking of attributes and classifier training and evaluation, we used Weka and its libraries. All classifiers were trained using 10-fold cross-

validation. Both the 3’ and 5’ mature miRNA sequences were extracted from each precursor miRNA predicted from *B. glabrata*.

**** Target prediction performed by Matheus de Souza Gomes research group* (Section 21)**

We identified 202 precursor, i.e. 95 known (identical to those predicted in Supplementary **Note** 21) and 107 novel, and associated mature miRNA from *B. glabrata* (Supplementary Data 32). No homologs to the identified novel *B. glabrata* precursor miRNAs were identified from the *Aplysia californica* or *Lottia gigantea* annotated miRNA, or present in available genomes.

To identify possible biological context of novel *B. glabrata* miRNA, mRNA targets were predicted from the 3' UTR of available *B. glabrata* transcripts (Supplementary Data 33). Interestingly, a significant proportion of the identified target genes of novel miRNA included multi-miRNA gene regulation of proteins involved in cellular processes such as secretory mucal proteins (Mucin-21-like)[237], matricellular proteins (Thrombospondin-3b-like)[238] and shell formation (Dentin sialophosphoprotein-like)[239] (Supplementary Fig. 66). These *B. glabrata* specific miRNAs may be involved in numerous cellular pathways and contribute that contribute to *B. glabrata* biology. The presented newly identified miRNA greatly enrich the repertoire of both mollusc and invertebrate miRNA, providing insights into mollusc miRNA function, as well as insights into evolution and biogenesis. Such species-specific miRNA may provide insight into estivation tolerance and may also present possible therapeutic targets of interest for *B. glabrata* population control[240]. Specifically we conclude the following:

1. Identification of conserved precursor and mature micoRNA in *Biomphalaria*. Combined efforts of the Storey and Matheus de Souza Gomes research groups identified a total of 95 conserved precursor and associated mature miRNA sequences from *Biomphalaria*.

2. Prediction of novel precursor miRNA in *Biomphalaria*.
In order to predict novel precursor miRNA with high accuracy, our precursor miRNA prediction approach generates data sets of real- and pseudo- miRNA from species that are closely related to *Biomphalaria*. This approach led to the successful identification of 107 novel precursor and associated mature miRNA sequences (additional to the 95 conserved sequences, for a total of 202 precursor miRNAs and associated mature miRNA from *Biomphalaria*).

3. Identification of candidate transcript targets of predicted novel miRNA. In collaboration with the research group of Matheus de Souza Gomes (Also see Section 21), we identified 82 unique transcriptional targets of the novel miRNA from *Biomphalaria*. Interestingly, the *Biomphalaria*-specific miRNAs target components of many cellular processes specific to snail biology. These include secretory mucosal proteins and those involved with shell formation. These novel miRNA-target interactions may provide insight into estivation tolerance and may also present possible *Biomphalaria*-specific therapeutic targets and provide insight for population control.

Supplementary Note 23. Potential circadian clock genes of *Biomphalaria glabrata*

The molecular mechanism of the *B. glabrata* circadian timing system is not yet understood, but previous studies of the gastropods *Aplysia californica* and *Bulla gouldiana* indicated that the circadian clock depends on daily transcription and protein synthesis[241-244]. The principle circadian clock in these species is present within a particular group of retinal neurons[246-246]. Circadian rhythms in the neural firing rate within eyes of *Bulla*, *Aplysia*, and opisthobranchs are well known[247]. The retinal circadian clock controls daily locomotor activity[248], but the location of the clock in *B. glabrata* has not been described. Daily rhythms in gene expression have been described in oysters and mussels[249-250], and homologs of several core clock gene are expressed in scallop eyes[251]. The timing cycle of the circadian clock in *Drosophila* and mammals depends on transcription-translation feedback loops that include a small set of core clock genes[252]. The *Bulla* retinal neurons that contain the circadian clock express a gene similar to the core circadian gene *per* of *Drosophila* [253] and a Per-like protein has been described in *Bulla* and *Aplysia* eyes[254]. To better understand circadian control of *B. glabrata* behavior and physiology we searched the genome for candidate orthologs of core clock genes from *Drosophila melanogaster*, mouse (*Mus musculus*), and *A. californica*.

The two interacting transcription-translation feedback loops that provide the near-24-hour timing of the circadian clock depend primarily on basic-helix-loop-helix (bHLH) proteins and retinoid-related orphan receptors (ROR) proteins[255]. The focus here was on basic-helix-loop-helix (bHLH) proteins and proteins containing two, sequential Per-Arnt-Sim (PAS) domains, a common feature of several core clock proteins. The predicted presence or absence of additional domains and protein motifs characteristic of core clock genes was also considered in the selection process. Selection was further restricted to candidates with nearby, upstream or downstream potential regulatory elements (E-box or E'-box), known binding sites for the subfamily of bHLH proteins that serve in the circadian clock, and also elements that bind ROR transcription factors. A strong basis for selection was provided by sequence similarities with previously characterized core clock genes of *Drosophila melanogaster*, mouse *(Mus musculus*), *A. californica,* and several arthropod species in the VectorBase collection of genomes. Comparisons with *Aplysia* were particularly useful because of its prominence as a model system for studies of circadian rhythms and because its genome is closely related to that of *B. glabrata*. The expressed mRNA of the clock *Period* gene of *B. gouldiana*, an opisthobranch related to *Aplysia*, has been sequenced and characterized and was also used in the analysis[252]. Several known circadian clock genes were queried in the *B. glabrata* database at VectorBase using BLAST, promoter analysis, and predicted protein features. These genes included *Per1*, *Per2 (period), Cry1, Cry2 (cryptochrome), Arntl (BMAL1), Clock, Nr1d1 (rev-erb α), Rora, Rorb (RAR-related orphan receptor)* of mouse; *per*, *cyc* (*cycle*, *Bmal1*), *clk (Clock), tim (timeless), vri (vrille), Eip75B* (*ecdysone-induced protein 75B*), *Hr51* (*Hormone receptor 51, UNF*) of *Drosophila;* the four *Aplysia* genes described as similar to known circadian clock genes (*period circadian protein-like* XP_005093378.1, *period circadian protein homolog 2-like* XP_005111020.1, and two *clock*-like genes, XP_005113503.1 and XP_005112430.1); and the *bPeriod* gene (AF353619.1) of *Bulla*.

The best candidate clock genes were identified as follows:

BGLB000136 (*BgPeriod-like1*) has two PAS domains, like Drosophila *per,* itd lacks a basic region for DNA binding, but unlike *per* lacks the period_C domain. It shows high similarity to the *Aplysia period circadian protein-like* gene ($E=5x10^{-54}$) and the *single-minded homolog 1* of mice ($E=7x10^{-154}$).

BGLB005764 (*BgCry-like1*) matches *Drosophila cyptochrome* ($E=10^{-17}$), is in the Cryptochrome family (by PANTHER), and contains the cryptochrome DNA-binding, FAD-binding, and photolyase

domains.
BGLB000055 (*BgCry-like2*) matches mouse *Cry1*, is in the Cryptochrome family, and contains the cryptochrome DNA-binding, FAD-binding, and photolyase domains.
BGLB012501 (*BgBMAL1-like*) matches mouse *arntl* (also known as *bmal1*, E=2x10^{-8}) and *Drosophila cycle* (*bmal1* ortholog, E=1x10^{-16}) and *Aplysia* period-like 2 peptide (E=2x10^{-13}) and *Aplysia* period-like peptide (E=4x10^{-19}). It has HLH domains, two PAS domains, and a non-canonical E-box overlapping a REV-ERB α binding site in the 2000 bps upstream.
BGLB000020 (*BgCLOCK-like*) matches mouse *clock* (E=3x10^{-24}) and *Aplysia period-like2 peptide* (E=4x10-7). Contains nuclear translocator, HLH, and PAS domains.
BGLB000113 (*BgRev-Erb-alpha like).* The predicted protein is a strong match (E=6x10^{-34}) with mouse Rev-erb α (*Nr1d1*) and contains two canonical E-boxes (CACGTG), one upstream and one in the first intron, which could provide control by a circadian bHLH protein, and one D-box in the first intron possibly providing additional clock control. It contains zinc-finger and nuclear receptor domains. The gene has been described as encoding one of the peroxisome proliferator-activated receptor (PPAR) proteins that are members of the same nuclear receptor family that includes Nr1d1. Mouse Nr1d1 shows about 84% identity with mouse PPAR δ.
BGLB011692 (*BgROR-alpha like*). The predicted protein is a strong match (E=9x10^{-49}) with mouse ROR-alpha and contains a canonical E-box upstream and one intronic D-box. Contains zinc-finger and nuclear receptor domains.

No strong BLAST matches (e-value<10^{-6}) were found for the *Per1* and *Per2* genes of mice as well as *Drosophila per, tim, vri,* and the mouse gene expressing the b-ZIP circadian clock protein DBP. Bg_Period-like, Bg_Cry-like1, and BgCLOCK-like were found among the total RNA transcripts over 500 bps in length expressed in the *B. glabrata* central nervous system, whereas BgCry-like2 and BgBMAL1-like were not found. The *Drosophila Eip75B* gene is a recently identified homolog of mammalian *Rev-erb α and Rev-erb β* [256,257]. It matches *B. glabrata* transcripts of BGLB004948 (E=6x10^{-9}), BGLB004947 (E=6x10^{-9}), and BGLB010069 *E75-like nuclear receptor* (E=8x10^{-7}). Another nuclear receptor gene serving in the *Drosophila* clock mechanism, *Hr51*, matched BGLB004019 (E=8x10^{-19}) and BGLB014038 *photoreceptor-specific nuclear receptor-like* (E=3x10^{-6}).

Candidate genes for several components of the eucoelomate circadian timing mechanism were identified, and distinct differences with known clock genes were observed. In particular, the *B. glabrata* period gene is very divergent from *per* genes of mammals and *Drosophila* but shows strong similarity to the opisthobranch mollusc *per* gene. Many zinc-finger nuclear receptor proteins provided a strong match with either of the ROR or REV-ERB genes, suggesting possible redundancy in linking of the two molecular timing loops or interaction with the clock by membrane-permeable hormones as in mammals. The presence of potential core clock genes in *B. glabrata* agrees with evidence of bHLH proteins being quite ancient with disparate species sharing a common ancestor over 600 million years ago[258].
Biomphalaria species show daily rhythms in cercarial release[259,260] that along with rhythms in locomotor activity[261] likely increase parasite contact with humans and infection during the daytime. Although an endogenous circadian clock in the snail has not been characterized, a freely expressed circadian rhythm in locomotion in constant experimental conditions devoid of external timing cues has been shown in a different pulmonate, *Limax maximus*[262]. Disruption of daily activity alters immune functions of *B. glabrata* and alters infection rate by miracidia[263]. Changing the expression levels of core clock genes alters the period or phase of circadian rhythms and can suppress oscillations. Functional studies need to be performed to verify that the expressed clock-related proteins act in providing timing of circadian rhythms in this snail.

Supplementary Note 24. Major neurohormones in *Biomphalaria glabrata*

Neuropeptides are the proteinaceous products of genes expressed by the nervous system in order to coordinate complicated physiological processes. They comprise a major class of signaling molecules, involved in the complete spectrum of animal functioning. *In silico* data mining of the *Biomphalaria glabrata* genome and tissue-specific transcriptomes identified 43 neuropeptide precursors, predicted to yield more than 250 mature signaling products.

To identify target sequences, the *Biomphalaria glabrata* gene coding region (CDS) databases were imported into the CLC Genomics Workbench (v6.0; Finlandsgade). Previously identified *Theba pisana* neuropeptides[264] and ovipostatin sequences were then used to query (TBLASTN and BLASTX) the databases. In parallel, open reading frames retrieved from the databases were translated and screened for the presence of recurrent KK; KR; RK; RR motifs. In many cases, *B. glabrata* gene CDS predictions could be supported from transcriptome database analyses. Multiple sequence alignments were created with MEGA software version 5.1[256]. Derived and actual amino acid sequences were aligned, guided by chain cleavage sites and conserved cysteines, where necessary intron donor/acceptor splice sites were identified using NetGene2[265]. Signal sequences and cleavage sites were identified by alignments with other mollusc sequences[266-268] and predicted through SignalP 4.0[269] and NeuroPred[270]. Sequence presentation and shading of multiple sequence alignments was performed using the LaTEX TEXshade package[271]. Phylogenetic trees were constructed using full length precursors or individual peptides with MEGA5.1 utilising the Maximum likelihood method[227]. Unrooted trees were generated with 1000 bootstrap trials and presented with a cut-off bootstrapping value of 50. Schematic diagrams of protein domain structures were prepared using Domain Graph (DOG, v2.0[272]).

The majority of neuropeptide sequences were identified in multiple tissues, though some were conspicuously specific to the CNS, namely: achatin, bursicon-α, fulicin-like peptide (LRNFVamide), gonadotropin-releasing hormone (GnRH), GPA5, GPB5, insulin-like peptides 2 and 3, neurokinin Y (NKY), and pleurin. Despite the other neuropeptides having a more heterogeneous presence throughout the organism, neuropeptide transcripts were most prominent within the CNS (24/43) and terminal genitalia databases (21/43). Precursor proteins or ‘prepropeptides’ are presented here accompanied by details of the tissues their transcripts have been identified in and their predicted bioactive cleavage products (Supplementary Datas 34-36). Additionally, the precursors of conopressin, GnRH, egg-laying hormone (ELH), whitnin and schistosomin-like peptides are presented with alignments and phylogenetic analysis, which demonstrates both gastropod idiosyncrasies and in stark contrast, domains that appear to be completely conserved (Supplementary Fig. 68).

This analysis has catalogued the secretory peptides employed by *B. glabrata* for higher-order neural control of its functioning and greatly facilitates the study of invertebrate neuropeptides with the hopes of stimulating advances in parasite-host ‘interactome’ research.

Supplementary Note 25. Ovipostatin-like male accessory gland protein in *Biomphalaria glabrata*.

In addition to queries for pheromones and neuropeptides that are known to be involved in regulation of reproduction in related snail species such as *Lymnaea stagnalis* and *Aplysia californica* (see Supplementary **Note** 24), we here focused on male accessory proteins (ACPs). Although relatively little is known about these substances in hermaphroditic snails, several Acps have recently been identified. Delivered with spermatozoa, ACPs modulate the reproductive physiology of hermaphroditic snails to augment fertilization success[273].

Blast searches with AcPs previously described from other gastropod species[273] were performed to identify related *B. glabrata* gene sequences. Using methods as described in Supplementay **Note** 24, a multiple sequence alignment (ClustalX) was generated for comparative phylogenetic analyses (Maximum likelihood) with MEGA5, using software estimated models for evolution. Schematic representation of ovipostatins in *B. glabrata* (Bg) compared to *L. stagnalis* (LyAcp10: accession number ADB12450) shows signal peptide, cysteine residues and intron locations. The scale bar indicates the number of amino acid substitutions per site.

Eight sequences matching one of ACPs from *L. stagnalis*, called Ovipostatin (LyAcp10), are also found in the genome of *B. glabrata*. Several of the *B. glabrata* ovipostatin sequences display conserved structural organization, primarily on the basis of their cysteine profiles and self-identity, see Supplementary Figure 69 and Supplementary Data 34 for the amino acid sequences of ovipostatins 1 to 8. Additional BLAST hits represented unreleated ependymin-related proteins and X-box binding proteins, and these did not not group together with the ovipostatin-like sequences in the gene tree (not shown).

The presence of an ovipostatin-like sequence is in agreement with observation that egg-laying is reduced under influence of ejaculate receipt in *B. glabrata*[274]. The fact that none of the other male accessory gland proteins identified in *L. stagnalis* are found in the *B. glabrata* genome could indicate that gastropod ACP sequences evolve rapidly under sexual selection and are species-specific. ACPs may thus provide a target for means to reduce reproduction of specific gastropods like *Biomphalaria* snails.

Supplementary Note 26. Endocrine mechanisms in *Biomphalaria glabrata*

The endocrine mechanisms governing reproduction and egg production in *B. glabrata* are poorly understood. The presence of both vertebrate steroids and arthropod ecdysteroids has been reported in molluscs, but their origin, dietary or de novo biosynthesis, has remained enigmatic. While steroids appear to be involved in molluscan reproduction (reviewed in Fernandes *et al*.[275]), data regarding the enzymatic pathways involved in steroid synthesis are fragmentary and there is some controversy as to which steroids are naturally produced in molluscs[77]. Identification of key components of reproductive physiology may provide targets for snail, and therefore parasite, control, since interrupting the intermediate host's life cycle through hormone manipulation, preventing egg development, would promote eradication. It would also further understanding of host-parasite interactions in the wider context, since many parasites curtail host reproduction via hormonal means. In particular, steroid hormones regulate essential processes during development and reproduction. In vertebrates, these are synthesized from cholesterol via steroidogenesis leading to estrogens, androgens, mineralocorticoids, and glucocorticoids from cholesterol. In arthropods, these are synthesized from dietary sterols leading to the polyhydroxylated molting hormones or ecdysteroids.

Sequences for each gene in the steroid synthesis pathway from vertebrates and arthropods were used to search the *B. glabrata* genome using TBLASTN. Identified sequences were reverse blasted using BLASTX against NCBI Genbank non-redundant database to confirm identity and or the presence of protein domains. The *B. glabrata* genes were then used to identify transcripts in the transcriptome (12 tissues, see Methods) to confirm/modify the predicted gene sequences.

We identified a CYP51 ortholog that encodes an enzyme required for sterol biosynthesis in all kingdoms of life. It is the most widely distributed P450 gene among animals with the notable exception of nematodes and arthropods. It catalyses the removal of the 14α methyl group from lanosterol (in animals) in the cholesterol biosynthetic pathway. The presence of the CYP51 gene indicates that snails can demethylate precursor sterols at the 14α position and therefore have the capability to synthesize sterols *de novo*.

Although we identified distant paralogs (about 30% amino acid identity) of the vertebrate steroidogenesis pathway genes 3β hydroxysteroid dehydrogenase, 17β hydroxysteroid dehydrogenase and 5α reductase, these are members of multigene families and it is difficult to ascertain their orthology in the absence of functional studies. The function in *B. glabrata* of the enzymes encoded is by these genes unknown. Based on identification of transcripts in the 12 tissue-specific libraries, the putative 5αR1 homolog is expressed in all except the heart/amebocyte-producing organ and salivary gland, while 5αR DET2 is expressed in fewer tissues but is expressed in the heart/amebocyte-producing organ (see Supplementary Data 37, Supplementary Figure 70).

Orthologs for the other genes of vertebrate steroid biosynthesis were not identified. These belong to the CYP superfamily of genes, which encode a group of structurally conserved P450 enzymes with diverse functions (see Supplementary **Note** 11). CYPs of specific interest for steroidogenesis are CYP17A1, CYP11A1, CYP11B1, CYP11B2 and CYP21A2 involved in different steps in the pathway and CYP19 (aromatase) which catalyses the conversion of androgens to estrogens. The absence of CYP11A1 suggests that molluscs lack the ability to cleave the side chain of cholesterol, the first committed step in steroid biosynthesis. It is therefore unclear whether molluscs do not synthesize such steroids or whether other, perhaps closely related genes (but not direct homologues) have evolved to make these or other mollusc specific steroids. The absence of CYP19, required for the formation of estrogens is particularly interesting, as *B. glabrata* has a homologue of the mammalian estrogen receptor[276]. The estrogen receptor of another mollusc,

Aplysia californica, can activate gene expression in the absence of ligand[277], a property that may therefore be common to molluscs, perhaps lophotrochozoa.

Similarly, we did not find orthologues of the P450 genes involved in arthropod ecdysteroid biosynthesis, even though molluscs have an ecdysteroid receptor (EcR) homolog[276,278]. Lack of ecdysteroid activation of the EcR of molluscs is therefore probable. We conclude that the endocrine biology of molluscs presents specific challenges, which will require further investigation.

Supplementary Note 27. Eukaryotic protein kinases (Epks) of *B. glabrata*

Most of eukaryotic protein phosphorylation processes which are key for the functioning and control of signaling pathways, are conducted by eukaryotic protein kinases (ePKs) that belong to a single superfamily and share conserved catalytic domains (PF00069 or PF07714). This superfamily is one of the largest in eukaryotes comprising from 1.5 to 2.5% of all proteins encoded by eukaryotic genomes[279]. We analyzed the *B. glabrata* proteome to identify the repertoire of ePKs encoded by this snail genome. Together with the protein phosphatases (Section 28), the annotation of *B. glabrata* ePKs presents a valuable framework for study of cellular regulation in this snail in growth, development and control of homeostasis, particularly in the face of environmental and pathogenic insult.

Eukaryotic protein kinases were identified as follows: InterProScan was used to assign domains to the predicted proteome of *B. glabrata*[28]. The output of InterProScan that integrated the following protein signature databases: BlastProDom, FPrintScan, HMM-PIR, HMM-Pfam, HMM-Smart, HMM-Tigr, ProfileScan, Pattern Scan, Superfamily, Gene3D and HMM-Panther was used to construct a relational database in Structured Query Language (SQL) used for querying and extracting information from the analysis using the graphical user interface DbVisualizer (http://dbvis.com/). Potential ePKs were identified based on the presence of Pfam conserved catalytic domains (PF00069 or PF07714) composed of 250-300 amino acids. Functional annotation using GO terms was also obtained using InterProScan[29]. In addition, the KEGG BRITE Database was used to classify *B. glabrata* ePKs by class (serine/threonine or tyrosine protein kinase), groups (AGC, CAMK, CK1, CMGC, STE, TK, RGC, TKL, and others) and families [280] in relation to the numbers of ePKs from humans and *S. mansoni* assigned for each of these groups.

Our analysis of the fasta sequences of the 14,141 proteins predicted to constitute the *B. glabrata* proteome, identified 240 potential ePKs due to the presence of diagnostic catalytic domains (Supplementary Data 3). The total number of ePKs corresponds to 1.7% of the *B. glabrata* predicted proteome. A total of 28 proteins have more than one match for the above-mentioned domains. According to KEGG BRITE, *B. glabrata* possesses 163 representatives of all except the RGC group (Supplementary Figure 71). Snail ePKs homologs were classified as AGC, CAMK, CK1, CMGC, STE, TK, and TKL group members. The largest group called ‘’Other'' consists of a mixed collection of kinases that could not be easily classified into the previous mentioned groups and theirs respective families. Furthermore, 77 proteins were not classified in any previously assigned group and were added to the "Unknown" kinases. Additional functional studies need to be performed to classify those proteins and further refine the classification, if possible to subfamily level, of all ePKs.

These findings provide a broad view of the *B. glabrata* ePKinome, improving eukaryotic kinase functional annotation. Our results can be used to guide new experiments designed to better understand the mechanisms regulating the activity of signaling pathways in *B. glabrata*, including how these may function in determining schistosome-susceptible and resistant phenotypes of *B. glabrata*.

Supplementary Note 28. Protein Phosphatases in the *Biomphalaria glabrata* genome.

The protein phosphatases regulate cellular processes by dephosphorylating specific substrate proteins thereby generally counteracting the effects of protein kinases. The complexity of protein kinase/phosphatase interactions is highlighted by the fact that they may work co-operatively to regulate the strength of a signal, and multiple kinase and phosphatase activities often contribute to the overall outcome of a particular signalling pathway. The phosphatases are now recognized as powerful (in some cases the dominant) controllers of many biological processes[281]. We aimed to identify candidate genes of phosphatases by *in silico* mining of the *B. glabrata* genome. Together with the kinases, the annotation of *B. glabrata* phosphatases presents a valuable framework for study of cellular regulation in this snail in growth, development and control of homeostasis, particularly in the face of pathogenic and environmental insult. To identify and classify *B. glabrata* protein phosphatases, the *B. glabrata* predicted proteome was queried through BLASTP search employing sequences of the conserved (Pfam) domains of representative phosphatases from all *Homo sapiens* phosphatase families [282,283]. Sequences of annotated phosphatases within NCBI from the molluscs *Aplysia californica* and *Crassostrea gigas* were also used in sequence similarity searches. BLAST hits were inspected and corrected or completed by eye/using RNAseq data as needed. Sequences were aligned (MUSCLE) to construct unrooted phylogenetic trees (MEGA5, neighbour-joining using software estimated evolutionary models) with 500 bootstrap replications.

Focusing particularly on the presence of the various types of phosphatase domains, sequence analysis identified approximately 59 putative protein phosphatases comprising ~35 protein Tyr phosphatases (PTPs) and ~24 protein Ser/Thr phosphatases (PSPs). The PSPs formed nine discrete clades and members of each of the three major PSP families were found: ~13 phosphoprotein phosphatases (PPPs), ~seven metal-dependent protein phosphatases (PPMs), and ~four aspartate-based phosphatases represented by FCP/SCP (Supplementary Figure 72). The PPPs appeared to be paraphyletic with the protein phosphatases 2A, 2B and 2C producing discrete distantly related clades indicating that they may have evolved independently (Supplementary Figure 72). Of the PTPs, we identified ~12 putative receptor PTPs and five non-receptor PTPs that together form the classical PTP family in *B. glabrata*. The remaining members broadly appear phylogenetically split from the classical PTPs (Supplementary Figure 73); these ‘dual-specificity phosphatases’ comprise ~18 proteins including ~three mitogen-activated protein kinase (MAPK) phosphatases (MKPs), ~seven atypical dual-specificity phosphatases (DUSPs), and ~four myotubularins (Supplementary Figure 73, 74), with the remainder being DUSP-related phosphatases of regenerating liver (PRLs), cell division cycle 14 proteins (DCD14s), and phosphatase and tensin homologues deleted on chromosome 10 (PTENs). The myotuberlarin-related phosphatases were the only group to form a descrete clade with the atypical DUSPs and the PTENs appearing paraphyletic (Supplementary Figure 74). Surprisingly the MKPs are shared across two discrete sister sub-clades which also contain a number of atypical DUSPs. This is indicative of the close evolutionary relationships between the DUSPs and also may indicate the multiple origins of functions that they perform. In the context of snail immunity and snail-schistosome interactions, the *B. glabrata* phosphatase orthologs (DUSPs 1, 7 and 10) that in humans dephosphorylate MAPK pathway components are likely to be important to the outcome of infection because MAPK pathways regulate anti-schistosome defence reactions in *B. glabrata* haemocytes[284-285] and are modulated in susceptible *B. glabrata* haemocytes by schistosome excretory-secretory products[286]. The findings on both phosphatases and the kinome provide important insight into the ‘yin’ and ‘yang’ of protein phosphorylation and cell signalling in *B. glabrata*. The analysis shows there to be considerable parallels to organisms such as humans and *Drosophila*, with similar evolutionary interrelationships within the PSPs and PTPs observed.

Supplementary Note 29. Evolutionary conservation of cardiac specification genes in *Biomphalaria glabrata*.

Higher animals, including molluscs, arthropods and chordates, are characterized at least in part by a muscular heart that functions to circulate blood or hemolymph through the body. The prevalence of heart-like organs in these diverse species has led to the proposal that the urbilaterian ancestor possessed a primitive heart, which was modified over evolutionary time into the diverse structures that we observe today. Evidence in support of this hypothesis comes from similarities in the genes for specification and differentiation of cardiac structures between insects (in particular Drosophila) and vertebrates[287,288]. Further support for this notion could come from the identification of a similar core of cardiac genes expressed in the molluscan heart.

The predominant cardiac transcription factor is the NK homeodomain protein Tinman/NKX2.5, which is essential for heart specification in Drosophila[289,290], and critical to normal heart development in vertebrates[291]. This gene has been a point of focus for molluscan studies of heart development. Elliott *et al.*[292] observed *LpNkx2.5* expression in the systemic heart (arterial system) of *Loligo pealii* at later stages of development, suggesting a conservation in function of NK domain factors in heart development. By contrast, Navet *et al.*[293] studied *Sepia officialis NK4*, and observed SoNK4 expression transiently and early in branchial hearts (venous system), but not in the developing systemic heart. Navet *et al.* concluded that, [293] while NK2 proteins may be important to cardiac development, they must function as part of a larger cadre of genes to mediate cardiac specification. We therefore sought to resolve this issue by identifying orthologs of core cardiac specification and differentiation genes in *Biomphalaria*, and determining if transcripts for these genes are enriched in the heart

We initially carried out RT-PCR using degenerate oligonucleotides designed to recognize the homeobox of *tin/Nkx2.5*[294], and we amplified from snail heart RNA a short sequence encoding the Tin homeodomain. Comparison of this sequence with predicted *Biomphalaria* transcripts identified *BGLB012592* as a putative *tin* ortholog. When SoNK4 and LpNkx2.5 were compared to the *B. glabrata* proteome, the same protein was identified. Moreover, BGLB012592 retains the tyrosine-rich domain and the NK2-specific domain characteristic of the Tin/Nkx family[292,295]. We therefore assign *BGLB012592* as the Biomphalaria ortholog of *tin/Nkx2.5*.

To identify other core cardiac genes, we used the *Drosophila* orthologs to identify *B. glabrata* genes through standard BLAST searches. We then used the identified *Biomphalaria* genes as queries in BLAST searches to determine if the closest *Drosophila* orthologs was in each case the *Drosophila* gene originally used in the search. Finally, we queried the *Biomphalaria* genes against human sequences to ensure that we had identified correct orthologs. A list of identified genes is presented in Supplementary Data 52. We conclude that, of the core cardiac regulatory factors and structural genes identified in *Drosophila* and mammals, essentially all of these genes have representatives in the *B. glabrata* genome.

Having identified the orthologous cardiac genes in *B. glabrata*, we next sought to determine if these genes showed enriched expression in cardiac tissues. Illumina short reads for the 12 tissues were used to assemble *de novo* the *B. glabrata* transcriptome, using Trinity software[125]. BLAST was used to identify assembled transcripts with sequence similarity to the cardiac genes. Normalized FPKM (Fragments per Kilobase of transcript per Million, Cufflinks) counts for these transcripts were aggregated on the gene level and used for creating heat maps, relative to the expression levels for each of the actin genes in the heart/APO (Figure 4). We note that of the 12 regulatory genes tested, all were expressed in the heart, and in many cases these genes showed enrichment in the heart when compared to expression levels in other tissues. Moreover, no other tissues analyzed showed

enrichment of all of the genes tested, indicating that expression of this core set of genes was a signature for heart tissue. The genes with the most striking heart-restriction in their expression were the orthologs of tin/NKX2.5, Hand/Hand1, eve/Evx1, pnr/GATA4, and svp/COUP-TFII, all of which have critical roles in cardiac development in insects and mammals.

We conclude from this analysis that a core set of regulatory factors required for cardiac specification in insects (Ecdysozoa) and mammals (Deuterostomia) is also present in *B. glabrata* (Lophotrochozoa) and that the expression of this complex of genes is enriched in the snail heart. These observations support the hypothesis that a primitive heart-like structure, that develops through the actions of a core heart toolkit, was present in the urbilaterian ancestor.

Supplementary Note 30. Evolution and expression analysis of actin genes in *Biomphalaria glabrata*.

Actin is an ubiquitous and highly-conserved globular protein that polymerizes into thin filaments that function in cell motility and, in animals, in muscle contraction. In higher animals, actin genes have duplicated and diversified to fulfill a number of tissue-specific roles, such as cytoplasmic versus sarcomeric actins; and the sarcomeric actins can be further diversified, to function in distinct muscle types[296].

While both insects and mammals have actin orthologs that are expressed in distinct muscle and cell types, the evolutionary origin of these genes is interesting. In particular, all of the six *Drosophila melanogaster* actin genes are closest in amino acid sequence similarity to mammalian cytoplasmic actins, rather than to any of the mammalian muscle actins[297]. This and subsequent data have led to the proposal that single actin gene precursors diverged independently in arthropod versus mammalian lineages, to give rise to the families of actin genes observed today[298].

Since molluscs (Lophotrochozoa) share a more recent common ancestor to mammals (Deuterostomia) than do arthropods (Ecdysozoa), it has been of interest to determine if the ancestral actin that diverged to give rise to the various mammalian genes was already diversifying during the origin of molluscs. Carlini *et al.*[299] studied cephalopod actin genes, and found that once again these genes are more closely related to one another than to any single mammalian gene. This work was supported by the observations of Sin *et al.*[300] studying actin gene sequence and expression in *Haliotis*. Thus, it has been proposed that actin diversification in arthropods, molluscs and vertebrates each occurred independently. However, it has not been determined if different molluscan lineages independently underwent actin gene divergence, and few studies have analyzed expression of mollusc actin genes in different tissues[300,301].

To investigate further these points, we identified the entire complement of actin genes encoded by the *B. glabrata* genome assenbly. *Biomphalaria glabrata* has ten actin genes, and whereas some of these genes are clustered in the genome (suggesting that they arose by tandem duplication), the ten genes as a whole occupy seven different genomic locations (Supplementary Data 39). Two genes, BGLB001013 and BGLB001649, show significant sequence divergence from the other actin genes, and might be considered to encode actin-related proteins; however, mollusc orthologs of canonical actin-related genes were also identified in our searches, and instead these two divergent genes appear to be novel to *Biomphalaria*.

To determine if the *Biomphalaria* actins showed tissue-specific expression patterns, we studied the accumulation of transcripts for each gene across the twelve tissues for which RNA-seq data were available. Illumina reads for the 12 tissues were used to assemble *de novo* the *B. glabrata* transcriptome, using the Trinity software[125]. BLAST was used to identify assembled transcripts with sequence similarity to the actin genes. Normalized FPKM (Fragments Per Kilobase of transcript per Million, Cufflinks) counts for these transcripts were aggregated on the gene level to create heat maps, relative to the expression levels for each of the actin genes in the heart/APO (Figure 4). We reliably detected expression of seven out of ten actin genes, the remaining three genes are either not expressed at high levels, or are expressed in tissues or at stages that are not well represented in the RNA-seq dataset. We found that four actin genes (*BGLB008298*, *BGLB008297*, *BGLB008299* and *BGLB003971* showed modest levels of expression across all tissues tested, suggesting that they might be cytoplasmic actin genes. Interestingly, three of these genes are clustered in the genome, and all four form a single clade within the *B. glabrata* actins (Figure 4). By contrast, three genes showed strongly differential expression (Figure 4). In particular *BGLB001649*, one of the divergent actin

genes, was highly expressed in the digestive gland while not detected in several other tissues. Our observations indicate that several *B. glabrata* actin genes show differential expression across tissues.

We next compared the amino acid sequences of the *B. glabrata* actins to actins from *Homo sapiens*. As was observed for other molluscan actin genes, all *B. glabrata* actins were most closely related to human cytoplasmic actin or to *D. melanogaster* cytoplasmic actin in species-specific comparisons (Supplementary Data 39). This might suggest that, similar to arthropods, the *B. glabrata* actin genes arose independently of their counterparts in the other non-mollusc lineages. Further comparison of *Biomphalaria* actin genes to actins of other molluscs (*Crassostrea gigas* and *Haliotis iris*, lophotrochozoans), an additional lophotrochozoan, the annelid *Hiruda medicinalis* (leech), *D. melanogaster* (arthropod, Ecdysozoa), *Amphimedon queenslandica* (sponge, Prebilateria) and the deuterostomes *Ciona intestinalis* (sea squirt) and *H. sapiens* were carried out, to determine if orthologous genes could be identified within the molluscan lineage. This was achieved through Maximum Likelihood analysis of encoding sequences (Phylogeny.fr;[61]). The comparison revealed that in each species the actin genes were most closely related to paralogs within their own genomes, rather than to other molluscan orthologs (Figure 4). One interpretation of these data is that actin genes diverged multiple independent times in the molluscan lineage, similar to prior conclusions for actin gene evolution in arthropods and chordates. An alternative explanation for these results is that, within a lineage, actin gene sequences are constrained to remain similar to one another, perhaps if the encoded proteins had overlapping functions. As a result, actins might appear monophyletic within a lineage due to selective pressures to keep the genes similar within each species. Some support for this alternate hypothesis comes from monophyly of actin coding sequences in diverse phyla tested (Figure 4). The following sequenes were used in this comparison (Species: actin gene, accession number): *Amphimedon queenslandica*: Aqu_LOC100638117, XM_003382837; Aqu_LOC100634046, XM_011405987; Aqu_LOC100633919, XM_003382889; *Hiruda medicinalis:* Hme_Act1, DQ333328; Hme_Act2, DQ333329; Hme_Act3, DQ333330; *Haliotis iris*: Hir_A1, AY921237; Hir_A1a, AY961954; Hir_A1b, AY961955; Hir_A1c, AY961956; Hir_A2, AY921238 Hir_A3, AY921239; *Crassostrea gigas*: Cgi_24, FJ669294; Cgi_39, FJ669293; Cgi_6, FJ669292; Cgi_12, FJ669291; Cgi_31, FJ669290; Cgi_15, FJ669289; Cgi_8, FJ669288; Cgi_10, FJ669287; Cgi_1, NM_001308859; Cgi_2, AB071191; *Drosophila melanogaster*: Dmel_5C, NM_001297986; Dmel_42A, NM_078901; Dmel_57B, NM_079076; Dmel_87E, NM_001300385; Dmel_79B, NM_001275271; Dmel_88F, NM_079643; *Ciona intestinalis:* Cin_LOC100185123, XM_002126220; Cin_LOC100176060, XM_002128912; Cin_LOC100176797, XM_002129572; Cin_LOC100183636, XM_002132016; Cin_LOC100184134, XM_002127468; Cin_LOC100178468, XM_002128635; Cin_LOC100180835, XM_002128571; Cin_LOC100183894, XM_002128888; Cin_LOC100185562, XM_002128926; *Homo sapiens:* Hsa_gamma2, BC094877; Hsa_gamma1, NM_001199954; Hsa_alpha1, BC012597; Hsa_beta, NM_001101; Hsa_alpha2, NM_001141945; Hsa_cardiac1, NM_005159.

In summary, we demonstrate differential tissue-specific expression of a subset of *Biomphalaria* actin genes, and provide a hypothesis for a novel mechanism of actin gene evolution.

Supplementary Note 31. Biomineralization genes in the Biomphalaria genome

By combining *in silico* techniques with the availability of both the genome and extensive transcriptome (Illumina RNAseq) data derived from different tissues, all from BB02 strain *B. glabrata*, we have identified a collection of putative shell-forming genes, some of which share orthology with biomineralisation genes in other molluscan species. These analyses indicate that some aspects of molluscan shell formation are deeply evolutionarily conserved.

The RNAseq data from 12 *B. glabrata* tissues were trimmed (fastq-mcf toolkit with the Illumina adapter file), combined and assembled using Trinity[125]. Each sample was aligned using Tophat2 to calculate differential expression levels across the tissues (cuffdiff with cummerbund[302]). Fold change was calculated as log2 (FPKM(x)/average FPKM (n1-12). All contigs in Supplementary Data 54 represent transcripts that were 2-fold more abundant in the mantle tissue relative to any other. Similarity searches were conducted against custom curated biomineralization databases using local installation of BLAST and processing output with in-house Perl scripts. Secreted proteins were identified using SignalP[31]. Ideogram figures were generated with Circos[303] and Circoletto[304].

A broad scale sequence similarity comparison was made of shell forming genes reported from several oysters (*Pinctada maxima*, *P. margaritifera* and *Crassostrea gigas*), the limpet (*Lottia gigantea*), the abalone (*Haliotis asinina*) and the grove snail (*Cepaea nemoralis*)[146, 305-308] against the bioinformatically predicted mantle-specific secretome of *B. glabrata*. Eighteen (10.2%) of the 177 *Biomphalaria* secreted shell-forming protein candidates shared similarity with those of other molluscs. The highest similarity was with carbonic anhydrase proteins and a novel protein with no identifiable domains, both in *C. nemoralis* (Figure 5). Of the 18 *B. glabrata* putative shell-forming proteins with similarities to other molluscan shell proteomes, nine (50%) returned significant hits when searched against SwissProt, including carbonic anhydrase, tyrosinase, and matrilin.

An analysis of the *Crassostrea gigas* (Pacific oyster) genome revealed that tyrosinase proteins are particularly abundant in both the shell and the mantle tissue[146]. We therefore searched for tyrosinase genes in all 12 RNASeq libraries of *B. glabrata* and identified a total of 31 unique isogroups (83 isotigs). Within the *B. glabrata* mantle RNASeq data there were seven isogroups (17 isotigs) containing tyrosinase-like domains. Six tyrosinase-like transcripts where abundantly expressed in the mantle tissue (>2 fold change) relative to all other tissues. These six transcripts mapped to three scaffolds: LG20i_random_Scaffold3077; LGUN_random_Scaffold2224; LGUN_random_Scaffold7808. The four transcripts mapping to LGUN_random_Scaffold2224, appear to be the products of alternative splicing derived from two genes (Supplementary Figure 84).

A fold change analysis of all *Biomphalaria* RNA-Seq data revealed 1,211 transcripts to be >2-fold up-regulated in the mantle tissue relative to the other 12 tissues. 1,066 of these up-regulated transcripts did not share similarity with protein sequences or domains in the NCBI nr database. Of the 145 transcripts that returned hits, 34 shared similarity with sequences known to be involved in the process of shell formation and biomineralization in molluscs (Supplementary Data 54).

Highly conserved calcification genes, such as carbonic anhydrase and tyrosinase, are well represented within the transcriptomes of the 12 *B. glabrata* tissues. Forty distinct carbonic anhydrase RNASeq isogroups (130 isotigs) were identified, and all 12 tissues contained at least one carbonic anhydrase transcript. In the shell forming mantle tissue two carbonic anhydrase isogroup sequences (8 isotigs) could be identified. Among these sequecnes, domain analysis using InterProScan identified an alpha carbonic anhydrase with a signal peptide suggesting it is an extra-cellular protein.

This initial genome-wide survey of shell forming genes in *B. glabrata* provides a foundation for future efforts that will aim to characterize the functions of these gene products, and highlights both the diversity and conservation of molluscan shell forming strategies.

Supplementary Note 32. Repeat and transposable element composition

Identification and classification of repetitive elements in the *B. glabrata* assembly were carried out with an annotation pipeline utilizing a *de novo* library of repeats combined with all known eukaryotic elements[309]. Briefly, outputs of RepeatModeler v. 1.0.8[310] with RepeatScout v. 1.0.4 (Price et al. 2005), RepeatMasker v.4.0.5[310] RM protein-based masking[311], and the REPCLASS workflow[312] were subjected to multiple iterations of manual editing for construction of a comprehensive "masking ready" expanded template library of consensus transposable element sequences. In addition to RepeatMasker annotation, a reference-free masking of the repetitive fraction of the snail genome sequence assembly was completed using the NCBI C++ tool kit application WindowMasker[313]. Tandem Repeat Finder version 4.0.4[314] was run to detect simple sequence repeats using default alignment parameters except for a reduced MaxPeriod value of 200 instead of the default 500, and with exclusion of HTML output. Results were summarized using RMPipeline, a set of generalized programs for analyzing RepeatMasker output written using Perl that are publicly available and free to use under the GPLv3 license at: https://github.com/hmsrc/RMPipeline.

The custom repeat library and the Repeatmasker output fileRepeat features were submitted to VectorBase (See vectorbase Biomphalaria-glabrata-BB02_REPEATS.lib, Biomphalaria-glabrata-BB02_REPEATFEATURES_BglaB1.gff3.gz).

We estimate that ~44.8% of the *B. glabrata* assembly is composed of interspersed repeats, largely derived from the activity of various transposable elements (TEs; Figure 1, Supplemental Fig. 85, Supplementary Data 55). The genomic fraction of interspersed repeats in *B. glabrata* is comparable to that of *Octopus bimaculoides* (43%)[315], slightly higher than that reported for the other mollusc genomes assembled to date, namely Pacific oyster, *Crassostrea gigas* (36%)[146], owl limpet, *Lottia gigantea* (21%)[91], and sea hare, *Aplysia californica* (30%)[316], and similar to that documented in *S. mansoni* (40%)[317].

Assembly-free masking methods using WindowMasker[309] revealed an almost identical estimate of genomic coverage by interspersed repeats (44%). Non-TE simple sequence repeats (i.e. microsatellites) comprise another 2.6% of the snail genome assembly and appear to be enriched for dinucleotide repeats.

As with other molluscs, the fraction of interspersed repeat families that remain unclassified as a specific type of TE, even after manual inspection, is non-negligible (17.6%). This can be attributed to the dearth of TE descriptions in molluscs and to the scarcity of comparative genome sequence data for a diversity of invertebrate clades. This 'unclassified' portion is likely to contain novel TE types as well as strongly degraded elements. Indeed, most of the unclassified repeat families are composed of highly diverged copies (Fig. 5). The most abundant classified repeats in the snail genome are retrotransposons of the long interspersed elements (LINEs) class (27% of the TE-derived DNA and 12.1 % of the genome assembly) followed by cut-and-paste DNA transposons (17.7% of the TE-derived DNA and 8% of the genome). Interestingly, LTR retroelements, which predominate in many other invertebrates, including *S. mansoni*[318], make up only a small percent of *B. glabrata* repeats and genome content (6% of TE-derived DNA and 1.7 % of the genome). A breakdown of each major TE classes into individual families (Supplementary Fig. 85, Supplementary Data 55) suggests a greater diversity of DNA transposons. Indeed, they include numerous low-copy number families relative to retrotransposons (LINEs and SINEs), which are represented by fewer, but generally more expanded families (Supplementary Fig. 86). Previous experimental studies have shown that the *Nimbus* family of LINEs is transcriptionally activated in response to *S. mansoni* infection, suggesting potential functional consequences of LINE transcription

in the intermediate host infection cycle[318]. Our analysis confirms that *Nimbus* elements are abundant in the snail's genome, together accounting for ~410 kb or ~0.05% of the genome assembly. The bulk of *Nimbus* elements are >5% diverged from their consensus ancestral sequence (Fig 87) and we could not identify any full-length copy with intact coding capacity. Nonetheless, given their sheer abundance, the impact of their transcriptional upregulation upon infection calls for future investigation. Overall, the dating of various TE families based on the divergence of individual copies to their consensus sequence indicates that very few TE families have expanded recently (Fig 1). Indeed, manual inspection of the most recent DNA transposon families, which belong to the *piggyBac* superfamily, revealed full-length copies riddled with mutations disrupting the transposase open reading frame, suggesting that they long ceased autonomously replicating. In light of the intermediate role of *B. glabrata* in the infection cycle of *S. mansoni*, we next examined the possibility that some of the TEs identified in the snail might have been shared with these parasites or some of their vertebrate hosts via horizontal transfer. To assess this, we ran BLAST searches with each of the consensus sequences in our *de novo* TE library for *B. glabrata* against the *S. mansoni* genome and against extensive repeat collections assembled previously for several vertebrate genomes[319]. While these searches revealed no clear instance of *B. glabrata* TE shared by *S. mansoni*, it uncovered a highly significant match (81% nucleotide identity across the entire element) between a ~2000-bp hAT DNA transposon of *B. glabrata* and the consensus sequence for the so-called *SPACE INVADERS (SPIN)* family[320]. Interestingly, it has been documented that *SPIN* transposons have horizontally infiltrated a wide range of animal species, including multiple tetrapods, the bloodsucking insect *Rhodnius prolixus*, and the pond snail *Lymnaea stagnalis,* another intermediate host for trematode parasites[320]. While the level of sequence similarity between the *B. glabrata SPIN*-like element and the vertebrate *SPINs* is not as extreme as those documented previously (up to 98%), it is still incompatible with a scenario of vertical acquisition of these elements from their common ancestor. Furthermore, the *SPIN*-like transposons residing in the *B. glabrata* genome (~1000 copies) are on average ~20% diverged from their consensus sequence, suggesting that the genomic invasion occurred relatively recently in the lineage of *B. glabrata* and independently than the invasion previously documented in the pond snail lineage[320]. In line with this previous evidence, we speculate that host-parasite interactions facilitated the horizontal introduction of the *SPIN*-like transposon in the lineage of *B. glabrata*.

Unclassified elements and relatively young elements are prominent in the snail genome although the current assembly does not provide evidence for full length active copies. New genomic data for economically important gastropod species and other promising biomedical models such as cephalopods will continue to help close gaps in our understanding of the impact of repeat and transposable elements on eukaryotic genome structure and function.

Supplementary Note 33. *Biomphalaria glabrata*, representation of the genome of the African species *Biomphalaria pfeifferi*.

The majority of the world's cases of *Schistosoma mansoni* occur in sub-Saharan Africa, transmitted primarily by the widespread African snail, *Biomphalaria pfeifferi*. To determine similarities between the Neotropical *B. glabrata* and African *B. pfeifferi*, we performed Illumina RNAseq of *B. pfeifferi* from a representative transmission focus in Western Kenya. Two uninfected *B. pfeifferi* snails were collected from Asao stream (0°19'04.8"S 35°00'24.7"E) in January 2013 and RNA was extracted using the Ambion PureLink RNA mini kit. Illumina HiSeq2000 RNAseq was performed at the National Center for Genome Resources (NCGR). The resulting ~58 million paired-end reads were *de novo* assembled using the Trinity platform[125].

A total of 85,776 isoforms greater than 200 nucleotides in length were generated with a GC content of 37.32% and average length of 1185 nucleotides. *Biomphalaria pfeifferi* isoforms were mapped to the overall *B. glabrata* transcriptome derived from RNAseq of 12 *B. glabrata* tissues using GMAP[321] and mapping was manually assessed by *B. pfeifferi* read coverage to annotated gene regions of the *B. glabrata* genome in WebApollo (http://webapollo.vectorbase.org/).

We identified *B. pfeifferi* homologs to 34 *B. glabrata* genes of interest described in this paper (Supplementary Data 42). These gene homologs have an average nucleotide identity of 97% and coverage of 90%. Homologs to an additional 162 genes were found by mapping *B. pfeifferi* transcripts to *B. glabrata* protein-coding entries from NCBI's Genbank using GMAP and BLASTN Supplementary Data 43). This second set of homologs has an average nucleotide identity and coverage of 96% and 93%, respectively. All of the above mapping procedures had default settings of 50% nucleotide identity and 50% coverage to be considered for further analysis. Genes were selected based on their known or predicted role in redox reactions, immune or defense response to macro- and microparasites, neurohormone and reproductive activity, and secretory-excretory functions. Fasta entries of *B. pfeifferi* sequences used in this study are included in Supplementary Data 42 and 43).

These results suggest that *B. glabrata* is a good source of sequence information that can guide studies of African *Biomphalaria* species. This makes sense because African *Biomphalaria* species were derived from a *B. glabrata*-like ancestor no more than 4.5 million years ago and possibly much more recently. Notable *B. glabrata* gene homologs absent in *B. pfeifferi* include the water-borne pheromones attractin and seductin, known to stimulate attraction and mating behavior in *Aplysia californica*[43]. This may provide insight into mating preference disparities between the two species, with *B. glabrata*'s affinity to outcross and *B. pfeifferi* as a preferential self-fertilizer[322].

Supplementary References

1. Rangel, N.M. Nota previa sobre o numero cromossomico de *Australorbis glabratus*. *Ciencia e cultura.* **3,** 284 (1951).
2. Fraga de Azevedo, J. & Goncalves, M.M. Ensaios sobre o estudo da numeracao cromosomica de algumas especies de moluscos de agua doce", *An. Inst. Med. Trpo.* **13**, 569-577 (1956).
3. Burch, J.B. Chromosome numbers of schistosome vector snails. *Zeitschrift fur Tropenmedizin und Parasitologie*, **11**, 442-449 (1960).
4. Narang, N. Chromosomal studies during spermatogenesis and the radiatin induced chromosomal aberrations in *Biomphalaria glabrata. Revista Brasileira de pesquisas edicas e biologicas,* **7**, 419-425 (1974).
5. Raghunathan, L. The karyotype of *Biomphalaria glabrata*, the snail vector of *Schistosoma mansoni*. *Malacologia*, **15**, 447-450 (1976).
6. Levan, A., Fredga, K. & Sandberg, A.A. Nomenclature or centromeric position on chromosomes. *Hereditas,* **52**, 201-220 (1964).
7. Hansen, E. L. in *Invertebrate Tissue Culture* (ed Karl Maramorosch) 75-99 (Elsevier, 1976).
8. Bayne, C.J., Owczarzak, A., Allen JR. Molluscan (*Biomphalaria*) cell line: Serology, Karyotype, Behavioral, and Enzyme Electrophoretic Characterization. *J. Invertebr. Pathol.,*. **32**, 35-39 (1978).
9. Odoemelam, EC., Raghavan, N., Miller, A., Bridger, J.M. & Knight, M. Revised karyotyping and gene mapping of the *Biomphalaria glabrata* embryonic (Bge) cell line. *Int. J. Parasitol.*. **39**, 675-681 (2009).
10. Rinaldi, G. *et al*. Cytometric analysis, genetic manipulation and antibiotic selection of the snail embryonic cell line Bge from *Biomphalaria glabrata*, the intermediate host of *Schistosoma mansoni*. *Int J Parasitol*. **45**, 527-535 (2015).
11. Goldman, M.A., Loverde, P.T., Chrisman, C.L. & Franklin, D.A. Chromosomal Evolution in Planorbid Snails of the Genera *Bulinus* and *Biomphalaria*. *Malacologia,* **25**, 427-446 (1984).
12. Kawano, T., Simoes, L.C.G., Foresti de Almeida Toledo, L. Nucleolar organizer regions in three species of the genus *Biomphalaria* (mollusca, gastropoda), *Brazillian Journal of Genetics* **4**, 695-707 (1987).
13. Raghavan, N., Knight, M. The snail (*Biomphalaria glabrata*) genome project. *Trends in Parasitology* **22**, 148-151 (2006).
14. Knight M, Ittiprasert W, Odoemelam EC, Adema CM, Miller A, Raghavan N, Bridger JM. Non-random organization of the *Biomphalaria glabrata* genome in interphase Bge cells and the spatial repositioning of activated genes in cells co-cultured with *Schistosoma mansoni*. *Int J Parasitol*. **41**, 61-70 (2011).
15. Arican-Goktas HD, Ittiprasert W, Bridger JM, Knight M. Differential spatio-epigenetic repositioning of activated genes in *Biomphalaria glabrata* resistant and susceptible snails infected with *Schistosoma mansoni*. *PLOS Negl. Trop. Dis.* **8**:e3013. (2014).
16. Knight M, Arican-Goktas H.D., Ittiprasert W., Miller A.N. and Bridger J.M. Schistosomes and Snails: a molecular encounter. *Front Genet*. 5:230. (2014).
17. Shaffer, L.G., Slovak, M.L. & Campbell, L.J. ISCN 2009 an international system for human cytogenetic nomenclature. *Human Genetics* **126** 603-604. (2009).
18. Odoemelam EC, Raghavan N, Ittiprasert W, Miller A, Bridger JM, Knight M.. FISH on chromosomes derived from the snail model organism *Biomphalaria glabrata*. *Methods Mol Biol*. **659**, 379-388 (2010).

19. Adema, C.M. *et al*. A bacterial artificial chromosome library for *Biomphalaria glabrata*, intermediate snail host of Schistosoma mansoni. *Mem Inst Oswaldo Cruz*, **101** Suppl 1, 167-177 (2006).
20. Francke U, Oliver N. Quantitative analysis of high-resolution trypsin-giemsa bands on human prometaphase chromosomes. *Hum Genet*. **45**, 137-165. (1978).
21. Baird, N. A. *et al.* Rapid SNP discovery and genetic mapping using sequenced RAD markers. *PLoS One* **3**, e3376, (2008).
22. Li, H. & Durbin, R. R. Fast and accurate short read alignment with Burrows-Wheeler transform. *Bioinformatics* **25**, 1754-1760, (2009).
23. Tennessen, J. A. *et al.* Genome-wide scan and test of candidate genes in the snail *Biomphalaria glabrata* reveal new locus influencing resistance to *Schistosoma mansoni*. *PLoS Negl. Trop. Dis.* **9**, e0004077, (2015).
24. Margarido, G. R. A., Souza, A. P. & Garcia, A. A. F. OneMap: Software for genetic mapping in outcrossing species. *Hereditas* **144**, 78-79, (2007).
25. Choi, J.-W. *et al.* Massively parallel sequencing of Chikso (Korean brindle cattle) to discover genome-wide SNPs and InDels. *Molecules and cells* **36**, 203-211, (2013).
26. Cingolani, P. *et al.* Using Drosophila melanogaster as a model for genotoxic chemical mutational studies with a new program, SnpSift. In *Toxicogenomics in non-mammalian species* (Vol. 3, p. 35). Frontiers E-books. (2012).
27. Moriya, Y. *et al*. KAAS: An Automatic Genome Annotation and Pathway Reconstruction Server. *Nucleic Acids Res.* **35**.Web Server issue W182–W185 (2007).
28. Hunter, S. *et al.* InterPro in 2011: New developments in the family and domain prediction database. *Nucleic Acids Res.* **40**, D306-D312, (2012).
29. Ashburner, M. *et al.* Gene ontology: Tool for the unification of biology. *Nat. Genet.* **25**, 25-29, (2000).
30. Reich, M. *et al*. GenePattern 2.0. *Nature genetics* 38:500-501 (2006).
31. Petersen, T.N., Brunak, S., von Heijne, G. & Nielsen, H. SignalP 4.0: discriminating signal peptides from transmembrane regions. *Nature Methods*, **8**:785-786, (2011)
32. Bendtsen, J.D., Nielsen, H., von Heijne, G., & Brunak, S. Improved prediction of signal peptides: SignalP 3.0. *J. Mol. Biol.* **340**, 783-795. (2004).
33. Emanuelsson, O., Nielsen, H., Brunak, S., von Heijne, G. Predicting subcellular localization of proteins based on their N-terminal amino acid sequence. *J Mol Biol.* **300**:1005-1016 (2000).
34. Krogh, A., Larsson, B., von Heijne, G. & Sonnhammer, E.L. Predicting transmembrane protein topology with a hidden Markov model: application to complete genomes. *J Mol Biol.* **305**:567-580 (2001).
35. Finn, R. D. *et al*. Pfam: the protein families database. *Nucleic Acids Res.* **42** (Database issue), D222–D230 (2014).
36. Adema, C.M. *et al*. A family of fibrinogen-related proteins that precipitates parasite-derived molecules is produced by an invertebrate after infection *Proc Natl Acad Sci USA.* **94**:8691–8696 (1997**).**
37. Rapado, L.N. *et al*. Ovicidal effect of piperaceae species on *Biomphalaria glabrata*, *Schistosoma mansoni* host. *Revista do Instituto de Medicina Tropical de São Paulo* **55**:421-424 (2013).
38. Tennessen J. A. *et al.* Hyperdiverse gene cluster in snail host conveys resistance to human schistosome parasites. *PLoS Genet.***11**: e1005067. (2015).
39. Mortazavi, A. *et al*. Mapping and quantifying mammalian transcriptomes by RNA-Seq. Nat. Methods. **5,** 621-628 (2008)

40. Young, N.D. *et al*. The Medicago genome provides insight into the evolution of rhizobial symbioses. Nature **480**, 520–524. (2011).
41. Landin, M.A. *et al.* Gene expression profiling during murine tooth development. *Front. Genet.***3**,139 (2012).
42. Hiller, K., Grote, A., Scheer, M., Munch, R., and Jahn, D. PrediSi: prediction of signal peptides and their cleavage positions. *Nucleic Acids Res.* **32**, W375-379. (2004).
43. Cummins, S. F., Nichols, A. E., Schein, C. H. & Nagle, G. T. Newly identified water-borne protein pheromones interact with attractin to stimulate mate attraction in *Aplysia*. *Peptides* **27**, 597-606, (2006).
44. Cummins, S. F., *et al.* Candidate chemoreceptor subfamilies differentially expressed in the chemosensory organs of the mollusc *Aplysia*. *BMC Biology* **7**, 1741-7007, (2009)
45. Cummins, S.F., Tollenaere, A., Degnan, B.M. & Croll, R.P. Molecular analysis of two FMRFamide-encoding transcripts expressed during the development of the tropical abalone *Haliotis asinina*. *J. Comp. Neurol.* **519**, 2043-2059. (2011).
46. McGladdery, S. E. in *Fish Diseases and Disorders* (eds P.T.K. Woo & D.W. Bruno) 748-854 (CAB International, 2011).
47. Pawar, K. D. *et al.* Bacterial diversity in different regions of gastrointestinal tract of Giant African snail (*Achatina fulica*). *Microbiology Open* **1**, 415-426, (2012).
48. Tamura, K. *et al.* MEGA5: Molecular evolutionary genetics analysis using maximum likelihood, evolutionary distance, and maximum parsimony methods. *Mol. Biol. Evol.* **28**, 2731-2739, (2011).
49. Delcher, A. L., Bratke, K. A., Powers, E. C. & Salzberg, S. L. Identifying bacterial genes and endosymbiont DNA with Glimmer. *Bioinformatics* **23**, 673-679, (2007).
50. Götz, S. *et al.* High-throughput functional annotation and data mining with the Blast2GO suite. *Nucleic Acids Res.* **36**, 3420-3435, (2008).
51. Haas *et al.*,. De novo transcript sequence reconstruction from RNA-seq using the Trinity platform for reference generation and analysis. *Nature Protocols* **8**,1494–1512 doi:10.1038/nprot.2013.084 (2013)
52. Altschul, S.F., Madden, T.L., Schäffer, A.A., Zhang, J., Zhang, Z., Miller, W. & Lipman, D.J. (1997) "Gapped BLAST and PSI-BLAST: a new generation of protein database search programs." *Nucleic Acids Res*. **25**:3389-3402.
53. Goic et al. RNA-mediated interference and reverse transcription control the persistence of RNA viruses in the insect model *Drosophila*. *Nat. Immunol*. **14**, 396-403 (2013).
54. Kampinga, H. H. *et al.* Guidelines for the nomenclature of the human heat shock proteins. *Cell Stress Chaperones* **14**, 105-111, (2009).
55. Kent W. J., *et al.* The human genome browser at UCSC. Genome Research. 12(6):996-1006 (2002a).
56. Kent, W.J. BLAT - the BLAST-like alignment tool. *Genome Res.* **12**, 656-664, (2002b).
57. Raghavan, N. *et al.* Nimbus (BgI): An active non-LTR retrotransposon of the *Schistosoma mansoni* snail host *Biomphalaria glabrata*. *Int. J. Parasitol.* **37**, 1307-1318, (2007).
58. Marchler-Bauer, A. *et al.* CDD: Conserved domains and protein three-dimensional structure. *Nucleic Acids Res.* **41**, D348-D352, (2013).
59. Yoshino, T. P., Wu, X. J. & Liu, H. D. Transfection and heat-inducible expression of molluscan promoter-luciferase reporter gene constructs in the *Biomphalaria glabrata* embryonic snail cell line. *The American Journal of Tropical Medicine and Hygiene* **59**, 414-420, (1998).

60. Hennessy, F., Nicoll, W. S., Zimmermann, R., Cheetham, M. E. & Blatch, G. L. Not all J domains are created equal: Implications for the specificity of Hsp40-Hsp70 interactions. *Protein Sci.* **14**, 1697-1709, (2005).
61. Dereeper, A. *et al.* Phylogeny.fr: Robust phylogenetic analysis for the non-specialist. *Nucleic Acids Res.* **36**, W465-W469, (2008).
62. Fedoroff, N. V. Transposable elements as a molecular evolutionary force. *Ann. N. Y. Acad. Sci.* **870**, 251-264, (1999).
63. Ittiprasert, W. *et al. Schistosoma mansoni* infection of juvenile *Biomphalaria glabrata* induces a differential stress response between resistant and susceptible snails. *Exp. Parasitol.* **123**, 203-211, (2009).
64. Renuse, S., Chaerkady, R. & Pandey, A. Proteogenomics. *Proteomics* **11**, 620-630, (2011)
65. Yoshino, T. P., Bickham, U. & Bayne, C. J. Molluscan cells in culture: Primary cell cultures and cell lines 1. *Can. J. Zool.* **91**, 391-404, (2013).
66. Yoshino, T. P., Coustau, C., Modat, S. & Castillo, M. G. The *Biomphalaria glabrata* (Bge) embryonic cell line: Establishment of an in vitro cellular model for the study of snail host-parasite interactions. *Malacologia* **41**, 331-343, (1999).
67. Humphries, J. E. & Yoshino, T. P. *Schistosoma mansoni* excretory–secretory products stimulate a p38 signalling pathway in *Biomphalaria glabrata* embryonic cells. *Int. J. Parasitol.* **36**, 37-46, (2006).
68. Jiang, Y., Loker, E. S. & Zhang, S.-M. In vivo and in vitro knockdown of *FREP2* gene expression in the snail *Biomphalaria glabrata* using RNA interference. *Dev. Comp. Immunol.* **30**, 855-866, (2006).
69. Yoshino, T. P. & Laursen, J. R. Production of *Schistosoma mansoni* daughter sporocysts from mother sporocysts maintained in synxenic culture with *Biomphalaria glabrata* embryonic (Bge) cells. *The Journal of Parasitology* **81**, 714-722, (1995).
70. Peterson, N. A., Anderson, T. K., Wu, X.-J. & Yoshino, T. P. In silico analysis of the fucosylation-associated genome of the human blood fluke *Schistosoma mansoni*: Cloning and characterization of the enzymes involved in GDP-L-fucose synthesis and Golgi import. *Parasit Vectors* **6**, 201-201, (2013).
71. Wu, X.-J. *et al.* Proteomic analysis of *Schistosoma mansoni* proteins released during in vitro miracidium-to-sporocyst transformation. *Mol. Biochem. Parasitol.* **164**, 32-44, (2009).
72. Dheilly, N. M. *et al.* A family of variable immunoglobulin and lectin domain containing molecules in the snail *Biomphalaria glabrata*. *Dev. Comp. Immunol.* **48**, 234-243, (2015).
73. Werck-Reichhart, D. & Feyereisen, R. Cytochromes P450: a success story. *Genome Biology* **1**, 30031-30039, (2000).
74. Feyereisen, R. Arthropod CYPomes illustrate the tempo and mode in P450 evolution. *Biochim. Biophys. Acta* **1814**, 19-28, (2011).
75. Sezutsu, H., Le Goff, G. & Feyereisen, R. Origins of P450 diversity. *Phil. Trans. R. Soc. B* **368**, 20120428, (2013).
76. Nelson, D. R., Goldstone, J. V. & Stegeman, J. J. The cytochrome P450 genesis locus: The origin and evolution of animal cytochrome P450s. *Phil. Trans. R. Soc. B* **368**, 20120474-20120474, (2013).
77. Scott, A. P. Do molluscs use vertebrate sex steroids as reproductive hormones? Part I: Critical appraisal of the evidence for the presence, biosynthesis and uptake of steroids. *Steroids* **77**, 1450-1468, (2012).

78. Teunissen, Y., Geraerts, W.P.M., van Heerikhuizen, H., Planta, R.J. & Joosse J. Molecular cloning of a cDNA encoding a member of a novel cytochrome P450 family in the mollusc *Lymnaea stagnalis*. Journal of Biochemistry **112**, 249-252 (1992).
79. Buckley, K. M. & Rast, J. P.. Characterizing immune receptors from new genome sequences. *Methods Mol Biol.* **748**:273-98 (2011)
80. Eddy, S. R. Accelerated profile HMM Searches. *PLoS Comput. Biol.* **7**, e1002195, (2011).
81. Gasteiger, E. *et al.* ExPASy: The proteomics server for in-depth protein knowledge and analysis. *Nucleic Acids Res.* **31**, 3784-3788, (2003).
82. Lemaitre, B., Nicolas, E., Michaut, L., Reichhart, J.-M. & Hoffmann, J. A. The dorsoventral regulatory gene cassette *spätzle*/*Toll*/*cactus* controls the potent antifungal response in *Drosophila* adults. *Cell* **86**, 973-983, (1996).
83. Poltorak, A. *et al.* Defective LPS signaling in C3H/HeJ and C57BL/10ScCr mice: Mutations in *TLR4* gene. *Science* **282**, 2085-2088, (1998).
84. Medzhitov, R., Preston-Hurlburt, P. & Janeway, C. A. A human homologue of the *Drosophila* Toll protein signals activation of adaptive immunity. *Nature* **388**, 394-397, (1997)
85. Messier-Solek, C., Buckley, K. M. & Rast, J. P. Highly diversified innate receptor systems and new forms of animal immunity. *Semin. Immunol.* **22**, 39-47, (2010).
86. Buckley, K. M. & Rast, J. P. Diversity of animal immune receptors and the origins of recognition complexity in the deuterostomes. *Dev. Comp. Immunol.* **49**, 179-189, (2015).
87. Buckley, K. M. & Rast, J. P. Dynamic evolution of toll-like receptor multigene families in echinoderms. *Front. Immunol*. **3**, 1–16 (2012).
88. Hibino, T. *et al.* The immune gene repertoire encoded in the purple sea urchin genome. *Dev. Biol.* **300**, 349-365, (2006).
89. Holland, L. Z. *et al.* The amphioxus genome illuminates vertebrate origins and cephalochordate biology. *Genome Res.* **18**, 1100-1111, (2008).
90. Davidson, C. R., Best, N. M., Francis, J. W., Cooper, E. L. & Wood, T. C. Toll-like receptor genes (TLRs) from *Capitella capitata* and *Helobdella robusta* (Annelida). *Dev. Comp. Immunol.* **32**, 608-612, (2008).
91. Simakov, O. *et al.* Insights into bilaterian evolution from three spiralian genomes. *Nature* **493**, 526-531, (2013).
92. Rock, F. L., Hardiman, G., Timans, J. C., Kastelein, R. A. & Bazan, J. F. A family of human receptors structurally related to *Drosophila* Toll. *Proc. Natl. Acad. Sci. U.S.A.* **95**, 588-593, (1998).
93. Leulier, F. & Lemaitre, B. Toll-like receptors - taking an evolutionary approach. *Nature Reviews Genetics* **9**, 165-178, (2008).
94. Wlasiuk, G. & Nachman, M. W. Adaptation and constraint at Toll-like receptors in primates. *Mol. Biol. Evol.* **27**, 2172-2186, (2010).
95. Cheng, X., Zhang, X., Pflugrath, J. W. & Studier, F. W. The structure of bacteriophage T7 lysozyme, a zinc amidase and an inhibitor of T7 RNA polymerase. *Proc. Natl. Acad. Sci. U.S.A.* **91**, 4034-4038, (1994).
96. Lu, X. *et al.* Peptidoglycan recognition proteins are a new class of human bactericidal proteins. *J. Biol. Chem.* **281**, 5895-5907, (2006).
97. Royet, J., Gupta, D. & Dziarski, R. Peptidoglycan recognition proteins: Modulators of the microbiome and inflammation. *Nat. Rev. Immunol.* **11**, 837-851, (2011).
98. Paredes, J. C., Welchman, D. P., Poidevin, M. & Lemaitre, B. Negative regulation by amidase PGRPs shapes the *Drosophila* antibacterial response and protects the fly from innocuous infection. *Immunity* **35**, 770-779, (2011).

99. Troll, J. V. *et al.* Taming the symbiont for coexistence: A host PGRP neutralizes a bacterial symbiont toxin. *Environ. Microbiol.* **12**, 2190-2203, (2010).
100. Zhang, S.-M., Zeng, Y. & Loker, E. S. Characterization of immune genes from the schistosome host snail *Biomphalaria glabrata* that encode peptidoglycan recognition proteins and gram-negative bacteria binding protein. *Immunogenetics* **59**, 883-898, (2007).
101. Littman, D. R. & Rudensky, A. Y. Th17 and regulatory T cells in mediating and restraining inflammation. *Cell* **140**, 845-858, (2010).
102. Ramirez-Carrozzi, V. *et al.* IL-17C regulates the innate immune function of epithelial cells in an autocrine manner. *Nat. Immunol.* **12**, 1159-1166, (2011).
103. Song, X. *et al.* IL-17RE is the functional receptor for IL-17C and mediates mucosal immunity to infection with intestinal pathogens. *Nat. Immunol.* **12**, 1151-1158, (2011).
104. Wu, S.-Z., Huang, X.-D., Li, Q. & He, M.-X. Interleukin-17 in pearl oyster (*Pinctada fucata*): Molecular cloning and functional characterization. *Fish and Shellfish Immunology* **34**, 1050-1056, (2013).
105. Roberts, S., Gueguen, Y., de Lorgeril, J. & Goetz, F. Rapid accumulation of an interleukin 17 homolog transcript in *Crassostrea gigas* hemocytes following bacterial exposure. *Dev. Comp. Immunol.* **32**, 1099-1104, (2008).
106. Baeza Garcia, A. *et al.* Involvement of the cytokine MIF in the snail host immune response to the parasite *Schistosoma mansoni*. *PLoS Pathog.* **6**, e1001115, (2010).
107. Zhang, L., Li, L. & Zhang, G. A *Crassostrea gigas* Toll-like receptor and comparative analysis of TLR pathway in invertebrates. *Fish and Shellfish Immunology* **30**, 653-660, (2011).
108. Elvitigala, D. A. S., Premachandra, H. K. A., Whang, I., Nam, B.-H. & Lee, J. Molecular insights of the first gastropod TLR counterpart from disk abalone (*Haliotis discus discus*), revealing its transcriptional modulation under pathogenic stress. *Fish and Shellfish Immunology* **35**, 334-342, (2013).
109. Toubiana, M. *et al.* Toll-like receptors and MyD88 adaptors in *Mytilus*: Complete cds and gene expression levels. *Dev. Comp. Immunol.* **40**, 158-166, (2013).
110. Cornet, V., Henry, J., Corre, E., Le Corguillé, G. & Zatylny-Gaudin, C. The Toll/NF-κB pathway in cuttlefish symbiotic accessory nidamental gland. *Dev. Comp. Immunol.* **53**, 42-46, (2015).
111. Pila, E.A., Tarrabain, M., Kabore, A.L. & Hanington PC. A novel Toll-Like receptor (TLR) influences compatibility between the gastropod *Biomphalaria glabrata*, and the digenean trematode *Schistosoma mansoni*. PLoS Pathog. **12**(3):e10055132016 (2016).
112. Kawai, T. & Akira, S. TLR signaling. *Cell Death Differ.* **13**, 816-825, (2006).
113. Aksoy, E. *et al.* Double-stranded RNAs from the helminth parasite Schistosoma activate *TLR3* in dendritic cells. *J. Biol. Chem.* **280**, 277-283, (2005).
114. Zhang, S.-M., Adema, C. M., Kepler, T. B. & Loker, E. S. Diversification of Ig superfamily genes in an invertebrate. *Science* **305**, 251-254, (2004).
115. Hanington, P. C. *et al.* Role for a somatically diversified lectin in resistance of an invertebrate to parasite infection. *Proc. Natl. Acad. Sci. U.S.A.* **107**, 21087-21092, (2010a).
116. Hanington, P. C., Forys, M. A. & Loker, E. S. A somatically diversified defense factor, FREP3, is a determinant of snail resistance to schistosome infection. *PLoS Negl. Trop. Dis.* **6**, e1591, (2012).
117. Mitta, G., Adema, C. M., Gourbal, B., Loker, E. S. & Theron, A. Compatibility polymorphism in snail/schistosome interactions: From field to theory to molecular mechanisms. *Dev. Comp. Immunol.* **37**, 1-8, (2012).

118. Tamura, K., Stecher, G., Peterson, D., Filipski, A. & Kumar, S. MEGA6: Molecular Evolutionary Genetics Analysis version 6.0. *Mol. Biol. Evol.* **30**, 2725-2729, (2013).
119. Zhang, S.-M., Léonard, P. M., Adema, C. M. & Loker, E. S. Parasite-responsive IgSF members in the snail *Biomphalaria glabrata*: Characterization of novel genes with tandemly arranged IgSF domains and a fibrinogen domain. *Immunogenetics* **53**, 992-992, (2001).
120. Gong, H. *et al.* A novel PAN/apple domain-containing protein from *Toxoplasma gondii*: Characterization and receptor identification. *PLoS One* **7**, e30169, (2012).
121. Léonard, P. M., Adema, C. M., Zhang, S.-M. & Loker, E. S. Structure of two FREP genes that combine IgSF and fibrinogen domains, with comments on diversity of the FREP gene family in the snail *Biomphalaria glabrata*. *Gene* **269**, 155-165, (2001).
122. Gorbushin, A. M., Panchin, Y. V. & Iakovleva, N. V. In search of the origin of FREPs: Characterization of *Aplysia californica* fibrinogen-related proteins. *Dev. Comp. Immunol.* **34**, 465-473, (2010).
123. Morley, B. J. & Walport, M. J. *The Complement FactsBook*. (Academic Press, 1999).
124. Mishra, J., Sahoo, P. K., Mohanty, B. R. & Das, A. Sequence information, ontogeny and tissue-specific expression of complement component C3 in Indian major carp, *Labeo rohita* (Hamilton). *Indian J. Exp. Biol.* **47**, 672-678, (2009).
125. Grabherr, M. G. *et al.* Full-length transcriptome assembly from RNA-Seq data without a reference genome. *Nat. Biotechnol.* **29**, 644-652, (2011).
126. Matsushita, M. *et al.* Origin of the classical complement pathway: Lamprey orthologue of mammalian C1q acts as a lectin. *Proc. Natl. Acad. Sci. U.S.A.* **204**, 10127-10131, (2004)
127. Blandin, S. & Levashina, E. A. Thioester-containing proteins and insect immunity. *Mol. Immunol.* **40**, 903-908, (2004).
128. Beinrohr, L. *et al.* Serpins and the complement system. *Methods Enzymol.* **499**, 55-75, (2011).
129. Emsley, J. *et al.* Structure of pentameric human serum amyloid P component. *Nature* **367**, 338-345, (1994).
130. Gál, P., Dobó, J., Beinrohr, L., Pál, G. & Závodszky, P. Inhibition of the serine proteases of the complement system. *Adv. Exp. Med. Biol.* **735**, 23-40, (2013).
131. Rutkowski, M. J. *et al.* Complement and the central nervous system: Emerging roles in development, protection and regeneration. *Immunol. Cell Biol.* **88**, 781-786, (2010).
132. Falschlehner, C. & Boutros, M. Innate immunity: Regulation of caspases by IAP-dependent ubiquitylation. *The EMBO Journal* **31**, 2750-2752, (2012).
133. Gervais, O., Renault, T. & Arzul, I. Induction of apoptosis by UV in the flat oyster, *Ostrea edulis*. *Fish and Shellfish Immunology* **46**, 232-242, (2015).
134. Xian, J.-A. *et al.* Haemocyte apoptosis of the tiger shrimp *Penaeus monodon* exposed to cadmium. *Bull. Environ. Contam. Toxicol.* **92**, 525-528, (2014).
135. Vandergaast, R., Mitchell, J. K., Byers, N. M. & Friesen, P. D. Insect inhibitor-of-apoptosis (IAP) proteins are negatively regulated by signal-induced N-terminal degrons absent within viral IAP proteins. *J. Virol.* **89**, 4481-4493, (2015).
136. Xu, D., Liu, W., Alvarez, A. & Huang, T. Cellular immune responses against viral pathogens in shrimp. *Dev. Comp. Immunol.* **47**, 287-297, (1999).
137. Du, Z.-Q., Lan, J.-F., Weng, Y.-D., Zhao, X.-F. & Wang, J.-X. *BAX* inhibitor-1 silencing suppresses white spot syndrome virus replication in red swamp crayfish, *Procambarus clarkii*. *Fish and Shellfish Immunology* **35**, 46-53, (2013).
138. Liu, B. *et al.* P53-mediated rapid induction of apoptosis conveys resistance to viral infection in *Drosophila melanogaster*. *PLoS Pathog.* **9**, e1003137, (2013).

139. Mizerska-Dudka, M. & Andrejko, M. *Galleria mellonella* hemocytes destruction after infection with *Pseudomonas aeruginosa*. *J. Basic Microbiol.* **54**, 232-246, (2014).
140. Fan, J., Xie, Y., Xue, J., Zhang, Y. & Yang, Q. Cellular apoptosis of hemocytes from *Dendrolimus tabulaeformis* Tsai et Liu larvae induced with the secondary metabolites of *Beauveria brongniartii* (Sacc.) Petch. *PLoS One* **8**, e71600, (2013).
141. Ramphul, U. N., Garver, L. S., Molina-Cruz, A., Canepa, G. E. & Barillas-Mury, C. *Plasmodium falciparum* evades mosquito immunity by disrupting JNK-mediated apoptosis of invaded midgut cells. *Proc. Natl. Acad. Sci. U.S.A.* **112**, 1273-1280, (2015).
142. Hay, B. A. & Guo, M. Caspase-dependent cell death in *Drosophila*. *Annu. Rev. Cell Dev. Biol.* **22**, 623-650, (2006)
143. Lettre, G. & Hengartner, M. O. Developmental apoptosis in *C. elegans*: A complex CEDnario. *Nature Reviews Molecular Cell Biology* **7**, 97-108, (2006).
144. Xu, D. *et al.* Genetic control of programmed cell death (apoptosis) in *Drosophila*. *Fly* **3**, 78-90, (2009).
145. Domingos, P. M. & Steller, H. Pathways regulating apoptosis during patterning and development. *Curr. Opin. Genet. Dev.* **17**, 294-299, (2007).
146. Zhang, G. *et al.* The oyster genome reveals stress adaptation and complexity of shell formation. *Nature* **490**, 49-54, (2012).
147. Nowak, T. S., Woodards, A. C., Jung, Y., Adema, C. M. & Loker, E. S. Identification of transcripts generated during the response of resistant *Biomphalaria glabrata* to *Schistosoma mansoni* infection using suppression subtractive hybridization. *The Journal of Parasitology* **90**, 1034-1040, (2004).
148. Lockyer, A. E. *et al. Biomphalaria glabrata* transcriptome: Identification of cell-signalling, transcriptional control and immune-related genes from open reading frame expressed sequence tags (ORESTES). *Dev. Comp. Immunol.* **31**, 763-782, (2007).
149. Lockyer, A. E. *et al. Biomphalaria glabrata* transcriptome: cDNA microarray profiling identifies resistant- and susceptible-specific gene expression in haemocytes from snail strains exposed to *Schistosoma mansoni*. *BMC Genomics* **9**, 634-634, (2008).
150. Adema, C. M. *et al.* Differential transcriptomic responses of *Biomphalaria glabrata* (Gastropoda, Mollusca) to bacteria and metazoan parasites, *Schistosoma mansoni* and *Echinostoma paraensei* (Digenea, Platyhelminthes). *Mol. Immunol.* **47**, 849-860, (2010).
151. Deleury, E. *et al.* Specific versus non-specific immune responses in an invertebrate species evidenced by a comparative de novo sequencing study. *PLoS One* **7**, e32512, (2012).
152. Zhang, L., Li, L., Zhu, Y.-B., Zhang, G. & Guo, X. Transcriptome analysis reveals a rich gene set related to innate immunity in the Eastern oyster (*Crassostrea virginica*). *Marine Biotechnology* **16**, 17-33, (2014)
153. Zmasek, C. M., Zhang, Q., Ye, Y. & Godzik, A. Surprising complexity of the ancestral apoptosis network. *Genome Biol.* **8**, R226, (2007).
154. Colin, J., Gaumer, S., Guenal, I. & Mignotte, B. Mitochondria, Bcl-2 family proteins and apoptosomes: Of worms, flies and men. *Front. Biosci.* **14**, 4127-4137, (2009).
155. Valencia, C. A., Cotten, S. W. & Liu, R. Cleavage of BNIP-2 and BNIP-XL by caspases. *Biochem. Biophys. Res. Commun.* **364**, 495-501, (2007).
156. Rojas-Rivera, D. & Hetz, C. TMBIM protein family: Ancestral regulators of cell death. *Oncogene* **34**, 269-280, (2015)
157. Vandenabeele, P. & Bertrand, M. J. M. The role of the IAP E3 ubiquitin ligases in regulating pattern-recognition receptor signalling. *Nat. Rev. Immunol.* **12**, 833-844, (2012).

158. Almeida, A. P. & Neves, A. G. A. The hemoglobin of *Biomphalaria glabrata*: chemical composition and some physicochemical properties. *Biochim. Biophys. Acta* **371**, 140-146, (1974).
159. Oliver, L. Observations on vectors of *Schistosomiasis mansoni* kept out of water in the laboratory. *The Journal of Parasitology* **42**, 277-286, (1956).
160. de Jong-Brink, M., Bergamin-Sassen, M. & Soto, M. S. Multiple strategies of schistosomes to meet their requirements in the intermediate snail host. *Parasitology* **123**, S129-S141, (2001).
161. Hahn, U. K., Bender, R. C. & Bayne, C. J. Production of reactive oxygen species by hemocytes of *Biomphalaria glabrata*: Carbohydrate-specific stimulation. *Dev. Comp. Immunol.* **24**, 531-541, (2000).
162. Hahn UK, Bender RC, Bayne CJ Killing of *Schistosoma mansoni* sporocysts by hemocytes from resistant *Biomphalaria glabrata*: role of reactive oxygen species. *Journal of Parasitology* **87**, 292-299, (2001).
163. Mourão de M, M., Dinguirard, N., Franco, G. R. & Yoshino, T. P. Role of the endogenous antioxidant system in the protection of *Schistosoma mansoni* primary sporocysts against exogenous oxidative stress. *PLoS Negl. Trop. Dis.* **3**, e550, (2009).
164. Hahn, U. K., Bender, R. C. & Bayne, C. J. Involvement of nitric oxide in killing of *Schistosoma mansoni* sporocysts by hemocytes from resistant *Biomphalaria glabrata*. *The Journal of Parasitology* **87**, 785-778, (2001).
165. Bienert, G. P., Schjoerring, J. K. & Jahn, T. P. Membrane transport of hydrogen peroxide. *Biochim. Biophys. Acta* **1758**, 994-1003, (2006).
166. Bender, R. C., Broderick, E. J., Goodall, C. P. & Bayne, C. J. Respiratory burst of Biomphalaria glabrata hemocytes: Schistosoma mansoni-resistant snails produce more extracellular H2O2 than susceptible snails. *The Journal of Parasitology* **91**, 275-279, (2005).
167. Bender, R. C., Goodall, C. P., Blouin, M. S. & Bayne, C. J. Variation in expression of Biomphalaria glabrata SOD1: A potential controlling factor in susceptibility/resistance to Schistosoma mansoni. *Dev. Comp. Immunol*. **31**, 874-878, (2007).
168. Goodall, C. P., Bender, R. C., Broderick, E. J. & Bayne, C. J. Constitutive differences in Cu/Zn superoxide dismutase mRNA levels and activity in hemocytes of *Biomphalaria glabrata* (Mollusca) that are either susceptible or resistant to *Schistosoma mansoni* (Trematoda). *Mol. Biochem. Parasitol.* **137**, 321-328, (2004)
169. Trachootham, D., Lu, W., Ogasawara, M. A., Nilsa, R.-D. V. & Huang, P. Redox regulation of cell survival. *Antioxidants and Redox Signaling* **10**, 1343-1374, (2008).
170. Pietraforte, D. *et al.* Redox control of platelet functions in physiology and pathophysiology. *Antioxidants and Redox Signaling* **21**, 177-193, (2014).
171. Bellin, D., Asai, S., Delledonne, M. & Yoshioka, H. Nitric oxide as a mediator for defense responses. *Mol. Plant. Microbe Interact.* **26**, 271-277, (2013).
172. Varga, G., Gattorno, M., Foell, D. & Rubartelli, A. Redox distress and genetic defects conspire in systemic autoinflammatory diseases. *Nature Reviews Rheumatology*, (2015).
173. Larson, M. K., Bender, R. C. & Bayne, C. J. Resistance of *Biomphalaria glabrata* 13-16-R1 snails to *Schistosoma mansoni* PR1 is a function of haemocyte abundance and constitutive levels of specific transcripts in haemocytes. *Int. J. Parasitol.* **44**, 343-353, (2014).
174. Grasberger, H. & Refetoff, S. Identification of the maturation factor for dual oxidase: Evolution of an eukaryotic operon equivalent. *J. Biol. Chem.* **281**, 18269-18272, (2006).
175. Gorrini, C., Harris, I. S. & Mak, T. W. Modulation of oxidative stress as an anticancer strategy. *Nature Reviews Drug Discovery* **12**, 931-947, (2013)

176. Allocati, N., Federici, L., Masulli, M. & Di Ilio, C. Glutathione transferases in bacteria. *FEBS J.* **276**, 58-75, (2009).
177. Sheehan, D., Meade, G., Foley, V. M. & Dowd, C. A. Structure, function and evolution of glutathione transferases: Implications for classification of non-mammalian members of an ancient enzyme superfamily. *Biochem. J.* **360**, 1-16, (2001).
178. Udomsinprasert, R. *et al.* Identification, characterization and structure of a new Delta class glutathione transferase isoenzyme. *Biochem. J.* **388**, 763-771, (2005).
179. Oakley, A. Glutathione transferases: A structural perspective. *Drug Metab. Rev.* **43**, 138-151, (2011).
180. Schuliga, M., Chouchane, S. & Snow, E. T. Upregulation of glutathione-related genes and enzyme activities in cultured human cells by sublethal concentrations of inorganic arsenic. *Toxicol. Sci.* **70**, 183-192, (2002).
181. Brogden, K. A. Antimicrobial peptides: Pore formers or metabolic inhibitors in bacteria? *Nature Reviews Microbiology* **3**, 238-250, (2005).
182. Bulet, P., Stocklin, R. & Menin, L. Anti-microbial peptides: From invertebrates to vertebrates. *Immunol. Rev.* **198**, 169-184 (2004).
183. Nguyen, L. T., Haney, E. F. & Vogel, H. J. The expanding scope of antimicrobial peptide structures and their modes of action. *Trends Biotechnol.* **29**, 464-472, (2011).
184. Mitta, G., Vandenbulcke, F., Hubert, F., Salzet, M. & Roch, P. Involvement of mytilins in mussel antimicrobial defense. *J. Biol. Chem.* **275**, 12954-12962, (2000).
185. Mitta, G., Vandenbulcke, F. & Roch, P. Original involvement of antimicrobial peptides in mussel innate immunity. *FEBS Lett.* **486**, 185-190, (2000).
186. Schmitt, P. *et al.* The antimicrobial defense of the Pacific oyster, *Crassostrea gigas*. How diversity may compensate for scarcity in the regulation of resident/pathogenic microflora. *Front. Microbiol.* **3**, 160-160, (2012).
187. Hathaway, J. J. M., Adema, C. M., Stout, B. A., Mobarak, C. D. & Loker, E. S. Identification of protein components of egg masses indicates parental investment in immunoprotection of offspring by *Biomphalaria glabrata* (Gastropoda, Mollusca). *Dev. Comp. Immunol.* **34**, 425-435, (2010).
188. Baron, O. L. *et al.* Parental transfer of the antimicrobial protein LBP/BPI protects *Biomphalaria glabrata* eggs against oomycete infections. *PLoS Pathog.* **9**, e1003792, (2013).
189. Galinier, R. *et al.* Biomphalysin, a new β pore-forming toxin involved in *Biomphalaria glabrata* immune defense against *Schistosoma mansoni*. *PLoS Pathog.* **9**, e1003216, (2013).
190. Bouchut, A., Coustau, C., Gourbal, B. & Mitta, G. Compatibility in the *Biomphalaria glabrata*/*Echinostoma caproni* model: New candidate genes evidenced by a suppressive subtractive hybridization approach. *Parasitology* **134**, 575-588, (2007).
191. Obara, K. *et al.* Molecular cloning of the antibacterial protein of the giant African snail, *Achatina fulica* Ferussac. *Eur. J. Biochem.* **209**, 1-6, (1992).
192. Kamiya, H., Muramoto, K. & Yamazaki, M. Aplysianin-A, an antibacterial and antineoplastic glycoprotein in the albumen gland of a sea hare, *Aplysia kurodai*. *Experientia* **42**, 1065-1067, (1986).
193. Takamatsu, N., Shiba, T., Muramoto, K. & Kamiya, H. Molecular cloning of the defense factor in the albumen gland of the sea hare *Aplysia kurodai*. *FEBS Lett.* **377**, 373-376, (1995).
194. Ehara, T., Kitajima, S., Kanzawa, N., Tamiya, T. & Tsuchiya, T. Antimicrobial action of achacin is mediated by L-amino acid oxidase activity. *FEBS Lett.* **531**, 509-512, (2002).
195. Jung, S. *et al.* Hydramacin-1, structure and antibacterial activity of a protein from the basal metazoan Hydra. *J. Biol. Chem.* **284**, 1896-1905, (2009).

196. Morris, K.M. *et al.* Identification and analysis of divergent immune gene families within the Tasmanian devil genome. *BMC Genomics* **16,** 1017 (2015).
197. Destoumieux, D. *et al.* Penaeidins, a new family of antimicrobial peptides isolated from the shrimp *Penaeus vannamei* (Decapoda). *The Journal of Biological Chemistry* **272**, 28398-28406, (1997).
198. Tasiemski, A. *et al.* Molecular characterization of two novel antibacterial peptides inducible upon bacterial challenge in an annelid, the leech *Theromyzon tessulatum*. *J. Biol. Chem.* **279**, 30973-30982, (2004).
199. Vidal-Dupiol, J. *et al.* Innate immune responses of a scleractinian coral to vibriosis. *J. Biol. Chem.* **286**, 22688-22698, (2011).
200. Castro-Faria-Neto, H. C. *et al.* Pro-inflammatory activity of enterolobin: A haemolytic protein purified from seeds of the Brazilian tree *Enterolobium contortisiliquum*. *Toxicon* **29**, 1143-1150, (1991).
201. Sher, D. *et al.* Hydralysins, a new category of beta-pore-forming toxins in cnidaria. *J. Biol. Chem.* **280**, 22847-22855, (2005).
202. Szczesny, P. *et al.* Extending the aerolysin family: From bacteria to vertebrates. *PLoS One* **6**, e20349, (2011).
203. Xiang, Y. *et al.* Host-derived, pore-forming toxin-like protein and trefoil factor complex protects the host against microbial infection. *Proc. Natl. Acad. Sci. U.S.A.* **111**, 6702-6707, (2014).
204. Hanington, P. C., Lun, C. M., Adema, C. M. & Loker, E. S. Time series analysis of the transcriptional responses of *Biomphalaria glabrata* throughout the course of intramolluscan development of *Schistosoma mansoni* and *Echinostoma paraensei*. *Int J Parasitol* **40**, 819-831 (2010b).
205. Jablonka, E. & Lamb, M. J. *Evolution in Four Dimensions: Genetic, Epigenetic, Behavioral, and Symbolic Variation in the History of Life*. (The MIT Press, 2005).
206. Azzi, A., Cosseau, C. & Grunau, C. *Schistosoma mansoni*: Developmental arrest of miracidia treated with histone deacetylase inhibitors. *Exp. Parasitol.* **121**, 288-291, (2009)
207. Lister, R. & Ecker, J. R. Finding the fifth base: Genome-wide sequencing of cytosine methylation. *Genome Res.* **19**, 959-966, (2009).
208. Xi, Y. & Li, W. BSMAP: Whole genome bisulfite sequence MAPping program. *BMC Bioinformatics* **10**, 232, (2009).
209. Fneich, S. *et al.* 5-methyl-cytosine and 5-hydroxy-methyl-cytosine in the genome of *Biomphalaria glabrata*, a snail intermediate host of *Schistosoma mansoni*. *Parasit Vectors* **6**, 167-167, (2013).
210. Geyer, K. K. *et al.* Cytosine methylation regulates oviposition in the pathogenic blood fluke *Schistosoma mansoni*. *Nature Communications* **2**, 424-424, (2011).
211. Parra, G., Bradnam, K. & Korf, I. CEGMA: A pipeline to accurately annotate core genes in eukaryotic genomes. *Bioinformatics* **23**, 1061-1067, (2007).
212. Andersen, C. L., Jensen, J. L. & Ørntoft, T. F. Normalization of real-time quantitative reverse transcription-PCR data: A model-based variance estimation approach to identify genes suited for normalization, applied to bladder and colon cancer data sets. *Cancer Res.* **64**, 5245-5250, (2004).
213. Bannister, A. J. & Kouzarides, T. Reversing histone methylation. *Nature* **436**, 1103-1106, (2005).
214. Zeng, W., Ball, A. R. & Yokomori, K. HP1: Heterochromatin binding proteins working the genome. *Epigenetics* **5**, 287-292, (2010).

215. Pal-Bhadra, M. *et al.* Heterochromatic silencing and HP1 localization in *Drosophila* are dependent on the RNAi machinery. *Science* **303**, 669-672, (2004).
216. Verdel, A., Vavasseur, A., Le Gorrec, M. & Touat-Todeschini, L. Common themes in siRNA-mediated epigenetic silencing pathways. *The International Journal of Developmental Biology* **53**, 245-257, (2009).
217. Grewal, S. I. S. RNAi-dependent formation of heterochromatin and its diverse functions. *Curr. Opin. Genet. Dev.* **20**, 134-141, (2010).
218. Pélisson, A., Sarot, E., Payen-Groschêne, G. & Bucheton, A. A novel repeat-associated small interfering RNA-mediated silencing pathway downregulates complementary sense gypsy transcripts in somatic cells of the *Drosophila* ovary. *J. Virol.* **81**, 1951-1960, (2007).
219. Manzano-Román, R. & Siles-Lucas, M. MicroRNAs in parasitic diseases: potential for diagnosis and targeting. *Mol. Biochem. Parasitol.* **186**, 81-86, (2012).
220. de Souza Gomes, M., Muniyappa, M. K., Carvalho, S. G., Guerra-Sá, R. & Spillane, C. Genome-wide identification of novel microRNAs and their target genes in the human parasite *Schistosoma mansoni*. *Genomics* **98**, 96-111, (2011).
221. Hofacker, I. L. RNA secondary structure analysis using the Vienna RNA package. *Current Protocols in Bioinformatics*, 12.12.11-12.12.16, (2009).
222. Burge, S. W. *et al.* Rfam 11.0: 10 years of RNA families. *Nucleic Acids Res.* **41**, D226-D232, (2013).
213. Jiang, P. *et al.* MiPred: Classification of real and pseudo microRNA precursors using random forest prediction model with combined features. *Nucleic Acids Res.* **35**, W339-W344, (2007).
224. Zhang, B., Pan, X., Cannon, C. H., Cobb, G. P. & Anderson, T. A. Conservation and divergence of plant microRNA genes. *The Plant Journal* **46**, 243-259, (2006).
225. Larkin, M. A. *et al.* Clustal W and Clustal X version 2.0. *Bioinformatics* **23**, 2947-2948, (2007).
226. Crooks, G. E., Hon, G., Chandonia, J.-M. & Brenner, S. E. WebLogo: A sequence logo generator. *Genome Res.* **14**, 1188-1190, (2004).
227. Saitou, N. & Nei, M. The neighbor-joining method: A new method for reconstructing phylogenetic trees. *Mol. Biol. Evol.* **4**, 406-425, (1987).
228. Enright, A. J. *et al.* MicroRNA targets in *Drosophila*. *Genome Biol.* **5**, R1, (2004).
229. John, B. *et al.* Human MicroRNA targets. *PLoS Biol.* **2**, e363-e363, (2004).
230. Campo-Paysaa, F., Sémon, M., Cameron, R. A., Peterson, K. J. & Schubert, M. microRNA complements in deuterostomes: Origin and evolution of microRNAs. *Evol Dev* **13**, 15-27, (2011).
231. Mandemakers, W. *et al.* Co-regulation of intragenic microRNA miR-153 and its host gene Ia-2 β: Identification of miR-153 target genes with functions related to IA-2β in pancreas and brain. *Diabetologia* **56**, 1547-1556, (2013).
232. Fabian, M. R., Sonenberg, N. & Filipowicz, W. Regulation of mRNA translation and stability by microRNAs. *Annu. Rev. Biochem.* **79**, 351-379, (2010).
233. Kozomara, A. & Griffiths-Jones, S. miRBase: Annotating high confidence microRNAs using deep sequencing data. *Nucleic Acids Res.* **42**, D68-73, (2014).
234. Huang, Y., Niu, B., Gao, Y., Fu, L. & Li, W. CD-HIT Suite: A web server for clustering and comparing biological sequences. *Bioinformatics* **26**, 680-682, (2010)
235. Chang, C.-C. & Lin, C.-J. LIBSVM: A library for support vector machines. *ACM Transactions on Intelligent Systems and ...* **2**, Article 27, (2011).
236. Leclercq, M., Diallo, A. B. & Blanchette, M. Computational prediction of the localization of microRNAs within their pre-miRNA. *Nucleic Acids Res.* **41**, 7200-7211, (2013).

237. Gabriel, U. I., Mirela, S. & Ionel, J. Quantification of mucoproteins (glycoproteins) from snails mucus, *Helix aspersa* and *Helix pomatia*. *Journal of Agroalimentary Processes and Technologies* **17**, 410-413, (2011).
238. Marxen, J. C. & Becker, W. Calcium binding constituents of the organic shell matrix from the freshwater snail *Biomphalaria glabrata*. *Comparative Biochemistry and Physiology. Part B, Biochemistry & Molecular Biology* **127**, 235-242, (2000).
239. Volk, T., Wang, S., Rotstein, B. & Paululat, A. Matricellular proteins in development: Perspectives from the *Drosophila* heart. *Matrix Biol.* **37**, 162-166, (2014).
240. Britton, C., Winter, A. D., Gillan, V. & Devaney, E. microRNAs of parasitic helminths – Identification, characterization and potential as drug targets. *International Journal for Parasitology: Drugs and Drug Resistance* **4**, 85-94, (2014).
241. Lotshaw, D. P. & Jacklet J. W. "Involvement of protein synthesis in circadian clock of *Aplysia* eye." *Am. J. Physiol.* **250**(1 Pt 2): R5-17, (1986).
242. Raju, U., Yeung S. J. & Eskin, A. Involvement of proteins in light resetting ocular circadian oscillators of *Aplysia*. *Am. J. Physiol*. **258**, R256-262, (1990)
243. Khalsa, S. B., Whitmore D. & Block G. D. Stopping the circadian pacemaker with inhibitors of protein synthesis. *Proc. Natl. Acad. Sci. U.S.A.* **89**: 10862-10866, (1992).
244. Khalsa, S. B., Whitmore D., Bogart B. & Block G. D. Evidence for a central role of transcription in the timing mechanism of a circadian clock. *Am. J. Physiol.* **271**: C1646-1651, (1996).
245. McMahon, D. G. and Block G. D. The *Bulla* ocular circadian pacemaker. I. Pacemaker neuron membrane potential controls phase through a calcium-dependent mechanism. *J. Comp. Physiol. A* **161**, 335-346, (1987).
246. Michel, S., Geusz, M. E., Zaritsky J. J. and Block G. D. Circadian rhythm in membrane conductance expressed in isolated neurons. *Science* **259**, 239-241, (1993).
247. Block, G. D., Geusz, M., Khalsa, S. B., Michel, S. & Whitmore, D. in *Progress in Brain Research Volume 111* Vol. 111 93-102 (Elsevier, 1996).
248. Page, T. L. & Hudson D. J. Circadian organization in *Aplysia californica*. *Fed Proc* **38,** 2580-2582, (1979).
249. Connor, K. M. & Gracey A. Y. Circadian cycles are the dominant transcriptional rhythm in the intertidal mussel *Mytilus californianus*. *Proc. Natl. Acad. Sci. U.S.A.* **108,** 16110-16115, (2011).
250. Tran, D., *et al.* The toxic dinoflagellate *Alexandrium minutum* disrupts daily rhythmic activities at gene transcription, physiological and behavioral levels in the oyster *Crassostrea gigas*. *Aquat. Toxicol.* **158**: 41-49, (2015).
251. Pairett, A. N. & Serb J. M. De novo assembly and characterization of two transcriptomes reveal multiple light-mediated functions in the scallop eye (Bivalvia: Pectinidae). *PLoS ONE* **8**, e69852. (2013).
252. Buhr, E. D. & Takahashi J. S. Molecular components of the Mammalian circadian clock. *Hand.b Exp. Pharmacol.* **217**), 3-27 (2013).
253. Constance, C. M., Green, C. B., Tei, H. & Block, G. D. *Bulla gouldiana* period exhibits unique regulation at the mRNA and protein levels. *J. Biol. Rhythms* **17**, 413-427, (2002).
254. Siwicki, K. K., Strack S., Rosbash M., Hall J. C. & Jacklet J. W. An antibody to the *Drosophila* period protein recognizes circadian pacemaker neurons in *Aplysia* and *Bulla*. *Neuron* **3,** 51-58, (1989).
255. Ukai-Tadenuma, M. *et al.* Delay in feedback repression by cryptochrome 1 is required for circadian clock function. *Cell* **144**, 268-281, (2011).

256. Kumar, S., Stecher, G., Peterson, D. & Tamura, K. MEGA-CC: computing core of molecular evolutionary genetics analysis program for automated and iterative data analysis. *Bioinformatics* **28**, 2685-2686. . (2012).

257. Jaumouille, E., Machado Almeida P., Stahli P., Koch R.& Nagoshi E. Transcriptional regulation via nuclear receptor crosstalk required for the *Drosophila* circadian clock. *Curr. Biol.* **25,** 1502-1508, (2015).

258. Ledent, V. & Vervoort, M. The basic helix-loop-helix protein family: Comparative genomics and phylogenetic analysis. *Genome Res.* **11**, 754-770, (2001).

259. Wolmarans, C. T., de Kock, K. N., Strauss, H. D. & Bornman, M. Daily emergence of *Schistosoma mansoni* and *S. haematobium* cercariae from naturally infected snails under field conditions. *J. Helminthol.* **76**, 273-277, (2002)

260. Bogéa, T., Favre, T. C., Rotenberg, L., Silva, H. S. & Pieri, O. S. Circadian pattern of cercarial emergence in *Schistosoma mansoni* (Platyhelminthes:Digenea) from isolated *Biomphalaria glabrata*. *Chronobiol. Int.* **13**, 93-101, (1996)

261. Hien, N. & Disko, R. Investigations on periodic phenomena in the snail *Biomphalaria glabrata*. *Z. Parasitenkd.* **64**, 217-231, (1981).

262. Sokolove, P. G., Beiswanger, C. M., Prior, D. J. & Gelperin, A. A circadian rhythm in the locomotive behaviour of the giant garden slug *Limax maximus*. *J. Exp. Bio.* **66**, 47-64, (1977).

263. Steinauer, M. L. & Bonner, K. M. Host susceptibility is altered by light intensity after exposure to parasites. *The Journal of Parasitology* **98**, 1052-1054, (2012).

264. Adamson, K.J. *et al.* Molecular insights into land snail neuropeptides through transcriptome and comparative gene analysis. *BMC Genomics*. **17**;16:308. (2015)..

265. Brunak, S., Engelbrecht, J., & Knudsen, S. Prediction of human mRNA donor and acceptor sites from the DNA sequence. *J. Mol. Biol.* **220**, 49-65. (1991).

266. Floyd, P.D., Li, L., Moroz, T.P. & Sweedler, J.V. Characterization of peptides from Aplysia using microbore liquid chromatography with matrix-assisted laser desorption/ionization time-of-flight mass spectrometry guided purification. *J. Chromatogr. A* **830**, 105-113. (1999).

267. Hamano, K., Awaji, M. & Usuki, H. cDNA structure of an insulin-related peptide in the Pacific oyster and seasonal changes in the gene expression. *J. Endocrinol.* **187**, 55-67. (2005).

268. Veenstra, J.A. Neurohormones and neuropeptides encoded by the genome of *Lottia gigantea*, with reference to other mollusks and insects. *Gen. Comp. Endocrinol.* **167**, 86-103. (2010).

269. Bendtsen JD *et al.* Feature-based prediction of non-classical and leaderless protein secretion. *Protein Eng Des Sel.* **17**:349-356. (2004).

270. Southey, B.R. et al. NeuroPred: a tool to predict cleavage sites in neuropeptide precursors and provide the masses of the resulting peptides. *Nucleic Acids Res.* **34**, W267-272. (2006).

271. Beitz, E. TEXshade: shading and labeling of multiple sequence alignments using LATEX2 epsilon. *Bioinformatics*, **16**, 135-139. (2000).

272. Ren, J. *et al.* XDOG 1.0: illustrator of protein domain structures. Cell Res 19, 271-273 (2009).

273. Koene, J.M. et al., Male accessory gland protein reduces egg laying in a simultaneous hermaphrodite. *PLoS ONE* 5: e10117 (2010).

274. Nakadera, Y. *et al.* Receipt of seminal fluid proteins causes reduction of male investment in a simultaneous hermaphrodite. *Current Biology* **24**, 1-4, (2014).

275. Fernandes, D., Loi, B. & Porte, C. Biosynthesis and metabolism of steroids in molluscs. *The Journal of Steroid Biochemistry and Molecular Biology* **127**, 189-195, (2011).

276. Kaur, S. *et al.* The nuclear receptors of *Biomphalaria glabrata* and *Lottia gigantea*: Implications for developing new model organisms. *PLoS One* **10**, e0121259, (2015).

277. Thornton, J. W., Need, E. & Crews, D. Resurrecting the ancestral steroid receptor: Ancient origin of estrogen signaling. *Science* **301**, 1714-1717, (2003).
278. Laguerre, M. & Veenstra, J. A. Ecdysone receptor homologs from molluscs, leeches and a polychaete worm. *FEBS Lett.* **584**, 4458-4462, (2010).
279. Manning, G., Plowman, G. D., Hunter, T. & Sudarsanam, S. Evolution of protein kinase signaling from yeast to man. *Trends Biochem. Sci.* **27**, 514-520, (2002).
280. Kanehisa, M. *et al.* Data, information, knowledge and principle: Back to metabolism in KEGG. *Nucleic Acids Res.* **42**, D199-D205, (2014).
281. Jeffrey, K. L., Camps, M., Rommel, C. & Mackay, C. R. Targeting dual-specificity phosphatases: Manipulating MAP kinase signalling and immune responses. *Nature Reviews Drug Discovery* **6**, 391-403, (2007).
282. Andersen, J.N. et al., Structural and evolutionary relationships among protein tyrosine phosphatase domains. Mol, Cell Biol. 21,7117-7136, (2001) .
283. Shi Y. Serine/threonine phosphatases: mechanism through structure. Cell. 2009 Oct 30;139(3):468-84. doi: 10.1016/j.cell.2009.10.006. Review. PubMed PMID: 19879837.
284. Humphries, J. E. & Yoshino, T. P. Regulation of hydrogen peroxide release in circulating hemocytes of the planorbid snail *Biomphalaria glabrata*. *Dev. Comp. Immunol.* **32**, 554-562, (2008).
285. Zelck, U. E., Gege, B. E. & Schmid, S. Specific inhibitors of mitogen-activated protein kinase and PI3-K pathways impair immune responses by hemocytes of trematode intermediate host snails. *Dev. Comp. Immunol.* **31**, 321-331, (2007).
286. Zahoor, Z., Davies, A. J., Kirk, R. S., Rollinson, D. & Walker, A. J. Disruption of ERK signalling in *Biomphalaria glabrata* defence cells by *Schistosoma mansoni*: Implications for parasite survival in the snail host. *Dev. Comp. Immunol.* **32**, 1561-1571, (2008).
287. Cripps, R. M. & Olson, E. N. Control of cardiac development by an evolutionarily conserved transcriptional network. *Dev. Biol.* **246**, 14-28, (2002).
288. Medioni, C. *et al.* The fabulous destiny of the *Drosophila* heart. *Curr. Opin. Genet. Dev.* **19**, 518-525, (2009).
289. Bodmer, R. The gene *tinman* Is required for specification of the heart and visceral muscles in *Drosophila*. *Development* **118**, 719-729, (1993).
290. Azpiazu, N. & Frasch, M. *tinman* and *bagpipe*: Two homeo box genes that determine cell fates in the dorsal mesoderm of *Drosophila*. *Genes Dev.* **7**, 1325-1340, (1993).
291. Lyons, I. *et al.* Myogenic and morphogenetic defects in the heart tubes of murine embryos lacking the homeo box gene *Nkx2-5*. *Genes Dev.* **9**, 1654-1666, (1995).
292. Elliott, D. A. *et al.* A tyrosine-rich domain within homeodomain transcription factor Nkx2-5 is an essential element in the early cardiac transcriptional regulatory machinery. *Development* **133**, 1311-1322, (2006).
293. Navet, S., Bassaglia, Y., Baratte, S., Martin, M. & Bonnaud, L. Somatic muscle development in *Sepia officinalis* (Cephalopoda - Mollusca): A new role for NK4. *Dev. Dyn.* **237**, 1944-1951, (2008).
294. Holland, N. D., Venkatesh, T. V., Holland, L. Z., Jacobs, D. K. & Bodmer, R. *AmphiNk2-tin*, an amphioxus homeobox gene expressed in myocardial progenitors: Insights into evolution of the vertebrate heart. *Dev. Biol.* **255**, 128-137, (2003).
295. Harvey, R. P. NK-2 homeobox genes and heart development. *Dev. Biol.* **178**, 203-216, (1996).
296. Fyrberg, E. A., Mahaffey, J. W., Bond, B. J. & Davidson, N. Transcripts of the six *Drosophila* actin genes accumulate in a stage- and tissue-specific manner. *Cell* **33**, 115-123, (1983).

297. Fryberg, E. A., Bond, B. J., Hershey, N. D., Mixter, K. S. & Davidson, N. The actin genes of *Drosophila*: Protein coding regions are highly conserved but intron positions are not. *Cell* **24**, 107-116, (1981).

298. Mounier, N., Gouy, M., Mouchiroud, D. & Prudhomme, J. C. Insect muscle actins differ distinctly from invertebrate and vertebrate cytoplasmic actins. *J. Mol. Evol.* **34**, 406-415, (1992).

299. Carlini, D. B., Reece, K. S. & Graves, J. E. Actin gene family evolution and the phylogeny of coleoid cephalopods (Mollusca: Cephalopoda). *Mol. Biol. Evol.* **17**, 1353-1370, (2000).

300. Sin, F. Y. T., Bryant, M. J. & Johnstone, A. Molecular evolution and phylogeny of actin genes in *Haliotis* species (Mollusca: Gastropoda). *Zool. Stud.* **46**, 734-745, (2007).

301. DesGroseillers, L., Auclair, D., Wickham, L. & Maalouf, M. A novel actin cDNA is expressed in the neurons of *Aplysia californica*. *Biochim. Biophys. Acta* **1217**, 322-324, (1994).

302. Trapnell, C. *et al.* Differential gene and transcript expression analysis of RNA-seq experiments with TopHat and Cufflinks. *Nat. Protoc.* **7**, 562-578, (2012).

303. Krzywinski, M. *et al.* Circos: An information aesthetic for comparative genomics. *Genes Dev.* **19**, 1639-1645, (2009).

304. Darzentas, N. Circoletto: Visualizing sequence similarity with Circos. *Bioinformatics* **26**, 2620-2621, (2010).

305. Takeuchi, T. *et al.* Draft genome of the pearl oyster *Pinctada fucata*: A platform for understanding bivalve biology. *DNA Res.* **19**, 117-130, (2012).

306. Mann, K. & Edsinger, E. The *Lottia gigantea* shell matrix proteome: Re-analysis including MaxQuant iBAQ quantitation and phosphoproteome analysis. *Proteome Science* **12**, 28-28, (2014).

307. Marie, B. *et al.* Proteomic analysis of the organic matrix of the abalone *Haliotis asinina* calcified shell. *Proteome Science* **8**, 54, (2010).

308. Mann, K. & Jackson, D. J. Characterization of the pigmented shell-forming proteome of the common grove snail *Cepaea nemoralis*. *BMC Genomics* **15**, 249, (2014).

309. Jurka, J. *et al.* Repbase Update, a database of eukaryotic repetitive elements. *Cytogenet. Genome Res.* **110**, 462-467, (2005).

310. Smit, A. F. A., Hubley, R. & Green, P. http://www.repeatmasker.org/. unpublished data Current Version: open-4.0.5 (RMLib:20140131 & Dfam:1.3)

311. Price, A.L, Jones, N. C. & Pevzner P. A. *De novo* identification of repeat families in large genomes. *Bioinformatics* **21** (Suppl 1), i351–i358. (2005).

312. Feschotte, C. et al. Exploring repetitive DNA landscapes using REPCLASS, a tool that automates the classification of transposable elements in eukaryotic genomes. *Genome Biology and Evolution.* **1**: 205-220 (2009).

313. Morgulis, A., Gertz, E. M., Schäffer, A. A. & Agarwala, R. WindowMasker: Window-based masker for sequenced genomes. *Bioinformatics* **22**, 134-141, (2006).

314. Benson, G Tandem repeats finder: a program to analyze DNA sequences. *Nucleic Acids Res.* **27**:573–580 (1999).

315. *A*lbertin, C. B. *et al.* The octopus genome and the evolution of cephalopod neural and morphological novelties. *Nature* **524**, 220-224, (2015).

316. Panchin, Y. & Moroz, L. L. Molluscan mobile elements similar to the vertebrate Recombination-Activating Genes. *Biochem. Biophys. Res. Commun.* **369**, 818-823, (2008).

317. Berriman, M. *et al.* The genome of the blood fluke *Schistosoma mansoni*. *Nature* **460**, 352-358, (2009).

318. Knight, M. *et al.* in *Mobile Genetic Elements in Metazoan Parasites* (ed Paul J. Brindley) 49-60 (Landes Bioscience, 2009).
319. Gilbert, C., Schaack, S., Pace, J. K., Brindley, P. J. & Feschotte, C. A role for host-parasite interactions in the horizontal transfer of transposons across phyla. *Nature* **464**, 1347-1350, (2010).
320. Pace, J. K., Gilbert, C., Clark, M. S., Feschotte, C. Repeated horizontal transfer of a DNA transposon in mammals and other tetrapods. *Proc Natl Acad Sci U S A.* **105**, 17023-17028, (2008).
321. Wu, T. D. & Watanabe, C. K. GMAP: A genomic mapping and alignment program for mRNA and EST sequences. *Bioinformatics* **21**, 1859-1875, (2005).
322. Charbonnel, N., Angers, B., Razatavonjizay, R., Brémond, P. & Jarne, P. Microsatellite variation in the freshwater snail *Biomphalaria pfeifferi*. *Mol. Ecol.* **9**, 1006-1007, (2000).
